# Supplementary material for: Disulfide modification and thiol protection via tris(trimethylsilyl)silane-mediated hydrosilylation of disulfides
Source: Nat Commun. 2026 Apr 1;17:4705. doi: 10.1038/s41467-026-71313-2 (PMC13212980; doi:10.1038/s41467-026-71313-2)
Supplement: Supplementary file 1 — Supplementary Information [file 41467_2026_71313_MOESM1_ESM.pdf]

Supplementary Information

Disulfide Modification and Thiol Protection via Tris(trimethylsilyl)silane-Mediated Hydrosilylation of Disulfides

Ying Zhang[1], Kejun Lin[1], Zhenming Zang[1], Jianhui Chen[2] and Tingshun Zhu[1]*

[1]Key Laboratory of Bioinorganic and Synthetic Chemistry of Ministry of Education, Guangdong Provincial Key Laboratory of Chiral Molecule and Drug Discovery, School of Chemistry, IGCME, Sun Yat-Sen University, Guangzhou 510275, China.

[2]Division of Gastrointestinal Surgery Center, the First Affiliated Hospital of Sun Yat-sen University, Guangzhou, Guangdong, 510080 P. R. China

*Corresponding author. E-mail: zhutshun@mail.sysu.edu.cn

Table of Contents

1. General Information 1

2. General Procedure 2

3. Synthesis of starting materials 3

4. Synthetic applications 8

5. Conditions Screening 13

6. Stability comparison of the protecting group 16

7. Unsuccessful substrates 16

8. Mechanism study 17

9. Characterization of Products 20

10. X-Ray Crystallographic Data 49

11. Copies of ^{1}H NMR, ^{13}C NMR, and ^{19}F NMR spectra of products 55

12. References cited in the SI 153

1. General Information

Commercial reagents were purchased from TCI, J&K, 3A Chemicals, Accela, Macklin, Energy, Bide, Meryer, CIL, or Adamas and used without further purification. The solvents used in the experiments were all purchased as anhydrous solvents and used directly. All reactions were carried out with oven-dried glassware. Analytical thin-layer chromatography was performed on 0.20 mm silica gel HSGF-254 plates (Huanghai, China), and visualized under 254 nm UV light. Column chromatography was performed on 200-300 mesh silica gel (General-Reagent, China) and sephadex LH-20 (Macklin).

^{1}H, ^{19}F, and ^{13}C NMR spectra were recorded on Bruker Ascend 400 MHz or 600 MHz spectrometers. Chemical shifts were recorded in parts per million (ppm, δ) relative to chloroform (for ^{1}H NMR, δ = 7.26 ppm, singlet; for ^{13}C NMR, δ = 77.16 ppm, triplet). ^{1}H NMR splitting patterns are designated as singlet (s), doublet (d), triplet (t), quartet (q), dd (doublet of doublets); m (multiplets), etc. All first-order splitting patterns were assigned on the basis of the appearance of the multiplet. Splitting patterns that could not be easily interpreted are designated as multiplet (m) or broad (br).

High-resolution mass spectra of new compounds were recorded on Thermo Q Exactive GC-Orbitrap (EI) and a Bruker timsTOF (APCI). Fourier transform infrared (FT-IR) spectra were recorded on the PerkinElmer Frontier spectrometer and reported in wavenumbers (cm^{-1}). Steady-state fluorescence spectra were measured on an Edinburgh Instruments FLS1000 spectrometer with a xenon lamp. Its X-ray diffraction data were collected on an Agilent Gemini Ultra diffractometer (CuKα radiation, Agilent, Oxfordshire, UK). Liquid chromatography-mass spectrometry (LC-MS) analysis was performed on a Shimadzu LC-MS-2020 system equipped with an electrospray ionization (ESI) source operating in either positive or negative ion mode. Gas chromatography-mass spectrometry (GC-MS) was recorded on GC-MS-QP2010 SE (Shimadzu). The Electron Spin Resonance (ESR) spectra of the trapped radical were recorded by ESR5000.

2. General Procedure

General procedure for the preparation of silyl sulfides 2a–2z18

Reactions were carried out in a 4 mL glass vial equipped with a magnetic stir bar. The disulfide substrate (0.10 mmol) and solvent (MTBE, toluene, or DMF/MTBE) were added, followed by tris(trimethylsilyl)silane (TTMSS, 2–100 equiv.) without inert atmosphere protection. For substrates **1a**–**1g** and **1j**–**1m**, the reaction mixture in MTBE was concentrated under reduced pressure at 35 °C for 5 min using a rotary evaporator. For substrates **1h** and **1i**, the reaction mixture was stirred in MTBE (0.1 M) at 35 °C for 6 h. For substrate **1n**, the reaction was conducted in toluene (0.1 M) at 35 °C for 6 h. For substrates **1o**, **1v**, **1z**, and **1z6**–**1z17**, the mixture was stirred in MTBE (0.1 M) with TTMSS (4 equiv.) at 35 °C for 12–24 h. For substrates **1p**, **1q**, **1r**, **1t**, **1x**, **1y**, and **1z5**, the reaction mixture was irradiated with 450 nm blue LEDs (6 W) and at room temperature in MTBE (0.1 M) with TTMSS (2 equiv.) for 12 h. For substrates **1s** and **1u**, the reaction was carried out under 450 nm irradiation (6 W) and in MTBE (0.1 M) with TTMSS (4 equiv.) at room temperature for 12 h. For substrate **1w**, the reaction was conducted in MTBE (0.5 M) with TTMSS (2 equiv.) at 35 °C for 8 h. For substrates **1z1**–**1z4**, the mixture was stirred in MTBE (0.1 M) with TTMSS (2 equiv.) at 35 °C for 6–12 h. For substrate **1z18**, the reaction was performed under 450 nm irradiation in a DMF/MTBE (3:1, 0.006 M) solvent mixture with TTMSS (100 equiv.) at room temperature for 24 h. The progress of the reactions was monitored by TLC. Upon completion, the solvent was removed under reduced pressure, and the crude residue was purified by flash column chromatography on silica gel (petroleum ether/ethyl acetate as eluent) to afford the corresponding silyl sulfides **2a**–**2z18**.

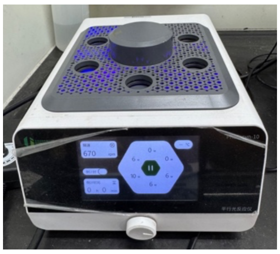

Supplementary Fig. 1. Reaction setup for the photochemical experiments.

3. Synthesis of starting materials

A. Synthesis of aryl disulfide

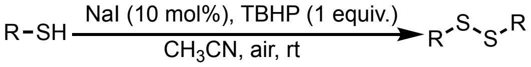

The aryl disulfide was synthesized according to a modified literature procedure.[1]

A 50 mL round-bottom flask equipped with a magnetic stir bar was charged with sodium iodide (10 mol%) and acetonitrile (5 mL), followed by the addition of thiophenol (1 mmol). *tert*-Butyl hydroperoxide (TBHP, 1 equiv.) was then added dropwise. The reaction mixture was stirred at room temperature for 6 hours. After completion, the mixture was concentrated in vacuo. The residue was then purified by column chromatography (silica gel, petroleum ether as eluent) to afford the desired aryl disulfides **1c**, **1d**, **1e**, **1f**, **1g**, **1h**, **1j**, **1l**, **1m**, **1n**, and **1t**.

B. Synthesis of 1,4-dihydrobenzo[2][1,2]dithiine

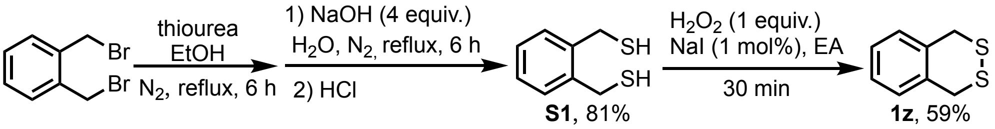

S1, 81% **1z**, 59%

The 1,4-dihydrobenzo[*d*][1,2]dithiine was synthesized according to a modified literature procedure.[3]

Step 1: Synthesis of o-xylene-α,α'-dithiol (S1)

A 35 mL pressure tube was charged with α,α'-dibromo-o-xylene (2 mmol) and thiourea (2.5 equiv.) in ethanol (10 mL). The solution was heated to 100 °C for 6 h under a nitrogen atmosphere. After completion, the mixture was removed under reduced pressure to afford a solid residue. To this residue was added NaOH (8 mL, 1 M aqueous solution), and the mixture was refluxed for an additional 6 hours under nitrogen. Upon cooling, the pH was adjusted to neutral by dropwise addition of HCl (2 M aqueous solution). The aqueous phase was extracted with dichloromethane (3 × 10 mL), and the combined organic extracts were dried over anhydrous sodium sulfate, filtered, and concentrated in vacuo. The residue was purified by flash column chromatography, providing o-xylene-α,α'-dithiol (275.4 mg, 81% yield).

Step 2: Synthesis of 1,4-dihydrobenzo[*d*][1,2]dithiine (S2)

In a 25 mL round-bottom flask, o-xylene-α,α'-dithiol (1 mmol) was dissolved in ethyl acetate (5 mL, 0.2 M). To this stirred solution was added 30% H_2O_2 (1 equiv.). After stirring at room temperature for 30 min, the mixture was concentrated in vacuo. The residue was purified by flash column chromatography (petroleum ether as eluent) to yield 1,4-dihydrobenzo[*d*][1,2]dithiine (**S2**) as a white solid (99 mg, 59% yield). **^{1}H NMR** (400 MHz, $CDCl_3$) δ 7.21 – 7.14 (m, 2H), 7.12 – 7.04 (m, 2H), 4.07 (s, 4H). **^{13}C NMR** (100 MHz, $CDCl_3$) δ 133.0, 130.3, 126.9, 34.7.

C. Synthesis of (±)-trans-1,2-dithiane-4,5-diyl diacetate

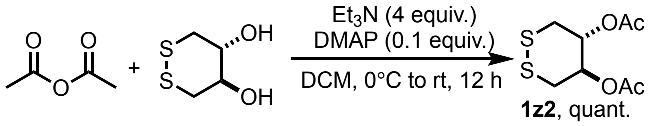

The compound was synthesized according to the literature procedure.[4]

A flame-dried 50 mL round-bottom flask equipped with a magnetic stir bar was charged with (±)-trans-1,2-dithiane-4,5-diol (300 mg, 2 mmol) and dichloromethane (10 mL). The solution was cooled to 0 °C in an ice bath, followed by sequential addition of 4-dimethylaminopyridine (24 mg, 0.2 mmol, 0.1 equiv.) and triethylamine (0.81 g, 8 mmol, 4.0 equiv.). Acetic anhydride (510 mg, 5 mmol, 2.5 equiv.) was then added dropwise over 5 min. The reaction mixture was allowed to warm to room temperature and stirred for 12 h. Upon completion, the mixture was diluted with dichloromethane (10 mL) and washed sequentially with 1 M aqueous HCl (10 mL) and brine (10 mL). The organic layer was dried over anhydrous sodium sulfate, filtered, and concentrated in vacuo. The crude product was purified by flash column chromatography (silica gel, petroleum ether/ethyl acetate = 10:1 to 4:1, v/v) to afford the desired compound as a white solid (472 mg, quantitative yield). The ^{1}H and ^{13}C NMR spectra were in full agreement with the literature values.

D. Synthesis of (±)-trans-4,5-bis(benzyloxy)-1,2-dithiane

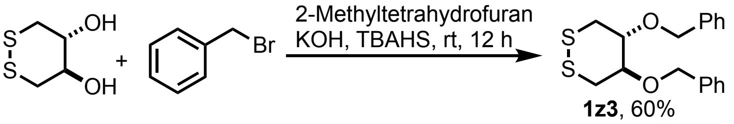

The compound was synthesized according to a modified literature procedure.[5]

A 50 mL round-bottom flask equipped with a magnetic stir bar was charged with (±)-trans-1,2-dithiane-4,5-diol (300 mg, 2 mmol) and benzyl bromide (524 μL, 4.4 mmol) in 2-methyl-THF (25 mL). The solution was stirred at room temperature, followed by the addition of KOH (6 mL, 5 M aqueous solution, 30 mmol) and tetrabutylammonium hydrogen sulfate (TBAHS, 170 mg, 0.5 mmol). The reaction mixture was stirred overnight at room temperature. Upon completion, the mixture was diluted with ethyl acetate (50 mL) and washed with brine (3 × 20 mL). The organic phase was separated, dried over anhydrous sodium sulfate, filtered, and concentrated in vacuo. The crude product was purified by flash column chromatography (silica gel, petroleum ether/ethyl acetate = 10:1, v/v) to afford **1z3** in 60% yield. The ^{1}H and ^{13}C NMR spectra were in full agreement with the literature values.[6]

E. Synthesis of (±)-trans-2,2-dimethyltetrahydro-[1,2]dithiino[4,5-*d*][1,3]dioxole

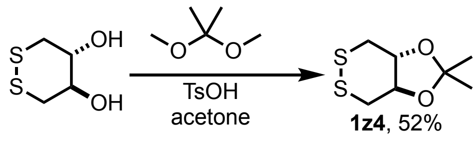

The compound was synthesized according to a modified literature procedure.[7]

A flame-dried 25 mL round-bottom flask equipped with a magnetic stir bar was charged with (±)-trans-1,2-dithiane-4,5-diol (300 mg, 2 mmol) and acetone (10 mL). To this solution was sequentially added 2,2-dimethoxypropane (290 μL, 2 mmol) and *p*-toluenesulfonic acid (*p*-TsOH, 103 mg, 30 mol%). The reaction mixture was stirred at room temperature for 10 min, then quenched by dropwise addition of saturated aqueous $NaHCO_3$ (5 mL) at 0 °C. The mixture was removed under reduced pressure, and the residue was diluted with ethyl acetate (20 mL). The organic layer was separated and washed sequentially with water (10 mL) and brine (10 mL), dried over anhydrous sodium sulfate, filtered, and concentrated in vacuo. The crude product was purified by

flash column chromatography (silica gel, hexane/ethyl acetate = 3:1 to 2:1, v/v) to afford compound **1z4** (200 mg, 52% yield). ^{1}H and ^{13}C NMR spectra were in full agreement with the literature values.

F. Synthesis of lipoic acid ester derivatives

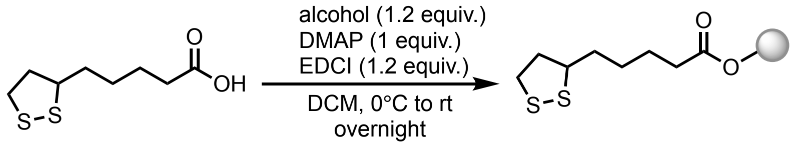

These compounds were synthesized according to the literature procedure.[8]

A flame-dried 25 mL round-bottom flask equipped with a magnetic stir bar was charged with lipoic acid (206 mg, 1 mmol, 1.0 equiv.) and 4-dimethylaminopyridine (DMAP, 122 mg, 1 mmol, 1.0 equiv.) in anhydrous dichloromethane (10 mL). The mixture was cooled to 0 °C in an ice bath, followed by dropwise addition of the corresponding alcohol derivative (1.2 mmol, 1.2 equiv.) under a nitrogen atmosphere. After stirring for 5 min, N-(3-dimethylaminopropyl)-N'-ethylcarbodiimide hydrochloride (EDCI, 230 mg, 1.2 mmol, 1.2 equiv.) was added in one portion. The reaction mixture was allowed to warm to room temperature and stirred for 12 h under nitrogen. Upon completion, the mixture was concentrated in vacuo, and the crude product was purified by flash column chromatography (silica gel, petroleum ether/ethyl acetate = 40:1 to 2:1, v/v) to afford the desired lipoic acid ester derivative **1z7** to **1z17** (yields: 99–75%).

G. Synthesis of N'-(*tert*-Butyloxy)carbonyl-l-cystine methyl ester

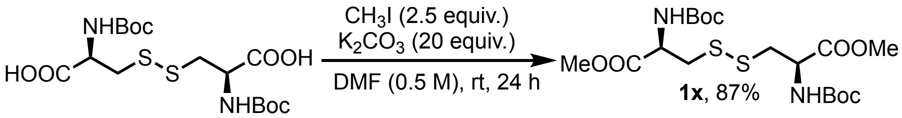

The compound was synthesized according to a modified literature procedure.[9]

A flame-dried 50 mL Schlenk flask equipped with a magnetic stir bar was charged with $(Boc\text{-}Cys\text{-}OH)_2$ (2.2 g, 5 mmol) and anhydrous DMF (10 mL). Potassium carbonate (13.8 g, 100 mmol, 20 equiv.) was added, followed by dropwise addition of iodomethane (779 µL, 12.5 mmol, 2.5 equiv.) at room temperature under a nitrogen atmosphere. The reaction mixture was stirred at ambient temperature for 24 h. Upon completion (monitored by TLC), the mixture was filtered through a Büchner funnel to

remove insoluble potassium carbonate. The filtrate was diluted with ethyl acetate (20 mL) and washed with water (3 × 20 mL). The combined organic layers were dried over anhydrous sodium sulfate, filtered, and concentrated in vacuo. The crude product was purified by flash column chromatography (silica gel, hexanes/ethyl acetate = 1:1, v/v) to afford compound **1x** as a white solid (2.04 g, 87% yield). ^{1}H and ^{13}C NMR spectra were in full agreement with the literature values.

H. Synthesis of 1,1,1,3,3,3-hexamethyl-2-(trimethylsilyl)trisilane-2-*d*

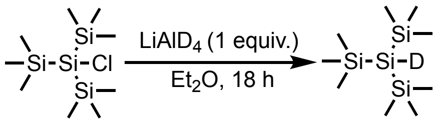

The compound was synthesized according to a modified literature procedure.[10]

A flame-dried 25 mL Schlenk flask equipped with a magnetic stir bar was charged with lithium aluminium deuteride (210 mg, 5 mmol) in anhydrous diethyl ether (2 mL) under nitrogen atmosphere. A solution of 2-chloro-1,1,1,3,3,3-hexamethyl-2-(trimethylsilyl)trisilane (1.41 g, 5 mmol) in anhydrous diethyl ether (5 mL) was added dropwise via syringe at 0 °C. The reaction mixture was allowed to warm to room temperature and stirred for 18 h under nitrogen. The reaction was quenched by careful dropwise addition of aqueous sodium hydroxide (1 M) at 0 °C, followed by stirring at room temperature for 15 min. The resulting mixture was filtered through a pad of Celite, and the filter cake was washed with diethyl ether (3 × 5 mL). The combined filtrates were concentrated in vacuo. The crude product was purified by flash column chromatography (silica gel, petroleum ether as eluent) to afford the deuterated silane (99% D) as a colorless oil. ^{1}H, ^{13}C, and ^{29}Si NMR spectra were in full agreement with the literature values.

4. Synthetic applications

A. Gram-scale synthesis

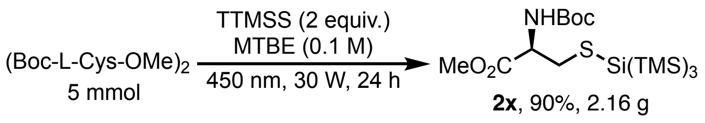

A 100 mL round-bottom flask equipped with a magnetic stir bar was charged with (Boc-Cys-OMe)$_2$ (5.0 mmol, 2.34 g) and MTBE (0.1 M, 50 mL). Tris(trimethylsilyl)silane (TTMSS, 2.0 equiv. 2.48 g, 10 mmol) was added, and the reaction mixture was irradiated with 450 nm blue LEDs (30 W) at room temperature for 24 h. After completion of the reaction (monitored by TLC), the solvent was removed under reduced pressure using a rotary evaporator. The crude residue was purified by flash column chromatography on silica gel (petroleum ether/ethyl acetate as eluent) to afford compound **2x** as a white solid in 90% yield (2.16 g, 4.5 mmol).

B. Deprotection of the Boc group

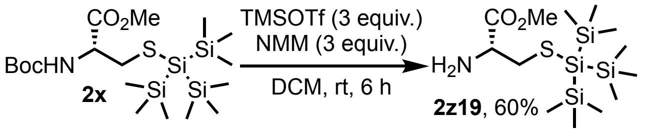

A 10 mL flame-dried Schlenk flask equipped with a magnetic stir bar was charged with 4-Methylmorpholine (NMM, 30.3 mg, 0.3 mmol, 3 equiv.) and **2x** (48.1 mg, 0.10 mmol) in anhydrous DCM (1 mL). TMSOTf (66.7 mg, 0.3 mmol, 3 equiv.) was added dropwise via syringe at room temperature under a nitrogen atmosphere. The reaction mixture was stirred for 6 hours at ambient temperature (monitored by TLC). Upon completion, the mixture was concentrated in vacuo. The crude product was purified by flash column chromatography (silica gel, petroleum ether/ethyl acetate = 10/1, v/v) to afford **2z19** as a colorless oil (22.8 mg, 60% yield). The relatively low yield may be attributed to the inherent instability of the product, which is susceptible to air oxidation under ambient conditions, as evidenced by the gradual color change from colorless to yellow upon standing in solution.

C. Synthesis of polypeptide

Step 1: Synthesis of dipeptide Boc-Val-Ser-OMe (2z20)

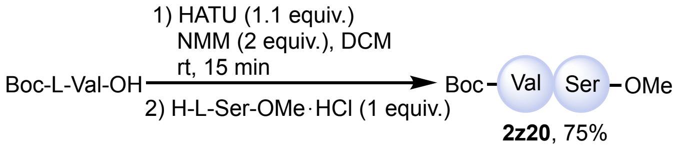

A 50 mL flame-dried Schlenk flask equipped with a magnetic stir bar was charged with Boc-L-Val-OH (1.09 g, 5.0 mmol) and HATU (2.09 g, 5.5 mmol, 1.1 equiv.) in anhydrous dichloromethane (20 mL) under a nitrogen atmosphere. 4-Methylmorpholine (NMM, 1.01 g, 1.10 mL, 10 mmol, 2 equiv.) was added dropwise via syringe at room temperature, and the resulting mixture was stirred for 15 min to activate the carboxylic acid. H-L-Ser-OMe•HCl (777.9 mg, 5.0 mmol, 1.0 equiv.) was then added in one portion. The reaction mixture was stirred at ambient temperature for 24 h (monitored by TLC). Upon completion, the mixture was concentrated in vacuo. The residue was dissolved in ethyl acetate (30 mL) and washed sequentially with saturated aqueous $NaHCO_3$ (20 mL) and water (3 × 20 mL). The combined organic layers were dried over anhydrous sodium sulfate, filtered, and concentrated in vacuo. The residue was purified by flash column chromatography (silica gel, petroleum ether/ethyl acetate = 2/1, v/v) to afford the title compound **2z20** as a white solid (1.2 g, 75% yield).

Step 2: Synthesis of tripeptide 2z21

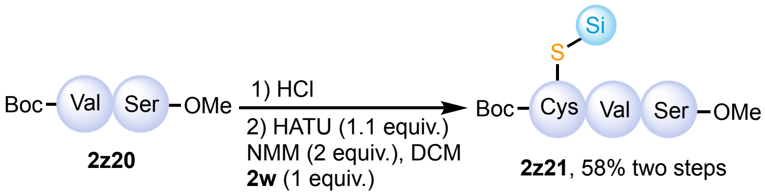

A 10 mL flame-dried Schlenk flask equipped with a magnetic stir bar was charged with Boc-Val-Ser-OMe (636 mg, 2.0 mmol) and THF (2 mL) under a nitrogen atmosphere. HCl (4 M in 1,4-dioxane, 2 mL) was added dropwise via syringe at an ice bath, and the resulting mixture was stirred at 0 °C for 1 hour. Upon complete deprotection, the mixture was concentrated in vacuo to afford H-Val-Ser-OMe•HCl as a white solid (638 mg, 90% yield).

A 50 mL flame-dried Schlenk flask equipped with a magnetic stir bar was charged with **2w** (1.09 g, 2 mmol) and HATU (836 mg, 2.2 mmol, 1.1 equiv.) in anhydrous dichloromethane (10 mL) under a nitrogen atmosphere. 4-Methylmorpholine (NMM, 404 mg, 439 μL, 4 mmol, 2 equiv.) was added dropwise via syringe at room temperature, and the resulting mixture was stirred for 15 min to activate the carboxylic acid. The freshly prepared H-Val-Ser-OMe•HCl was then added in one portion. The reaction mixture was stirred at ambient temperature for 24 h (monitored by TLC). Upon completion, the mixture was concentrated in vacuo. The residue was dissolved in ethyl acetate (20 mL) and washed sequentially with saturated aqueous $NaHCO_3$ (10 mL) and water (3 × 10 mL). The combined organic layers were dried over anhydrous sodium sulfate, filtered, and concentrated in vacuo. The residue was purified by flash column chromatography (silica gel, petroleum ether/ethyl acetate = 2/1, v/v) to afford the title compound **2z21** as a colorless oil (773.7 mg, 58% yield, two steps).

Step 3: Sequential desilylation and oxidation

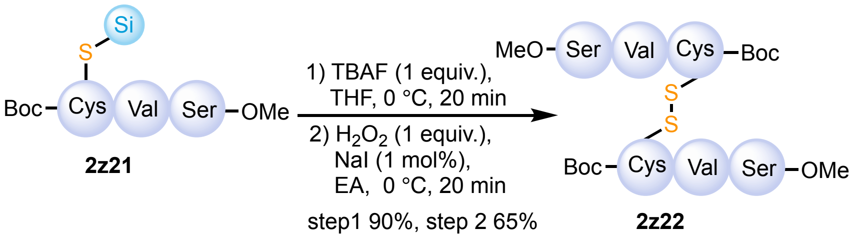

A 10 mL flame-dried Schlenk flask equipped with a magnetic stir bar was charged with **2z21** (333.5 mg, 0.5 mmol) and THF (2 mL) under a nitrogen atmosphere. TBAF (1 M in THF, 0.5 mL, 1 equiv.) was added dropwise via syringe at an ice bath, and the resulti1lng mixture was stirred at 0 °C for 20 min. Upon complete deprotection, the mixture was concentrated in vacuo to afford the desilylated product of **2z21** as an oil, which was used directly in the next step without further purification (90% purity by ^{1}H NMR confirmed).

A 10 mL flame-dried Schlenk flask equipped with a magnetic stir bar was charged with the prepared desilylated product of **2z21** and sodium iodide (0.75 mg, 1mol%) in 1 mL of ethyl acetate under a nitrogen atmosphere. 30% of H_2O_2 (1 equiv.) was added dropwise via syringe at an ice bath, and the resulting mixture was stirred at 0 °C for 20

min. Upon complete deprotection, the mixture was concentrated in vacuo, and the residue was purified by flash column chromatography (silica gel, dichloromethane/methanol = 20/1, v/v) to afford the compound **2z22** as a colorless oil (247.8 mg, 59% yield, two steps).

D. Thiol-disulfide exchange

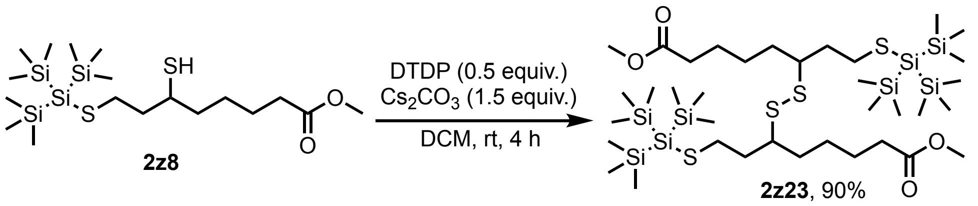

A 4 mL glass vial equipped with a magnetic stir bar was charged with Cs_2CO_3 (24.4 mg, 1.5 equiv.) and 1,2-di(pyridin-2-yl)disulfane (5.5 mg, 0.025 mmol, 0.5 equiv.) in 1 mL of DCM. **2z8** (23.4 mg, 0.05 mmol, 1 equiv.) was added without inert atmosphere protection. The reaction mixture was stirred at room temperature for 4 hours. Upon completion, the mixture was concentrated in vacuo and purified by flash column chromatography (silica gel, PE/EA = 25/1, v/v, as eluent) to afford **2z23** as a colorless oil (21.2 mg, 90% yield).

E. Thia-Michael addition of 2z7

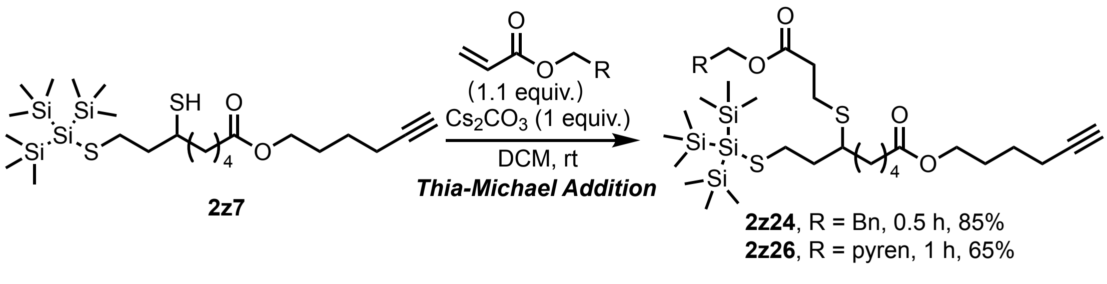

A 4 mL glass vial equipped with a magnetic stir bar was charged with Cs_2CO_3 (32.6 mg, 1 equiv.) and **2z7** (53.4 mg, 0.10 mmol) in 1 mL of DCM. Benzyl acrylate (17.8 mg, 0.11 mmol, 1.1 equiv.) or pyren-1-ylmethyl acrylate (31.5 mg, 0.11 mmol, 1.1 equiv.) was added without inert atmosphere protection. The reaction mixture was stirred at room temperature for 30 min or 1 hour. Upon completion, the mixture was concentrated in vacuo and purified by flash column chromatography (silica gel, PE/EA = 15/1, v/v, as eluent) to afford **2z24** as a colorless oil (59.1 mg, 85% yield) and **2z26**

as a colorless oil (53.3 mg, 65% yield).

F. CuAAC reaction

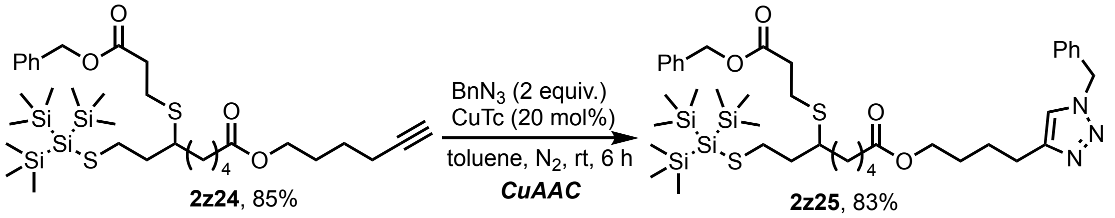

The compound was synthesized according to the literature procedure.[11] A 15 mL flame-dried Schlenk flask equipped with a magnetic stir bar was charged with Copper(I) thiophene-2-carboxylate hydrate (CuTc, 1.9 mg, 0.01 mmol, 20 mol%) and **2z24** (34.8 mg, 0.05 mmol) in anhydrous toluene (1 mL). A solution of (azidomethyl)benzene (13.3 mg, 0.10 mmol, 2.0 equiv.) in anhydrous toluene (0.5 mL) was added dropwise via syringe at room temperature under a nitrogen atmosphere. The reaction mixture was stirred for 6 hours at ambient temperature (monitored by TLC). Upon completion, the mixture was concentrated in vacuo. The crude product was purified by flash column chromatography (silica gel, petroleum ether/ethyl acetate = 3/1, v/v) to afford **2z25** as a colorless oil (34.3 mg, 83% yield).

5. Conditions Screening

To a 4 mL glass vial equipped with a magnetic stir bar were added the solvent (0.1 M) and disulfide substrate **1a** (0.10 mmol). TTMSS was then introduced without inert atmosphere protection. After stirring the reaction mixture at room temperature for 30 min, the solution was concentrated under reduced pressure using a rotary evaporator. 0.10 mmol of 1,3,5-trimethoxybenzene and $CDCl_3$ (0.5 mL) were added to the residue for ^{1}H NMR analysis.

Supplementary Table 1. Optimization of aryl disulfide thiosilylation with TTMSS[a]

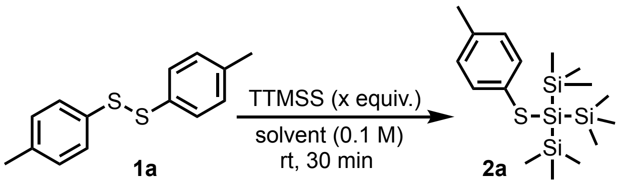

Entry	Variation from conditions	Yield of **2a** (%)[b]
1	none	quant. (99%[c])
2	DCM instead of MTBE	18
3	THF instead of MTBE	quant.
4	acetone instead of MTBE	quant.
5	Et_2O instead of MTBE	98
6	CH_3CN instead of MTBE	quant.
7	$PhCH_3$ instead of MTBE	quant.
8	DCE instead of MTBE	82
9	EA instead of MTBE	98
10	H_2O:MTBE = 1:1 (v/v) instead of MTBE	92
11	1 equiv. of TTMSS	70
12	1.2 equiv. of TTMSS	88
13	1.5 equiv. of TTMSS	98
14	With K_2CO_3 (1 equiv.), 9 h	34
15	With DABCO (1 equiv.), 9 h	28
16	With TEA (1 equiv.), 9 h	24
17	With TEMPO (3 equiv.)	14
18[d]	35 °C instead of rt, 5 min	quant.

[a]Conditions: **1a** (0.10 mmol), TTMSS (2 equiv.), MTBE (1 mL), rt, 30 min. [b]^{1}H NMR yields using 1,3,5-trimethoxybenzene as the internal standard. [c]Isolated yield. [d]After **1a** and TTMSS were added to the MTBE, the mixture was concentrated under reduced pressure at 35 °C for 5 min using a rotary evaporator. TTMSS = tris(trimethylsilyl)silane, TEMPO = 2,2,6,6-tetramethyl-1-piperinedinyloxys, MTBE = 2-methoxy-2-methylpropane.

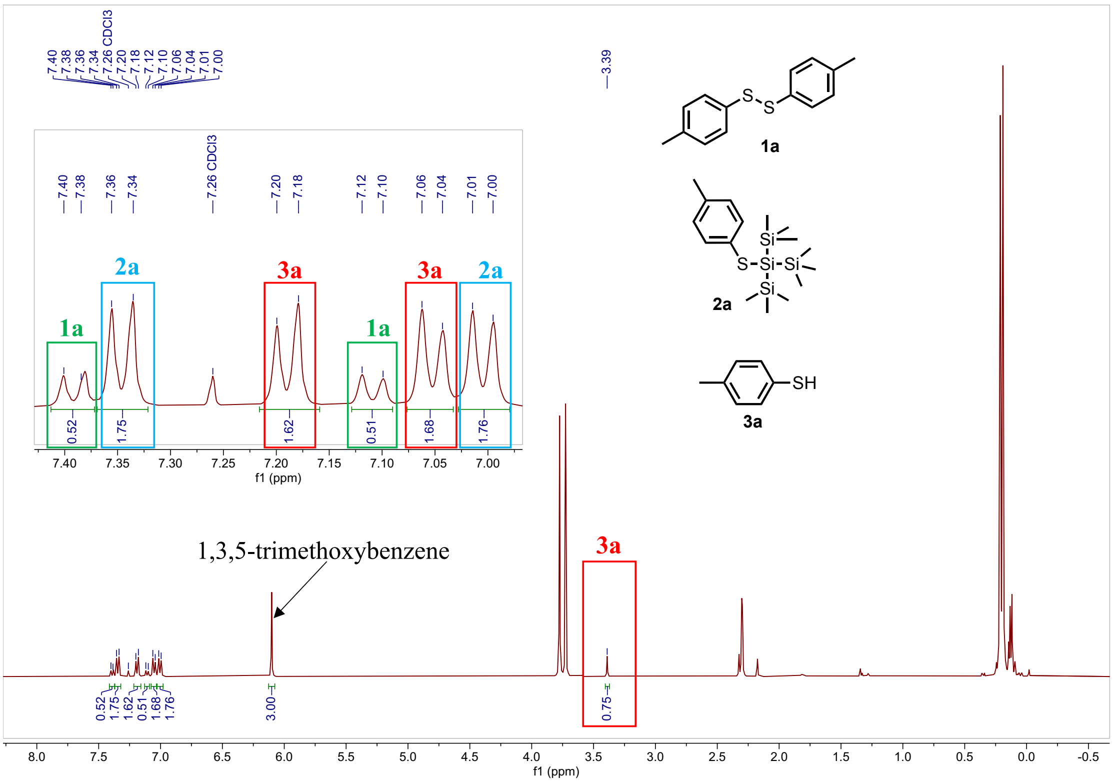

Supplementary Fig. 2. Crude ^{1}H NMR spectrum of the reaction in dichloroethane. 1,3,5-trimethoxybenzene as an internal standard.

Supplementary Table 2. Oxidant Screening[a]

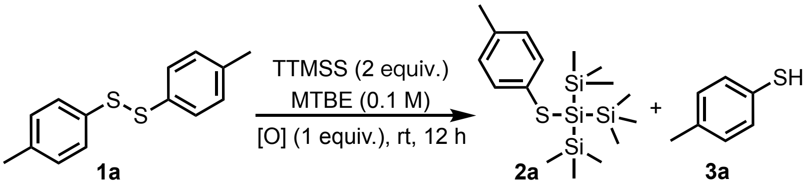

Entry	[O]	Yield of **2a** (%)[b]	Yield of **3a** (%)[b]	s.m. (%)[b]
1	$Cu(NO_3)_2$•$3H_2O$	24	0	54
2	$Cu(acac)_2$	37	0	39
3	$Cu(OTf)_2$	0	0	97
4	CuO	4	0	90
5	$Cu(OAc)_2$	28	0	51
6	I_2	0	5	88
7	$CuSO_4$	26	7	32
8	DDQ	0	0	100
9	HFIP	17	24	38
10	S_8	0	0	95

[a]Conditions: **1a** (0.10 mmol), TTMSS (2 equiv.), MTBE (0.1 M), [O] (1 equiv.), rt, 12 h. [b]^{1}H NMR yields using 1,3,5-trimethoxybenzene as the internal standard. Yields are based on RSSR.

Supplementary Table 3. Optimization of alkyl disulfide thiosilylation with TTMSS[a]

To a 4 mL glass vial equipped with a magnetic stir bar were added the MTBE (0.1 M) and disulfide substrate **1o** (0.10 mmol). TTMSS was then introduced without inert atmosphere protection. After the mixture was stirred for an appropriate period, the solution was concentrated under reduced pressure using a rotary evaporator. 0.10 mmol of 1,3,5-trimethoxybenzene and $CDCl_3$ (0.5 mL) were added to the residue for ^1H NMR analysis.

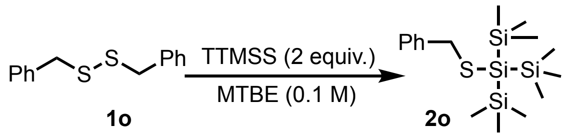

Entry	Variation from conditions	Yield of **2o** (%)[b]
1	rt, 24 h	6
2	35 °C, 24 h	78
3	70 °C, 10 h	93[c]
4	35 °C, 24 h, TTMSS (3 equiv.)	81
5	35 °C, 24 h, TTMSS (4 equiv.)	85 (82[c])

[a]Conditions: **1o** (0.10 mmol), TTMSS (2 equiv.), MTBE (1 mL). [b]NMR yields using 1,3,5-trimethoxybenzene as the internal standard. [c] Isolated yield.

Supplementary Table 4. Substrate yields under varied conditions[a]

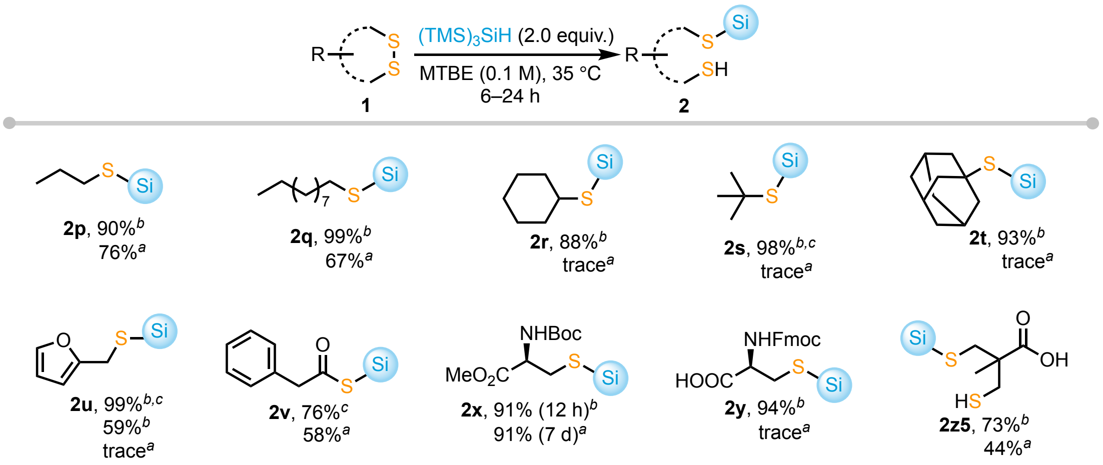

[a]The reactions were performed with 1 (0.10 mmol), TTMSS (2.0 equiv.), MTBE (0.1 M), 35 °C, Isolated yields are shown. [b]450 nm, 6 W, rt. [c]TTMSS (4.0 equiv.) was used.

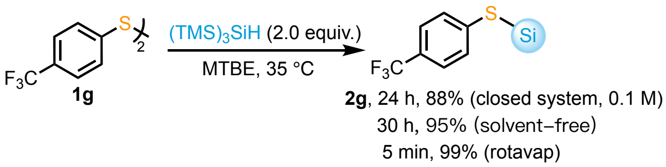

Supplementary Fig. 3. Comparison of sealed-vial, solvent-free and rotary evaporation conditions. Under standard conditions (MTBE, 35 °C, 0.1 M) in a 4 mL sealed vial, substrate **1g** was converted to the corresponding thiosilane in 88% isolated yield after 24 h. The solvent-free reaction proceeded more slowly, affording 95% yield after 30 h. In contrast, under rotary evaporation at 35 °C, near-quantitative conversion (99%) was achieved within approximately 5 min.

6. Stability comparison of the protecting group

Supplementary Table 5. Stability comparison of OTMS, OTBS, and S-Si(TMS)3 protecting group[a]

Conditions	*p*-Tol-OTMS	*p*-Tol-OTBS	*p*-Tol-S-Si(TMS)3
Silica gel/MeOH, rt	30 min (0%)	24 h (>95%)	24 h (>95%)
HCl (1 M), 20% H2O, rt	5 min (0%)	2 h (8%)	2 h (42%)
HCl (4 M), 20% H2O, rt	5 min (0%)	10 min (0%)	10 min (6%)
TBAF (1 M in THF), rt	3 min (0%)	10 min (0%)	15 min (0%)

[a]General conditions: Substrate (0.05 mmol). For silica gel stability tests: silica gel (50 mg), MeOH (1.0 mL). For acidic conditions: HCl (1 M or 4 M in 1,4-dioxane, 1.0 mL) with H_2O (0.2 mL). For fluoride-mediated deprotection: TBAF (1 M in THF, 1.0 equiv.), THF (1 mL). Reactions were conducted at room temperature. The yields were isolated recovery yields. *p*-Tol = 4-methylphenyl, TMS = trimethylsilyl, TBS = *tert*-butyldimethylsilyl.

7. Unsuccessful substrates

Supplementary Table 6. Unsuccessful substrates[a]

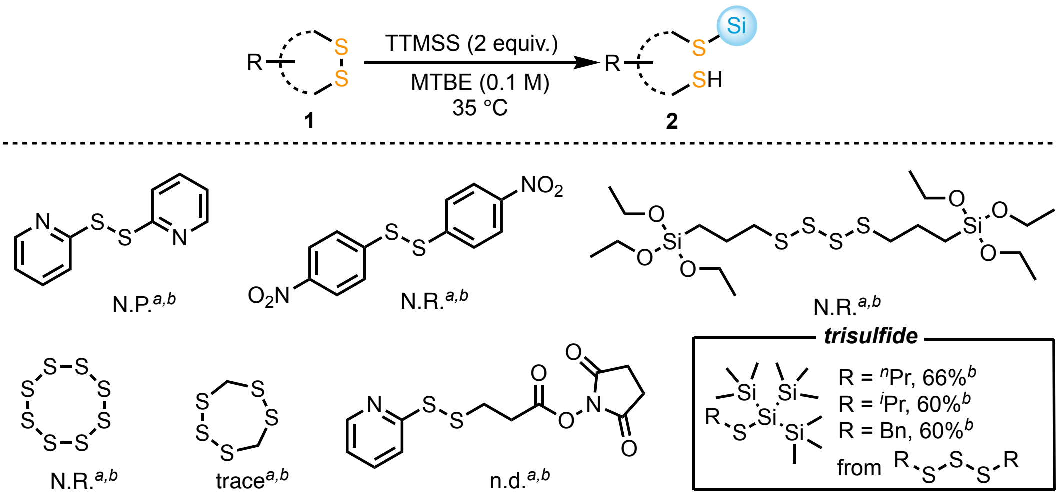

[a]Reaction conditions: **1** (0.10 mmol), TTMSS (2 equiv.), MTBE (0.1 M), 35 °C. Isolated yields are

shown. ^{b}450 nm, 6 w, room temperature.

8. Mechanism study

A. Radical trapping experiment with TEMPO

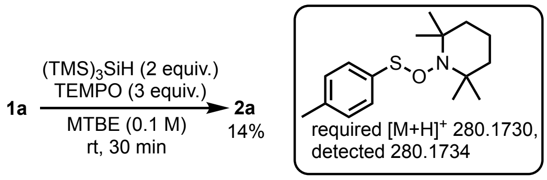

A 4 mL glass vial equipped with a magnetic stir bar was charged with 1,2-di-p-tolyldisulfane (24.6 mg, 0.10 mmol) and 2,2,6,6-tetramethylpiperidin-1-oxyl (TEMPO, 46.8 mg, 0.30 mmol, 3 equiv.) dissolved in MTBE (1 mL). Tris(trimethylsilyl)silane (TTMSS, 2 equiv.) was added under ambient atmosphere. The reaction mixture was stirred at room temperature for 30 min. Analysis by ^{1}H NMR revealed a 14% yield after workup, and HRMS confirmed the presence of an arylthio-TEMPO adduct. These observations suggest a side reaction involving radical trapping, leading to the formation of a stable adduct rather than the desired transformation.

B. Spin-trapping experiment

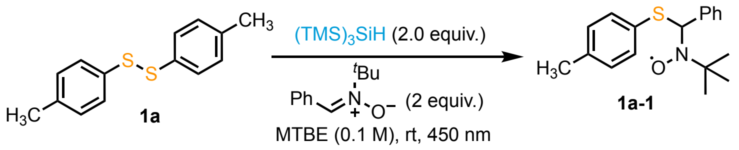

The Electron Spin Resonance (ESR) spectra of the trapped radical were recorded by ESR5000. The ESR spectra were obtained using the following parameters: Microwave power 10 mW, Sweep width: 0.2 mT. The simulation of the ESR spectra was using Easyspin version 6.0.6 and the garlic function was used to output the simulation spectra.

The spin trapping experiment was performed via the following procedure:

A 4 mL glass vial was charged with 1a (0.05 mmol, 1.0 equiv.) and N-*tert*-Butyl-alpha-phenylnitrone (PBN) (0.10 mmol, 2.0 equiv.). MTBE (0.5 mL) and TTMSS (0.10 mmol, 2.0 equiv.) were then added by syringe. The mixture was stirred until the solids had

completely dissolved. The resulting solution was transferred to a 3 mm quartz tube and irradiated for 1 min. The ESR spectrum was recorded immediately after irradiation. The spin-trapping result is shown in Supplementary Fig. 4.

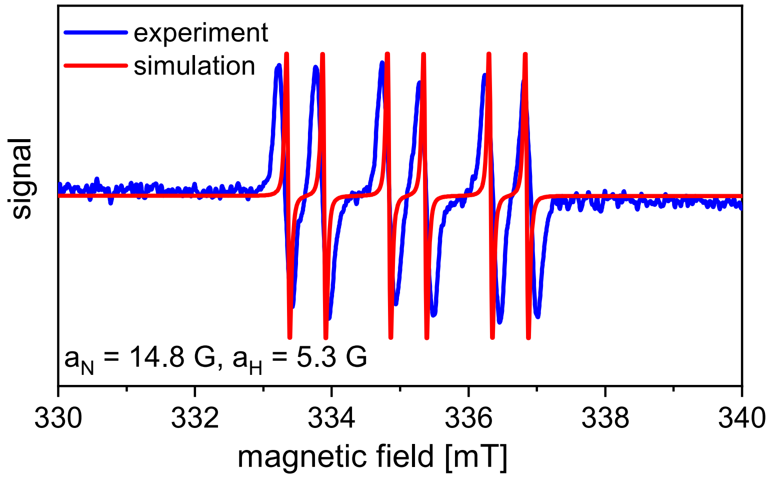

Supplementary Fig. 4. EPR spin-trapping experiment showing the PBN-thiyl radical adduct (blue line: experimental; red line: simulated). Radical **1a-1**: g = 2.0053, a_N = 14.8 G, a_H = 5.3 G.

C. Kinetic isotope effect (KIE) experiment

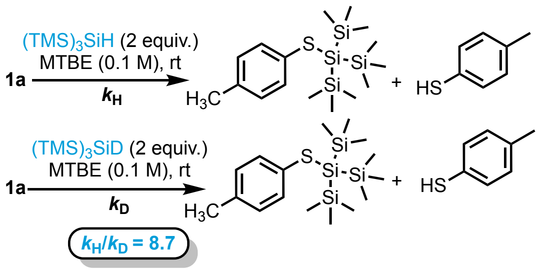

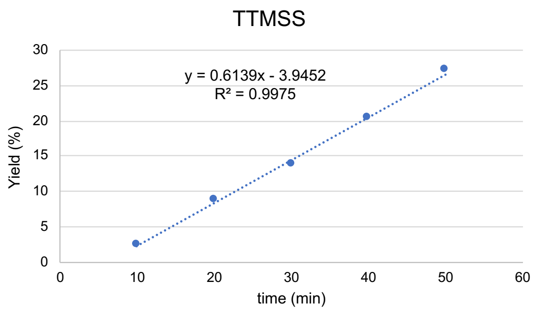

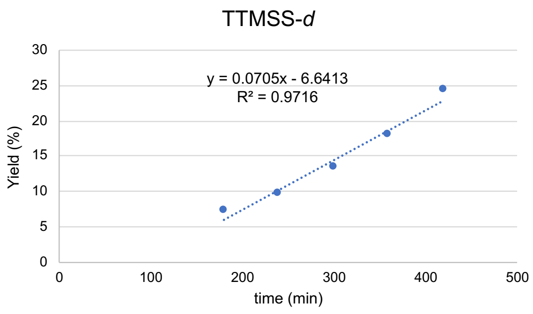

A 4 mL glass vial equipped with a magnetic stir bar was charged with MTBE (0.1 M) and 1,2-di-*p*-tolyldisulfane (12.3 mg, 0.05 mmol). Tris(trimethylsilyl)silane or Tris(trimethylsilyl)silane-*d* (2 equiv.) was added without inert atmosphere protection. Reaction progress was monitored by periodic sampling (10 μL aliquots), with each sample immediately diluted with chloroform (1.0 mL) for GC-MS analysis. Kinetic

analysis revealed a primary isotope effect ($k_H/k_D = 8.7$), confirming Si-H bond cleavage as the rate-determining step.

D. Fluorescence Spectra of TTMSS and cyclohexyldisulfide

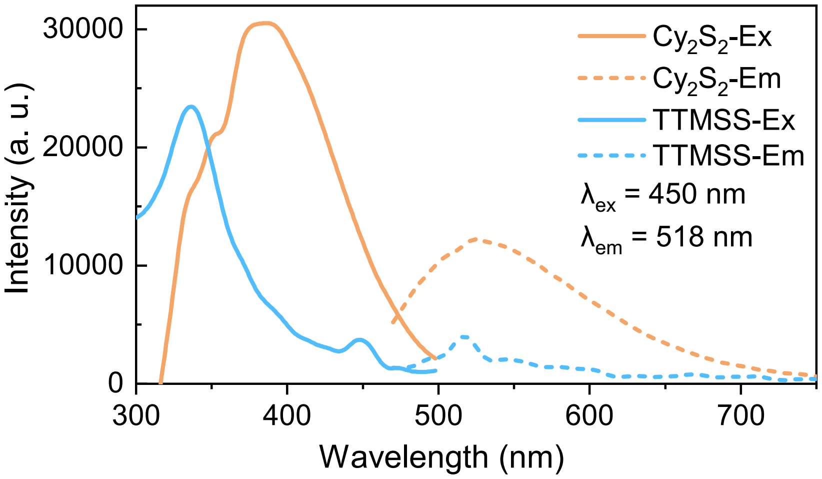

Supplementary Fig. 5. Fluorescence spectra of TTMSS and cyclohexyldisulfide (Cy_2S_2) in MTBE (λ_{ex} = 450 nm and λ_{em} = 518 nm). Solid lines denote excitation (Ex) spectra and dashed lines denote emission (Em) spectra. Intensity is given in arbitrary units (a. u.).

E. Proposed mechanism

We propose two possible mechanistic pathways—a stepwise radical mechanism and a concerted radical pathway—based on the observed kinetic isotope effect (KIE = 8.7), radical trapping experiments, and fluorescence spectral analysis.

(a) stepwise pathway

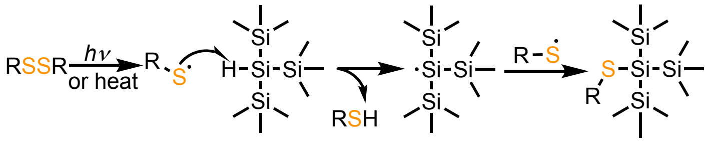

(b) concerted pathway

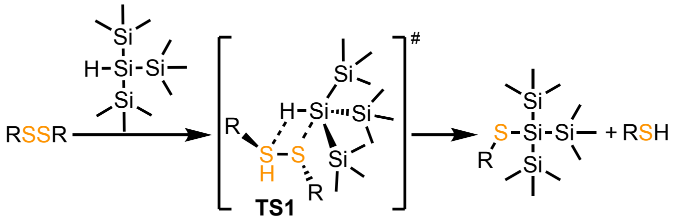

9. Characterization of Products

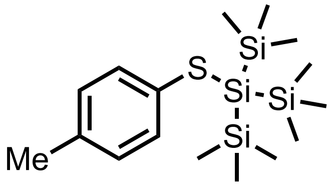

1,1,1,3,3,3-hexamethyl-2-(p-tolylthio)-2-(trimethylsilyl)trisilane (2a)

Colorless oil, 36.7 mg, 99% yield.

^{1}H NMR (400 MHz, $CDCl_3$) δ 7.34 (d, *J* = 8.1 Hz, 2H), 7.00 (d, *J* = 7.9 Hz, 2H), 2.29 (s, 3H), 0.18 (s, 27H).

^{13}C NMR (100 MHz, $CDCl_3$) δ 136.2, 134.4, 131.3, 129.5, 21.1, 0.8.

^{29}Si NMR (79 MHz, $CDCl_3$) δ -11.14, -49.94.

HRMS (EI): m/z calcd. for $C_{16}H_{34}SSi_4^+$ $[M]^+$ 370.1458, found 370.1452.

IR (neat, cm^{-1}): 2949, 2893, 1490, 1243, 1088, 806, 826, 687, 622.

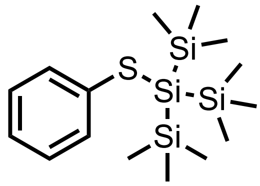

1,1,1,3,3,3-hexamethyl-2-(phenylthio)-2-(trimethylsilyl)trisilane (2b)

Colorless oil, 35 mg, 98% yield.

^{1}H NMR (400 MHz, $CDCl_3$) δ 7.49 – 7.44 (m, 2H), 7.21 – 7.16 (m, 3H), 0.19 (s, 27H).

^{13}C NMR (100 MHz, $CDCl_3$) δ 135.3, 134.4, 128.7, 126.4, 0.8.

^{29}Si NMR (79 MHz, $CDCl_3$) δ -11.05, -49.90.

HRMS (EI): m/z calcd. for $C_{15}H_{32}SSi_4^+$ $[M]^+$ 356.1302, found 356.1302.

IR (neat, cm^{-1}): 2949, 2893, 1581, 1474, 1438, 1244, 1082, 828, 741, 689, 623.

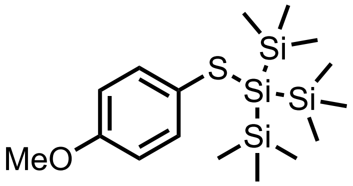

2-((4-methoxyphenyl)thio)-1,1,1,3,3,3-hexamethyl-2-(trimethylsilyl)trisilane (2c)

Colorless oil, 31.8 mg, 82% yield.

^{1}**H NMR** (400 MHz, $CDCl_3$) δ 7.36 (d, *J* = 8.7 Hz, 2H), 6.74 (d, *J* = 8.7 Hz, 2H), 3.77 (s, 3H), 0.17 (s, 27H).

^{13}**C NMR** (100 MHz, $CDCl_3$) δ 158.8, 135.9, 125.2, 114.4, 55.5, 0.8.

^{29}**Si NMR** (79 MHz, $CDCl_3$) δ -11.28, -49.31.

HRMS (EI): m/z calcd. for $C_{16}H_{34}OSSi_4^+$ $[M]^+$ 386.1407, found 386.1398.

IR (neat, cm^{-1}): 2950, 1591, 1490, 1241, 1170, 1034, 822, 688, 622.

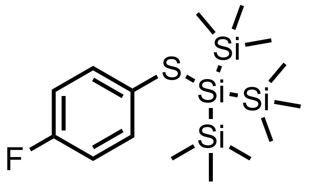

2-((4-fluorophenyl)thio)-1,1,1,3,3,3-hexamethyl-2-(trimethylsilyl)trisilane (2d)

Colorless oil, 35.2 mg, 94% yield.

^{1}**H NMR** (400 MHz, $CDCl_3$) δ 7.48 – 7.36 (m, 2H), 6.95 – 6.84 (m, 2H), 0.18 (s, 27H).

^{13}**C NMR** (100 MHz, $CDCl_3$) δ 162.0 (d, *J* = 246.1 Hz), 136.1 (d, *J* = 7.9 Hz), 130.2 (d, *J* = 3.4 Hz), 115.7 (d, *J* = 21.6 Hz), 0.8.

^{19}**F NMR** (376 MHz, $CDCl_3$) δ -116.02.

HRMS (EI): m/z calcd. for $C_{15}H_{31}FSSi_4^+$ $[M]^+$ 374.1208, found 374.1189.

IR (neat, cm^{-1}): 2950, 2894, 1589, 1487, 1245, 1229, 827, 689, 624.

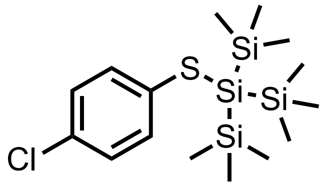

2-((4-chlorophenyl)thio)-1,1,1,3,3,3-hexamethyl-2-(trimethylsilyl)trisilane (2e)

Colorless oil, 32.8 mg, 84% yield.

^{1}**H NMR** (400 MHz, $CDCl_3$) δ 7.37 (d, *J* = 8.5 Hz, 2H), 7.16 (d, *J* = 8.5 Hz, 2H), 0.19 (s, 27H).

^{13}**C NMR** (100 MHz, $CDCl_3$) δ 135.4, 134.2, 132.4, 128.8, 0.9.

^{29}**Si NMR** (79 MHz, $CDCl_3$) δ -10.92, -49.22.

HRMS (EI): m/z calcd. for : $C_{15}H_{31}ClSSi_4^+$ $[M]^+$ 390.0912, found 390.0906.

IR (neat, cm^{-1}): 2950, 1474, 1245, 1092, 1013, 834, 689, 623.

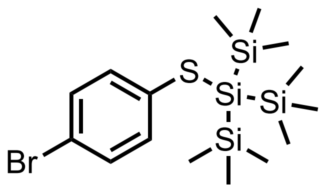

2-((4-bromophenyl)thio)-1,1,1,3,3,3-hexamethyl-2-(trimethylsilyl)trisilane (2f)

Colorless oil, 42.2 mg, 97% yield.

^{1}H NMR (400 MHz, $CDCl_3$) δ 7.31 (s, 4H), 0.19 (s, 27H).

^{13}C NMR (100 MHz, $CDCl_3$) δ 135.64, 134.95, 131.66, 120.23, 0.87.

^{29}Si NMR (79 MHz, $CDCl_3$) δ -10.89, -49.42.

HRMS (EI): m/z calcd. for $C_{15}H_{31}BrSSi_4^+$ $[M]^+$ 434.0407, found 434.0403.

IR (neat, cm^{-1}): 2950, 1471, 1245, 1086, 1010, 834, 690, 623.

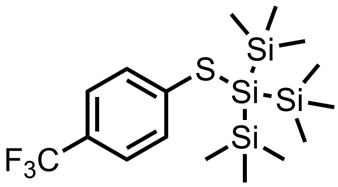

1,1,1,3,3,3-hexamethyl-2-((4-(trifluoromethyl)phenyl)thio)-2-(trimethylsilyl)trisilane (2g)

Colorless oil, 37.5 mg, 88% yield.

^{1}H NMR (400 MHz, $CDCl_3$) δ 7.53 (d, J = 8.1 Hz, 2H), 7.43 (d, J = 8.2 Hz, 2H), 0.20 (s, 27H).

^{13}C NMR (150 MHz, $CDCl_3$) δ 141.7, 133.5, 128.18 (q, J = 32.6 Hz), 125.35 (q, J = 3.8 Hz), 124.4 (q, J = 271.9 Hz), 0.9.

^{19}F NMR (376 MHz, $CDCl_3$) δ -62.38.

^{29}Si NMR (79 MHz, $CDCl_3$) δ -10.65, -50.29.

HRMS (EI): m/z calcd. for $C_{16}H_{31}F_3SSi_4^+$ $[M]^+$ 424.1176, found 424.1179.

IR (neat, cm^{-1}): 2953, 1580, 1324, 1246, 1128, 1091, 832, 690, 623.

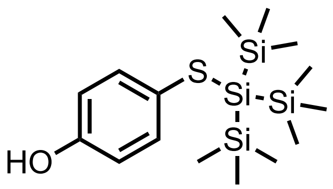

4-((1,1,1,3,3,3-hexamethyl-2-(trimethylsilyl)trisilan-2-yl)thio)phenol (2h)

Colorless oil, 36.2 mg, 96% yield.

^{1}H NMR (400 MHz, $CDCl_3$) δ 7.32 (d, J = 8.6 Hz, 2H), 6.68 (d, J = 8.6 Hz, 2H), 4.97 (s, 1H), 0.17 (s, 27H).

^{13}C NMR (100 MHz, $CDCl_3$) δ 154.7, 136.2, 125.4, 115.9, 0.8.

^{29}Si NMR (79 MHz, $CDCl_3$) δ -11.25, -48.87.

HRMS (EI): m/z calcd. for $C_{15}H_{32}OSSi_4^+$ $[M]^+$ 372.1251, found 372.1244.

IR (neat, cm^{-1}): 3398, 2950, 1582, 1491, 1426, 1242, 822, 688, 621.

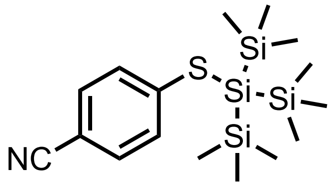

4-((1,1,1,3,3,3-hexamethyl-2-(trimethylsilyl)trisilan-2-yl)thio)benzonitrile (2i)

Colorless oil, 37.8 mg, 99% yield.

^{1}H NMR (400 MHz, $CDCl_3$) δ 7.50 (d, J = 8.1 Hz, 2H), 7.45 (d, J = 8.7 Hz, 2H), 0.21 (s, 27H).

^{13}C NMR (100 MHz, $CDCl_3$) δ 144.5, 133.4, 131.9, 79.0, 109.0, 0.9.

^{29}Si NMR (79 MHz, $CDCl_3$) δ -10.42, -50.40.

HRMS (EI): m/z calcd. for $C_{16}H_{31}NSSi_4^+$ $[M]^+$ 381.1254, found 381.1243.

IR (neat, cm^{-1}): 2950, 2894, 2227, 1590, 1482, 1397, 1246, 1084, 830, 690, 623.

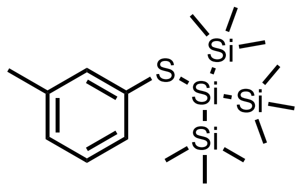

1,1,1,3,3,3-hexamethyl-2-(*m*-tolylthio)-2-(trimethylsilyl)trisilane (2j)

Colorless oil, 36.7 mg, 99% yield.

^{1}H NMR (600 MHz, $CDCl_3$) δ 7.28 (s, 1H), 7.26 – 7.23 (m, 1H), 7.07 (t, J = 7.6 Hz, 1H), 7.00 – 6.94 (m, 1H), 2.28 (s, 3H), 0.19 (s, 27H).

^{13}C NMR (150 MHz, $CDCl_3$) δ 138.3, 135.0, 134.9, 131.2, 128.5, 127.1, 21.18, 0.9.

^{29}Si NMR (119 MHz, $CDCl_3$) δ -11.04, -50.43.

HRMS (EI): m/z calcd. for $C_{16}H_{34}SSi_4^+$ $[M+H]^+$ 370.1458, found 370.1451.

IR (neat, cm^{-1}): 2949, 1590, 1473, 1244, 833, 775, 690, 623.

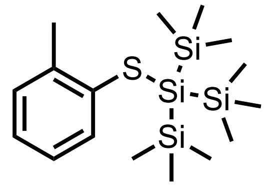

1,1,1,3,3,3-hexamethyl-2-(*o*-tolylthio)-2-(trimethylsilyl)trisilane (2k)

Colorless oil, 35.6 mg, 96% yield.

^{1}H NMR (600 MHz, $CDCl_3$) δ 7.44 (dd, J = 7.5, 1.6 Hz, 1H), 7.12 (dd, J = 7.4, 1.8 Hz, 1H), 7.05 (dtd, J = 18.5, 7.3, 1.6 Hz, 2H), 2.46 (s, 3H), 0.19 (s, 27H).

^{13}C NMR (150 MHz, $CDCl_3$) δ 140.6, 135.3, 135.0, 130.1, 126.3, 126.1, 22.0, 0.9.

^{29}Si NMR (119 MHz, $CDCl_3$) δ -10.98, -50.81.

HRMS (EI): m/z calcd. for $C_{16}H_{34}SSi_4^+$ $[M+H]^+$ 370.1458, found 370.1450.

IR (neat, cm^{-1}): 2949, 2894, 1582, 1466, 1244, 1058, 831, 744, 689, 623.

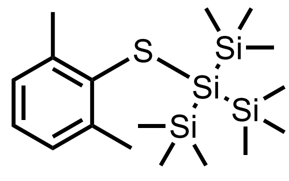

2-((2,6-dimethylphenyl)thio)-1,1,1,3,3,3-hexamethyl-2-(trimethylsilyl)trisilane (2l)

Colorless oil, 33.8 mg, 88% yield.

^{1}H NMR (400 MHz, $CDCl_3$) δ 7.04 (s, 3H), 2.52 (s, 6H), 0.16 (s, 27H).

^{13}C NMR (100 MHz, $CDCl_3$) δ 143.1, 133.8, 127.9, 126.8, 24.0, 1.0.

^{29}Si NMR (79 MHz, $CDCl_3$) δ -10.65, -50.43.

HRMS (EI): m/z calcd. for $C_{17}H_{36}SSi_4^+$ $[M]^+$ 384.1615, found 384.1608.

IR (neat, cm^{-1}): 2949, 2894, 1460, 1243,1043, 825, 765, 687, 622.

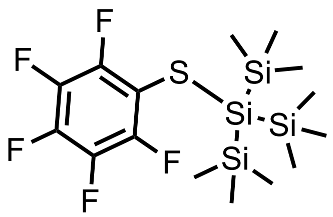

1,1,1,3,3,3-hexamethyl-2-((perfluorophenyl)thio)-2-(trimethylsilyl)trisilane (2m)

White solid, 41.5 mg, 93% yield.

^{1}H NMR (400 MHz, $CDCl_3$) δ 0.19 (s, 27H).

^{13}C NMR (100 MHz, $CDCl_3$) δ 149.7 – 149.5 (m), 147.3 – 147.1 (m), 142.13 – 141.07 (m), 139.7 – 138.7 (m), 137.00 – 136.14 (m), 110.1 – 109.7 (m), 0.42.

^{19}F NMR (376 MHz, $CDCl_3$) δ -132.11 (dd, J = 25.2, 7.8 Hz, 2F), -157.90 (t, J = 21.0 Hz, 1F), -164.67 (ddd, J = 25.1, 20.7, 7.8 Hz, 2F).

^{29}Si NMR (79 MHz, $CDCl_3$) δ -10.05, -42.18.

HRMS (EI): m/z calcd. for $C_{15}H_{27}F_5SSi_4^+$ $[M]^+$ 446.0831, found 446.0827.

IR (neat, cm^{-1}): 2952, 2896, 1636, 1509, 1482, 1246, 1086, 978, 824, 746, 689, 621.

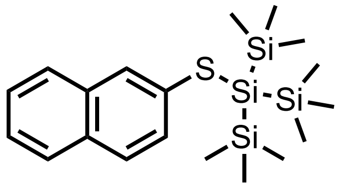

1,1,1,3,3,3-hexamethyl-2-(naphthalen-2-ylthio)-2-(trimethylsilyl)trisilane (2n)

Colorless oil, 31.6 mg, 78% yield.

^{1}H NMR (400 MHz, $CDCl_3$) δ 7.92 (s, 1H), 7.77 (d, J = 7.8 Hz, 1H), 7.68 (t, J = 7.6 Hz, 2H), 7.51 (dd, J = 8.6, 1.9 Hz, 1H), 7.48 – 7.39 (m, 2H), 0.21 (s, 27H).

^{13}C NMR (100 MHz, $CDCl_3$) δ 133.8, 133.2, 131.9, 131.8, 131.7, 128.0, 127.8, 126.8, 126.5, 125.6, 0.9.

^{29}Si NMR (79 MHz, $CDCl_3$) δ -10.94, -50.87.

HRMS (EI): m/z calcd. for $C_{19}H_{34}SSi_4^+$ $[M]^+$ 406.1458, found 406.1457.

IR (neat, cm^{-1}): 2949, 1590, 1245, 834, 742, 689, 623.

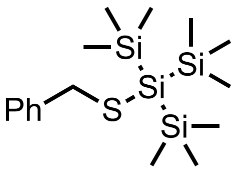

2-(benzylthio)-1,1,1,3,3,3-hexamethyl-2-(trimethylsilyl)trisilane (2o)

Colorless oil, 30.3 mg, 82% yield.

^{1}H NMR (400 MHz, $CDCl_3$) δ 7.35 – 7.27 (m, 4H), 7.24 – 7.18 (m, 1H), 3.72 (s, 2H), 0.26 (s, 27H).

^{13}C NMR (100 MHz, $CDCl_3$) δ 140.9, 128.7, 128.6, 127.1, 35.0, 1.0.

^{29}Si NMR (79 MHz, $CDCl_3$) δ -11.23, -54.93.

HRMS (EI): m/z calcd. for $C_{16}H_{34}SSi_4^+$ $[M]^+$ 370.1458, found 370.1449.

IR (neat, cm^{-1}): 3287, 2953, 1643, 1505, 1366, 1244, 1163, 831, 688, 623.

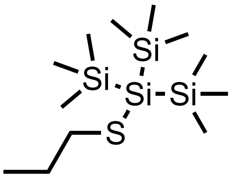

1,1,1,3,3,3-hexamethyl-2-(propyldisulfaneyl)-2-(trimethylsilyl)trisilane (2p)

Colorless oil, 29 mg, 90% yield.

^{1}H NMR (400 MHz, $CDCl_3$) δ 2.49 (t, J = 7.3 Hz, 2H), 1.69 – 1.52 (m, 2H), 0.98 (t, J = 7.3 Hz, 3H), 0.22 (s, 27H).

^{13}C NMR (100 MHz, $CDCl_3$) δ 33.0, 26.7, 13.7, 0.9.

^{29}Si NMR (79 MHz, $CDCl_3$) δ -11.53, -55.93.

HRMS (EI): m/z calcd. for $C_{12}H_{34}SSi_4^+$ $[M]^+$ 322.1458, found 322.1452.

IR (neat, cm^{-1}): 2949, 1243, 1051, 827, 744, 687, 622.

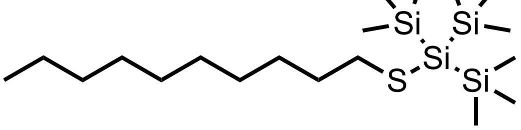

2-(decylthio)-1,1,1,3,3,3-hexamethyl-2-(trimethylsilyl)trisilane (2q)

Colorless oil, 41.8 mg, 99% yield.

¹H NMR (600 MHz, $CDCl_3$) δ 2.50 (t, *J* = 7.5 Hz, 2H), 1.59 (p, *J* = 7.5 Hz, 2H), 1.40 – 1.34 (m, 2H), 1.30 – 1.21 (m, 12H), 0.88 (t, *J* = 6.9 Hz, 3H), 0.23 (s, 27H).

¹³C NMR (100 MHz, $CDCl_3$) δ 33.5, 32.1, 31.0, 29.7, 29.7, 29.5, 29.4, 29.1, 22.8, 14.3, 0.9.

²⁹Si NMR (79 MHz, $CDCl_3$) δ -11.53, -56.05.

HRMS (EI): m/z calcd. for $C_{19}H_{48}SSi_4^+$ $[M]^+$ 420.2554, found 420.2549.

IR (neat, cm^{-1}): 2924, 2854, 1244, 1049, 829, 688, 623.

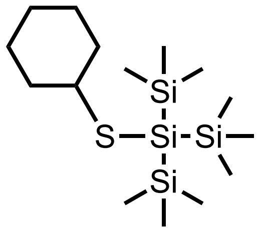

2-(cyclohexylthio)-1,1,1,3,3,3-hexamethyl-2-(trimethylsilyl)trisilane (2r)

Colorless oil, 32 mg, 88%

¹H NMR (400 MHz, $CDCl_3$) δ 2.68 – 2.54 (m, 1H), 2.11 – 1.90 (m, 2H), 1.81 – 1.70 (m, 2H), 1.43 – 1.32 (m, 2H), 1.31 – 1.12 (m, 4H), 0.22 (s, 27H).

¹³C NMR (100 MHz, $CDCl_3$) δ 44.2, 38.3, 27.0, 25.5, 1.0.

²⁹Si NMR (79 MHz, $CDCl_3$) δ -11.73, -57.46.

HRMS (EI): m/z calcd. for $C_{15}H_{38}SSi_4^+$ $[M]^+$ 362.1771, found 362.1765.

IR (neat, cm^{-1}): 2929, 2852, 1448, 1243, 995, 825, 745, 686, 622.

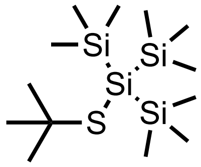

2-(*tert*-butylthio)-1,1,1,3,3,3-hexamethyl-2-(trimethylsilyl)trisilane (2s)

Colorless oil, 33 mg, 98%

¹H NMR (400 MHz, $CDCl_3$) δ 1.41 (s, 9H), 0.25 (s, 27H).

¹³C NMR (100 MHz, $CDCl_3$) δ 43.0, 35.4, 1.4.

²⁹Si NMR (79 MHz, $CDCl_3$) δ -11.51, -64.85.

HRMS (EI): m/z calcd. for $C_{13}H_{36}SSi_4^+$ $[M]^+$ 336.1615, found 336.1607.

IR (neat, cm^{-1}): 2952, 2894, 1362, 1244, 1165, 1044, 831, 745, 688, 623.

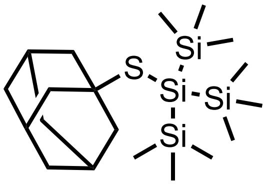

2-(adamantan-1-ylthio)-1,1,1,3,3,3-hexamethyl-2-(trimethylsilyl)trisilane (2t)

White solid, 38.6 mg, 93% yield.

^{1}H NMR (600 MHz, $CDCl_3$) δ 2.06 – 1.99 (m, 3H), 1.90 (d, J = 2.9 Hz, 6H), 1.65 (t, J = 3.1 Hz, 6H), 0.24 (s, 27H).

^{13}C NMR (100 MHz, $CDCl_3$) δ 47.6, 45.2, 36.1, 30.7, 1.6.

^{29}Si NMR (79 MHz, $CDCl_3$) δ -11.23, -67.54.

HRMS (EI): m/z calcd. for $C_{19}H_{42}SSi_4^+$ $[M]^+$ 414.2084, found 414.2079.

IR (neat, cm^{-1}): 2903, 2850, 1450, 1296, 1242, 1037, 824, 745, 684, 622.

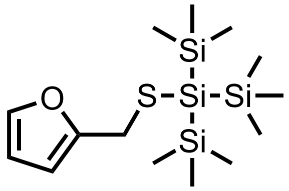

2-((furan-2-ylmethyl)thio)-1,1,1,3,3,3-hexamethyl-2-(trimethylsilyl)trisilane (2u)

Colorless oil, 35.6 mg, 99% yield.

^{1}H NMR (400 MHz, $CDCl_3$) δ 7.33 (d, J = 1.8 Hz, 1H), 6.28 (d, J = 2.5 Hz, 1H), 6.17 (s, 1H), 3.73 (s, 2H), 0.25 (s, 27H).

^{13}C NMR (100 MHz, $CDCl_3$) δ 153.7, 142.0, 110.6, 106.8, 26.7, 0.9.

^{29}Si NMR (79 MHz, $CDCl_3$) δ -11.14, -54.10.

HRMS (EI): m/z calcd. for $C_{14}H_{32}OSSi_4^+$ $[M]^+$ 360.1251, found 360.1249.

IR (neat, cm^{-1}): 3351, 2949, 1397, 1244, 1010, 829, 732, 687, 622.

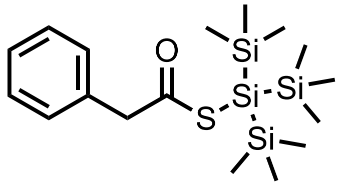

S-(1,1,1,3,3,3-hexamethyl-2-(trimethylsilyl)trisilan-2-yl) 2-phenylethanethioate (2v)

Colorless oil, 30.2 mg, 76% yield.

^{1}H NMR (400 MHz, $CDCl_3$) δ 7.35 – 7.23 (m, 5H), 3.93 (s, 2H), 0.19 (s, 27H).

^{13}C NMR (100 MHz, $CDCl_3$) δ 199.2, 134.8, 129., 15, 28.6, 127.2, 53.5, 0.8.

^{29}Si NMR (79 MHz, $CDCl_3$) δ -10.11, -60.23.

HRMS (EI): The fragmentation peak corresponding to $C_{14}H_{25}OSSi_3^+$ (325.0926) was detected due to elimination of trimethylsilyl (m/z calcd. for $C_{14}H_{25}OSSi_3^+$ 325.0934).

IR (neat, cm^{-1}): 2949, 2894, 1674, 1243, 827, 689, 622.

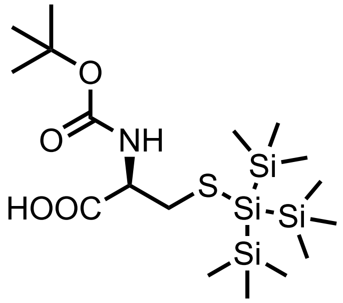

N-(*tert*-butoxycarbonyl)-S-(1,1,1,3,3,3-hexamethyl-2-(trimethylsilyl)trisilan-2-yl)-L-cysteine (2w)

White solid, 40.6 mg, 86% yield.

^{1}H NMR (600 MHz, $CDCl_3$) δ 5.41 (d, J = 8.1 Hz, 1H), 4.56 (s, 1H), 3.08 (d, J = 11.5 Hz, 1H), 2.92 (dd, J = 12.1, 4.6 Hz, 1H), 1.46 (s, 9H), 0.23 (s, 27H).

^{13}C NMR (100 MHz, $CDCl_3$) δ 175.9, 155.5, 80.4, 54.3, 32.4, 28.4, 0.9.

^{29}Si NMR (79 MHz, $CDCl_3$) δ -11.16, -53.47.

HRMS (ESI): m/z calcd. for $C_{17}H_{40}NO_4SSi_4^-$ [M-H]$^-$ 466.1760, found 466.1763.

IR (neat, cm^{-1}): 2949, 1716, 1500, 1367, 1245, 1163, 831, 689, 623.

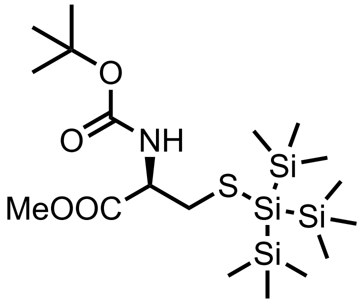

methyl N-(*tert*-butoxycarbonyl)-S-(1,1,1,3,3,3-hexamethyl-2-(trimethylsilyl)trisilan-2-yl)-L-cysteinate (2x)

White solid, 43.8 mg, 91% yield (0.1 mmol scale).

^{1}H NMR (400 MHz, $CDCl_3$) δ 5.42 (d, J = 8.7 Hz, 1H), 4.57 (dt, J = 8.8, 4.5 Hz, 1H), 3.75 (s, 3H), 3.03 (dd, J = 11.9, 4.4 Hz, 1H), 2.88 (dd, J = 11.8, 4.4 Hz, 1H), 1.44 (s, 9H), 0.22 (s, 27H).

^{13}C NMR (100 MHz, $CDCl_3$) δ 171.2, 155.3, 80.1, 54.4, 52.6, 32.8, 28.4, 0.9.

^{29}Si NMR (79 MHz, $CDCl_3$) δ -11.19, -54.00.

HRMS (EI): The fragmentation peak corresponding to $C_{14}H_{34}NO_3SSi_4^+$ (408.1331) was detected due to elimination of *tert*-butoxy (m/z calcd. for $C_{14}H_{34}NO_3SSi_4^+$ 408.1336).

IR (neat, cm^{-1}): 2951, 1717, 1498, 1366, 1244, 1162, 1053, 828, 688, 621.

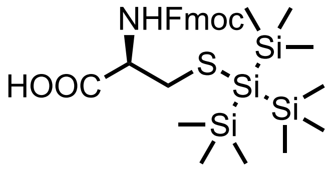

N-(((9H-fluoren-9-yl)methoxy)carbonyl)-S-(1,1,1,3,3,3-hexamethyl-2-(trimethylsilyl)trisilan-2-yl)-L-cysteine (2y)

White solid, 55.4 mg, 94% yield.

^{1}H NMR (400 MHz, $CDCl_3$) δ 7.77 (d, J = 7.5 Hz, 2H), 7.63 (dd, J = 7.8, 3.0 Hz, 2H), 7.40 (t, J = 7.5 Hz, 2H), 7.32 (t, J = 7.4 Hz, 2H), 5.74 (d, J = 8.3 Hz, 1H), 5.13 (s, 1H), 4.69 (dt, J = 8.8, 4.7 Hz, 1H), 4.40 (d, J = 7.3 Hz, 2H), 4.26 (t, J = 7.4 Hz, 1H), 3.14 (dd, J = 12.1, 4.7 Hz, 1H), 3.00 (dd, J = 12.2, 4.5 Hz, 1H), 0.24 (s, 27H).

^{13}C NMR (100 MHz, $CDCl_3$) δ 174.9, 156.1, 143.9, 141.4, 127.9, 127.2, 125.4, 120.1, 67.6, 54.8, 47.2, 32.2, 0.9.

^{29}Si NMR (79 MHz, $CDCl_3$) δ -11.07, -53.09.

HRMS (ESI): m/z calcd. for $C_{27}H_{42}NO_4SSi_4^-$ $[M-H]^-$ 588.1917, found 588.1921.

IR (neat, cm^{-1}): 2950, 1663, 1518, 1244, 1048, 832, 739, 688, 621.

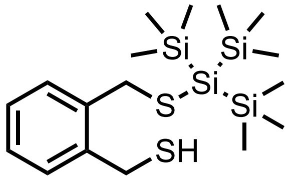

(2-(((1,1,1,3,3,3-hexamethyl-2-(trimethylsilyl)trisilan-2-yl)thio)methyl)phenyl)methanethiol (2z)

Colorless oil, 39.2 mg, 94%

^{1}H NMR (400 MHz, $CDCl_3$) δ 7.31 – 7.27 (m, 2H), 7.21 – 7.17 (m, 2H), 3.90 (d, J = 7.3 Hz, 2H), 3.83 (s, 2H), 1.91 (t, J = 7.3 Hz, 1H), 0.29 (s, 27H).

^{13}C NMR (100 MHz, $CDCl_3$) δ 139.4, 138.3, 130.4, 129.8, 128.0, 127.8, 32.1, 26.4, 1.0.

^{29}Si NMR (119 MHz, $CDCl_3$) δ -11.16, -55.32.

HRMS (ESI): m/z calcd. for $C_{17}H_{36}S_2Si_4Na^+$ $[M+Na]^+$ 439,1228, found 439.1234.

IR (neat, cm^{-1}): 2949, 1453, 1244, 1047, 827, 755, 688, 623.

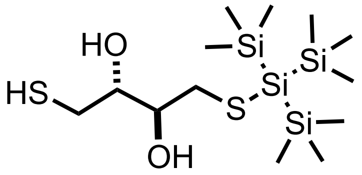

(±)-trans-1-((1,1,1,3,3,3-hexamethyl-2-(trimethylsilyl)trisilan-2-yl)thio)-4-mercaptobutane-2,3-diol (2z1)

White solid, 36.2 mg, 90% yield.

^{1}H NMR (400 MHz, $CDCl_3$) δ 3.73 – 3.59 (m, 2H), 2.81 – 2.61 (m, 6H), 1.53 (t, J = 8.6 Hz, 1H), 0.23 (s, 27H).

^{13}C NMR (100 MHz, $CDCl_3$) δ 73.6, 72.7, 34.7, 28.8, 0.9.

^{29}Si NMR (79 MHz, $CDCl_3$) δ -11.18, -53.34.

HRMS (EI): The fragmentation peak corresponding to $C_{10}H_{27}O_2S_2Si_3^+$ (327.0751) was detected due to elimination of trimethylsilyl (m/z calcd. for $C_{10}H_{27}O_2S_2Si_3^+$ 327.0760).

IR (neat, cm^{-1}): 3412, 2948, 2893, 1397, 1244, 1044, 826, 688, 622.

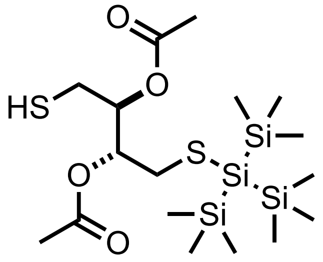

(±)-trans-1-((1,1,1,3,3,3-hexamethyl-2-(trimethylsilyl)trisilan-2-yl)thio)-4-mercaptobutane-2,3-diyl diacetate (2z2)

Colorless oil, 48.4 mg, 99%

^{1}H NMR (400 MHz, $CDCl_3$) δ 5.34 – 5.23 (m, 1H), 5.17 – 5.09 (m, 1H), 2.76 – 2.62 (m, 4H), 2.12 (s, 3H), 2.10 (s, 3H), 1.57 (t, J = 8.9 Hz, 1H), 0.22 (s, 27H).

^{13}C NMR (100 MHz, $CDCl_3$) δ 170.4, 170.2, 74.4, 72.7, 30.9, 24.6, 21.0, 21.0, 0.9.

^{29}Si NMR (79 MHz, $CDCl_3$) δ -11.27, -53.82.

HRMS (ESI): m/z calcd. for $C_{17}H_{40}O_4S_2Si_4Na^+$ $[M+Na]^+$ 507.1338, found 507.1350.

IR (neat, cm^{-1}): 2950, 1742, 1371, 1244, 1214, 1025, 828, 746, 688, 622.

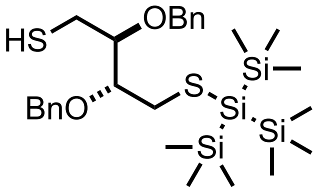

(±)-trans-2,3-bis(benzyloxy)-4-((1,1,1,3,3,3-hexamethyl-2-(trimethylsilyl)trisilan-2-yl)thio)butane-1-thiol (2z3)

Colorless oil, 46.8 mg, 81%

^{1}H NMR (400 MHz, $CDCl_3$) δ 7.40 – 7.27 (m, 10H), 4.77 (d, J = 11.8 Hz, 1H), 4.71 – 4.61 (m, 2H), 4.57 (d, J = 11.6 Hz, 1H), 3.81 (hept, J = 3.2 Hz, 1H), 3.72 (tt, J = 6.3, 3.2 Hz, 1H), 2.96 – 2.88 (m, 1H), 2.80 – 2.68 (m, 2H), 2.70 – 2.58 (m, 1H), 1.39 – 1.30 (m, 1H), 0.24 (s, 27H).

^{13}C NMR (100 MHz, $CDCl_3$) δ 138.4, 138.3, 128.5, 128.5, 128.4, 128.2, 127.9, 127.9, 81.3, 80.1, 73.5, 73.2, 30.4, 24.7, 1.0.

^{29}Si NMR (79 MHz, $CDCl_3$) δ -11.38, -55.00.

HRMS (ESI): m/z calcd. for $C_{27}H_{48}O_2S_2Si_4Na^+$ $[M+Na]^+$ 603.2065, found 603.2078.

IR (neat, cm^{-1}): 2949, 1723, 1453, 1245, 1027, 834, 734, 695, 623.

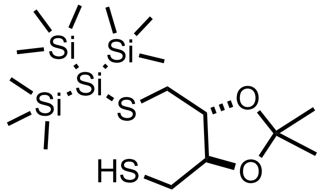

(±)-(trans-5-(((1,1,1,3,3,3-hexamethyl-2-(trimethylsilyl)trisilan-2-yl)thio)methyl)-2,2-dimethyl-1,3-dioxolan-4-yl)methanethiol (2z4)

Colorless oil, 41 mg, 93%

^{1}H NMR (400 MHz, $CDCl_3$) δ 3.96 – 3.88 (m, 2H), 2.96 – 2.86 (m, 1H), 2.86 – 2.75 (m, 2H), 2.73 – 2.64 (m, 1H), 1.64 (t, J = 8.3 Hz, 1H), 1.42 (s, 3H), 1.40 (s, 3H), 0.23 (s, 27H).

^{13}C NMR (100 MHz, $CDCl_3$) δ 109.4, 81.9, 80.3, 33.3, 28.0, 27.5, 0.9.

^{29}Si NMR (79 MHz, $CDCl_3$) δ -11.29, -53.48.

HRMS (EI): The fragmentation peak corresponding to $C_{15}H_{37}O_2S_2Si_4^+$ (425.1304) was detected due to elimination of methyl (m/z calcd. for $C_{15}H_{37}O_2S_2Si_4^+$ 425.1312).

IR (neat, cm^{-1}): 2949, 2893, 1378, 1244, 1055, 825, 745, 688, 622.

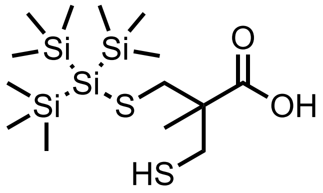

3-((1,1,1,3,3,3-hexamethyl-2-(trimethylsilyl)trisilan-2-yl)thio)-2-(mercaptomethyl)-2-methylpropanoic acid (2z5)

White solid, 30 mg, 73% yield.

^{1}H NMR (600 MHz, $CDCl_3$) δ 2.95 (d, J = 11.5 Hz, 1H), 2.85 (dd, J = 9.3, 2.8 Hz, 2H), 2.81 (d, J = 11.7 Hz, 1H), 1.55 (t, J = 9.3 Hz, 1H), 1.36 (s, 3H), 0.24 (s, 27H).

^{13}C NMR (150 MHz, $CDCl_3$) δ 181.1, 49.4, 36.4, 30.8, 21.7, 1.0.

^{29}Si NMR (119 MHz, $CDCl_3$) δ -11.24, -55.36.

HRMS (EI): The fragmentation peak corresponding to $C_{14}H_{35}O_2S_2Si_4^+$ (411.1151) was detected due to elimination of hydrogen (m/z calcd. for $C_{14}H_{35}O_2S_2Si_4^+$ 411.1156).

IR (neat, cm^{-1}): 2949, 2893, 1703, 1398, 1244, 825, 744, 687, 622.

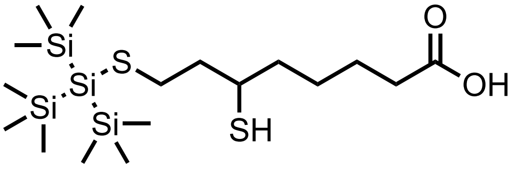

8-((1,1,1,3,3,3-hexamethyl-2-(trimethylsilyl)trisilan-2-yl)thio)-6-mercaptooctanoic acid (2z6)

Colorless oil, 40.2 mg, 89% yield.

^{1}H NMR (400 MHz, $CDCl_3$) δ 2.98 – 2.88 (m, 1H), 2.74 – 2.60 (m, 2H), 2.37 (t, J = 7.3 Hz, 2H), 1.97 – 1.84 (m, 1H), 1.77 – 1.62 (m, 4H), 1.55 – 1.40 (m, 3H), 1.31 (d, J = 7.5 Hz, 1H), 0.23 (s, 27H).

^{13}C NMR (100 MHz, $CDCl_3$) δ 179.9, 42.1, 39.9, 38.8, 34.0, 28.4, 26.6, 24.5, 0.9.

^{29}Si NMR (79 MHz, $CDCl_3$) δ -11.43, -55.55.

HRMS (EI): The fragmentation peak corresponding to $C_{17}H_{41}O_2S_2Si_4^+$ (453.1625) was detected due to elimination of hydrogen (m/z calcd. for $C_{17}H_{41}O_2S_2Si_4^+$ 453.1618).

IR (neat, cm^{-1}): 2947, 1708, 1243, 828, 688, 622.

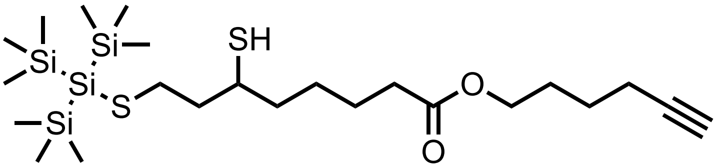

hex-5-yn-1-yl 8-((1,1,1,3,3,3-hexamethyl-2-(trimethylsilyl)trisilan-2-yl)thio)-6-mercaptooctanoate (2z7)

Colorless oil, 49.2 mg, 92%

^{1}H NMR (400 MHz, $CDCl_3$) δ 4.10 (t, J = 6.5 Hz, 2H), 3.01 – 2.84 (m, 1H), 2.67 (h, J = 11.5 Hz, 2H), 2.31 (t, J = 7.4 Hz, 2H), 2.24 (dt, J = 8.0, 4.0 Hz, 2H), 1.98 – 1.94 (m,

1H), 1.94 – 1.86 (m, 1H), 1.80 – 1.70 (m, 3H), 1.67 – 1.58 (m, 5H), 1.56 – 1.39 (m, 3H), 1.30 (d, J = 7.6 Hz, 1H), 0.23 (s, 27H).

^{13}C NMR (100 MHz, $CDCl_3$) δ 173.7, 84.0, 68.9, 63.9, 42.1, 39.9, 38.9, 34.3, 28.4, 27.8, 26.7, 25.1, 24.8, 18.2, 0.9.

^{29}Si NMR (79 MHz, $CDCl_3$) δ -11.44, -55.49.

HRMS (APCI): m/z calcd. for $C_{23}H_{51}O_2S_2Si_4^+$ $[M+H]^+$ 535.2402, found 535.2400.

IR (neat, cm^{-1}): 3291, 2928, 2850, 1730, 1455, 1242, 1174, 639.

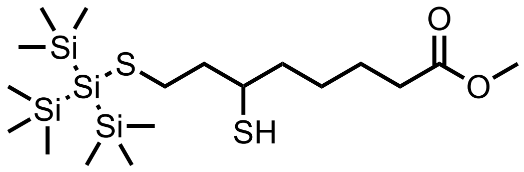

methyl 8-((1,1,1,3,3,3-hexamethyl-2-(trimethylsilyl)trisilan-2-yl)thio)-6-mercaptooctanoate (2z8)

Colorless oil, 44 mg, 93% yield.

^{1}H NMR (400 MHz, $CDCl_3$) δ 3.67 (s, 3H), 2.99 – 2.85 (m, 1H), 2.67 (pd, J = 11.7, 6.7 Hz, 2H), 2.32 (t, J = 7.4 Hz, 2H), 2.03 – 1.83 (m, 1H), 1.79 – 1.59 (m, 4H), 1.57 – 1.39 (m, 3H), 1.30 (d, J = 7.4 Hz, 1H), 0.23 (s, 27H).

^{13}C NMR (100 MHz, $CDCl_3$) δ 174.2, 51.7, 42.1, 39.9, 38.9, 34.1, 28.4, 26.7, 24.8, 0.9.

^{29}Si NMR (79 MHz, $CDCl_3$) δ -11.44, -55.54.

HRMS (EI): The fragmentation peak corresponding to $C_{15}H_{35}O_2S_2Si_3^+$ (395.1377) was detected due to elimination of trimethylsilyl (m/z calcd. for $C_{15}H_{35}O_2S_2Si_3^+$ 395.1386).

IR (neat, cm^{-1}): 2949, 1742, 1244, 1053, 830, 689, 623.

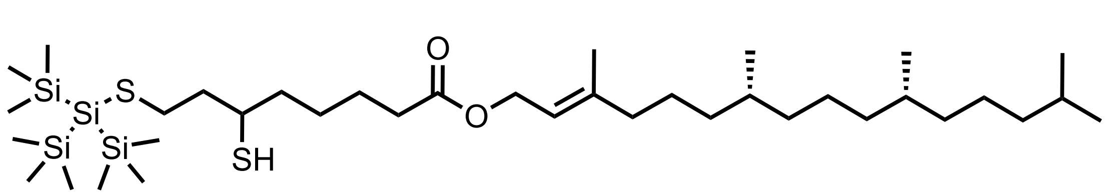

(7R,11R,E)-3,7,11,15-tetramethylhexadec-2-en-1-yl 8-((1,1,1,3,3,3-hexamethyl-2-(trimethylsilyl)trisilan-2-yl)thio)-6-mercaptooctanoate (2z9)

Colorless oil, 63 mg, 86%

¹H NMR (400 MHz, $CDCl_3$) δ 5.36 – 5.28 (m, 1H), 4.59 (d, *J* = 7.1 Hz, 2H), 2.98 – 2.85 (m, 1H), 2.75 – 2.60 (m, 2H), 2.31 (t, *J* = 7.4 Hz, 2H), 2.03 – 1.96 (m, 2H), 1.97 – 1.85 (m, 1H), 1.69 (s, 3H), 1.67– 1.62 (m, 2H), 1.58 – 1.47 (m, 4H), 1.48 – 1.32 (m, 8H), 1.28 (d, *J* = 2.6 Hz, 1H), 1.27 – 1.17 (m, 4H), 1.20 – 1.07 (m, 4H), 1.10 – 0.98 (m, 4H), 0.89 – 0.81 (m, 12H), 0.23 (s, 27H).

¹³C NMR (100 MHz, $CDCl_3$) δ 173.7, 142.8, 118.2, 61.4, 42.1, 40.0, 39.9, 39.5, 38.9, 37.6, 37.5, 37.4, 36.8, 34.4, 32.9, 32.8, 28.4, 28.1, 26.7, 25.2, 24.9, 24.8, 24.6, 22.9, 22.8, 19.9, 19.9, 16.5, 0.9.

²⁹Si NMR (79 MHz, $CDCl_3$) δ -11.45, -55.51.

HRMS (APCI): m/z calcd. for $C_{37}H_{81}O_2S_2Si_4^+$ $[M+H]^+$ 733.4750, found 733.4751.

IR (neat, cm^{-1}): 2927, 1736, 1508, 1244, 1160, 832, 689, 623.

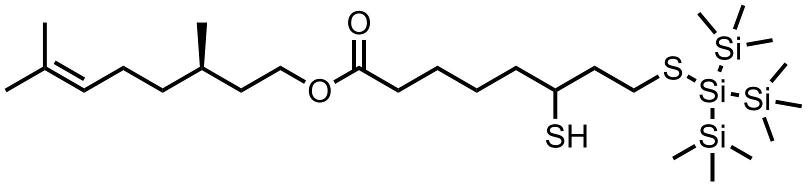

(R)-3,7-dimethyloct-6-en-1-yl 8-((1,1,1,3,3,3-hexamethyl-2-(trimethylsilyl)trisilan-2-yl)thio)-6-mercaptooctanoate (2z10)

Colorless oil, 53.4 mg, 90%

¹H NMR (400 MHz, $CDCl_3$) δ 5.11 – 5.06 (m, 1H), 4.14 – 4.06 (m, 2H), 2.97 – 2.85 (m, 1H), 2.74 – 2.60 (m, 2H), 2.30 (t, *J* = 7.4 Hz, 2H), 2.06 – 1.94 (m, 2H), 1.96 – 1.84 (m, 1H), 1.77 – 1.70 (m, 2H), 1.66 – 1.63 (m, 2H), 1.60 (s, 6H), 1.57 – 1.48 (m, 2H), 1.49 – 1.34 (m, 4H), 1.34 – 1.27 (m, 2H), 1.24 – 1.13 (m, 1H), 0.91 (d, *J* = 6.6 Hz, 3H), 0.23 (s, 27H).

¹³C NMR (100 MHz, $CDCl_3$) δ 173.8, 131.5, 124.7, 63.0, 42.1, 39.9, 38.9, 37.1, 35.6, 34.4, 29.7, 28.4, 26.7, 25.9, 25.5, 24.8, 19.6, 17.8, 0.9.

²⁹Si NMR (79 MHz, $CDCl_3$) δ -11.43, -55.50.

HRMS (APCI): m/z calcd. for $C_{27}H_{61}O_2S_2Si_4^+$ $[M+H]^+$ 593.3185, found 593.3173.

IR (neat, cm^{-1}): 2951, 1736, 1244, 1170, 1047, 830, 688, 623.

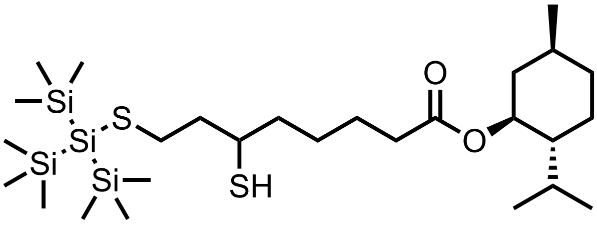

(1S,2R,5S)-2-isopropyl-5-methylcyclohexyl 8-((1,1,1,3,3,3-hexamethyl-2-(trimethylsilyl)trisilan-2-yl)thio)-6-mercaptooctanoate (2z11)

Colorless oil, 54.5 mg, 92%

^{1}H NMR (600 MHz, $CDCl_3$) δ 4.67 (td, J = 10.9, 4.4 Hz, 1H), 2.96 – 2.87 (m, 1H), 2.73 – 2.60 (m, 2H), 2.28 (t, J = 7.3 Hz, 2H), 2.00 – 1.94 (m, 1H), 1.92 – 1.84 (m, 2H), 1.75 – 1.63 (m, 4H), 1.57 – 1.40 (m, 4H), 1.40 – 1.30 (m, 2H), 1.29 (d, J = 7.4 Hz, 1H), 1.04 (qd, J = 13.4, 3.8 Hz, 1H), 0.95 (q, J = 11.5 Hz, 2H), 0.89 (dd, J = 6.8, 3.7 Hz, 6H), 0.75 (d, J = 7.0 Hz, 3H), 0.23 (s, 27H).

^{13}C NMR (100 MHz, $CDCl_3$) δ 173.3, 74.1, 47.2, 42.1, 41.1, 39.9, 38.9, 34.7, 34.4, 31.5, 28.4, 26.7, 26.4, 25.0, 23.6, 22.2, 20.9, 16.5, 0.9.

^{29}Si NMR (79 MHz, $CDCl_3$) δ -11.44, -55.52.

HRMS (APCI): m/z calcd. for $C_{27}H_{61}O_2S_2Si_4^+$ $[M+H]^+$ 593.3185, found 593.3180.

IR (neat, cm^{-1}): 2951, 11729, 1456, 1244, 1177, 1039, 829, 745, 688, 622.

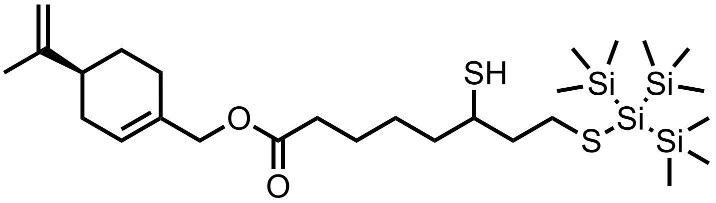

((S)-4-(prop-1-en-2-yl)cyclohex-1-en-1-yl)methyl 8-((1,1,1,3,3,3-hexamethyl-2-(trimethylsilyl)trisilan-2-yl)thio)-6-mercaptooctanoate (2z12)

Colorless oil, 55.8 mg, 95%

^{1}H NMR (400 MHz, $CDCl_3$) δ 5.77 – 5.73 (m, 1H), 4.87 – 4.65 (m, 2H), 4.46 (s, 2H), 2.97 – 2.87 (m, 1H), 2.74 – 2.62 (m, 2H), 2.34 (t, J = 7.4 Hz, 2H), 2.21 – 2.12 (m, 2H), 2.11 – 2.04 (m, 2H), 2.02 – 1.94 (m, 1H), 1.93 – 1.83 (m, 2H), 1.73 (s, 3H), 1.71 – 1.61 (m, 4H), 1.55 – 1.43 (m, 4H), 1.30 (d, J = 7.4 Hz, 1H), 0.23 (s, 27H).

^{13}C NMR (100 MHz, $CDCl_3$) δ 173.6, 149.8, 132.8, 125.9, 108.9, 68.5, 42.1, 41.0, 39.9, 38.9, 34.4, 30.6, 28.4, 27.5, 26.7, 26.6, 24.9, 20.9, 0.9.

^{29}Si NMR (119 MHz, $CDCl_3$) δ -11.44, -55.48.

HRMS (APCI): m/z calcd. for $C_{27}H_{57}O_2S_2Si_4^+$ $[M+H]^+$ 589.2872, found 589.2866.

IR (neat, cm^{-1}): 3287, 2953, 1643, 1505, 1366, 1244, 1163, 1060, 831, 688, 623.

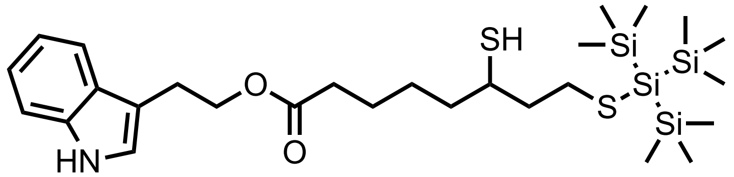

2-(1H-indol-3-yl)ethyl 8-((1,1,1,3,3,3-hexamethyl-2-(trimethylsilyl)trisilan-2-yl)thio)-6-mercaptooctanoate (2z13)

Colorless oil, 52 mg, 87%

^{1}H NMR (400 MHz, $CDCl_3$) δ 8.11 (s, 1H), 7.64 (d, J = 7.8 Hz, 1H), 7.37 (d, J = 8.1 Hz, 1H), 7.24 – 7.16 (m, 1H), 7.16 – 7.10 (m, 1H), 7.05 (d, J = 2.4 Hz, 1H), 4.37 (t, J = 7.1 Hz, 2H), 3.11 (t, J = 7.1 Hz, 2H), 2.94 – 2.84 (m, 1H), 2.75 – 2.61 (m, 2H), 2.31 (t, J = 7.4 Hz, 2H), 1.96 – 1.83 (m, 1H), 1.76 – 1.67 (m, 1H), 1.60 – 1.55 (m, 2H), 1.54 – 1.31 (m, 4H) 1.29 (d, J = 7.4 Hz, 1H), 0.25 (s, 27H).

^{13}C NMR (100 MHz, $CDCl_3$) δ 173.8, 136.3, 127.6, 122.2, 122.1, 112.3, 111.3, 64.6, 42.1, 39.9, 38.9, 34.4, 28.41 26.7, 24.9, 24.8, 0.9.

^{29}Si NMR (79 MHz, $CDCl_3$) δ -11.38, -55.39.

HRMS (APCI): m/z calcd. for $C_{27}H_{52}NO_2S_2Si_4^+$ $[M+H]^+$ 598.2511, found 598.2511.

IR (neat, cm^{-1}): 2951, 1729, 1456, 1369, 1244, 1177, 829, 745, 688, 622.

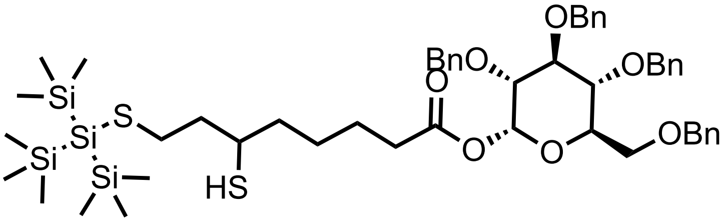

(2R,3R,4S,5R,6R)-3,4,5-tris(benzyloxy)-6-((benzyloxy)methyl)tetrahydro-2H-pyran-2-yl 8-((1,1,1,3,3,3-hexamethyl-2-(trimethylsilyl)trisilan-2-yl)thio)-6-

mercaptooctanoate (2z14)

Colorless oil, 74.2 mg, 76%

^{1}H NMR (400 MHz, $CDCl_3$) δ 7.36 – 7.24 (m, 18H), 7.18 – 7.14 (m, 2H), 5.65 (d, *J* = 8.1 Hz, 1H), 4.91 (d, *J* = 11.0 Hz, 1H), 4.86 – 4.81 (m, 2H), 4.8 – 4.74 (m, 2H), 4.64 (d, *J* = 12.1 Hz, 1H), 4.53 (dd, *J* = 21.8, 11.4 Hz, 2H), 3.79 – 3.70 (m, 4H), 3.63 – 3.58 (m, 2H), 2.88 (dd, *J* = 15.7, 7.7 Hz, 1H), 2.75 – 2.63 (m, 2H), 2.42 – 2.26 (m, 2H), 1.95 – 1.86 (m, 1H), 1.78 – 1.69 (m, 1H), 1.63 – 1.36 (m, 6H), 1.30 (d, *J* = 8.9 Hz, 1H), 0.26 (s, 27H).

^{13}C NMR (100 MHz, $CDCl_3$) δ 172.0, 138.5, 138.2, 138.1, 138.0, 128.54 128.5, 128.5, 128.1, 128.0, 127.9, 127.8, 94.1, 84.9, 81.2, 77.4, 75.8, 75.6, 75.1, 75.1, 73.6, 68.2, 42.1, 39.9, 38.9, 34.2, 28.3, 26.6, 24.4, 0.9.

^{29}Si NMR (79 MHz, $CDCl_3$) δ -11.41, -55.52.

HRMS (APCI): m/z calcd. for $C_{51}H_{77}O_7S_2Si_4^+$ [M+H]$^+$ 977.4182, found 977.4180.

IR (neat, cm^{-1}): 2947, 1756, 1453, 1360, 1244, 1073, 1028, 834, 734, 695, 623.

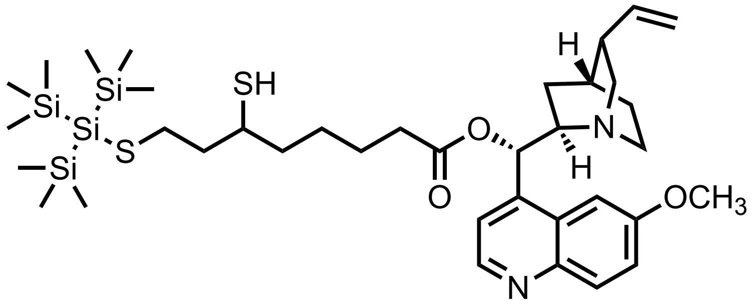

(S)-(6-methoxyquinolin-4-yl)((1S,2R,4R,5S)-5-vinylquinuclidin-2-yl)methyl 8-((1,1,1,3,3,3-hexamethyl-2-(trimethylsilyl)trisilan-2-yl)thio)-6-mercaptooctanoate (2z15)

Colorless oil, 55 mg, 72%

^{1}H NMR (400 MHz, $CDCl_3$) δ 8.73 (d, *J* = 5.1 Hz, 1H), 8.00 (d, *J* = 9.3 Hz, 1H), 7.44 (s, 1H), 7.35 (t, *J* = 7.8 Hz, 2H), 6.49 (d, *J* = 7.3 Hz, 1H), 5.83 (dt, *J* = 17.5, 8.8 Hz, 1H), 5.08 – 4.94 (m, 2H), 3.95 (s, 3H), 3.37 (q, *J* = 8.1 Hz, 1H), 3.16 – 2.97 (m, 2H), 2.91 – 2.82 (m, 1H), 2.68 – 2.58 (m, 4H), 2.38 (t, *J* = 7.6 Hz, 2H), 2.31 – 2.23 (m, 1H),

1.91 – 1.83 (m, 3H), 1.76 – 1.67 (m, 2H), 1.66 – 1.57 (m, 4H), 1.55 – 1.39 (m, 4H), 1.27 (d, *J* = 7.3 Hz, 1H), 0.22 (s, 27H).

^{13}C NMR (100 MHz, $CDCl_3$) δ 172.7, 158.0, 147.6, 144.9, 143.8, 141.8, 131.9, 127.2, 121.9, 119.0, 114.7, 101.6, 73.7, 59.2, 56.7, 55.8, 42.5, 42.1, 39.8, 38.8, 34.4, 28.3, 27.9, 27.7, 26.7, 24.7, 0.9.

^{29}Si NMR (79 MHz, $CDCl_3$) δ -11.44, -55.47.

HRMS (APCI): m/z calcd. for $C_{37}H_{65}N_2O_3S_2Si_4^+$ $[M+H]^+$ 761.3508, found 761.3493.

IR (neat, cm^{-1}): 2945, 1739, 1621, 1508, 1242, 1030, 829, 689, 623.

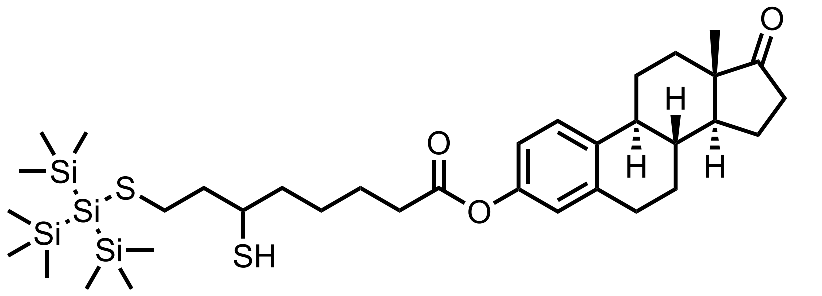

(8R,9S,13S,14S)-13-methyl-17-oxo-7,8,9,11,12,13,14,15,16,17-decahydro-6H-cyclopenta[a]phenanthren-3-yl 8-((1,1,1,3,3,3-hexamethyl-2-(trimethylsilyl)trisilan-2-yl)thio)-6-mercaptooctanoate (2z16)

Colorless oil, 58.6 mg, 83%

^{1}H NMR (400 MHz, $CDCl_3$) δ 7.30 (d, *J* = 8.5 Hz, 1H), 6.87 (dd, *J* = 8.4, 2.8 Hz, 1H), 6.84 – 6.81 (m, 1H), 2.98 – 2.90 (m, 3H), 2.76 – 2.66 (m, 2H), 2.57 (t, *J* = 7.3 Hz, 2H), 2.54 – 2.48 (m, 1H), 2.45 – 2.39 (m, 1H), 2.36 – 2.25 (m, 1H), 2.17 (q, *J* = 9.2 Hz, 1H), 2.12 – 2.02 (m, 2H), 2.02 – 1.89 (m, 3H), 1.80 – 1.71 (m, 4H), 1.69 – 1.59 (m, 4H), 1.59 – 1.55 (m, 2H), 1.51 – 1.42 (m, 2H), 1.34 (d, *J* = 7.5 Hz, 1H), 0.92 (s, 3H), 0.26 (s, 27H).

^{13}C NMR (100 MHz, $CDCl_3$) δ 220.8, 172.4, 148.7, 138.1, 137.4, 126.5, 121.7, 118.9, 50.5, 48.0, 44.2, 42.1, 40.0, 38.8, 38.1, 35.9, 34.3, 31.7, 29.5, 28.3, 26.6, 26.4, 25.9, 24.8, 21.7, 0.9.

^{29}Si NMR (79 MHz, $CDCl_3$) δ -11.43, -55.48.

HRMS (APCI): m/z calcd. for $C_{35}H_{63}O_3S_2Si_4^+$ $[M+H]^+$ 707.3290, found 707.3287.

IR (neat, cm^{-1}): 2945, 1738, 1508, 1243, 1151, 832, 734, 689, 622.

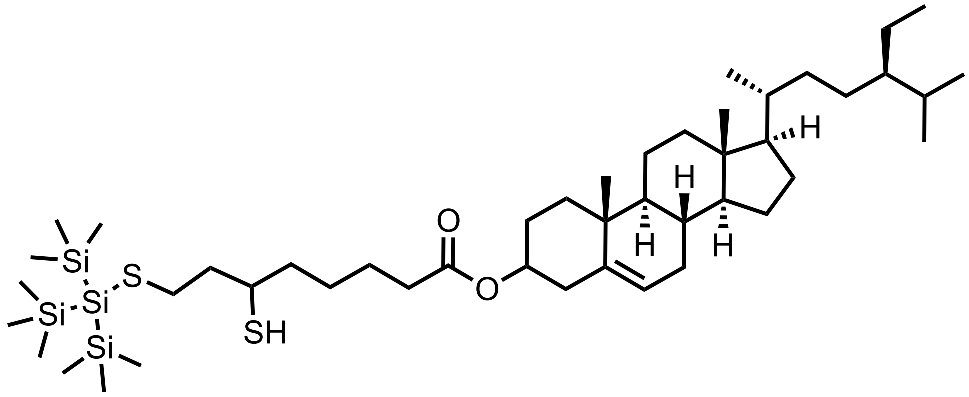

(8S,9S,10R,13R,14S,17R)-17-((2R,5R)-5-ethyl-6-methylheptan-2-yl)-10,13-dimethyl-2,3,4,7,8,9,10,11,12,13,14,15,16,17-tetradecahydro-1H-cyclopenta[a]phenanthren-3-yl 8-((1,1,1,3,3,3-hexamethyl-2-(trimethylsilyl)trisilan-2-yl)thio)-6-mercaptooctanoate (2z17)

Colorless oil, 78.5 mg, 92%

^{1}H NMR (600 MHz, $CDCl_3$) δ 5.37 (d, *J* = 5.1 Hz, 1H), 4.61 (tdd, *J* = 10.9, 6.9, 4.3 Hz, 1H), 2.92 (td, *J* = 8.8, 4.6 Hz, 1H), 2.75 – 2.60 (m, 2H), 2.34 – 2.23 (m, 4H), 2.03 – 1.93 (m, 2H), 1.94 – 1.87 (m, 1H), 1.89 – 1.81 (m, 3H), 1.77 – 1.69 (m, 1H), 1.71 – 1.57 (m, 6H), 1.56 – 1.40 (m, 7H), 1.38 – 1.32 (m, 2H), 1.32 – 1.19 (m, 6H), 1.21 – 1.13 (m, 4H), 1.12 – 1.05 (m, 2H), 1.02 (s, 3H), 1.02 – 0.93 (m, 2H), 0.92 (d, *J* = 6.5 Hz, 3H), 0.83 (ddd, *J* = 12.6, 8.7, 6.2 Hz, 9H), 0.68 (s, 3H), 0.24 (s, 27H).

^{13}C NMR (100 MHz, $CDCl_3$) δ 173.1, 139.8, 122.8, 74.0, 56.8, 56.2, 50.2, 46.0, 42.5, 42.1, 39.9, 39.9, 38.9, 38.3, 37.2, 36.8, 36.3, 34.7, 34.1, 32.0, 29.3, 28.4, 28.0, 26.7, 26.2, 24.9, 24.4, 23.2, 21.2, 20.0, 19.5, 19.2, 18.9, 12.1, 12.0, 0.9.

^{29}Si NMR (79 MHz, $CDCl_3$) δ -11.45, -55.50.

HRMS (APCI): m/z calcd. for $C_{46}H_{91}O_2S_2Si_4^+$ $[M+H]^+$ 851.5532, found 851.5498.

IR (neat, cm^{-1}): 2949, 2866, 1727, 1264, 1244, 1030, 834, 736, 690, 623.

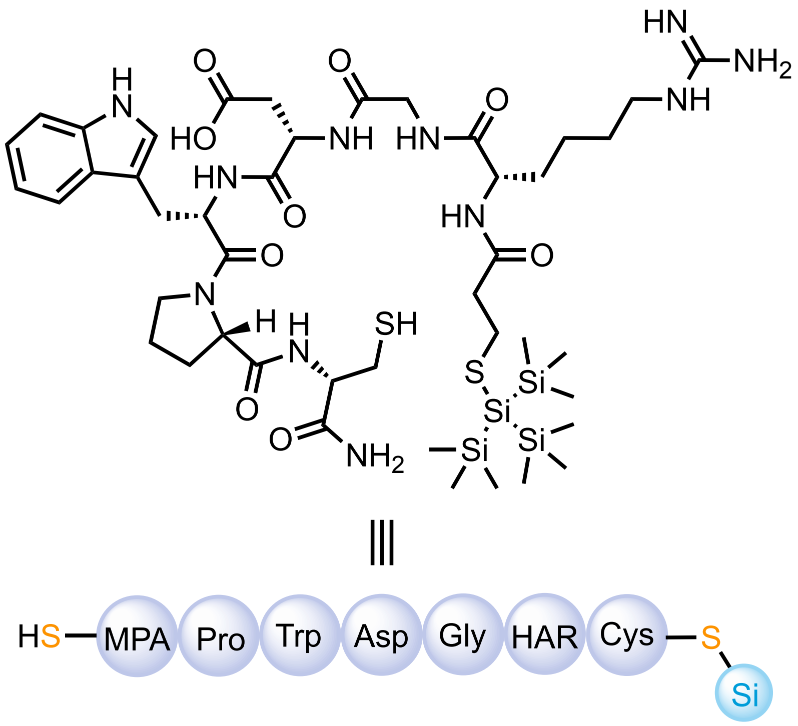

(9S,15S)-15-(((S)-1-((S)-2-(((S)-1-amino-3-mercapto-1-oxopropan-2-yl)carbamoyl)pyrrolidin-1-yl)-3-(1H-indol-3-yl)-1-oxopropan-2-yl)carbamoyl)-9-(4-guanidinobutyl)-2,2-dimethyl-7,10,13-trioxo-3,3-bis(trimethylsilyl)-4-thia-8,11,14-triaza-2,3-disilaheptadecan-17-oic acid (2z18)

The synthesis steps of **2z18**: A 4 mL glass vial equipped with a magnetic stir bar was charged with DMF/MTBE (0.3 mL/0.1 mL) and eptifibatide (2 mg, 0.0024 mmol). Tris(trimethylsilyl)silane (74 µL, 100 equiv.) was added without inert atmosphere protection. The reaction mixture was stirred at 450 nm irradiation at room temperature for 24 h. The reaction mixture was analyzed by LC-MS using a BaseLine® C18 column (5 µm, 250 × 4.6 mm) with methanol as the eluent. The retention times were as follows: starting material, 6.9 min; product **2z18**, 7.5 min; DMF, 9.4 min; TTMSS, 14.3 min. The yield of **2z18** was determined to be 50% based on the peak area percentage.

HRMS (ESI): m/z calcd. for $C_{44}H_{78}N_{11}O_9S_2Si_4^+$ $[M+H]^+$ 1080.4497, found 1080.4483.

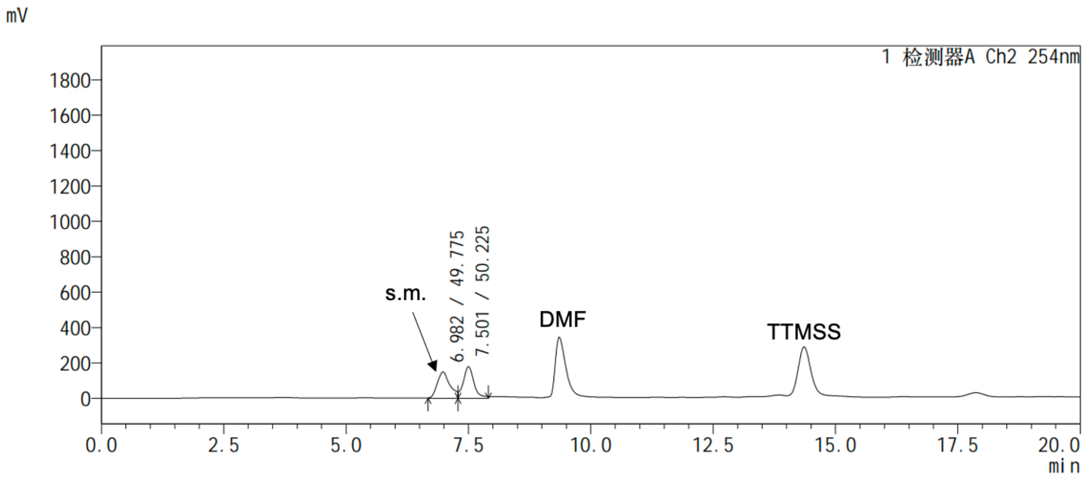

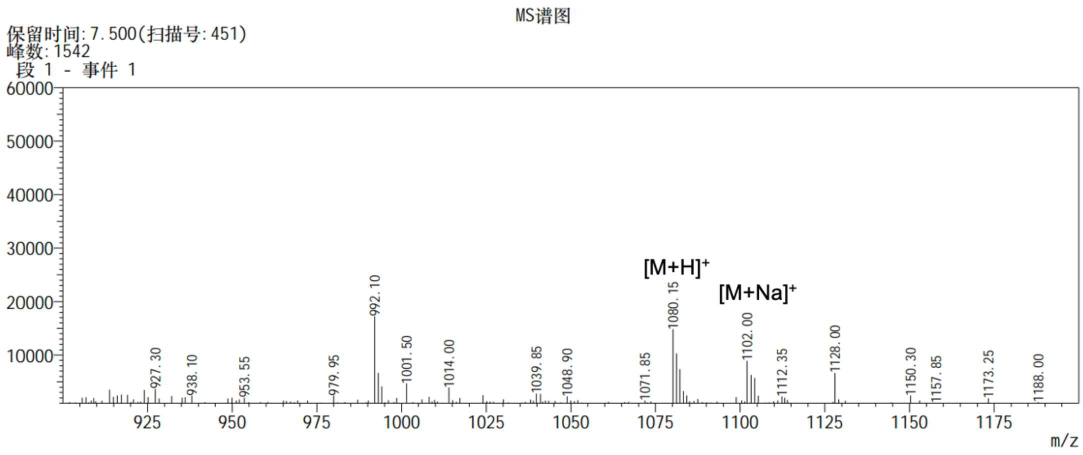

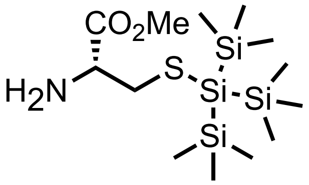

methyl S-(1,1,1,3,3,3-hexamethyl-2-(trimethylsilyl)trisilan-2-yl)-L-cysteinate (2z19)

Colorless oil, 22 mg, 60% yield.

^{1}H NMR (400 MHz, $CDCl_3$) δ 3.73 (s, 3H), 3.63 (dd, *J* = 7.3, 4.2 Hz, 1H), 2.91 (dd, *J* = 11.8, 4.3 Hz, 1H), 2.78 (dd, *J* = 11.8, 7.3 Hz, 1H), 0.22 (s, 27H).

^{13}C NMR (100 MHz, $CDCl_3$) δ 174.3, 56.4, 52.4, 35.5, 0.9.

^{29}Si NMR (79 MHz, $CDCl_3$) δ -11.23, -54.24.

HRMS (ESI): m/z calcd. for $C_{12}H_{36}NO_2SSi_4^+$ $[M]^+$ 382.1539, found 382.1545.

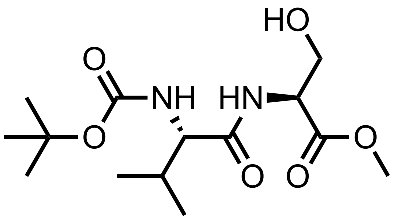

methyl (*tert*-butoxycarbonyl)-L-valyl-L-serinate (2z20)

White solid, 120 mg, 75%

^{1}H NMR (600 MHz, $CDCl_3$) δ 7.07 (m, 1H), 5.40 – 5.22 (m, 1H), 4.67 (m, 1H), 4.06 – 3.84 (m, 3H), 3.77 (s, 3H), 2.07 (m, 1H), 1.42 (s, 9H), 1.03 – 0.90 (m, 6H).

^{13}C NMR (100 MHz, $CDCl_3$) δ 172.3, 171.0, 156.4, 80.3, 62.8, 60.3, 54.8, 52.7, 28.4, 19.3, 18.2.

HRMS (ESI): m/z calcd. for $C_{14}H_{26}NO_6Na^+$ $[M+Na]^+$ 341.1683, found 341.1692.

IR (neat, cm^{-1}): 3312, 2966, 1744, 1656, 1505, 1366, 1161, 735.

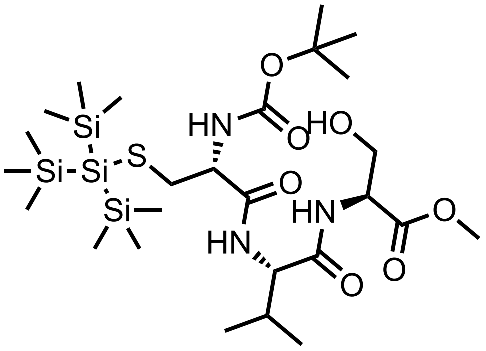

methyl N-(*tert*-butoxycarbonyl)-S-(1,1,1,3,3,3-hexamethyl-2-(trimethylsilyl)trisilan-2-yl)-L-cysteinyl-L-valyl-L-serinate (2z21)

White solid, 773.7 mg, 58%

^{1}H NMR (600 MHz, $CDCl_3$) δ 7.02 (d, J = 7.9 Hz, 1H), 6.76 (d, J = 8.5 Hz, 1H), 5.46 – 5.33 (m, 1H), 4.61 (dt, J = 7.5, 3.5 Hz, 1H), 4.30 – 4.16 (m, 2H), 3.93 (qd, J = 11.6, 3.5 Hz, 2H), 3.78 (s, 3H), 3.06 (s, 1H), 2.78 (dd, J = 11.9, 6.0 Hz, 1H), 2.35 – 2.12 (m, 1H), 1.45 (s, 9H), 0.99 (dd, J = 15.9, 6.8 Hz, 6H), 0.23 (s, 27H).

^{13}C NMR (100 MHz, $CDCl_3$) δ 171.1, 171.0, 170.7, 164.4, 80.9, 62.8, 56.1, 55.0, 52.8, 32.4, 28.4, 19.5, 18.1, 0.9.

^{29}Si NMR (119 MHz, $CDCl_3$) δ -11.14, -52.98.

HRMS (APCI): m/z calcd. for $C_{26}H_{58}N_3O_7SSi_4^+$ $[M+H]^+$ 668.3067, found 668.3058.

IR (neat, cm^{-1}): 3287, 2953, 1643, 1505, 1366, 1244, 1163, 1060, 831, 688, 623.

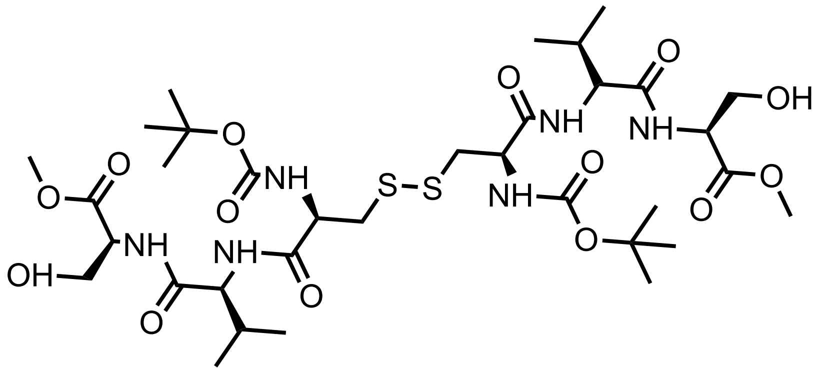

methyl N-(*tert*-butoxycarbonyl)-S-(((R)-2-((*tert*-butoxycarbonyl)amino)-3-(((R)-1-(((S)-3-hydroxy-1-methoxy-1-oxopropan-2-yl)amino)-3-methyl-1-oxobutan-2-yl)amino)-3-oxopropyl)thio)-L-cysteinyl-L-valyl-L-serinate (2z22)

White solid, 247.8 mg, 59%

^{1}H NMR (600 MHz, $CDCl_3$) δ 8.00 (s, 1H), 7.78 (s, 1H), 7.65 (s, 2H), 7.53 (d, J = 7.4 Hz, 1H), 7.43 (s, 1H), 5.81 (s, 1H), 5.57 (s, 1H), 5.44 – 5.34 (m, 1H), 4.78 – 4.69 (m, 2H), 4.58 (s, 1H), 4.50 (t, J = 8.1 Hz, 1H), 4.47 – 4.38 (m, 2H), 4.36 – 4.31 (m, 1H), 3.93 – 3.84 (m, 2H), 3.77 (s, 3H), 3.76 (s, 3H), 3.29 – 3.17 (m, 2H), 3.11 – 3.00 (m, 2H), 2.16 – 2.12 (m, 2H), 1.45 (s, 9H), 1.43 (s, 9H), 1.02 – 0.97 (m, 12H).

HRMS (ESI): m/z calcd. for $C_{34}H_{61}N_6O_{14}S_2^+$ $[M+H]^+$ 841.3671, found 841.3682.

IR (neat, cm^{-1}): 3286, 2959, 1744, 1692, 1637, 1520, 1367, 1226, 1315, 1046.

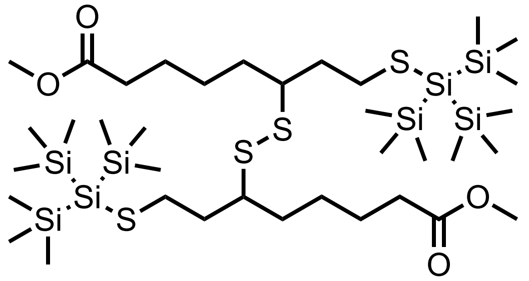

dimethyl 6,6'-disulfanediylbis(8-((1,1,1,3,3,3-hexamethyl-2-(trimethylsilyl)trisilan-2-yl)thio)octanoate) (2z23)

Colorless oil, 21.2 mg, 90% yield.

^{1}H NMR (400 MHz, $CDCl_3$) δ 3.67 (s, 3H), 2.80 – 2.72 (m, 1H), 2.63 (td, J = 7.8, 3.3 Hz, 2H), 2.31 (t, J = 7.5 Hz, 2H), 1.90 – 1.80 (m, 2H), 1.68 – 1.59 (m, 4H), 1.49 – 1.41 (m, 2H), 0.23 (s, 27H).

^{13}C NMR (100 MHz, $CDCl_3$) δ 174.1, 51.6, 51.3, 51.2, 37.5, 34.2, 34.1, 28.2, 26.4, 25.0, 0.9.

^{29}Si NMR (79 MHz, $CDCl_3$) δ -11.49, -55.60.

HRMS (ESI): m/z calcd. for $C_{36}H_{86}O_4S_4Si_8Na^+$ $[M+Na]^+$ 957.3456, found 957.3455.

IR (neat, cm^{-1}): 2948, 1740, 1436, 1244, 830, 688, 623, 509.

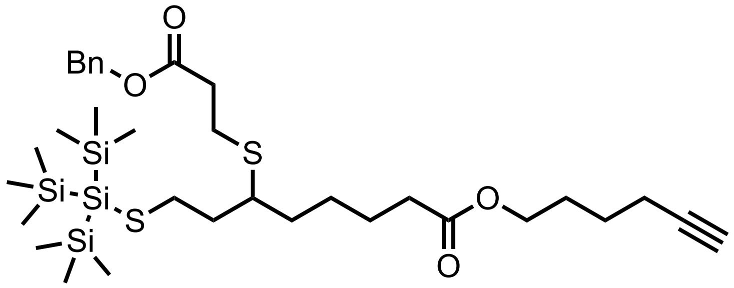

hex-5-yn-1-yl 6-((3-(benzyloxy)-3-oxopropyl)thio)-8-((1,1,1,3,3,3-hexamethyl-2-(trimethylsilyl)trisilan-2-yl)thio)octanoate (2z24)

Colorless oil, 59.1 mg, 85%

^{1}H NMR (600 MHz, $CDCl_3$) δ 7.38 – 7.31 (m, 5H), 5.13 (s, 2H), 4.09 (t, *J* = 6.5 Hz, 2H), 2.81 – 2.70 (m, 3H), 2.68 – 2.60 (m, 4H), 2.29 (t, *J* = 7.5 Hz, 2H), 2.23 (td, *J* = 7.0, 2.6 Hz, 2H), 1.96 (t, *J* = 2.7 Hz, 1H), 1.88 – 1.71 (m, 4H), 1.65 – 1.58 (m, 4H), 1.58 – 1.49 (m, 2H), 1.50 – 1.39 (m, 2H), 0.23 (s, 27H).

^{13}C NMR (150 MHz, $CDCl_3$) δ 173.6, 171.7, 135.8, 128.6, 128.3, 83.9, 68.7, 66.5, 63.8, 45.0, 38.0, 35.2, 34.7, 34.2, 28.0, 27.7, 26.3, 25.3, 25.0, 24.9, 18.1, 0.8.

^{29}Si NMR (119 MHz, $CDCl_3$) δ -11.46, -55.48.

HRMS (APCI): m/z calcd. for $C_{33}H_{60}O_4S_2Si_4^+$ $[M+H]^+$ 697.3083, found 697.3088.

IR (neat, cm^{-1}): 3310, 2950, 2921, 1735, 1455, 1244, 1165, 834, 747, 691, 624.

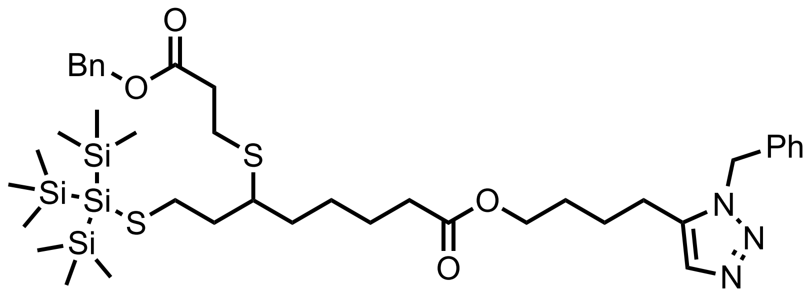

4-(1-benzyl-1H-1,2,3-triazol-5-yl)butyl 6-((3-(benzyloxy)-3-oxopropyl)thio)-8-

((1,1,1,3,3,3-hexamethyl-2-(trimethylsilyl)trisilan-2-yl)thio)octanoate (2z25)

Colorless oil, 34.3 mg, 83%

^{1}H NMR (400 MHz, $CDCl_3$) δ 7.46 – 7.36 (m, 8H), 7.34 – 7.28 (m, 2H), 5.55 (s, 2H), 5.19 (s, 2H), 4.13 (t, J = 6.1 Hz, 2H), 2.89 – 2.75 (m, 5H), 2.75 – 2.64 (m, 4H), 2.33 (t, J = 7.5 Hz, 2H), 1.94 – 1.70 (m, 7H), 1.70 – 1.46 (m, 6H), 0.29 (d, J = 0.9 Hz, 27H).

^{13}C NMR (100 MHz, $CDCl_3$) δ 173.74, 171.85, 148.32, 135.94, 135.07, 129.21, 128.70, 128.39, 128.12, 120.72, 66.61, 64.13, 54.15, 45.17, 38.17, 35.28, 34.85, 34.31, 28.37, 28.10, 26.45, 25.97, 25.44, 25.37, 25.01, 0.93.

^{29}Si NMR (79 MHz, $CDCl_3$) δ -11.47, -55.48.

HRMS (APCI): m/z calcd. for $C_{40}H_{68}N_3O_4S_2Si_4^+$ $[M+H]^+$ 830.3723, found 830.3727.

IR (neat, cm^{-1}): 2943, 2853, 1733, 1456, 1244, 1168, 835, 747, 695, 623.

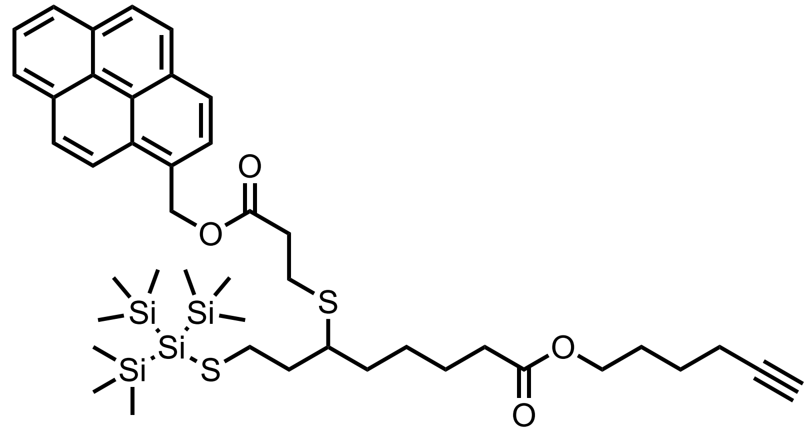

hex-5-yn-1-yl 8-((1,1,1,3,3,3-hexamethyl-2-(trimethylsilyl)trisilan-2-yl)thio)-6-((3-oxo-3-(pyren-1-ylmethoxy)propyl)thio)octanoate (2z26)

Colorless oil, 53.3 mg, 65%

^{1}H NMR (400 MHz, $CDCl_3$) δ 8.27 (d, J = 9.2 Hz, 1H), 8.23 – 8.18 (m, 2H), 8.17 – 8.13 (m, 2H), 8.07 – 8.00 (m, 4H), 5.86 (s, 2H), 4.07 (t, J = 6.5 Hz, 2H), 2.81 – 2.76 (m, 2H), 2.74 – 2.70 (m, 1H), 2.69 – 2.60 (m, 4H), 2.27 – 2.19 (m, 4H), 1.96 (t, J = 2.6 Hz, 1H), 1.83 – 1.71 (m, 4H), 1.61 – 1.55 (m, 4H), 1.54 – 1.48 (m, 2H), 1.46 – 1.37 (m, 2H), 0.22 (s, 27H).

^{13}**C NMR** (100 MHz, $CDCl_3$) δ 173.6, 171.9, 131.9, 131.3, 130.8, 129.6, 128.8, 128.3, 127.9, 127.9, 127.4, 126.2, 125.6, 125.6, 125.0, 124.7, 123.0, 84.0, 68.9, 65.0, 63.8, 45.1, 38.1, 35.3, 34.8, 34.2, 28.0, 27.8, 26.4, 25.4, 25.0, 25.0, 18.2, 0.9.

^{29}**Si NMR** (79 MHz, $CDCl_3$) δ -11.48, -55.49.

HRMS (ESI): m/z calcd. for $C_{43}H_{64}O_4S_2Si_4Na^+$ $[M+Na]^+$ 843.3216, found 843.3214.

IR (neat, cm^{-1}): 2946, 2851, 1733, 1244, 1165, 834, 689, 623.

10. X-Ray Crystallographic Data

The absolute configuration of product **2m** was assigned based on the crystal X-ray. A colorless rodlike crystal of **2m** (CCDC number: 2464851) was obtained by vaporization of DCM solution of compound **2m.**

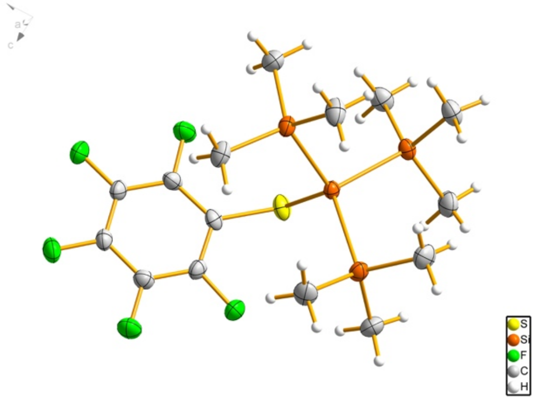

Identification code	**2m**
Empirical formula	$C_{15}H_{27}F_5SSi_4$
Formula weight	446.78
Temperature/K	101(2)
Crystal system	triclinic
Space group	P-1
a/Å	9.0235(4)
b/Å	9.3553(5)
c/Å	15.1632(4)
α/°	89.334(3)
β/°	86.137(3)
γ/°	65.888(4)
Volume/Å^3	1165.52(9)
Z	2
ρ_{calc}g/cm^3	1.273
μ/mm^{-1}	3.553
F(000)	468.0
Crystal size/mm^3	0.15 × 0.13 × 0.03
Radiation	Cu Kα (λ = 1.54184)
2Θ range for data collection/°	5.842 to 159.928
Index ranges	$-11 \le h \le 10$, $-11 \le k \le 10$, $-18 \le l \le 15$
Reflections collected	11221
Independent reflections	4546 [R_{int} = 0.0541, R_{sigma} = 0.0777]
Data/restraints/parameters	4546/0/235
Goodness-of-fit on F^2	1.021
Final R indexes [I>=2σ (I)]	R_1 = 0.0443, wR_2 = 0.1183
Final R indexes [all data]	R_1 = 0.0703, wR_2 = 0.1269
Largest diff. peak/hole / e Å^{-3}	0.40/-0.41

The absolute configuration of product **2o** was assigned based on the crystal X-ray. A colorless rodlike crystal of **2o** (CCDC number: 2464846) was obtained by vaporization of DCM solution of compound **2o.**

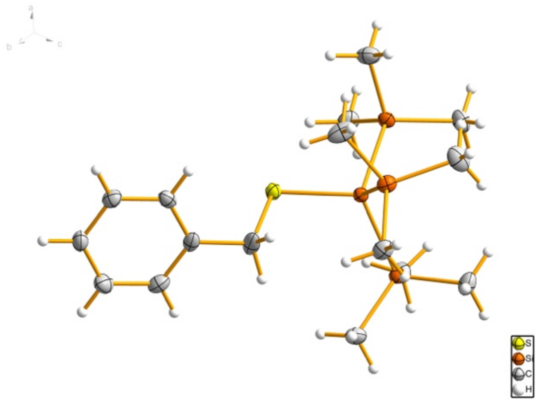

Identification code	**2o**
Empirical formula	$C_{16}H_{34}SSi_4$
Formula weight	370.85
Temperature/K	100.2(8)
Crystal system	monoclinic
Space group	$P2_1/n$
a/Å	10.08020(10)
b/Å	26.9585(2)
c/Å	25.0976(2)
α/°	90
β/°	92.7240(10)
γ/°	90
Volume/Å^3	6812.49(10)
Z	12
ρ_{calc}g/cm^3	1.085
μ/mm^{-1}	3.225
F(000)	2424.0
Crystal size/mm^3	0.4 × 0.3 × 0.2
Radiation	Cu Kα (λ = 1.54184)
2Θ range for data collection/°	4.814 to 147.942
Index ranges	$-12 \leq h \leq 10$, $-32 \leq k \leq 31$, $-27 \leq l \leq 31$
Reflections collected	38680
Independent reflections	13373 [R_{int} = 0.0200, R_{sigma} = 0.0203]
Data/restraints/parameters	13373/0/595
Goodness-of-fit on F^2	1.059
Final R indexes [I>=2σ (I)]	R_1 = 0.0269, wR_2 = 0.0728
Final R indexes [all data]	R_1 = 0.0297, wR_2 = 0.0741
Largest diff. peak/hole / e Å^{-3}	0.29/-0.23

The absolute configuration of product **2t** was assigned based on the crystal X-ray. A colorless rodlike crystal of **2t** (CCDC number: 2469752) was obtained by vaporization of DCM solution of compound **2t.**

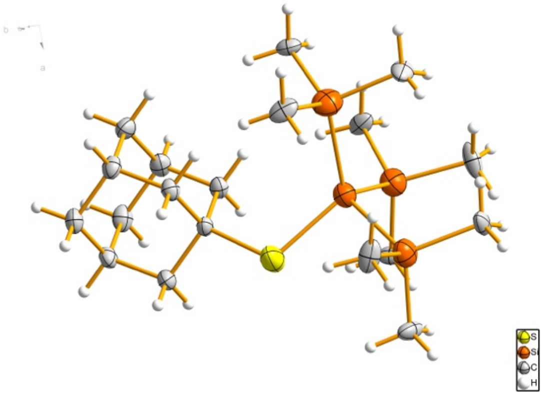

Identification code	**2t**
Empirical formula	$C_{19}H_{42}SSi_4$
Formula weight	414.94
Temperature/K	100.2(4)
Crystal system	monoclinic
Space group	$P2_1/c$
a/Å	13.00310(10)
b/Å	10.84600(10)
c/Å	17.98370(10)
α/°	90
β/°	93.7150(10)
γ/°	90
Volume/Å^3	2530.94(3)
Z	2
ρ_{calc}g/cm^3	1.089
μ/mm^{-1}	2.937
F(000)	912.0
Crystal size/mm^3	0.15 × 0.12 × 0.08
Radiation	Cu Kα (λ = 1.54184)
2Θ range for data collection/°	6.812 to 147.906
Index ranges	$-16 \leq h \leq 11$, $-13 \leq k \leq 12$, $-20 \leq l \leq 22$
Reflections collected	13838
Independent reflections	4985 [R_{int} = 0.0247, R_{sigma} = 0.0283]
Data/restraints/parameters	4985/0/226
Goodness-of-fit on F^2	1.046
Final R indexes [I>=2σ (I)]	R_1 = 0.0303, wR_2 = 0.0802
Final R indexes [all data]	R_1 = 0.0324, wR_2 = 0.0816
Largest diff. peak/hole / e Å^{-3}	0.30/-0.22

The absolute configuration of product **2w** was assigned based on the crystal X-ray. A colorless rodlike crystal of **2w** (CCDC number: 2469753) was obtained by vaporization of DCM solution of compound **2w.**

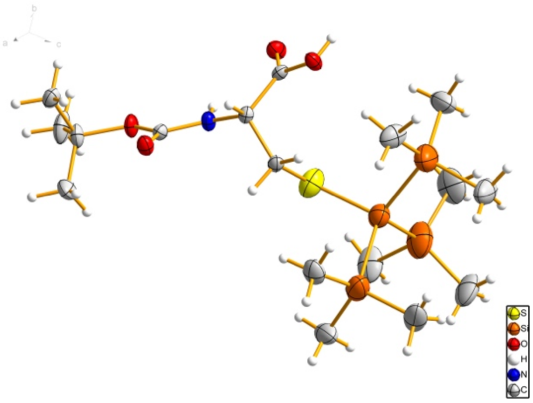

Identification code	**2w**
Empirical formula	$C_{17}H_{41}NO_4SSi_4$
Formula weight	467.93
Temperature/K	103(4)
Crystal system	monoclinic
Space group	$P2_1$
a/Å	9.7792(2)
b/Å	9.4268(2)
c/Å	16.1765(3)
α/°	90
β/°	106.123(2)
γ/°	90
Volume/Å^3	1432.60(5)
Z	2
ρ_{calc}g/cm^3	1.085
μ/mm^{-1}	2.763
F(000)	508.0
Crystal size/mm^3	0.2 × 0.04 × 0.03
Radiation	Cu Kα (λ = 1.54184)
2Θ range for data collection/°	5.686 to 147.542
Index ranges	$-12 \leq h \leq 11$, $-11 \leq k \leq 6$, $-20 \leq l \leq 19$
Reflections collected	7822
Independent reflections	3835 [R_{int} = 0.0523, R_{sigma} = 0.0657]
Data/restraints/parameters	3835/1/261
Goodness-of-fit on F^2	1.085
Final R indexes [I>=2σ (I)]	R_1 = 0.0493, wR_2 = 0.1201
Final R indexes [all data]	R_1 = 0.0631, wR_2 = 0.1235
Largest diff. peak/hole / e Å^{-3}	0.39/-0.28

The absolute configuration of product **2z1** was assigned based on the crystal X-ray. A colorless rodlike crystal of **2z1** (CCDC number: 2464852) was obtained by vaporization of DCM solution of compound **2z1.**

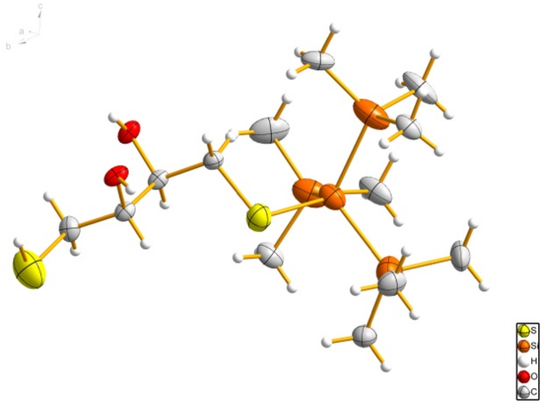

Identification code	**2z1**
Empirical formula	$C_{13}H_{36}O_2S_2Si_4$
Formula weight	400.90
Temperature/K	99.9(5)
Crystal system	triclinic
Space group	P-1
a/Å	9.92260(10)
b/Å	13.4447(2)
c/Å	20.0186(2)
α/°	107.8590(10)
β/°	101.3820(10)
γ/°	95.4090(10)
Volume/Å^3	2457.40(5)
Z	4
ρ_{calc}g/cm^3	1.084
μ/mm^{-1}	3.845
F(000)	872.0
Crystal size/mm^3	0.25 × 0.13 × 0.1
Radiation	Cu Kα (λ = 1.54184)
2Θ range for data collection/°	7.004 to 147.948
Index ranges	$-12 \leq h \leq 12$, $-16 \leq k \leq 16$, $-20 \leq l \leq 24$
Reflections collected	25645
Independent reflections	9612 [Rint = 0.0323, Rsigma = 0.0350]
Data/restraints/parameters	9612/0/406
Goodness-of-fit on F^2	1.045
Final R indexes [I>=2σ (I)]	R_1 = 0.0395, wR_2 = 0.0978
Final R indexes [all data]	R_1 = 0.0479, wR_2 = 0.1006
Largest diff. peak/hole / e Å^{-3}	0.60/-0.67

The absolute configuration of product **2z2** was assigned based on the crystal X-ray. A colorless rodlike crystal of **2z2** (CCDC number: 2469758) was obtained by vaporization of DCM solution of compound **2z2**.

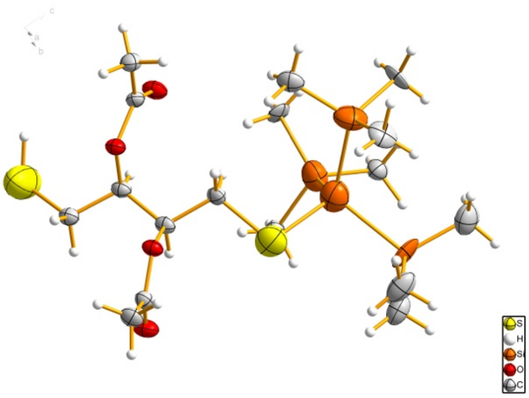

Identification code	**2z2**
Empirical formula	$C_{17}H_{40}O_4S_2Si_4$
Formula weight	1939.87
Temperature/K	108(2)
Crystal system	monoclinic
Space group	$P2_1/c$
a/Å	9.39500(10)
b/Å	16.9041(2)
c/Å	35.5143(3)
α/°	90
β/°	93.0810(10)
γ/°	90
Volume/Å^3	5632.02(10)
Z	2
ρ_{calc}g/cm^3	1.144
μ/mm^{-1}	3.493
F(000)	2096.0
Crystal size/mm^3	0.18 × 0.15 × 0.1
Radiation	Cu Kα (λ = 1.54184)
2Θ range for data collection/°	4.984 to 147.966
Index ranges	$-10 \le h \le 11, -14 \le k \le 20, -44 \le l \le 43$
Reflections collected	31716
Independent reflections	11088 [R_{int} = 0.0283, R_{sigma} = 0.0338]
Data/restraints/parameters	11088/793/747
Goodness-of-fit on F^2	1.047
Final R indexes [I>=2σ (I)]	R_1 = 0.0433, wR_2 = 0.1175
Final R indexes [all data]	R_1 = 0.0495, wR_2 = 0.1217
Largest diff. peak/hole / e Å^{-3}	0.66/-0.54

11. Copies of ^{1}H NMR, ^{13}C NMR, and ^{19}F NMR spectra of products

^{1}H NMR of compound 2a (400 MHz, $CDCl_3$)

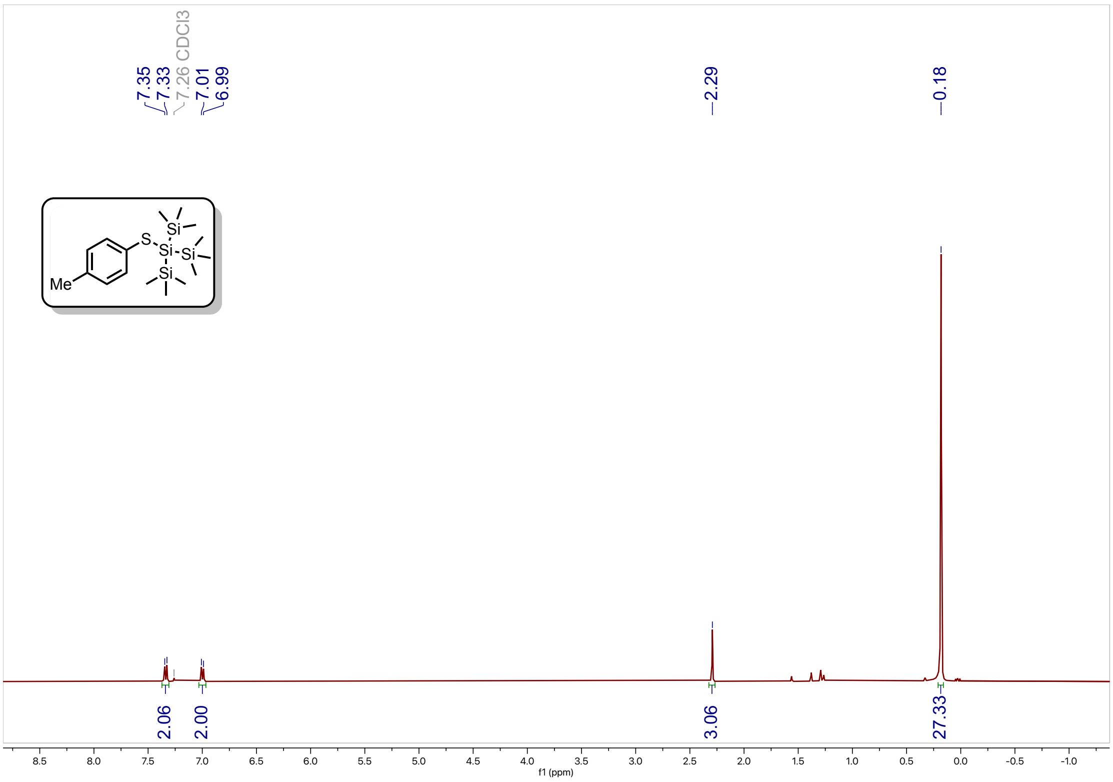

^{13}C NMR of compound 2a (100 MHz, $CDCl_3$)

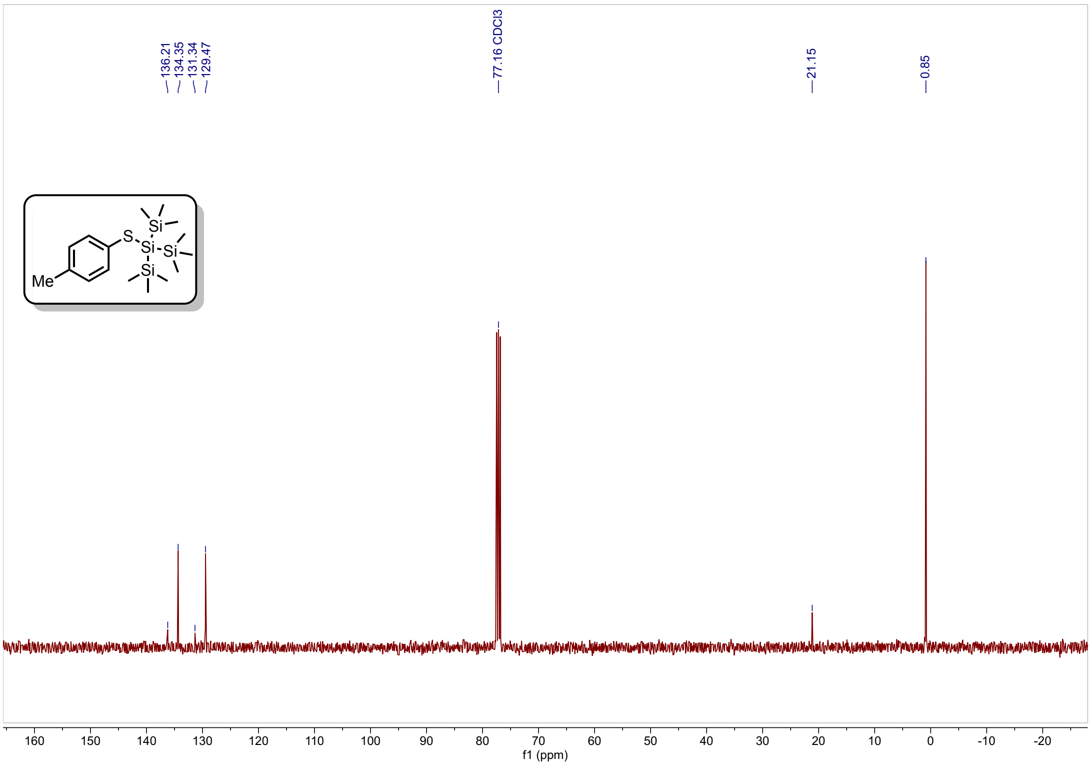

^{29}Si NMR of compound 2a (79 MHz, $CDCl_3$)

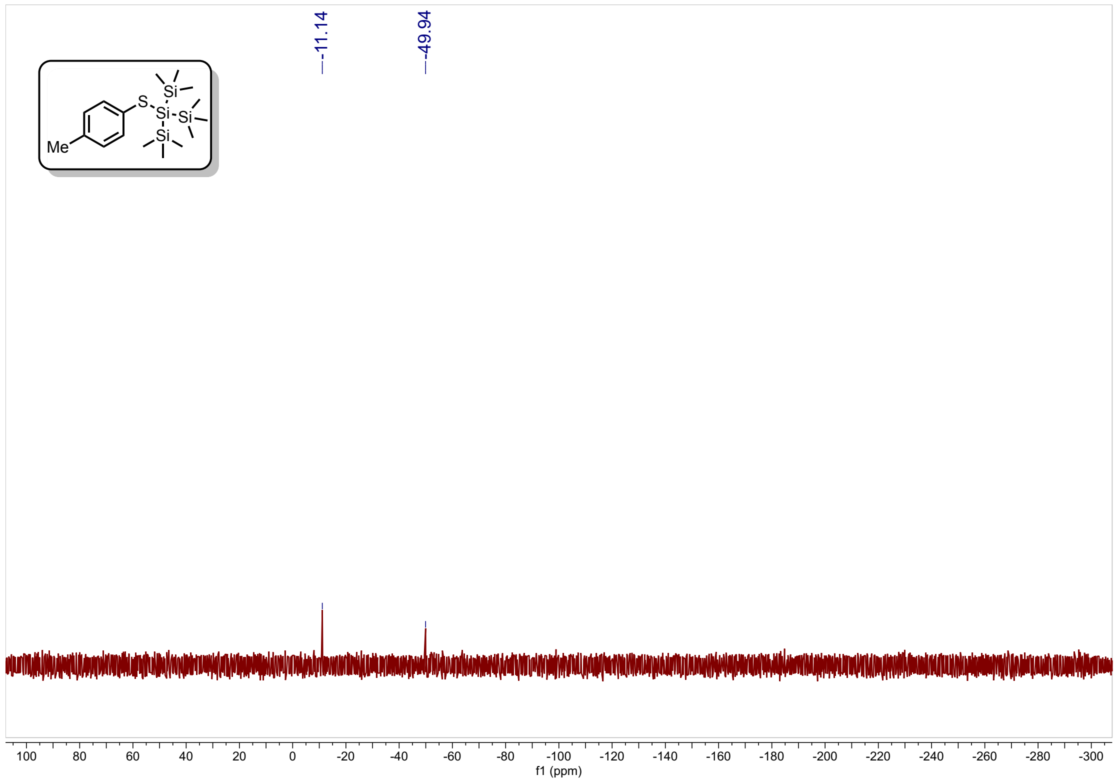

^{1}H NMR of compound 2b (400 MHz, $CDCl_3$)

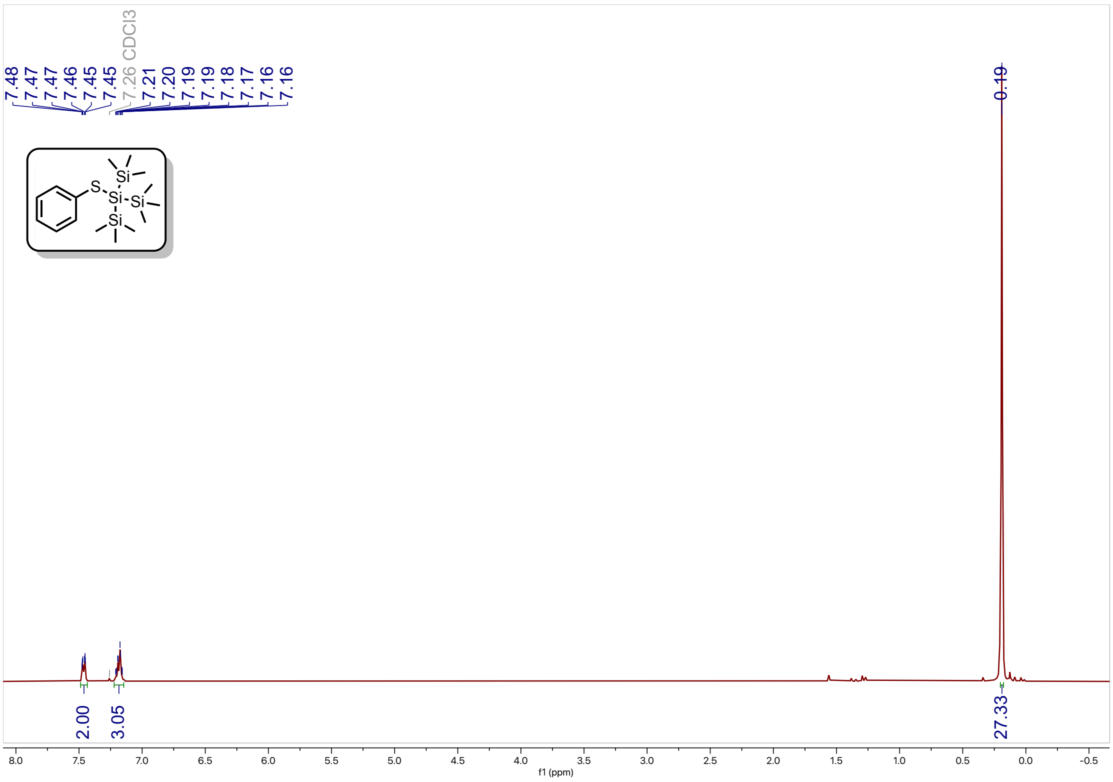

^{13}C NMR of compound 2b (100 MHz, $CDCl_3$)

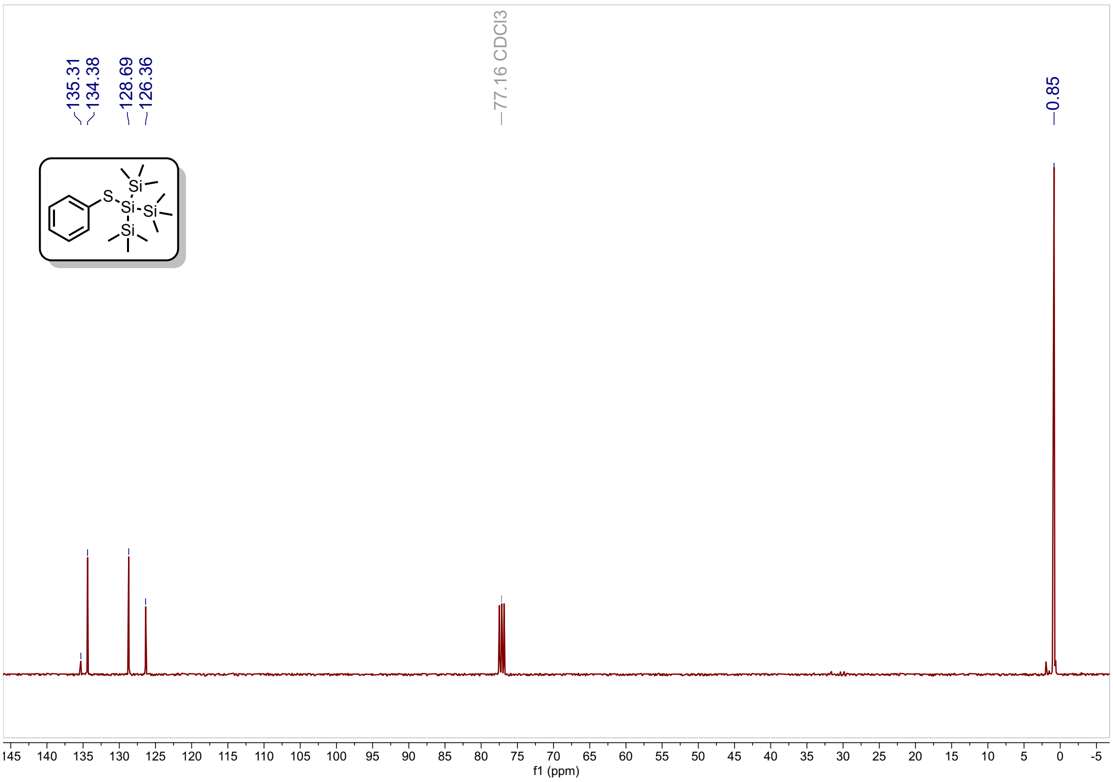

^{29}Si NMR of compound 2b (79 MHz, $CDCl_3$)

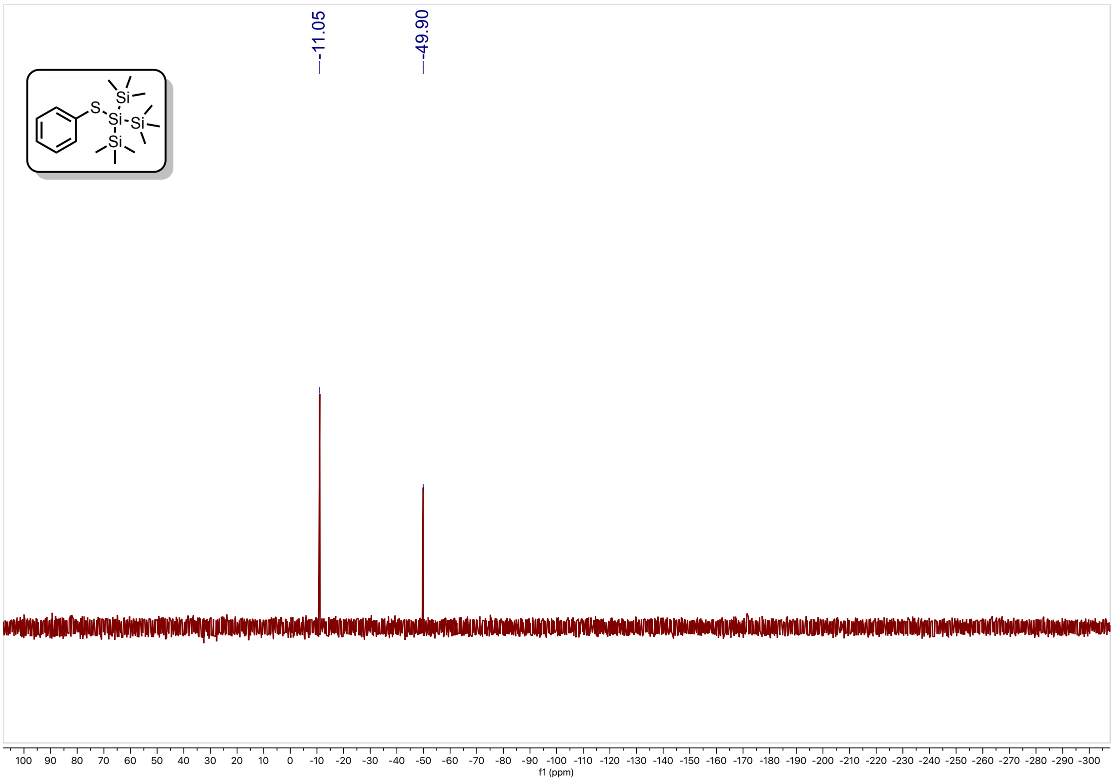

^{1}H NMR of compound 2c (400 MHz, $CDCl_3$)

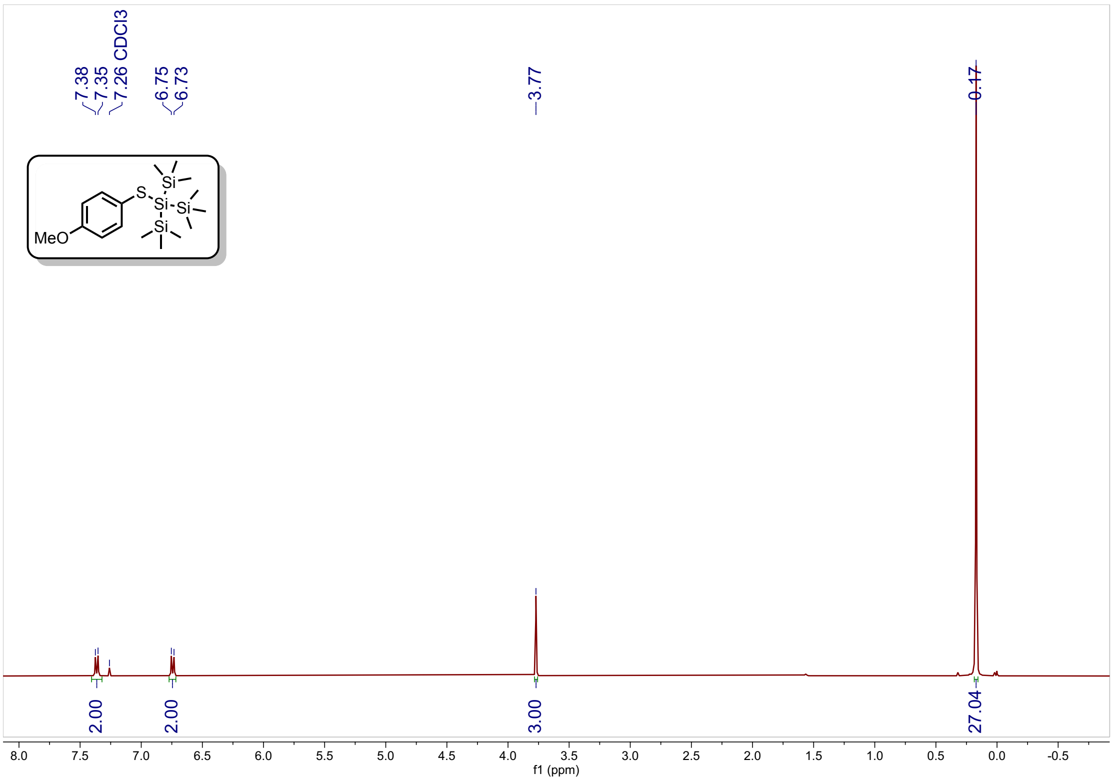

^{13}C NMR of compound 2c (100 MHz, $CDCl_3$)

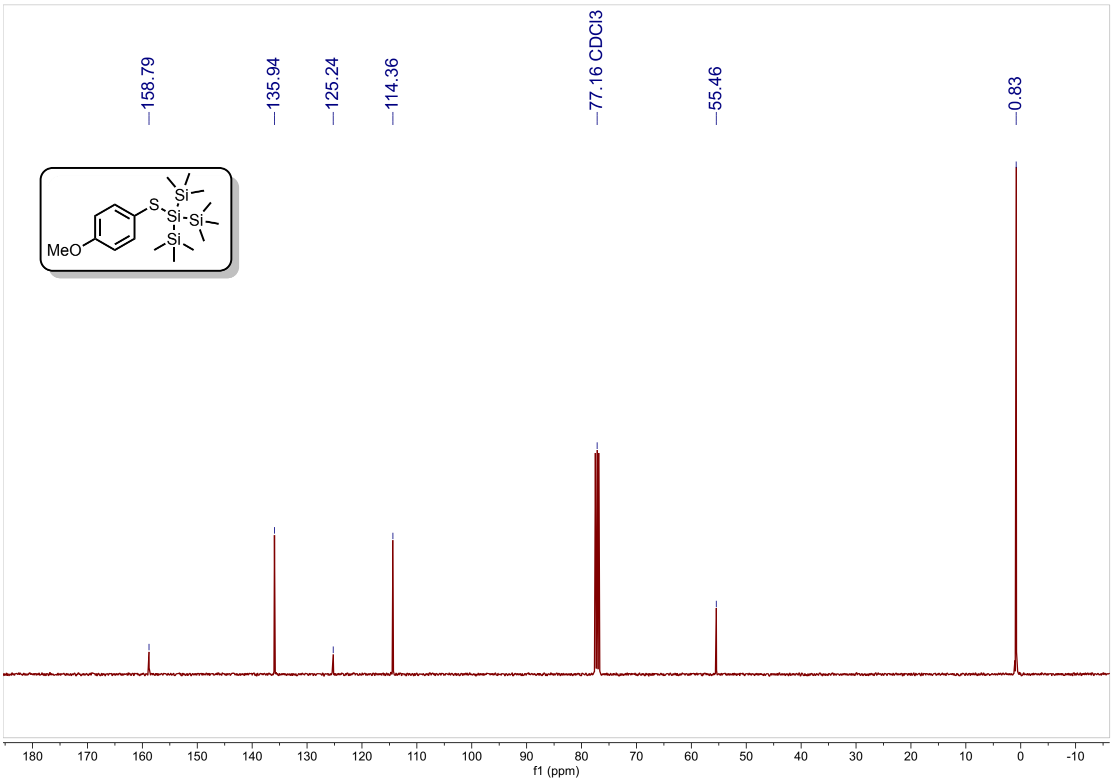

^{29}Si NMR of compound 2c (79 MHz, CDCl$_3$)

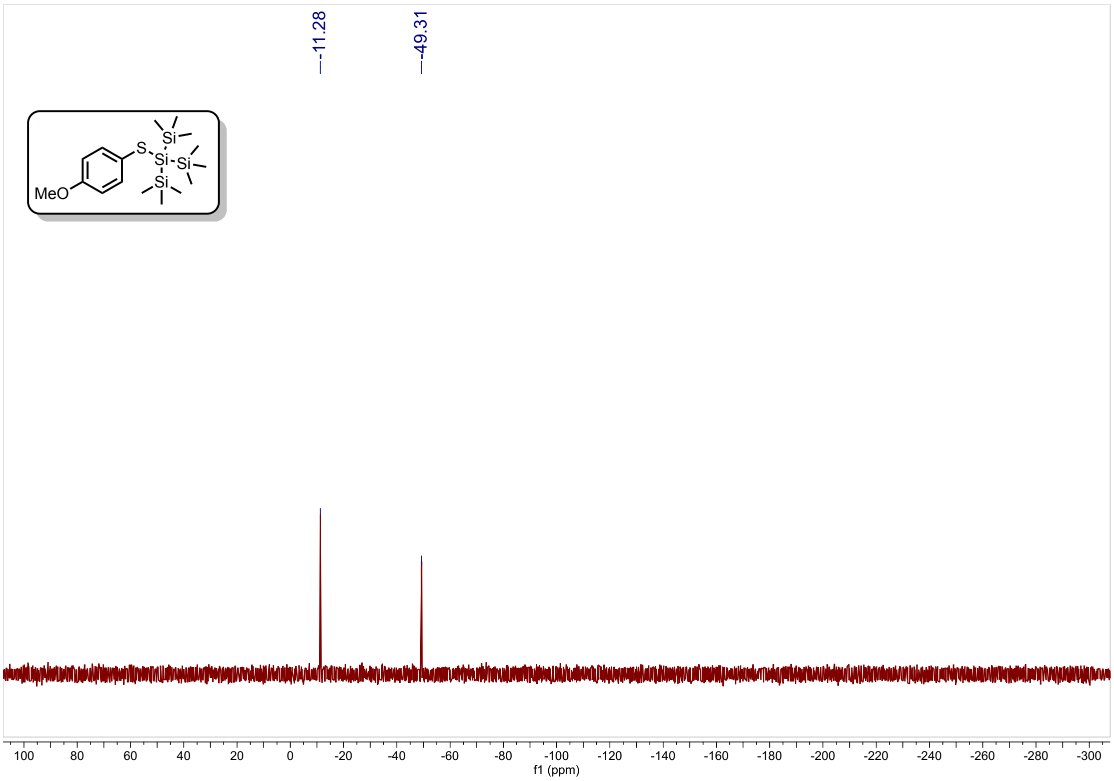

^{1}H NMR of compound 2d (400 MHz, $CDCl_3$)

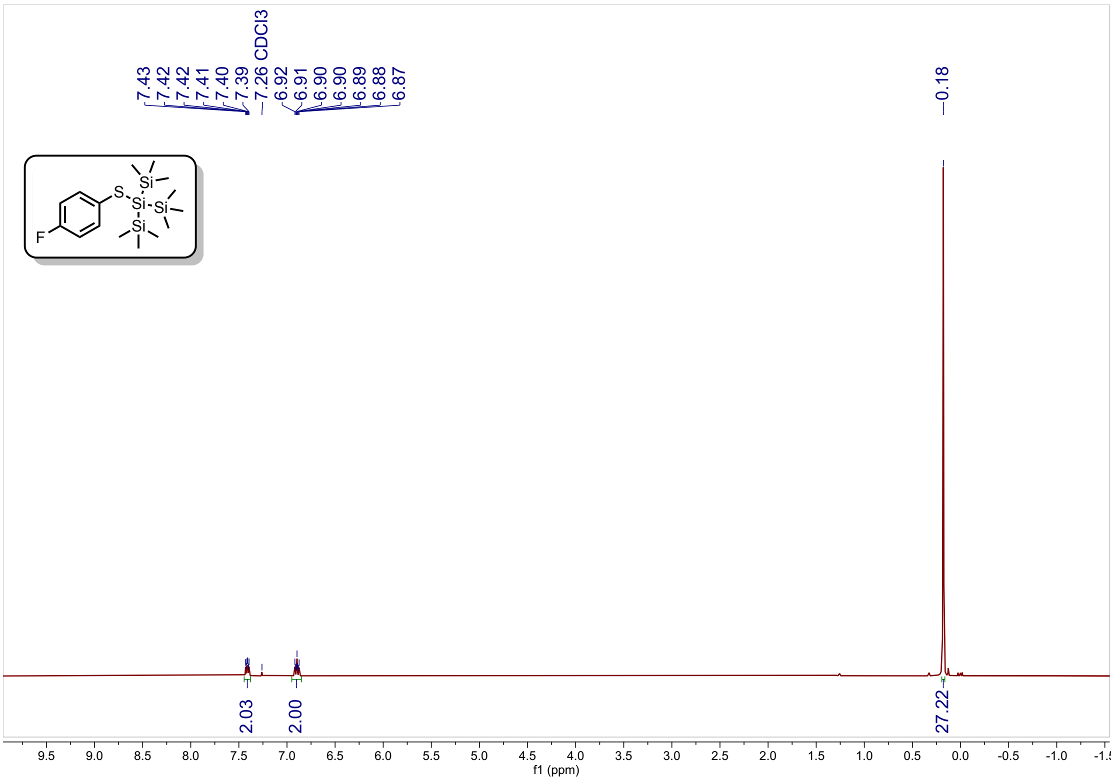

^{13}C NMR of compound 2d (100 MHz, $CDCl_3$)

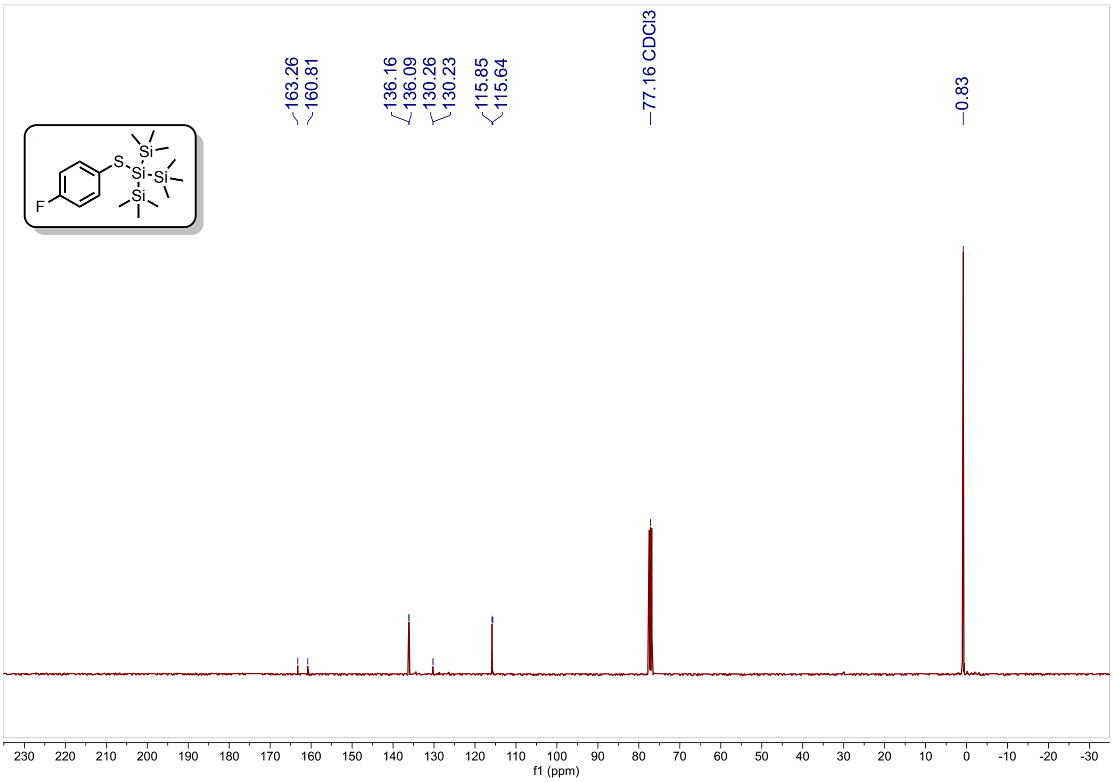

^{19}F NMR of compound 2d (376 MHz, $CDCl_3$)

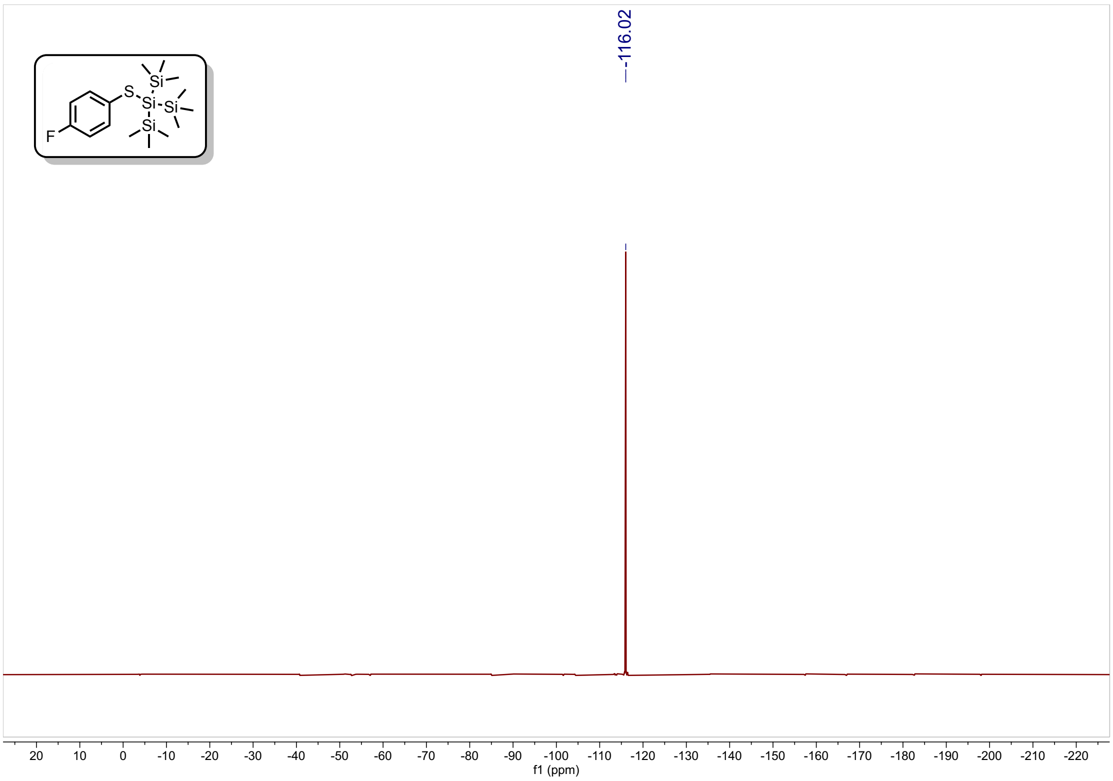

^{29}Si NMR of compound 2d (79 MHz, $CDCl_3$)

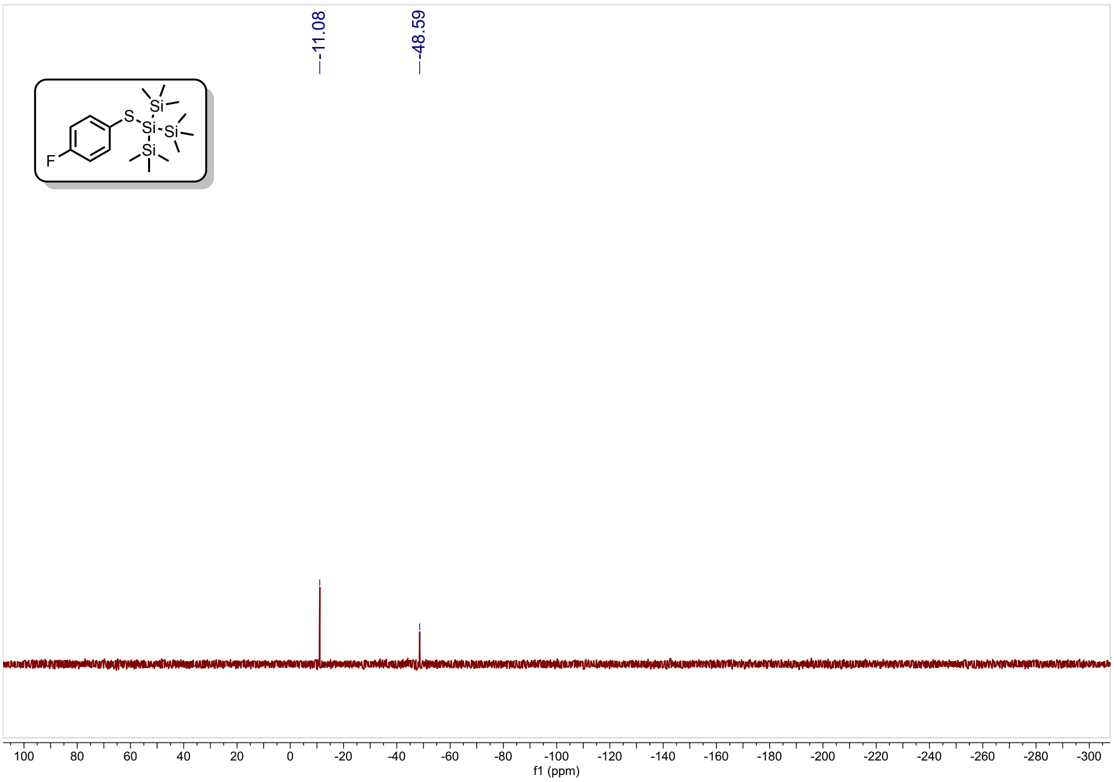

^{1}H NMR of compound 2e (400 MHz, $CDCl_3$)

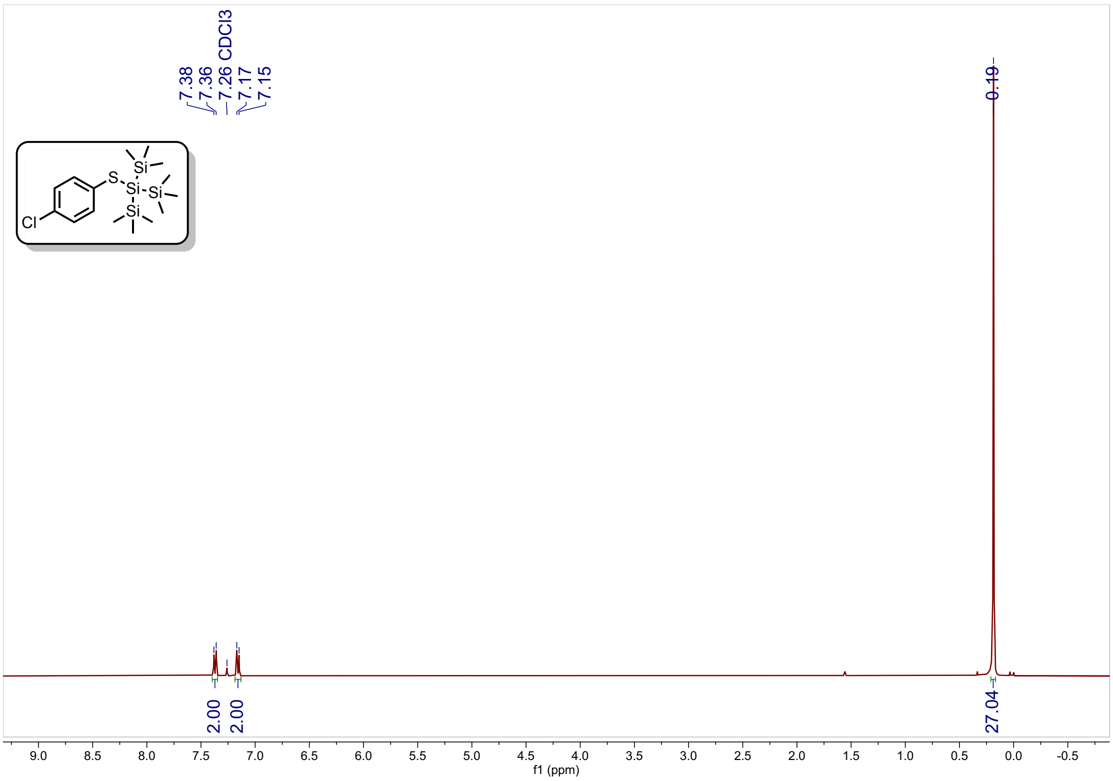

^{13}C NMR of compound 2e (100 MHz, $CDCl_3$)

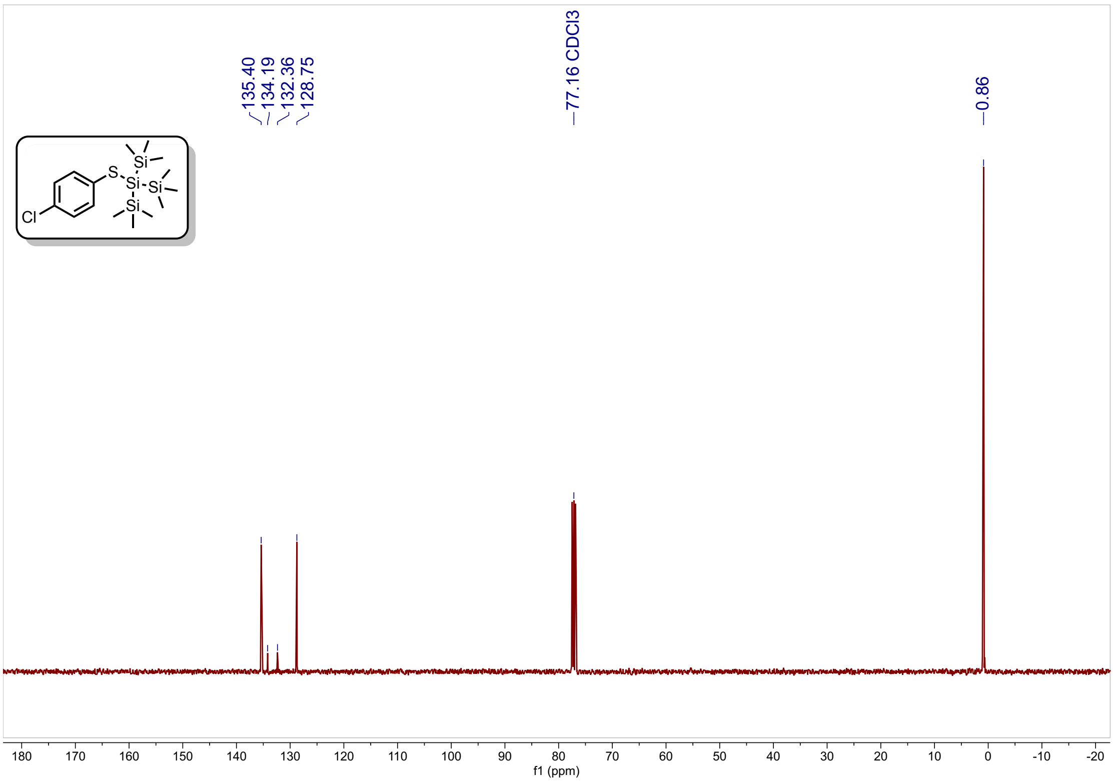

^{29}Si NMR of compound 2e (79 MHz, $CDCl_3$)

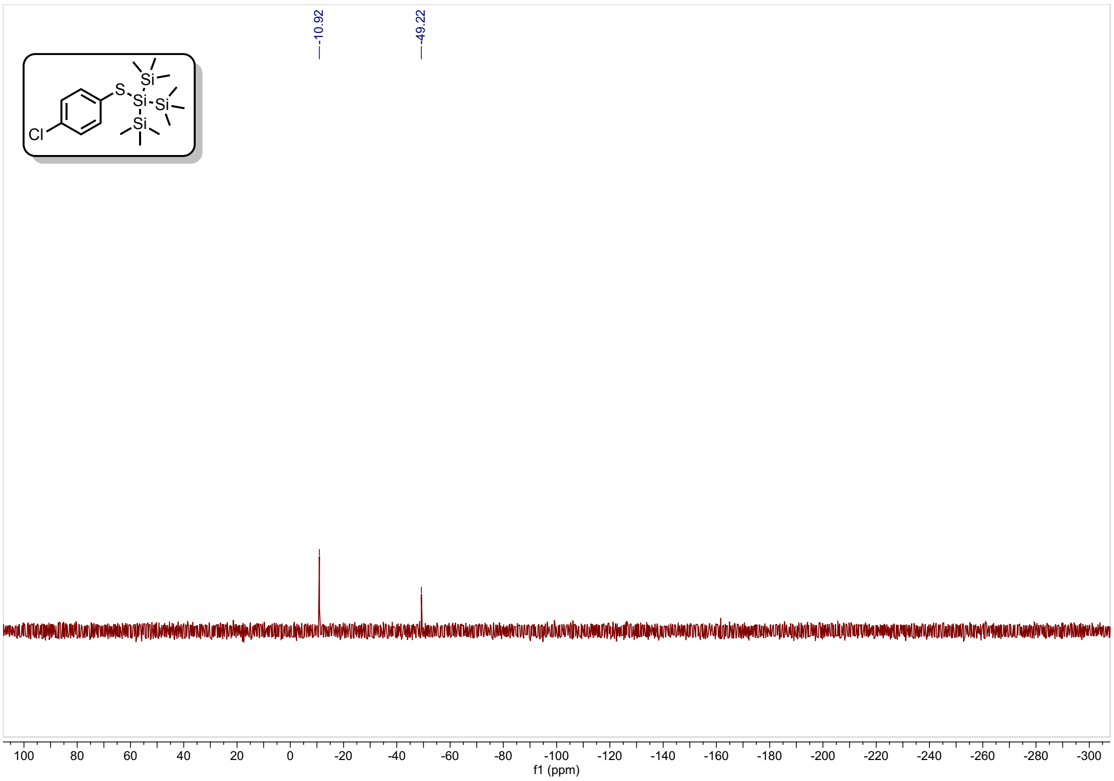

^{1}H NMR of compound 2f (400 MHz, $CDCl_3$)

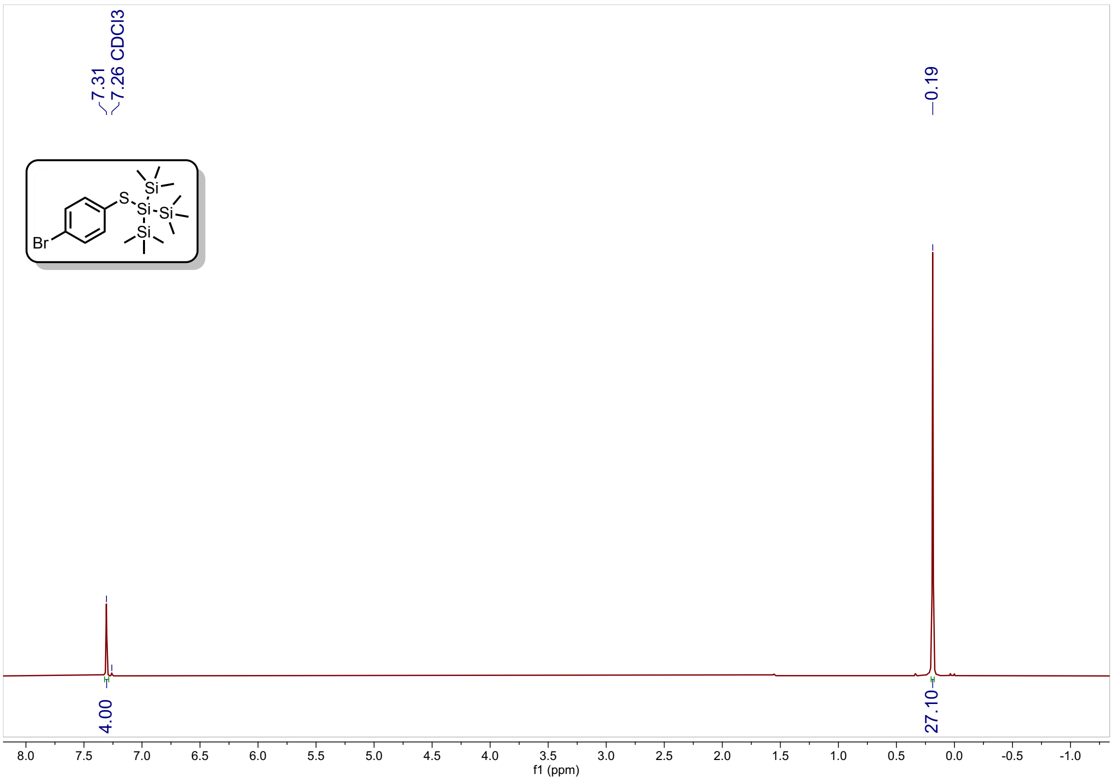

^{13}C NMR of compound 2f (100 MHz, $CDCl_3$)

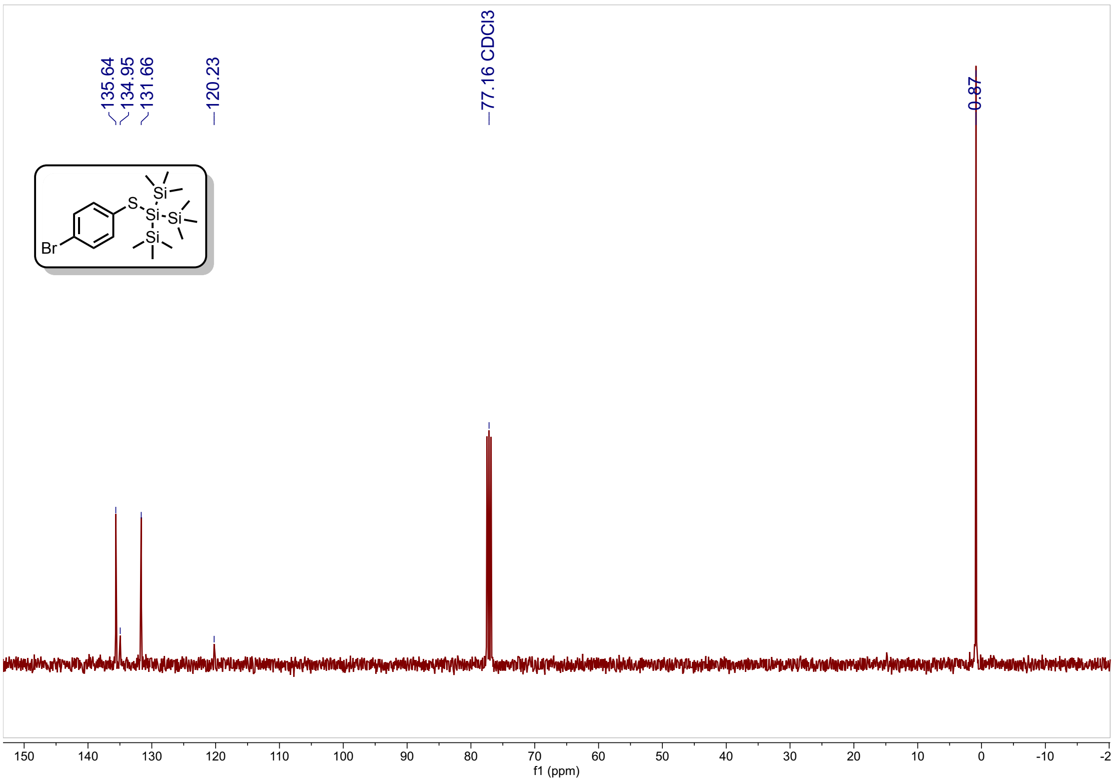

^{29}Si NMR of compound 2f (79 MHz, CDCl$_3$)

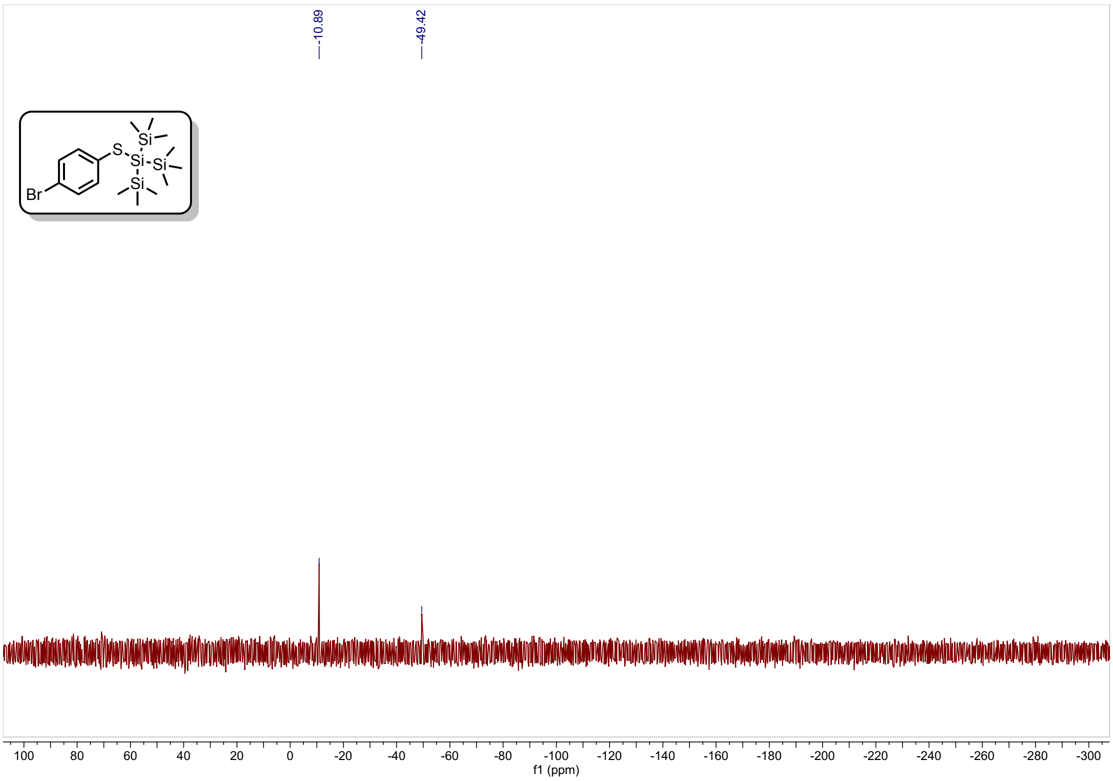

^{1}H NMR of compound 2g (400 MHz, $CDCl_3$)

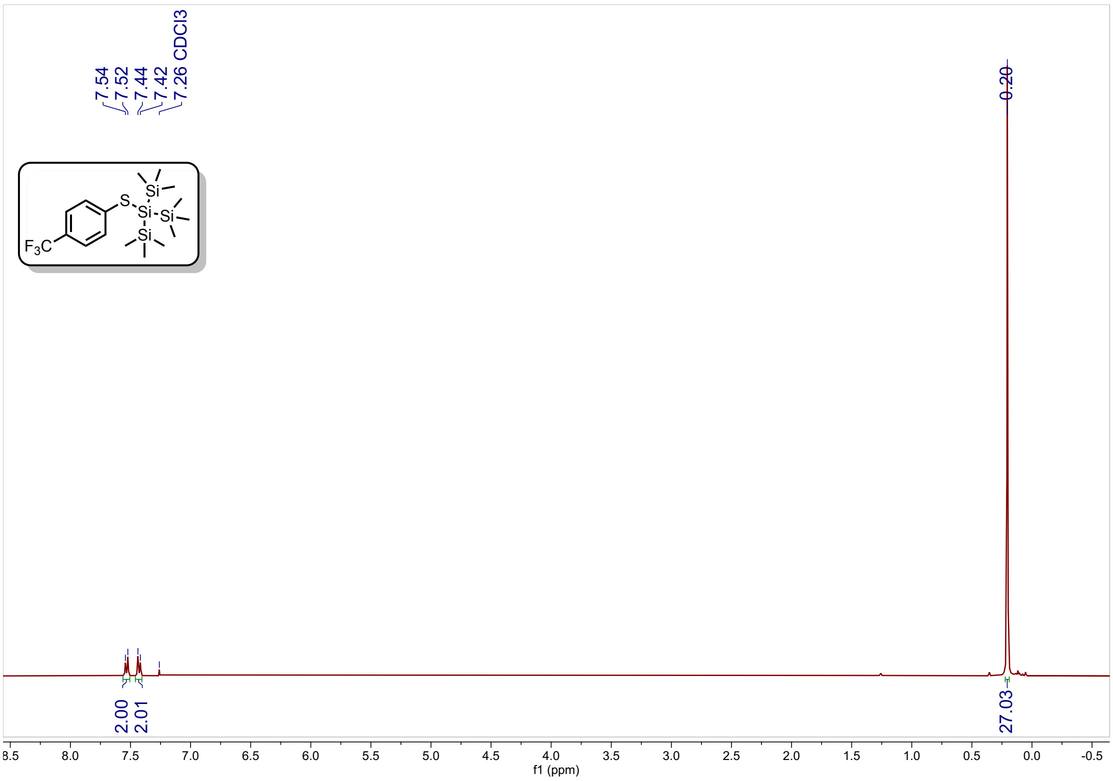

^{13}C NMR of compound 2g (150 MHz, $CDCl_3$)

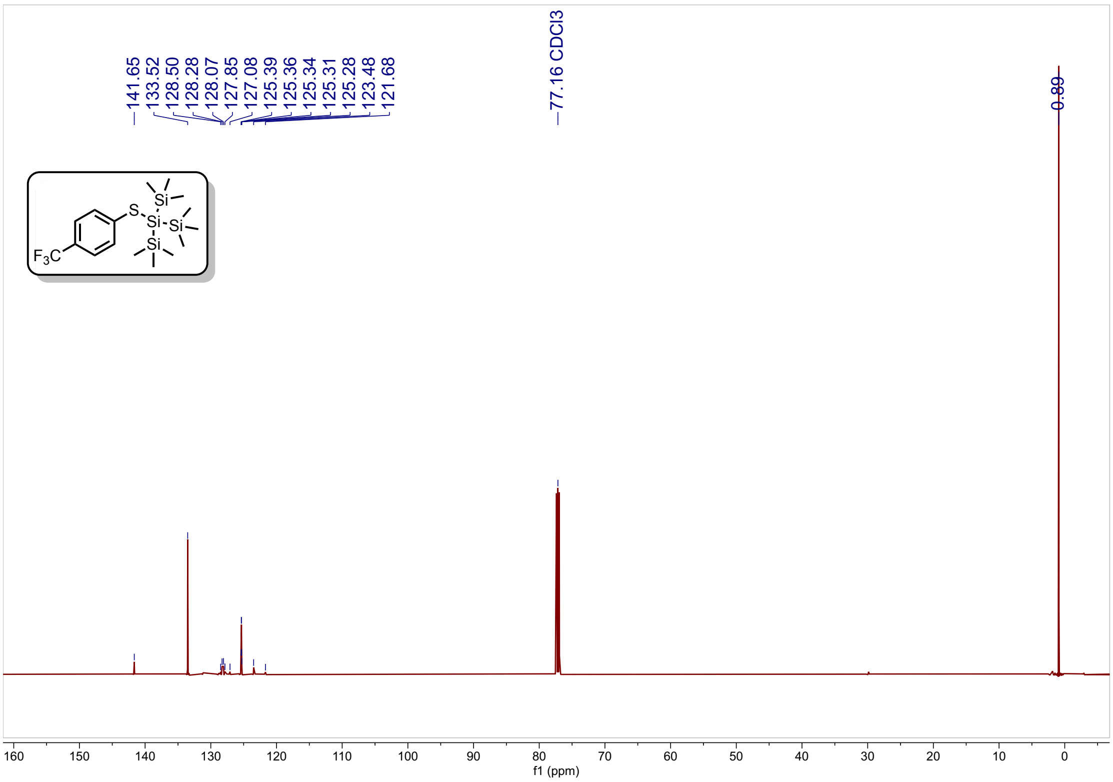

^{19}F NMR of compound 2g (376 MHz, $CDCl_3$)

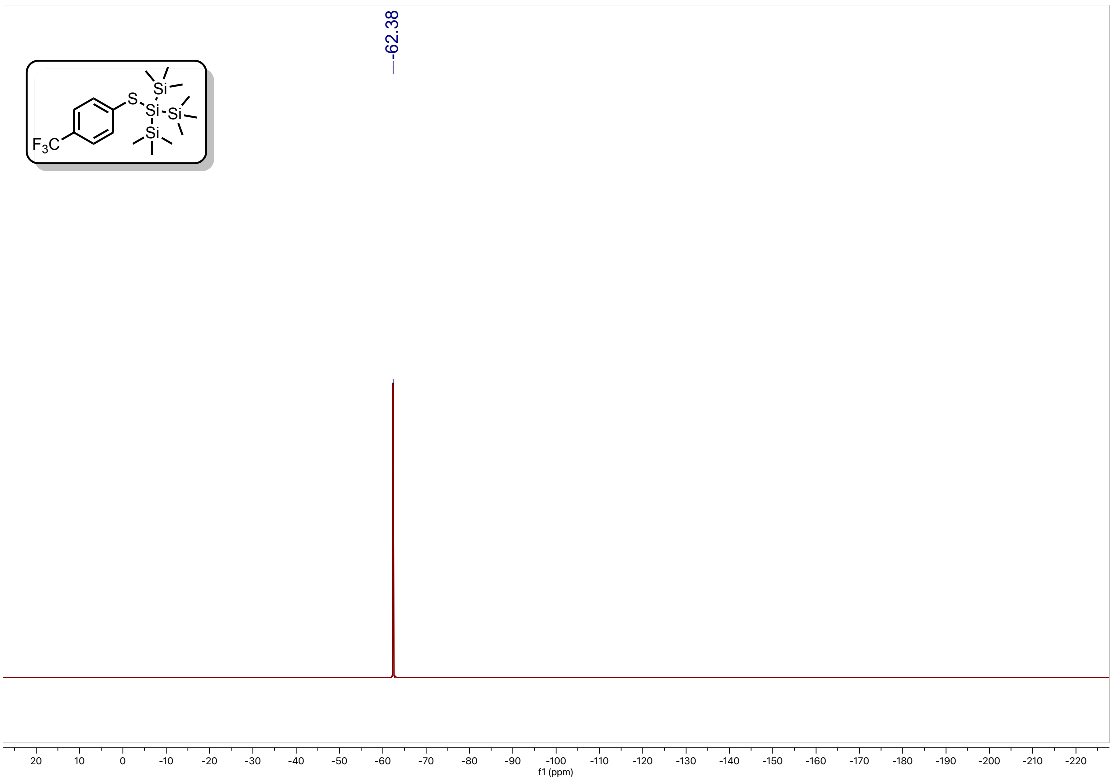

^{29}Si NMR of compound 2g (79 MHz, $CDCl_3$)

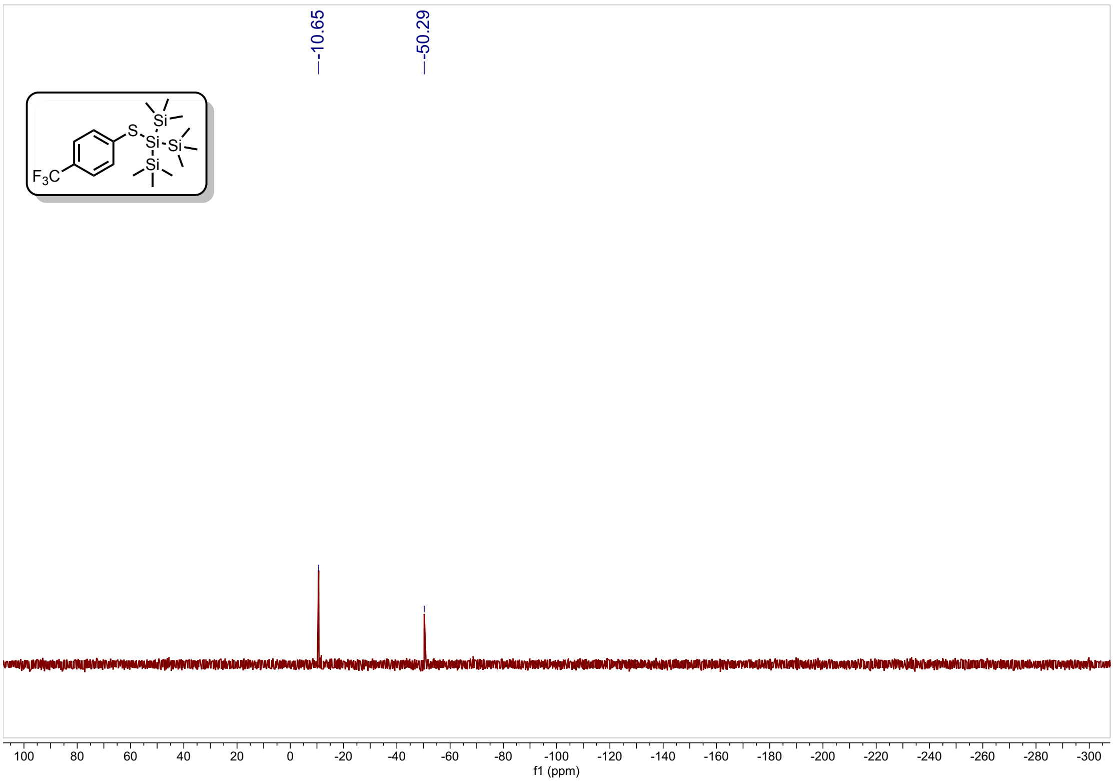

^{1}H NMR of compound 2h (400 MHz, $CDCl_3$)

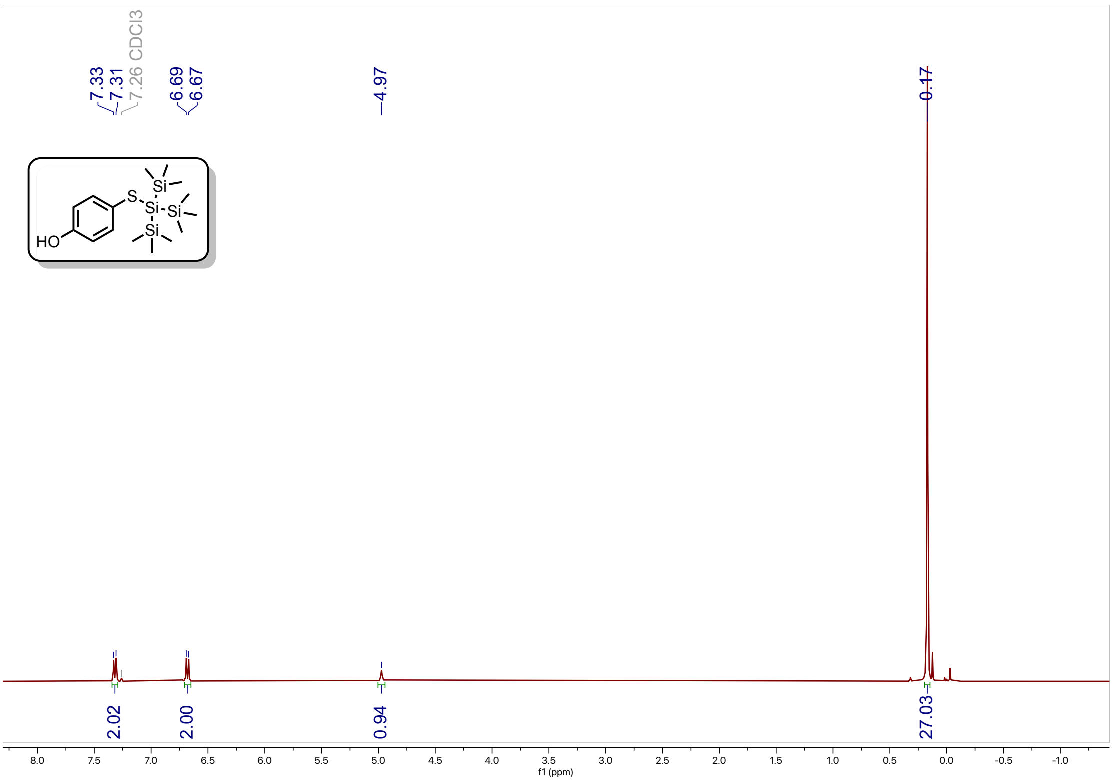

^{13}C NMR of compound 2h (100 MHz, $CDCl_3$)

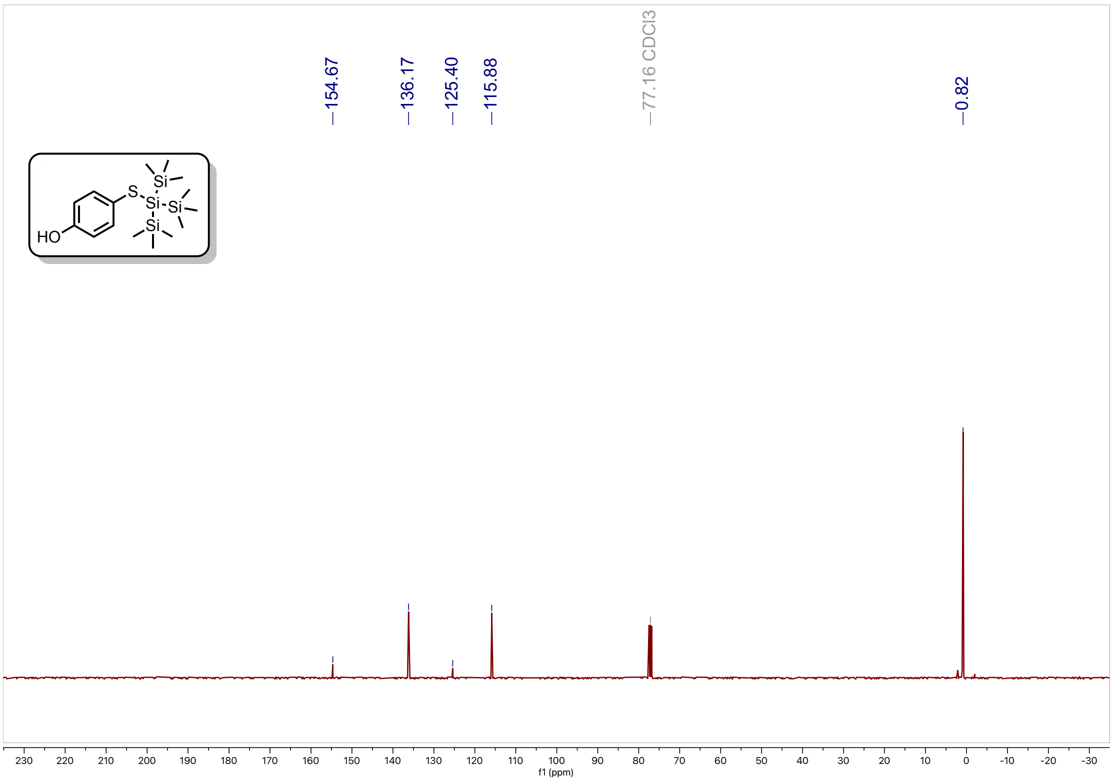

^{29}Si NMR of compound 2h (79 MHz, $CDCl_3$)

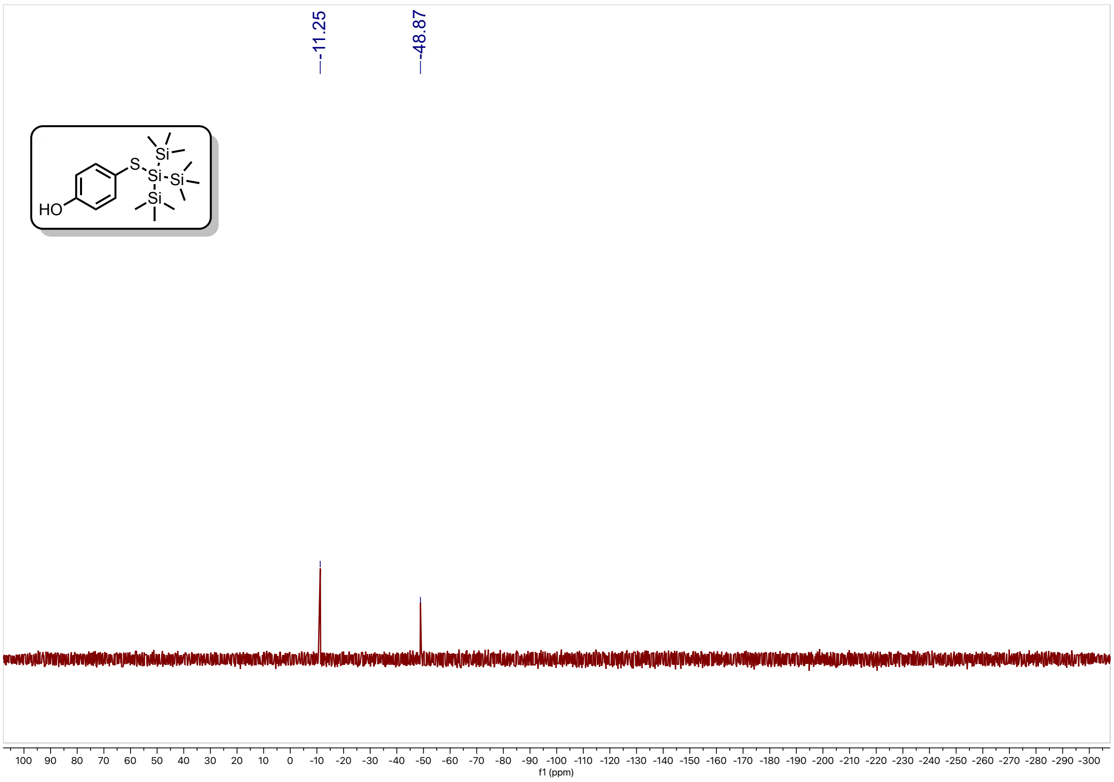

^{1}H NMR of compound 2i (400 MHz, $CDCl_3$)

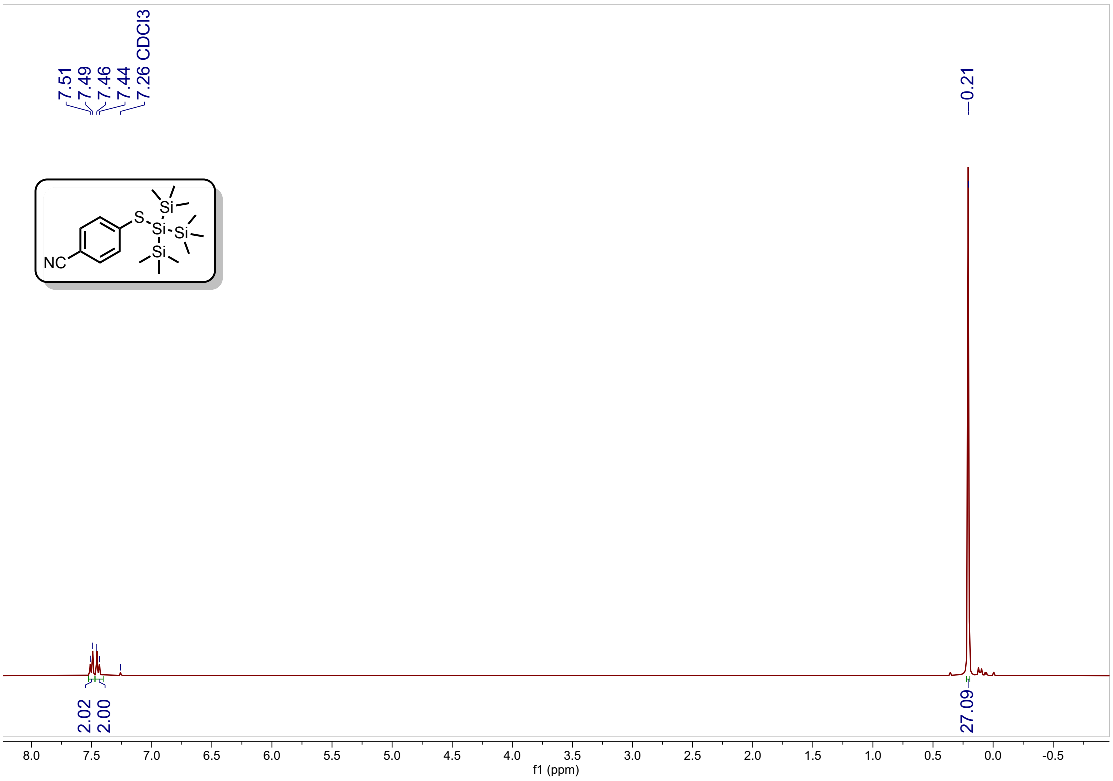

^{13}C NMR of compound 2i (100 MHz, $CDCl_3$)

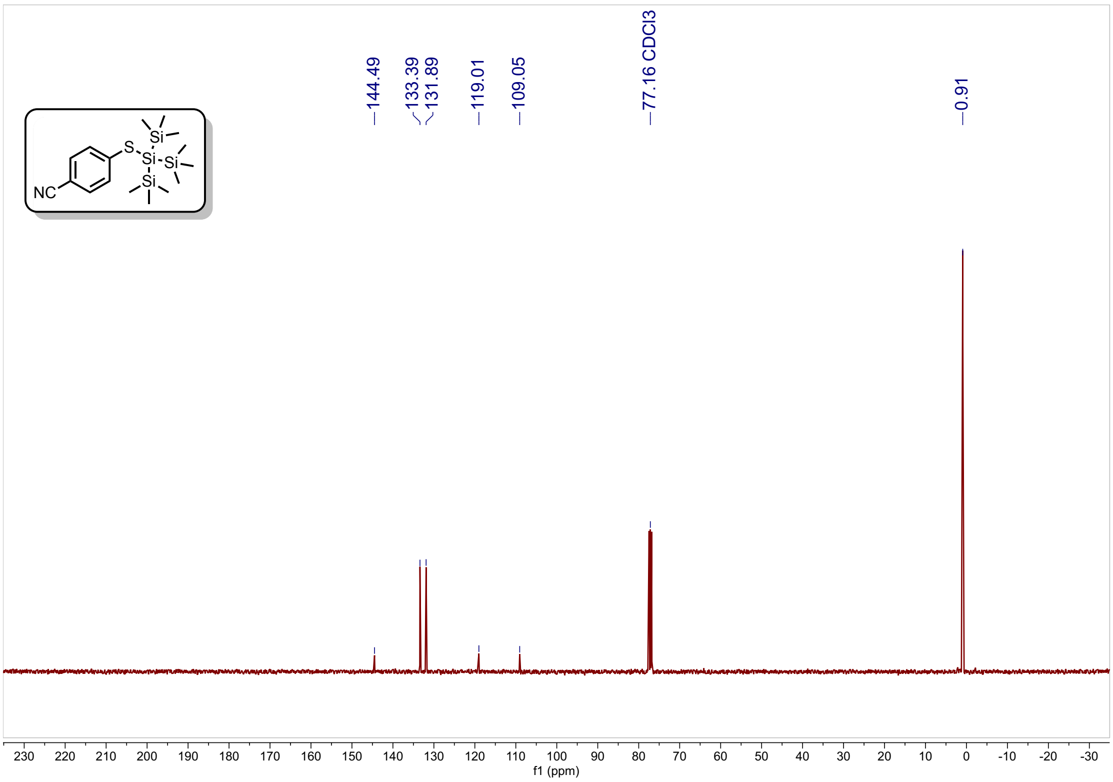

^{29}Si NMR of compound 2i (79 MHz, $CDCl_3$)

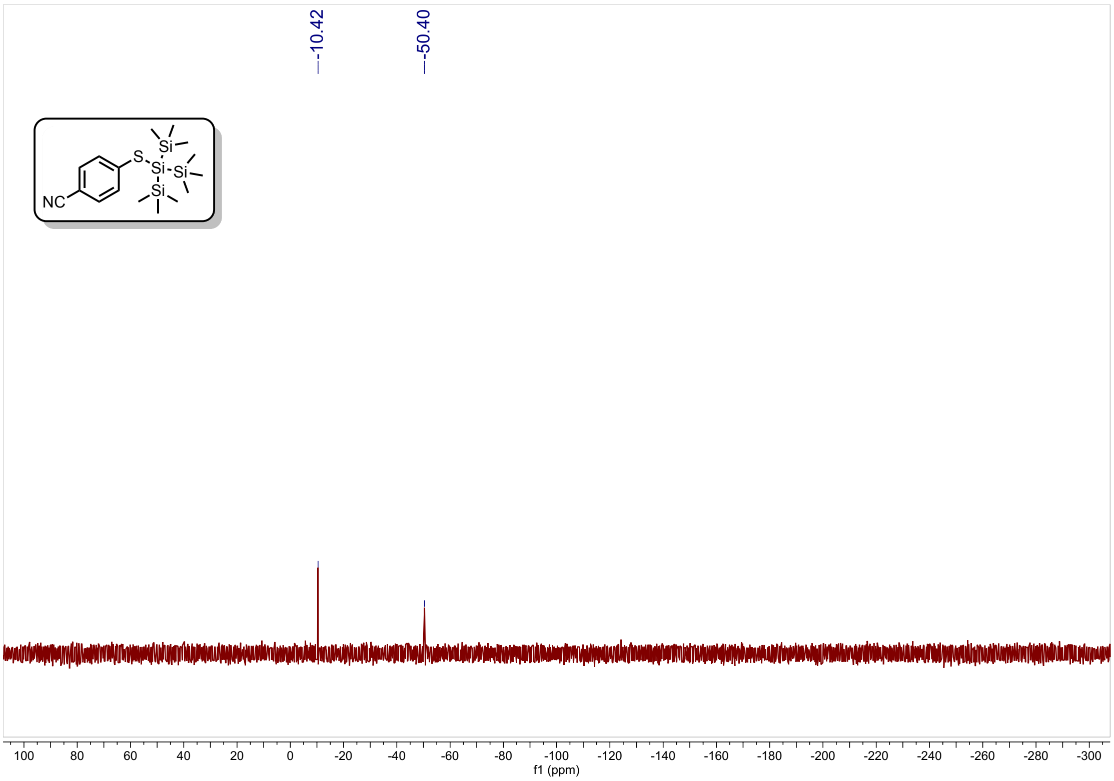

^{1}H NMR of compound 2j (600 MHz, $CDCl_3$)

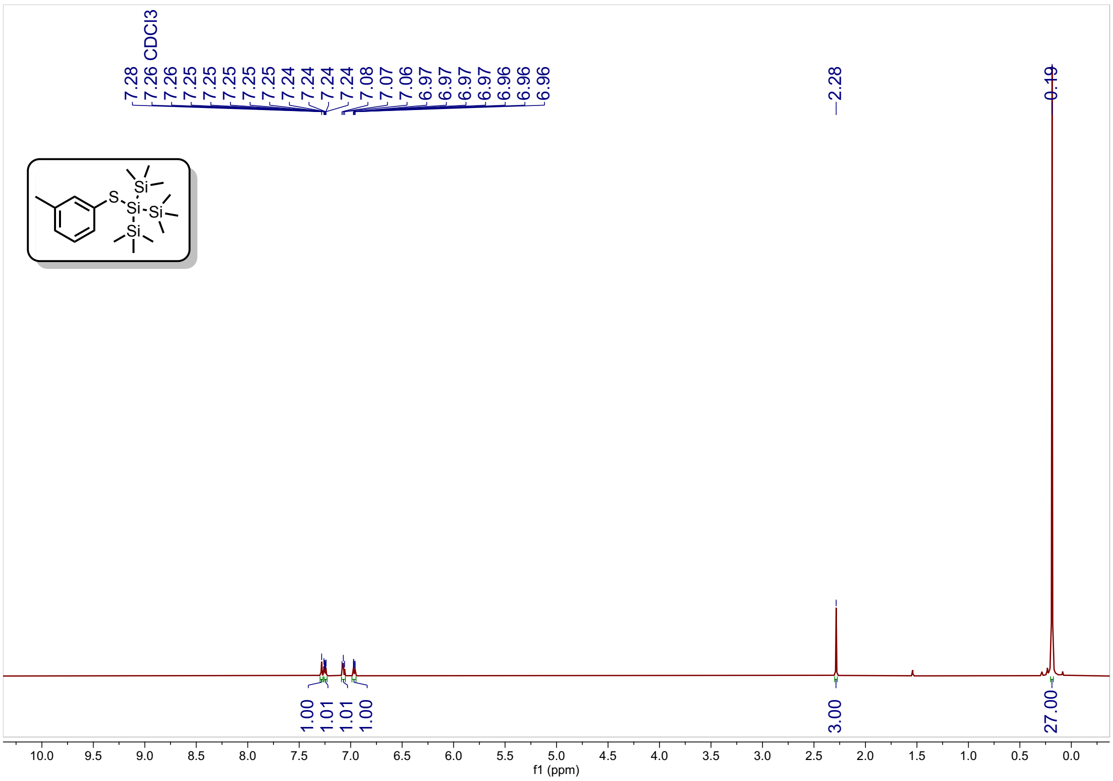

^{13}C NMR of compound 2j (150 MHz, $CDCl_3$)

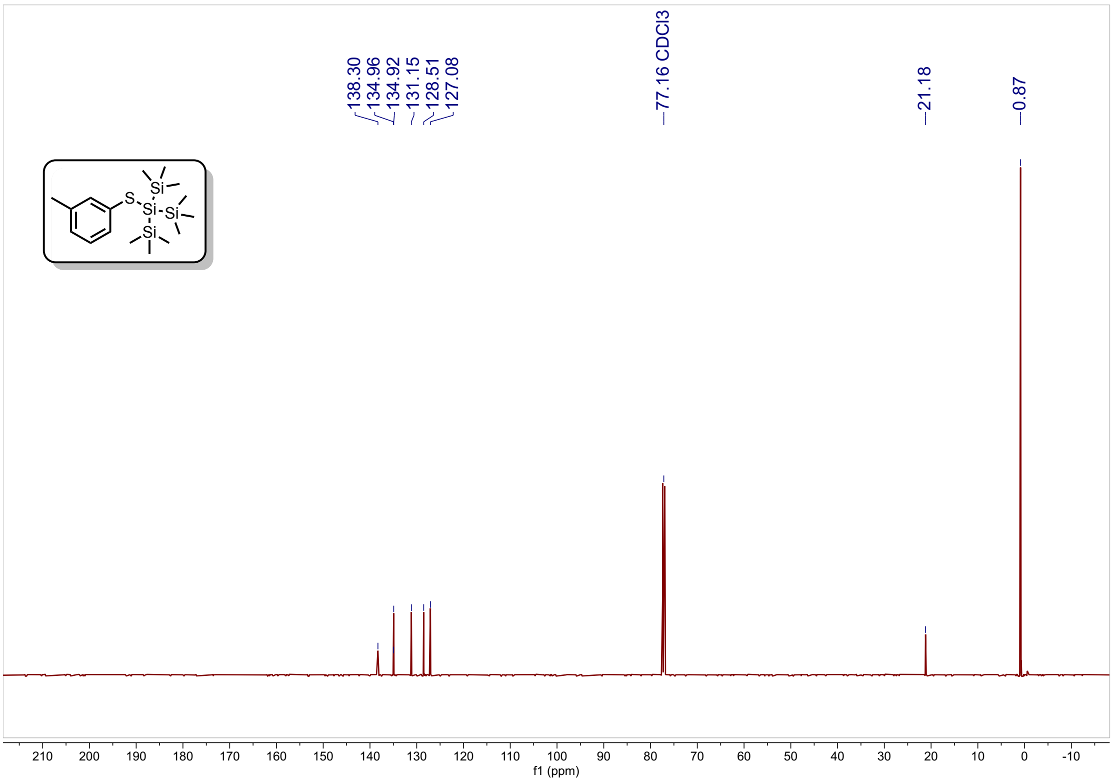

^{29}Si NMR of compound 2j (119 MHz, $CDCl_3$)

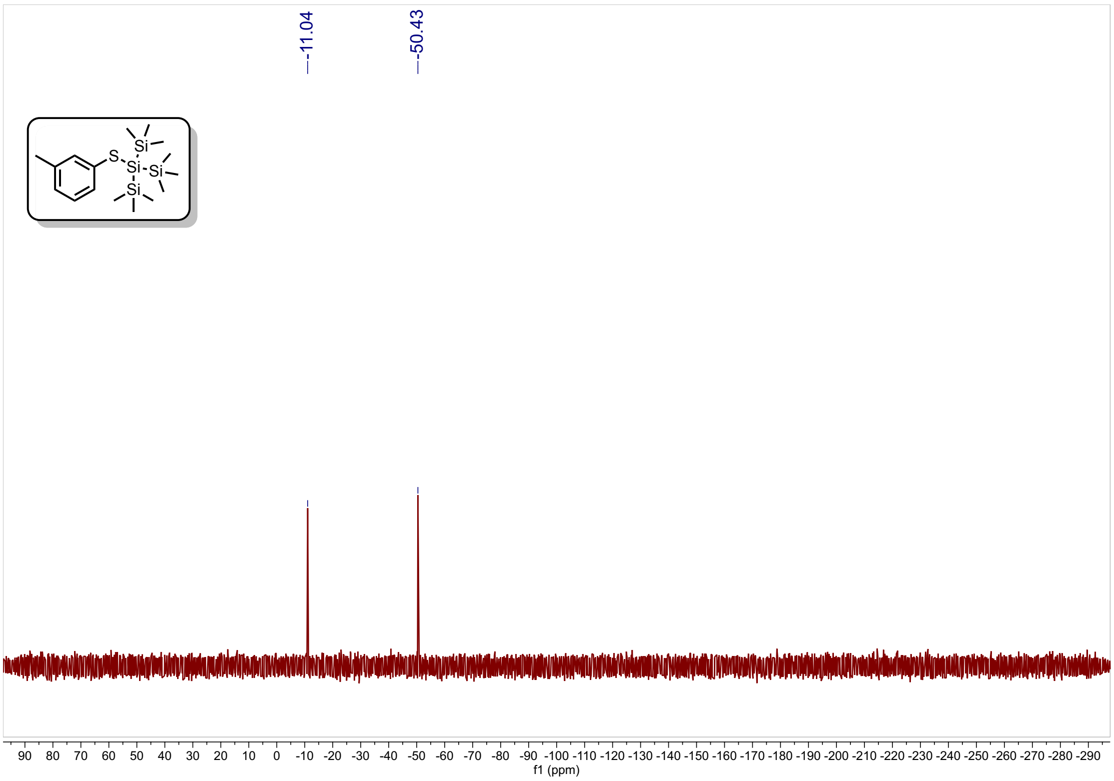

^{1}H NMR of compound 2k (600 MHz, $CDCl_3$)

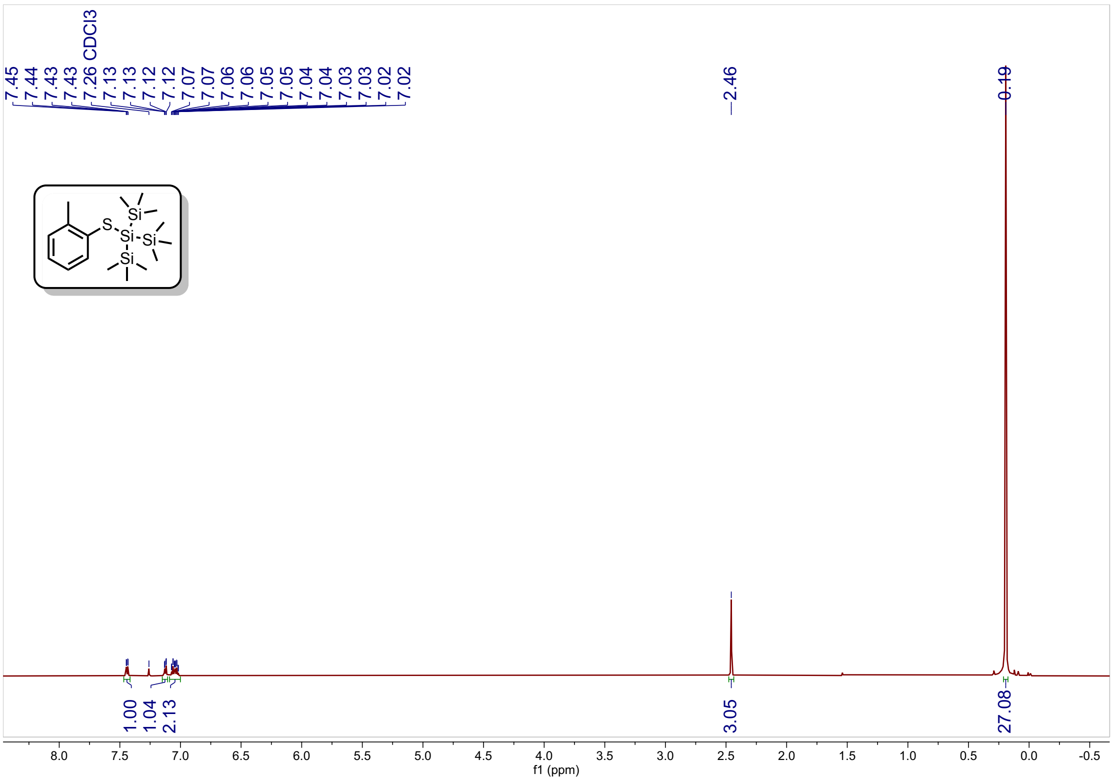

^{13}C NMR of compound 2k (150 MHz, $CDCl_3$)

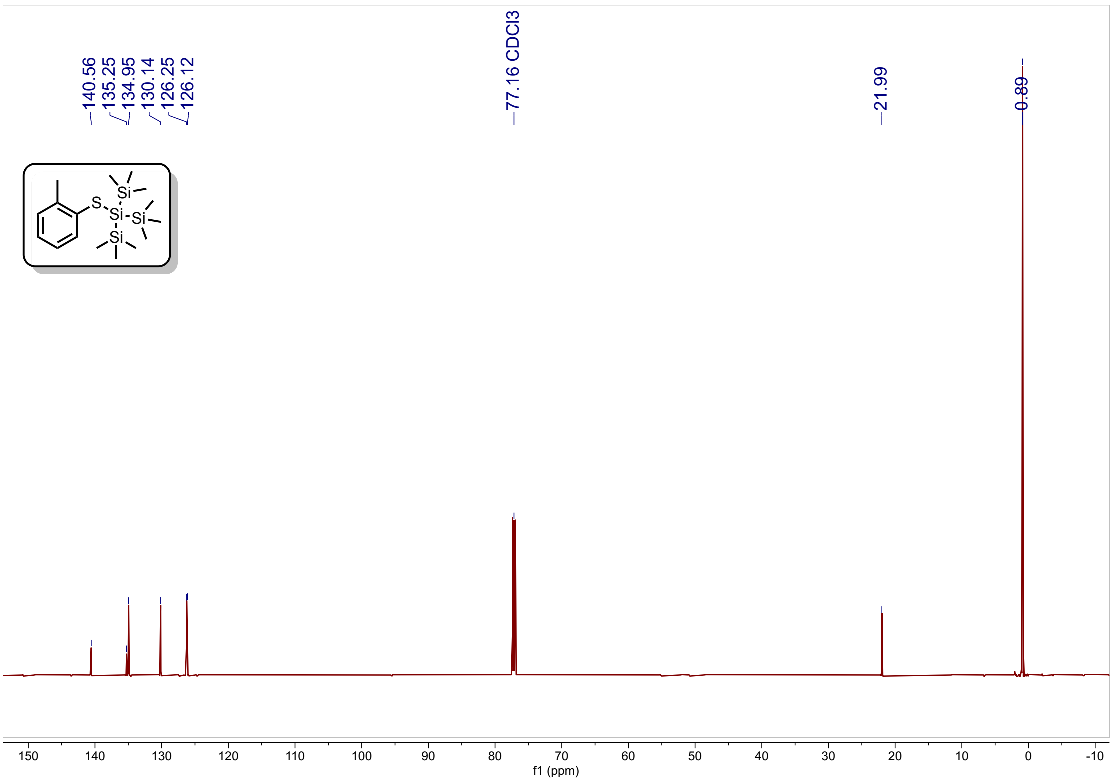

^{29}Si NMR of compound 2k (119 MHz, $CDCl_3$)

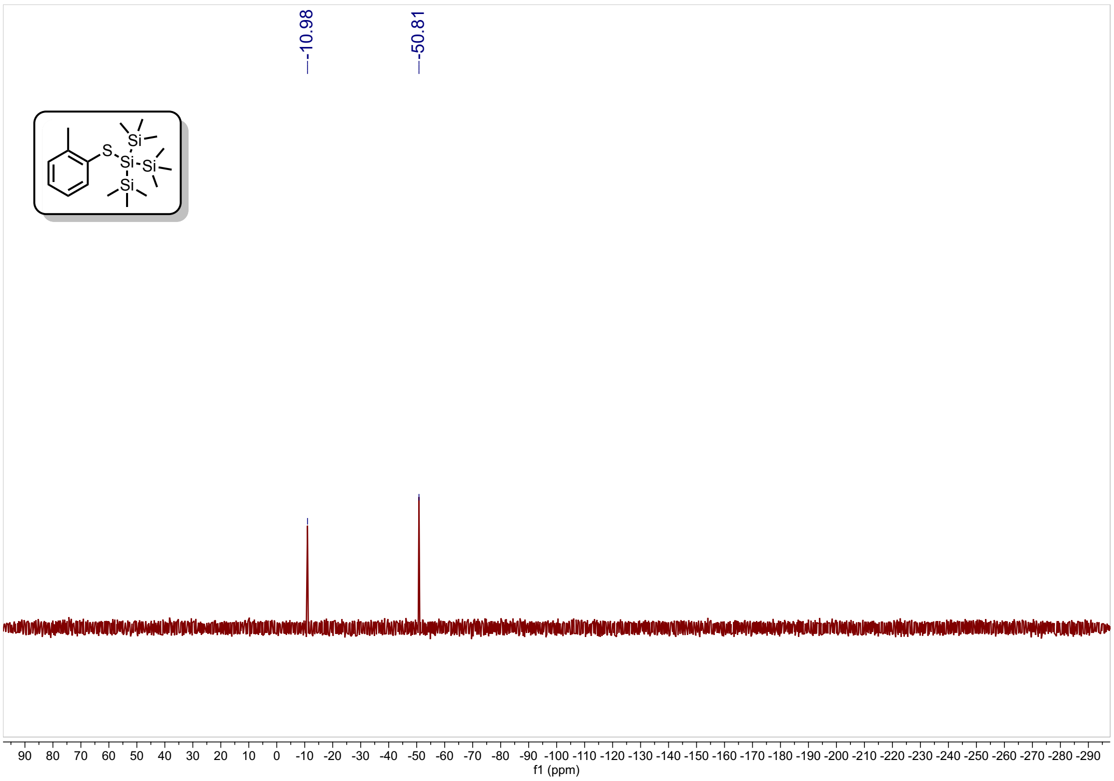

^{1}H NMR of compound 2l (400 MHz, $CDCl_3$)

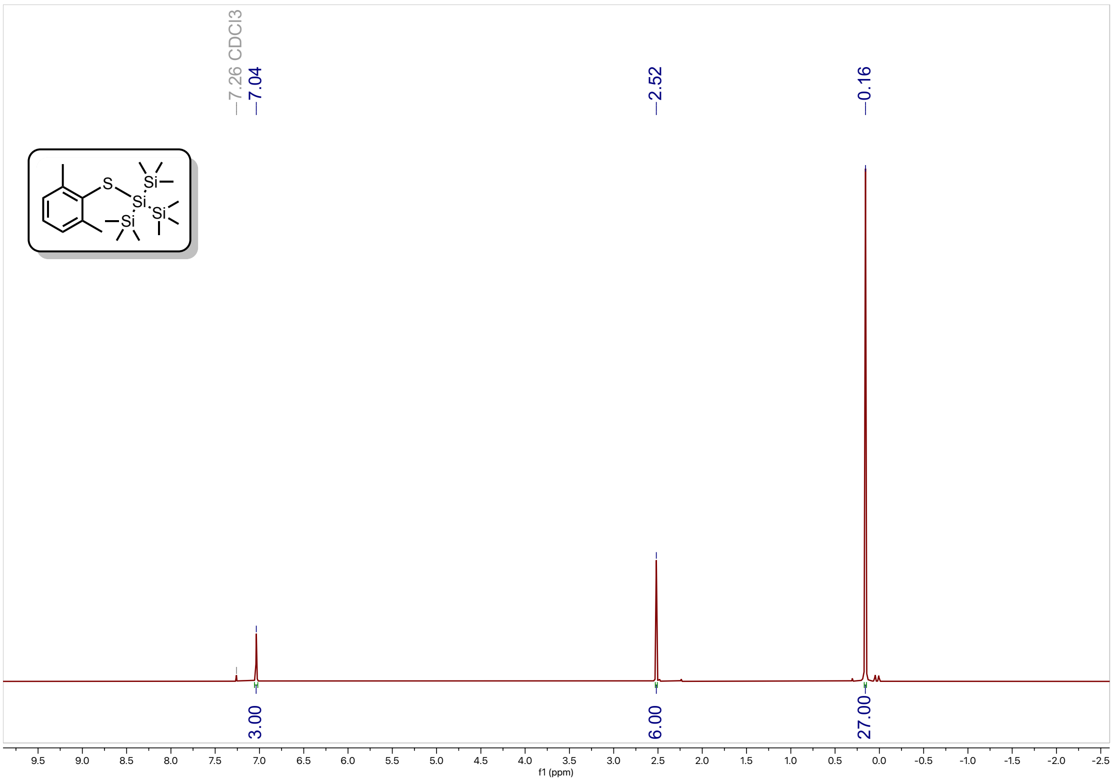

^{13}C NMR of compound 2l (100 MHz, $CDCl_3$)

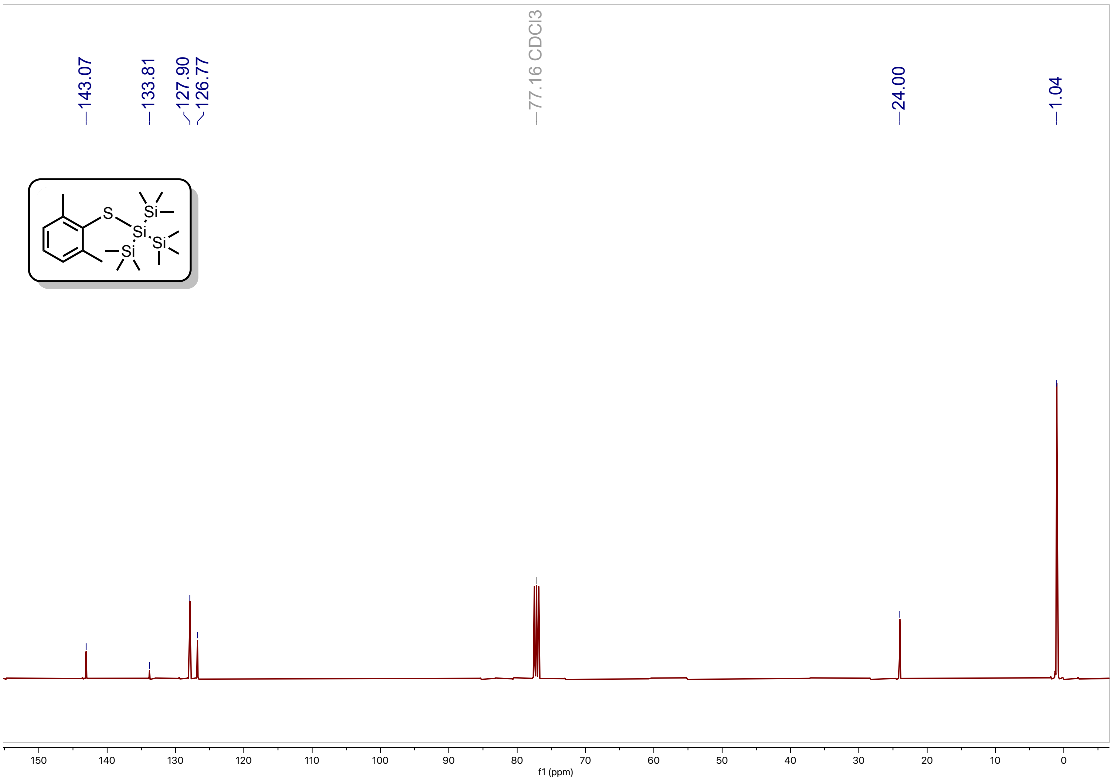

^{29}Si NMR of compound 2l (79 MHz, $CDCl_3$)

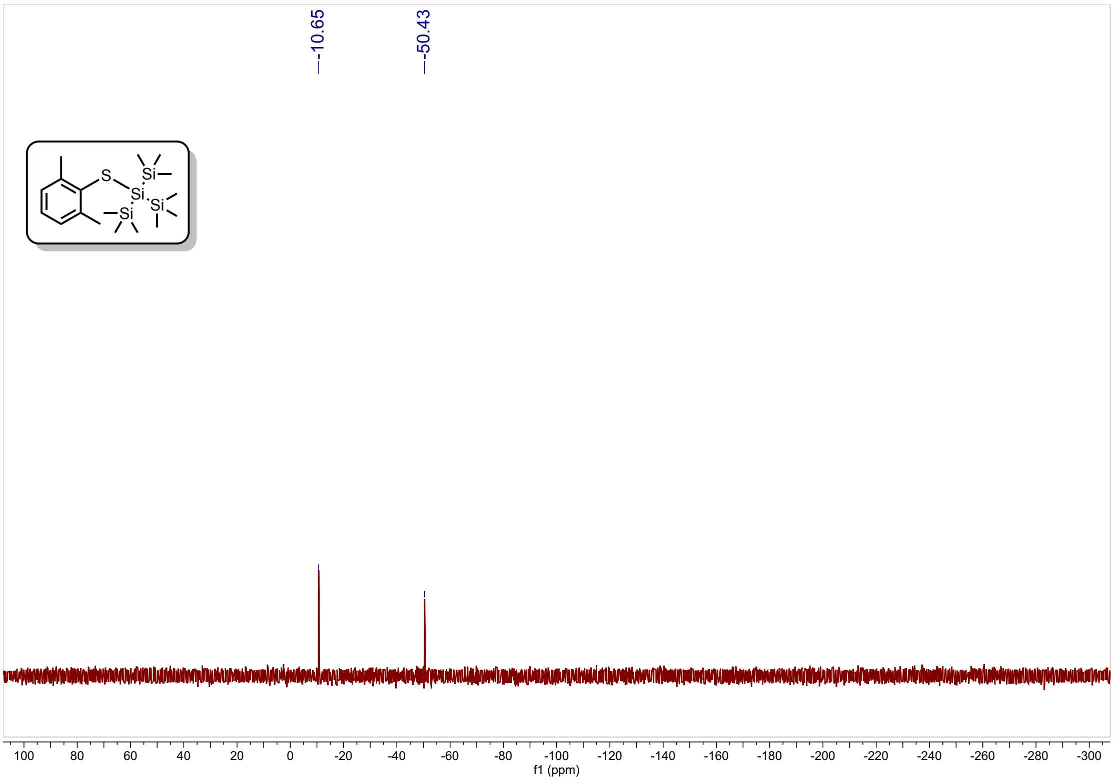

^{1}H NMR of compound 2m (400 MHz, $CDCl_3$)

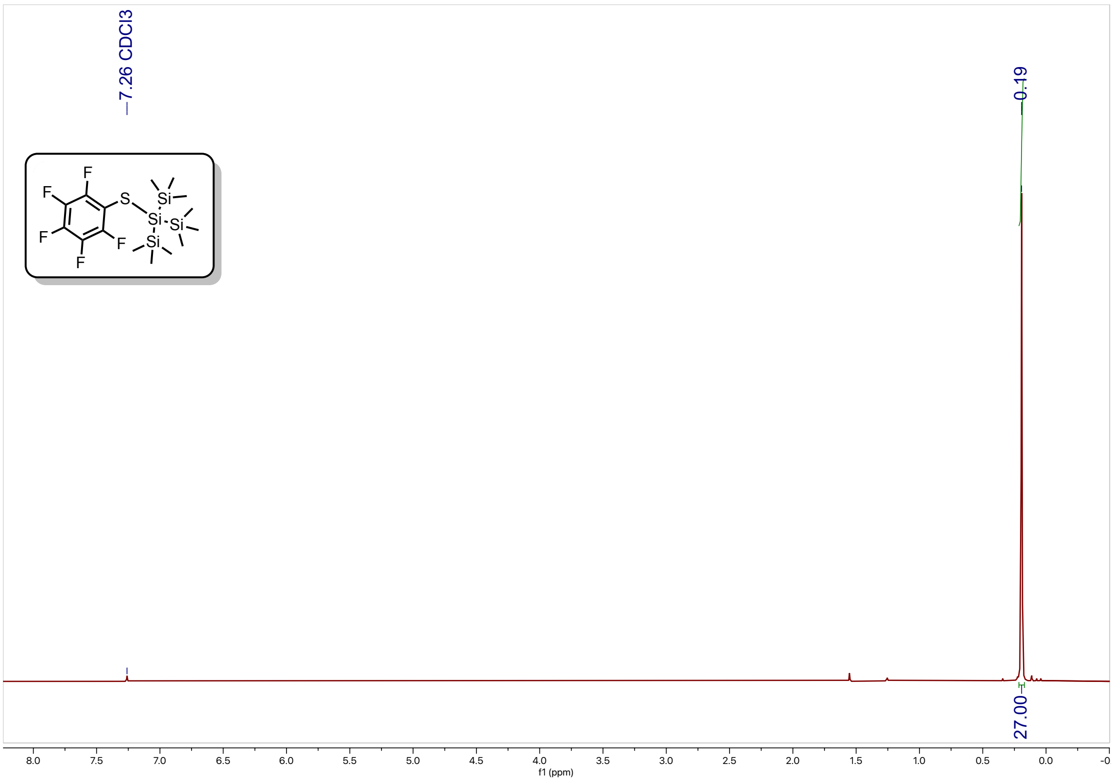

^{13}C NMR of compound 2m (100 MHz, $CDCl_3$)

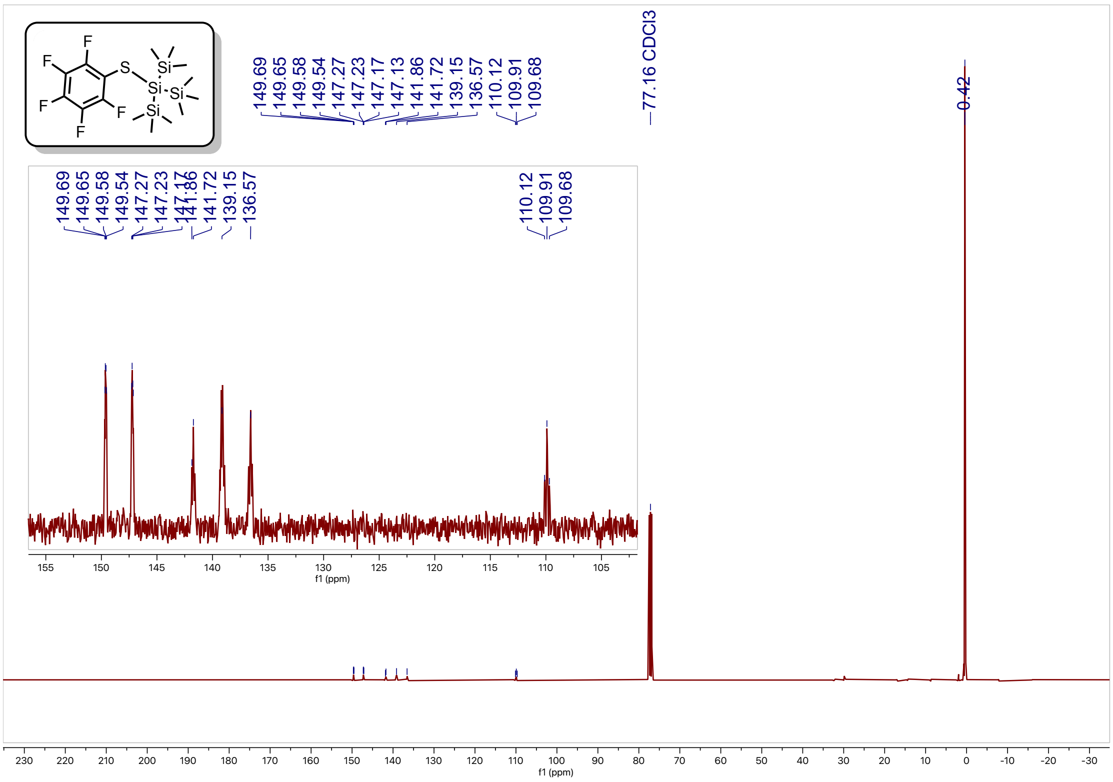

^{19}F NMR of compound 2m (376 MHz, $CDCl_3$)

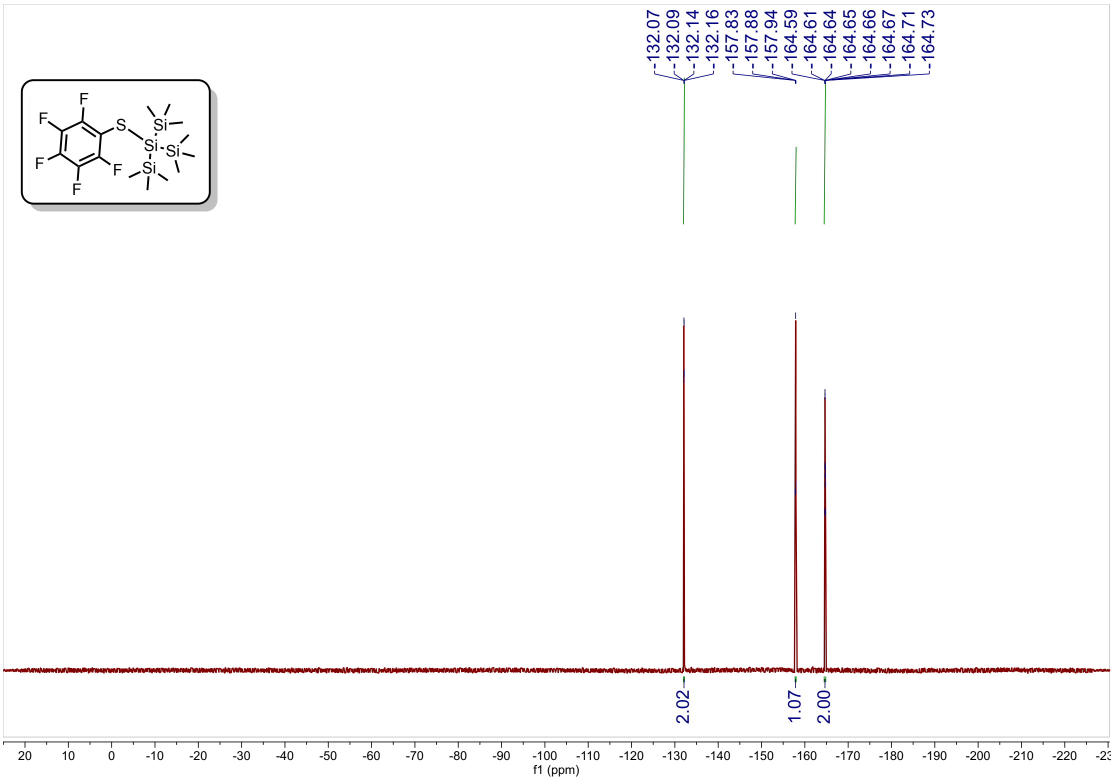

^{29}Si NMR of compound 2m (79 MHz, $CDCl_3$)

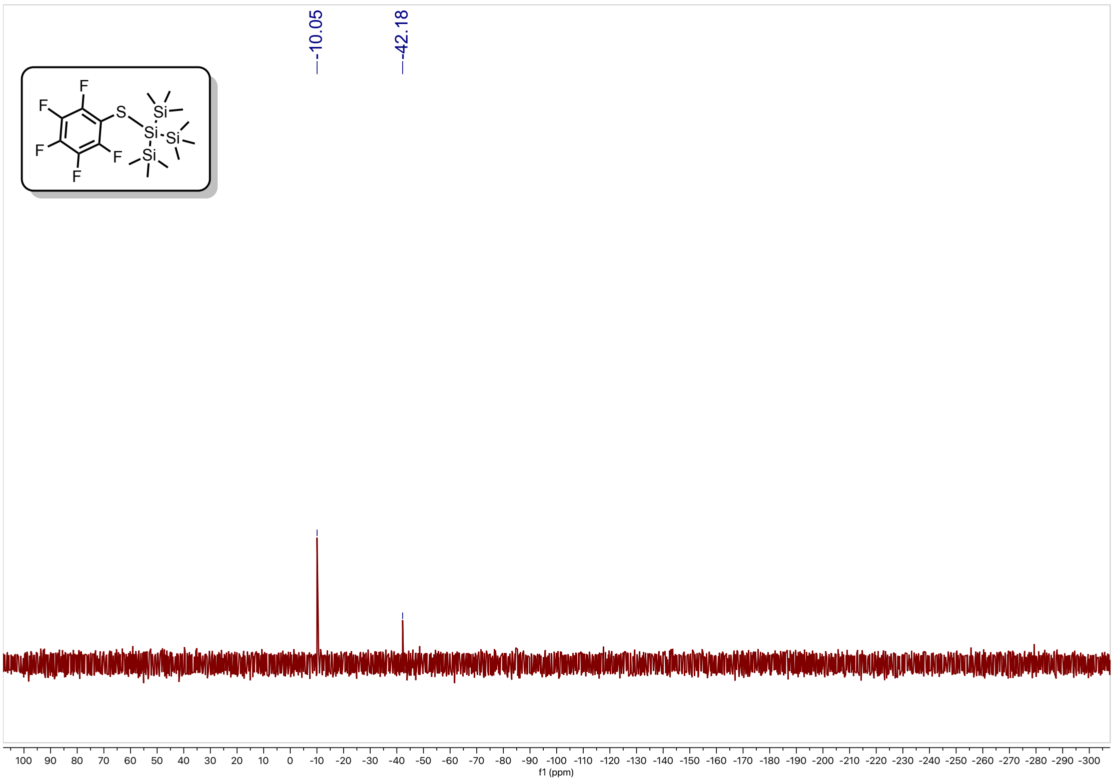

^{1}H NMR of compound 2n (400 MHz, $CDCl_3$)

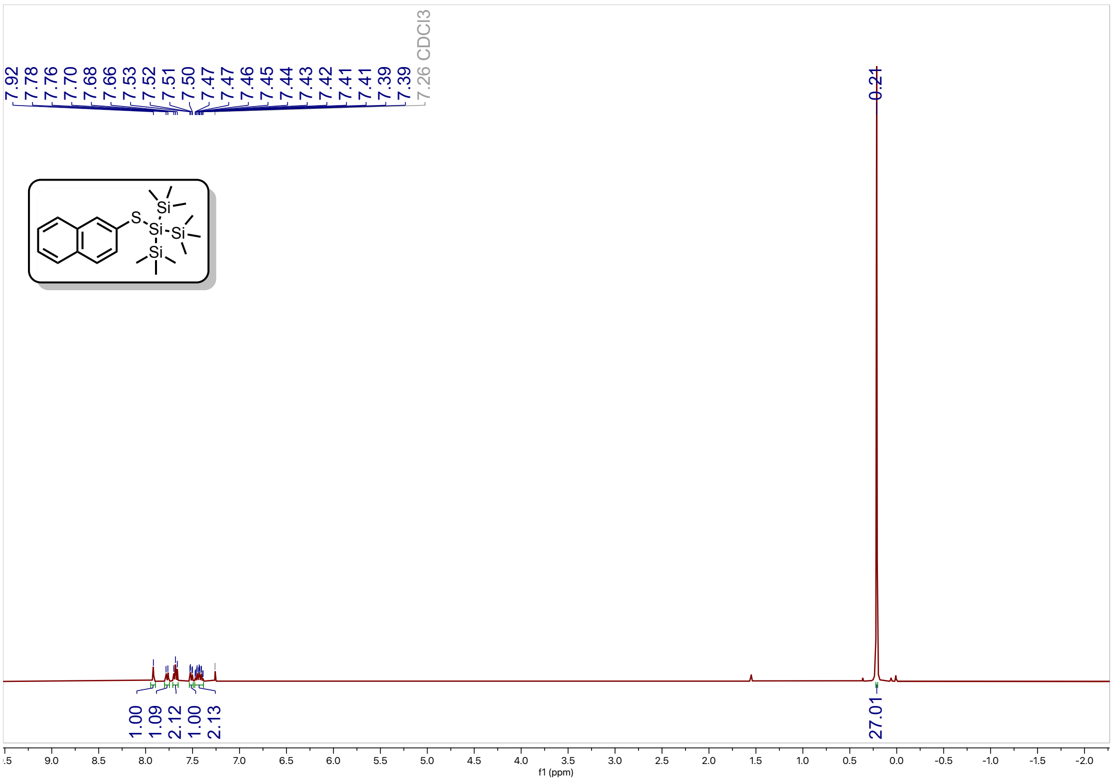

^{13}C NMR of compound 2n (100 MHz, $CDCl_3$)

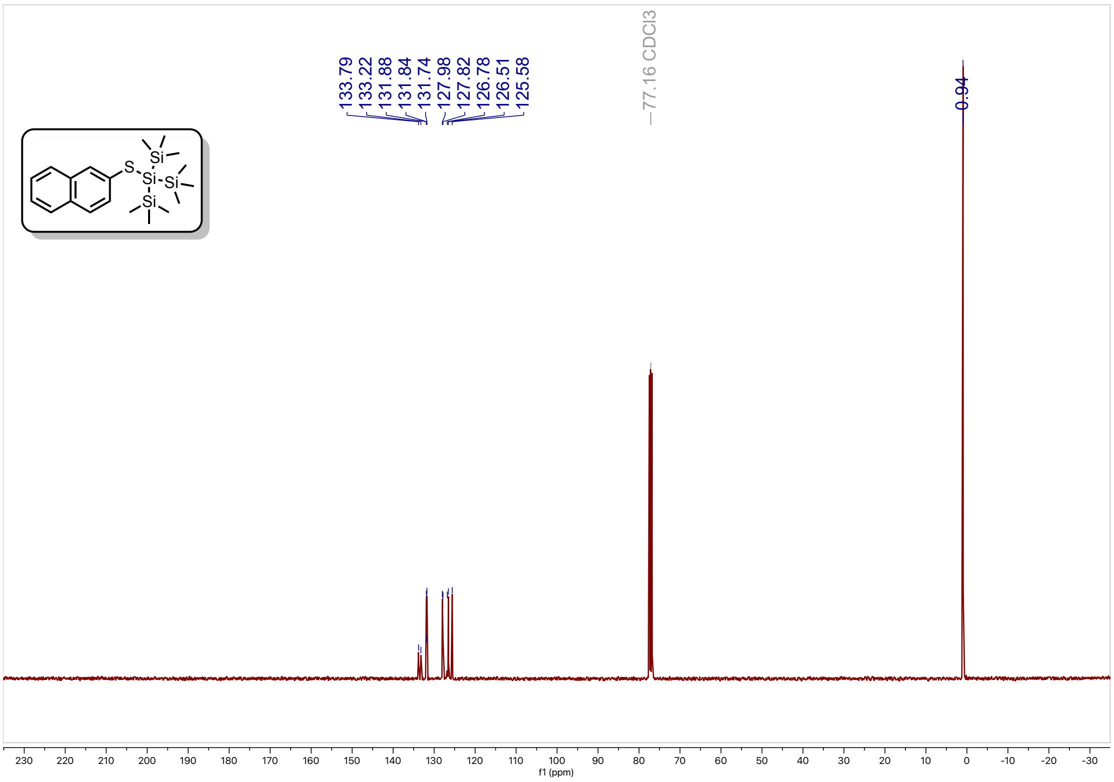

^{29}Si NMR of compound 2n (79 MHz, $CDCl_3$)

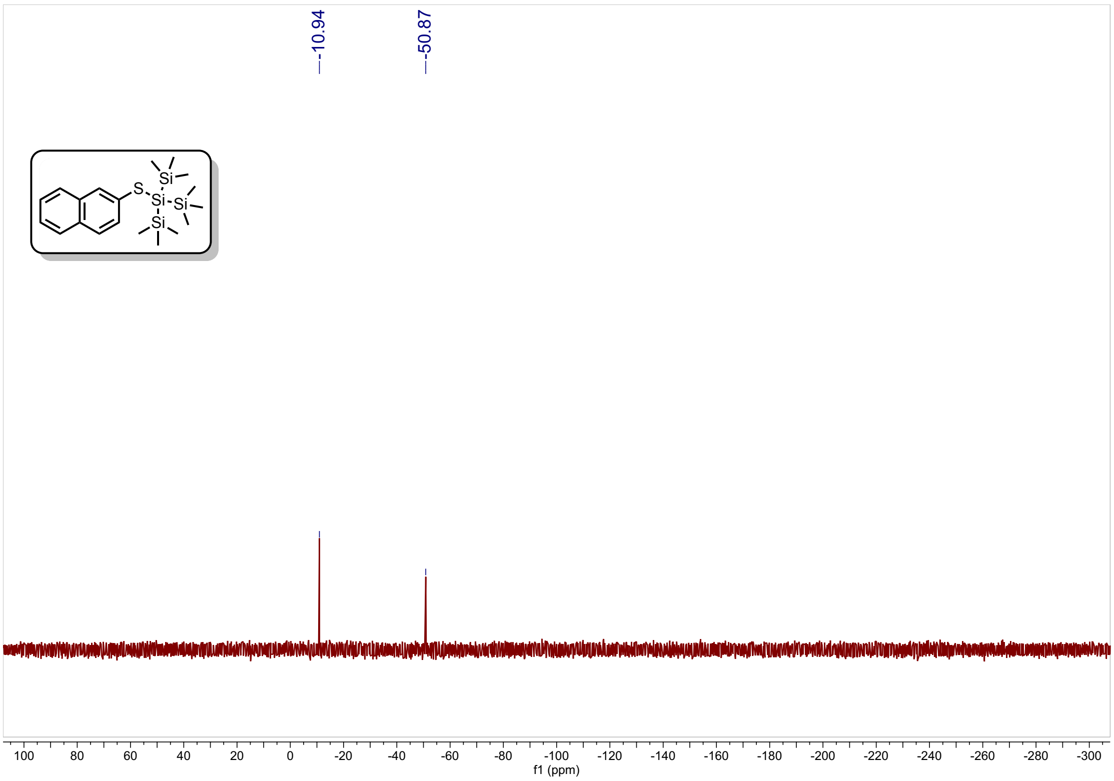

^{1}H NMR of compound 2o (400 MHz, $CDCl_3$)

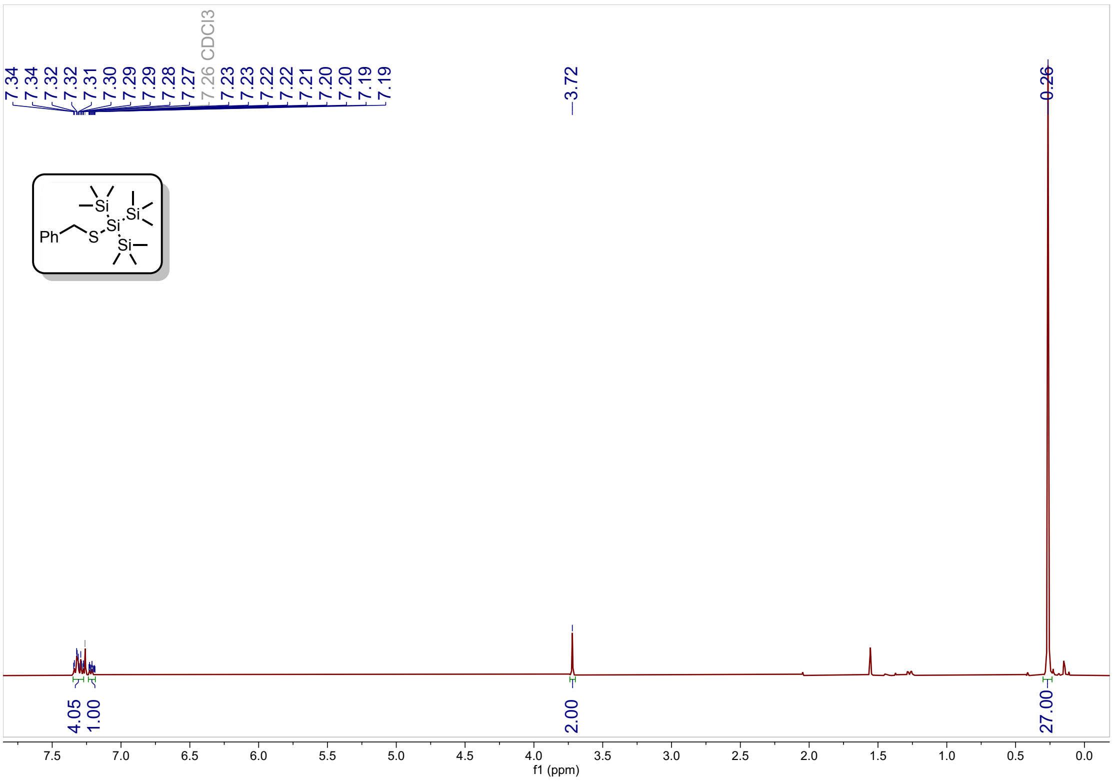

^{13}C NMR of compound 2o (100 MHz, $CDCl_3$)

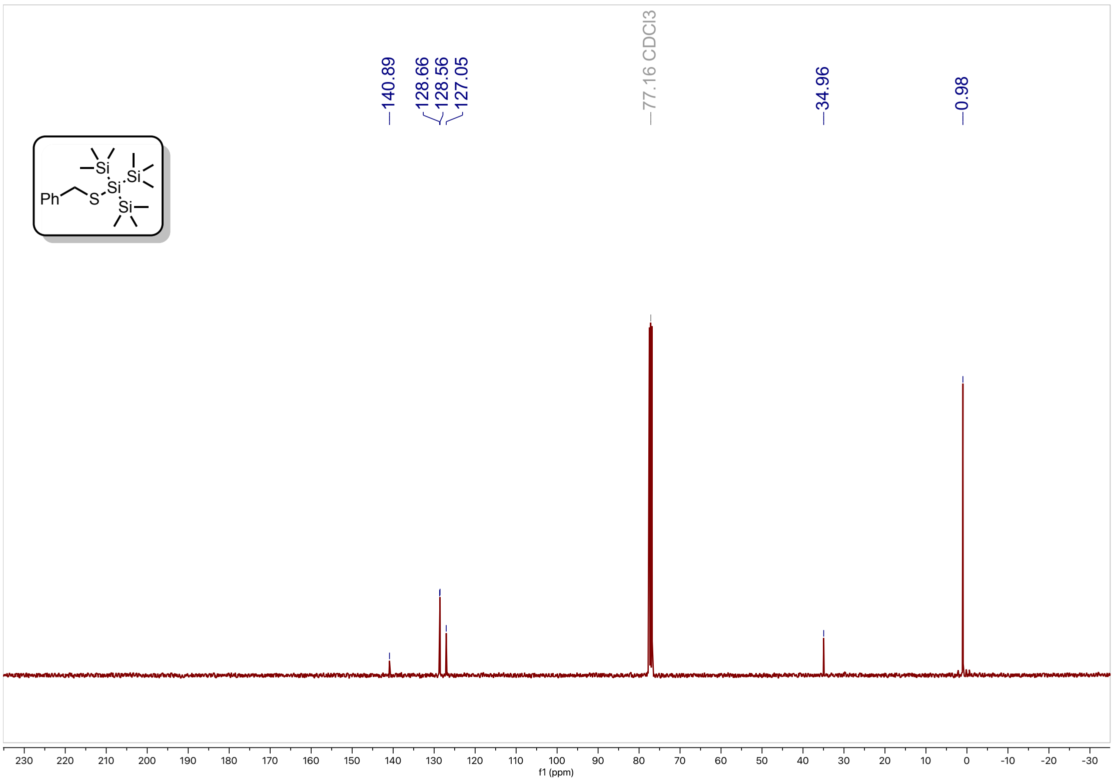

^{29}Si NMR of compound 2o (79 MHz, $CDCl_3$)

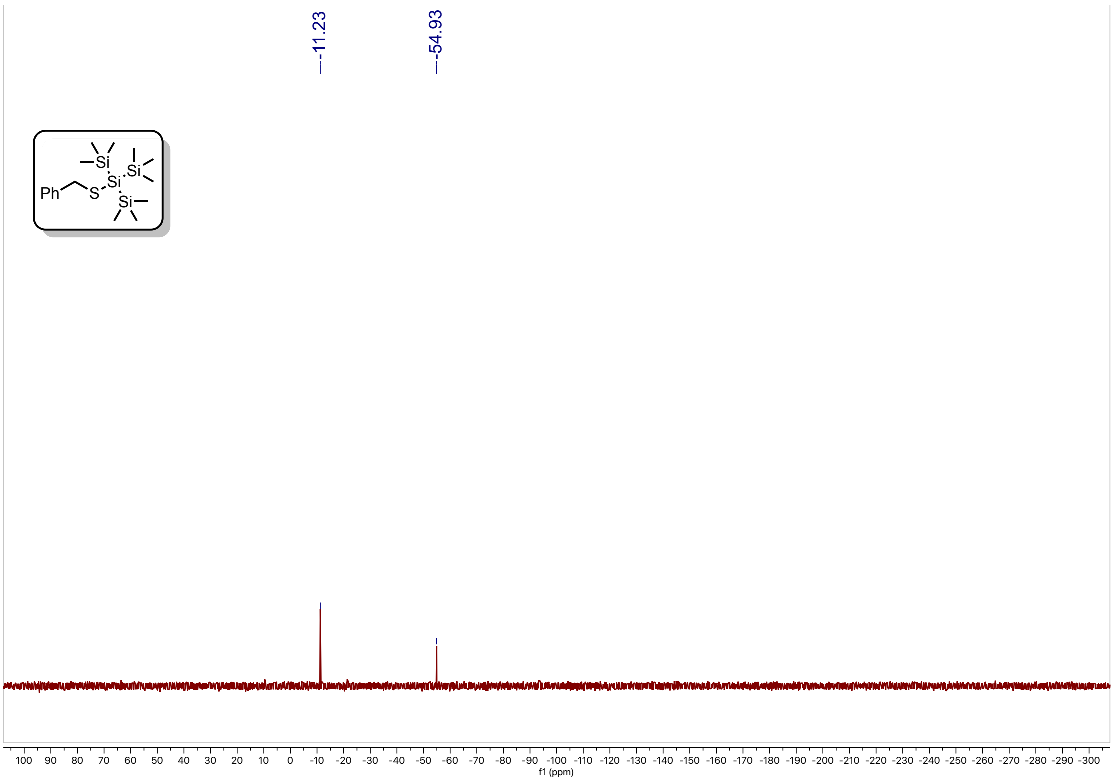

^{1}H NMR of compound 2p (400 MHz, $CDCl_3$)

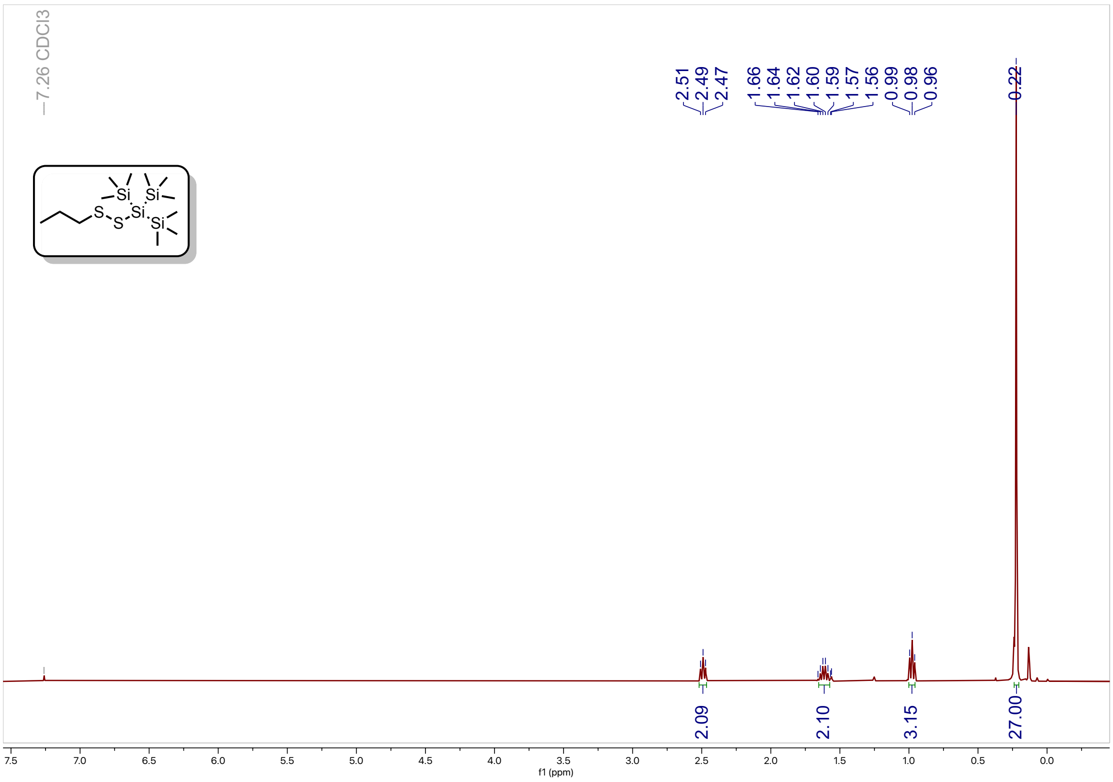

^{13}C NMR of compound 2p (100 MHz, $CDCl_3$)

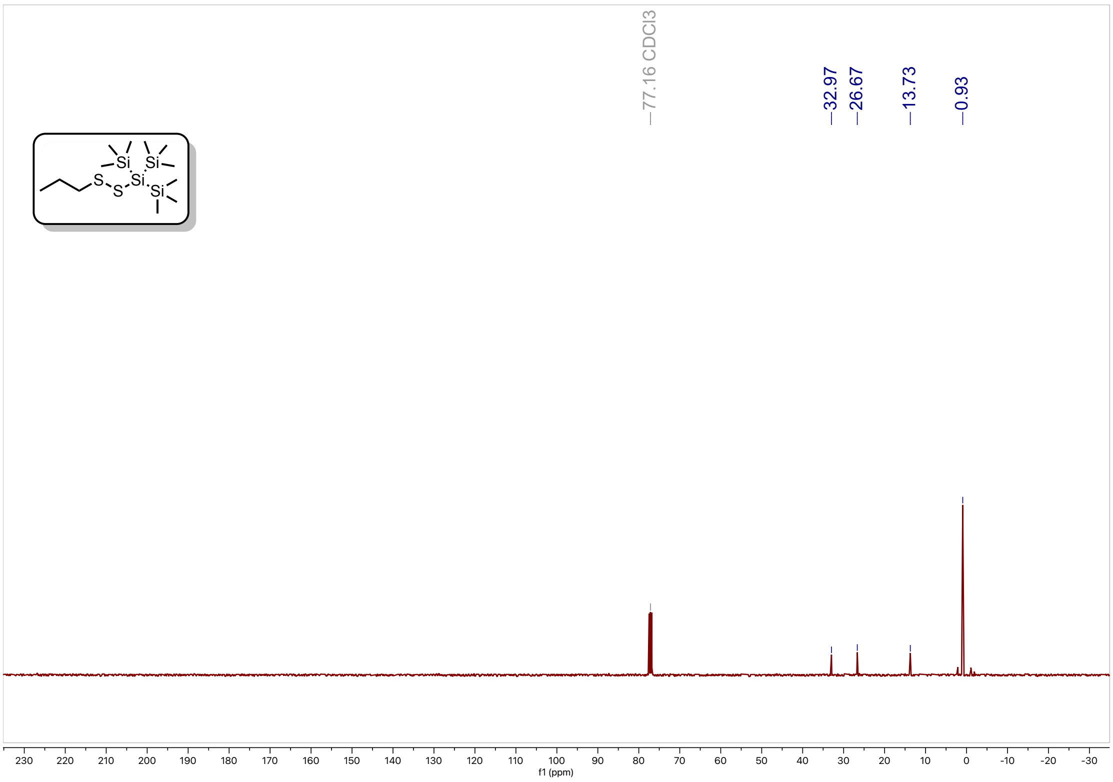

^{29}Si NMR of compound 2p (79 MHz, $CDCl_3$)

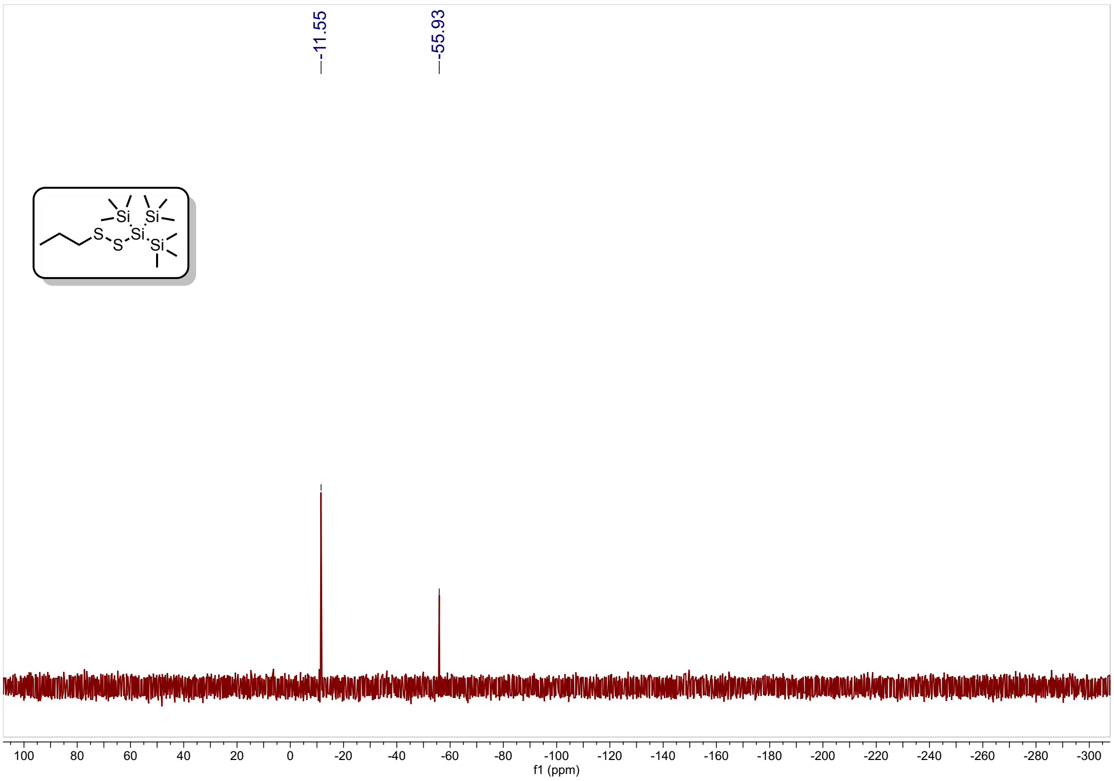

^{1}H NMR of compound 2q (600 MHz, $CDCl_3$)

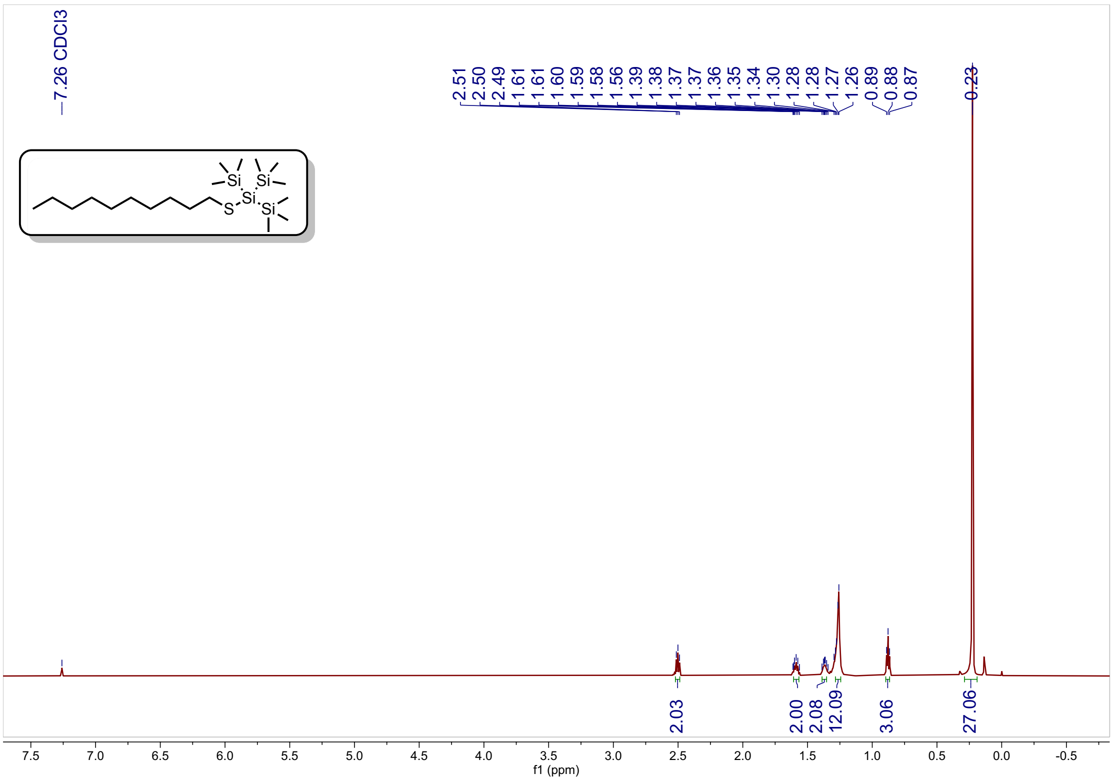

^{13}C NMR of compound 2q (100 MHz, $CDCl_3$)

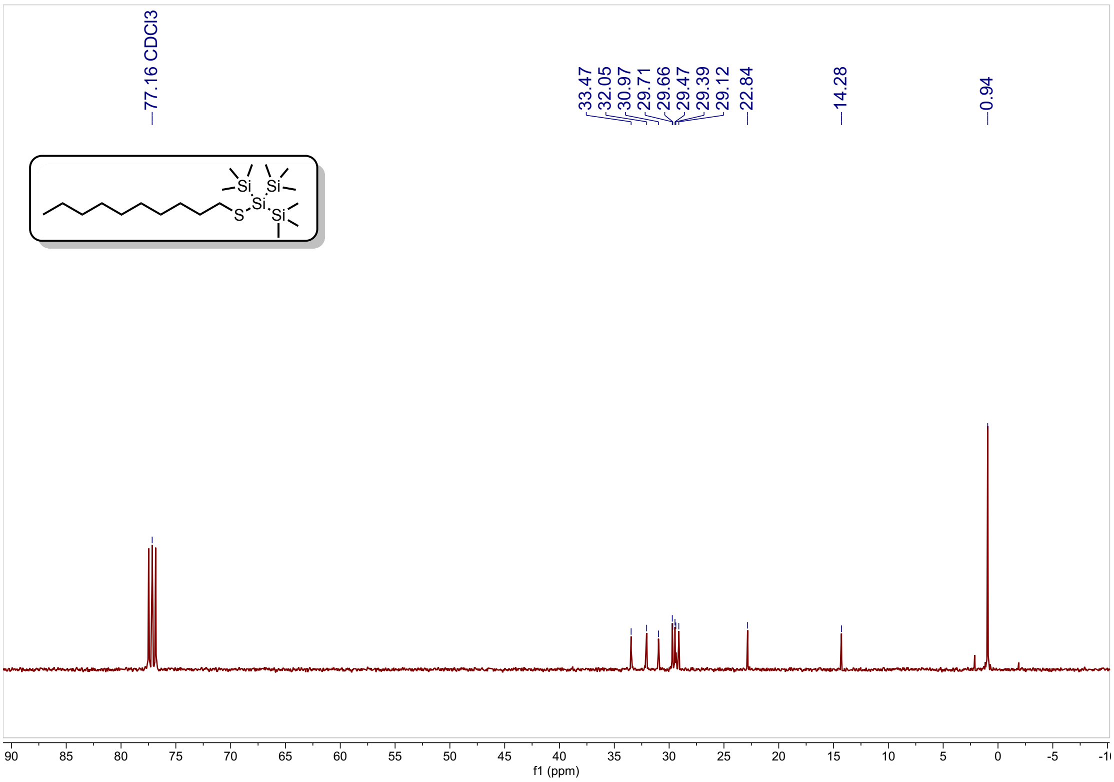

^{29}Si NMR of compound 2q (79 MHz, $CDCl_3$)

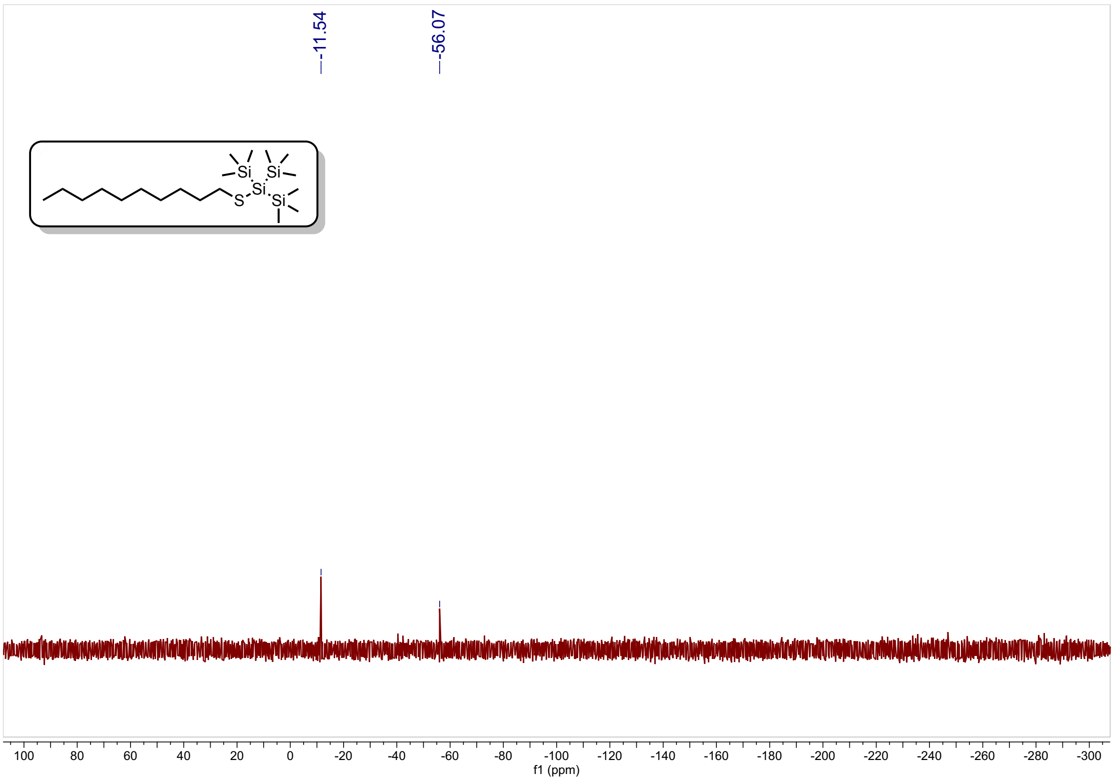

^{1}H NMR of compound 2r (400 MHz, $CDCl_3$)

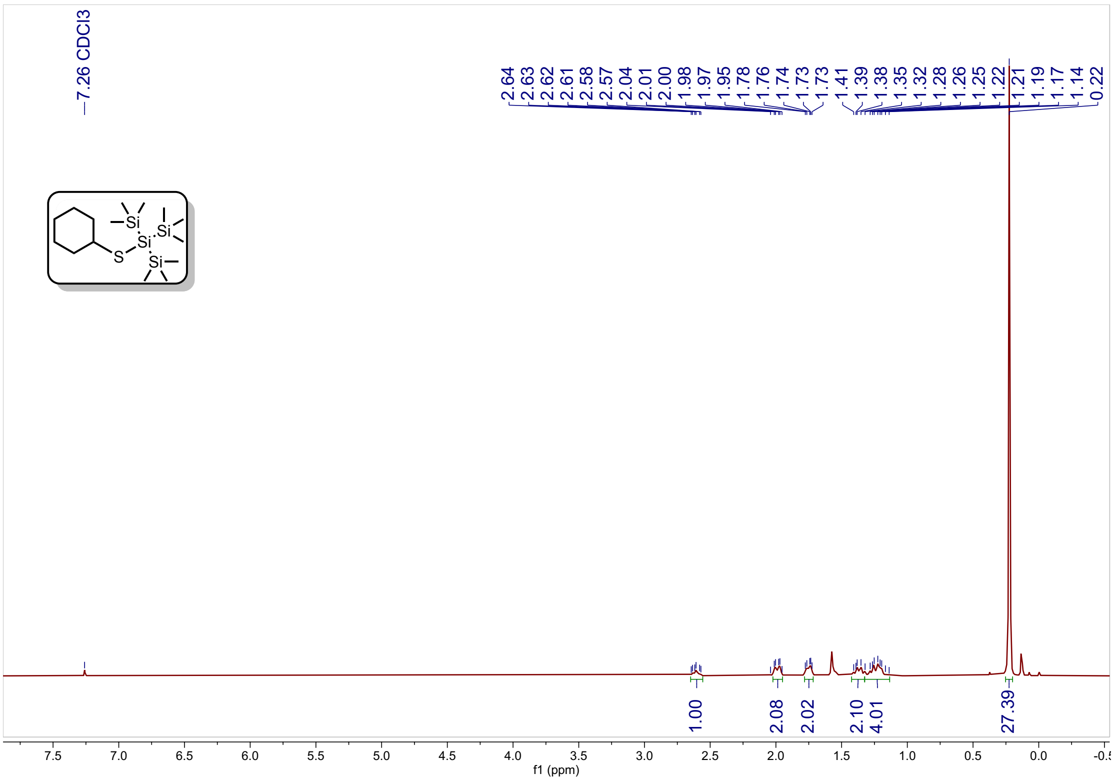

^{13}C NMR of compound 2r (100 MHz, $CDCl_3$)

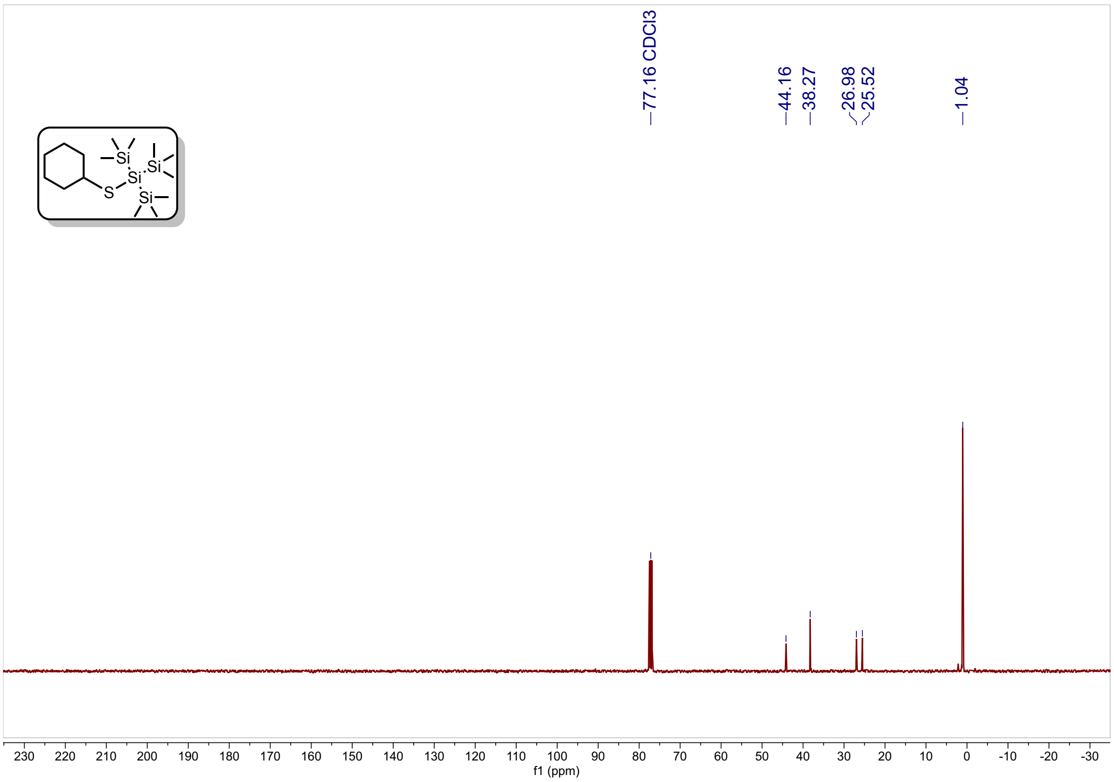

^{29}Si NMR of compound 2r (79 MHz, $CDCl_3$)

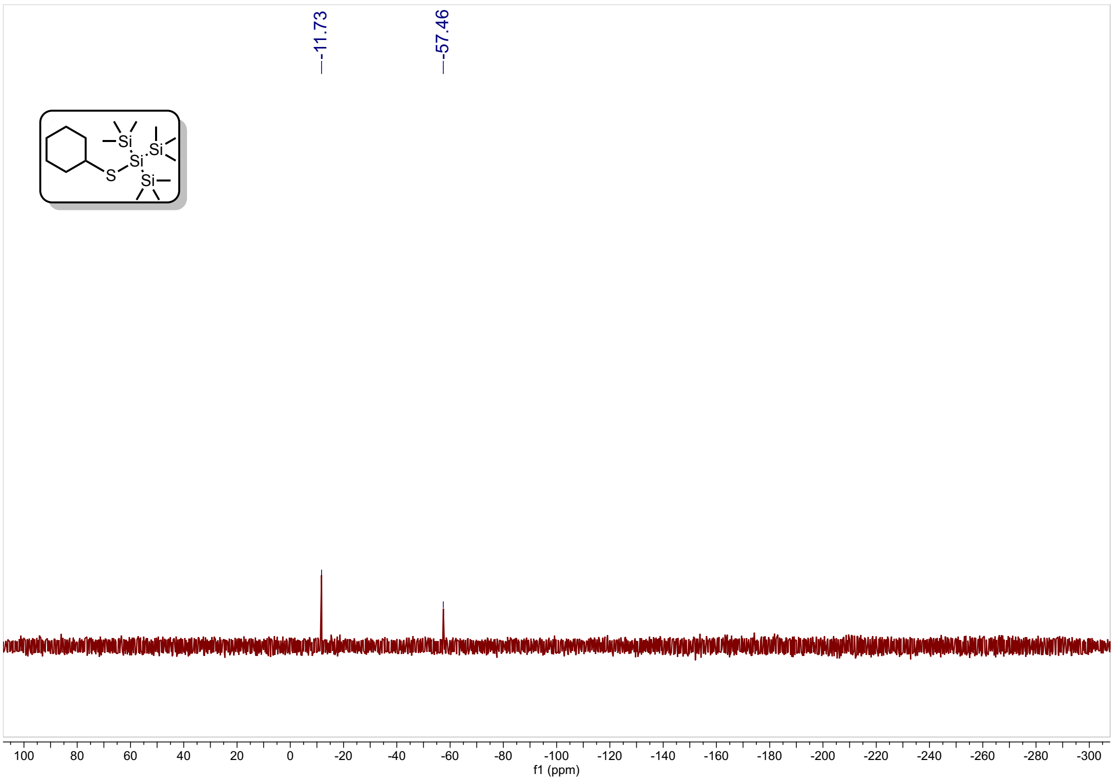

^{1}H NMR of compound 2s (400 MHz, $CDCl_3$)

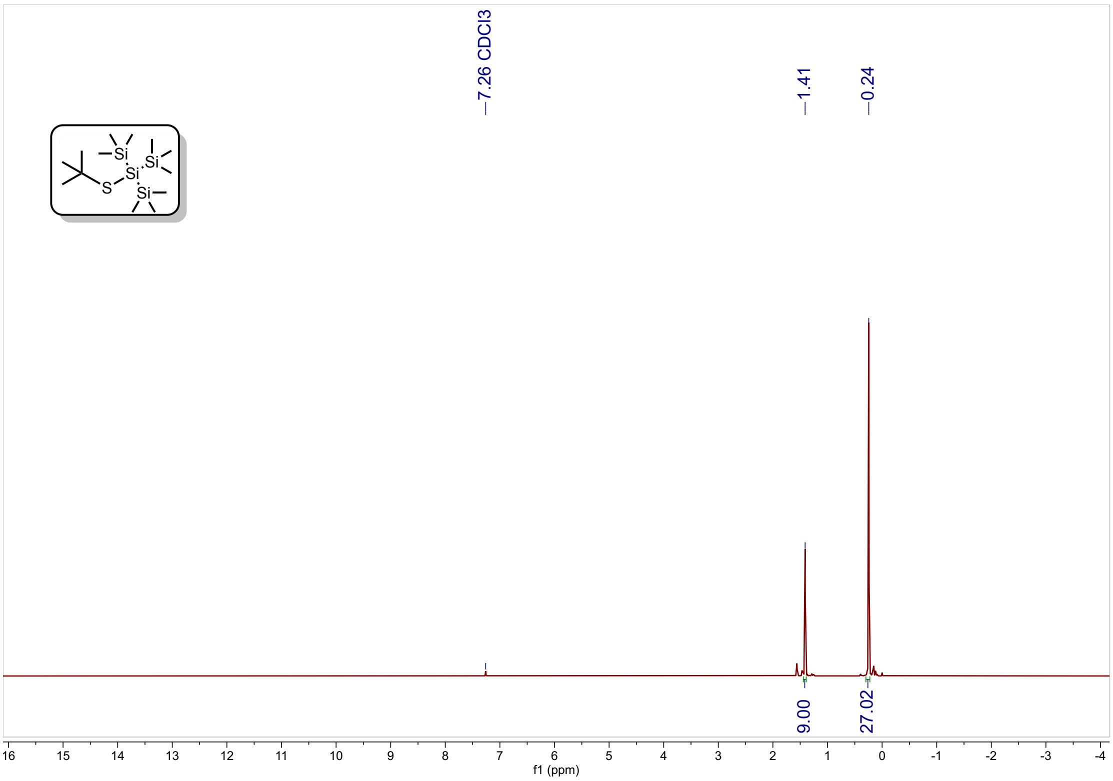

^{13}C NMR of compound 2s (100 MHz, $CDCl_3$)

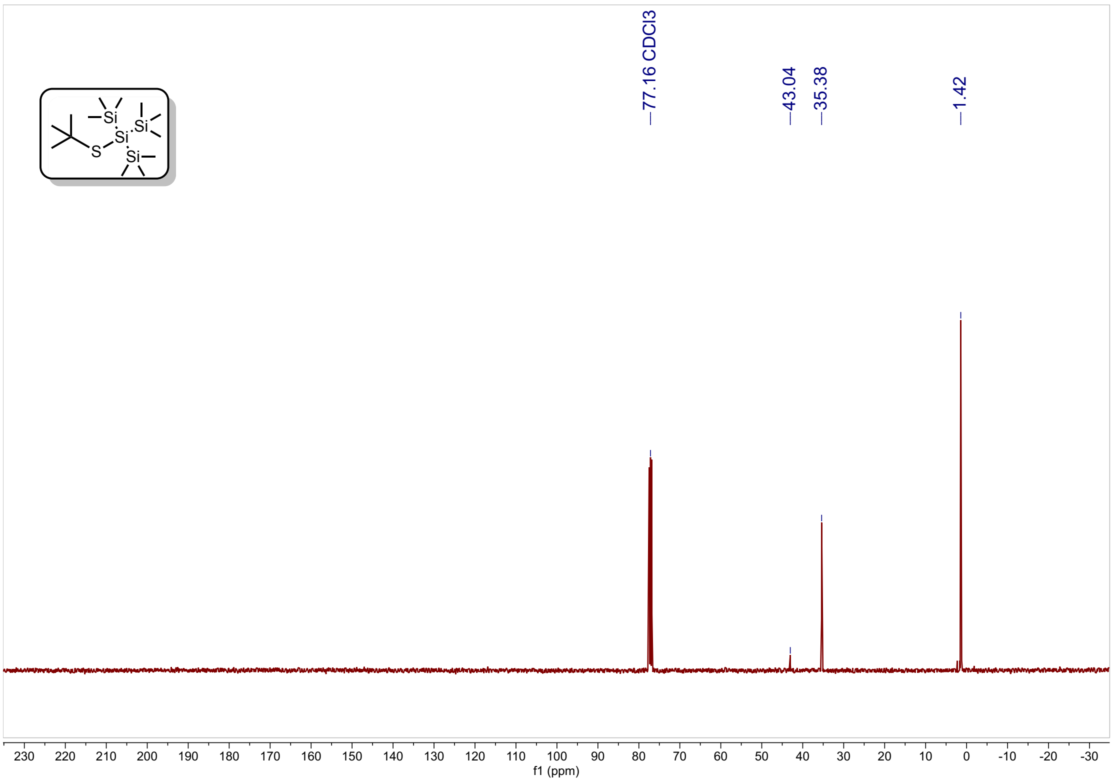

^{29}Si NMR of compound 2s (79 MHz, $CDCl_3$)

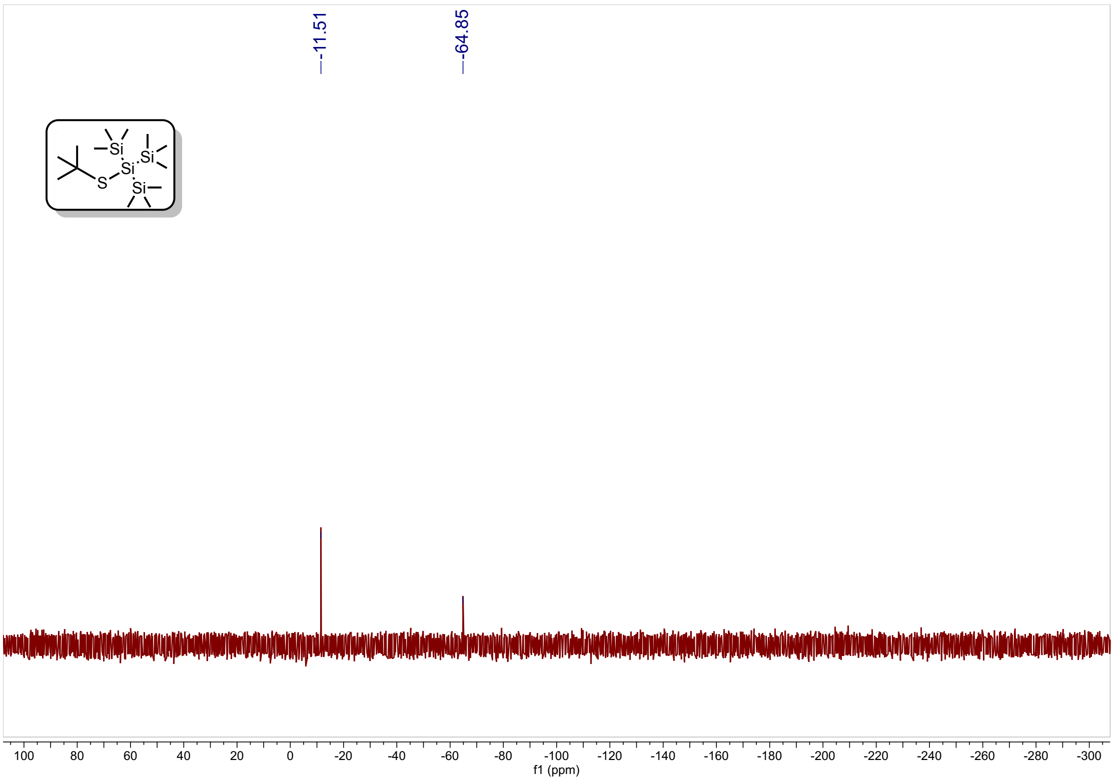

^{1}H NMR of compound 2t (600 MHz, $CDCl_3$)

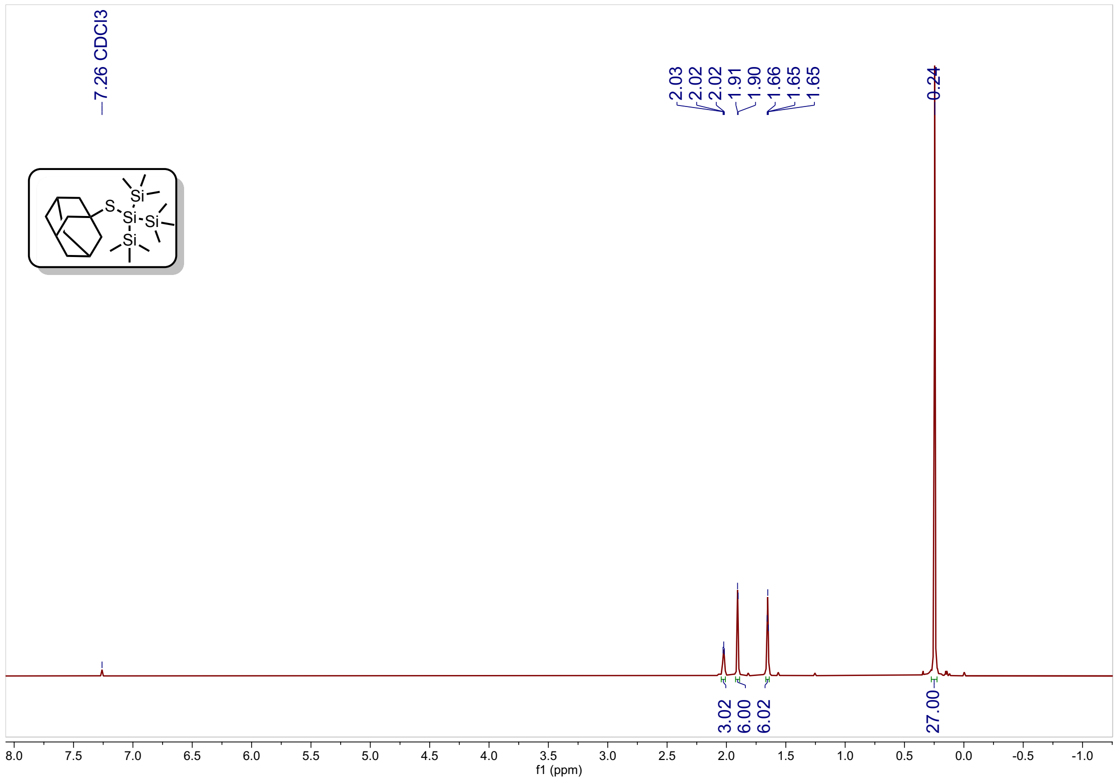

^{13}C NMR of compound 2t (100 MHz, $CDCl_3$)

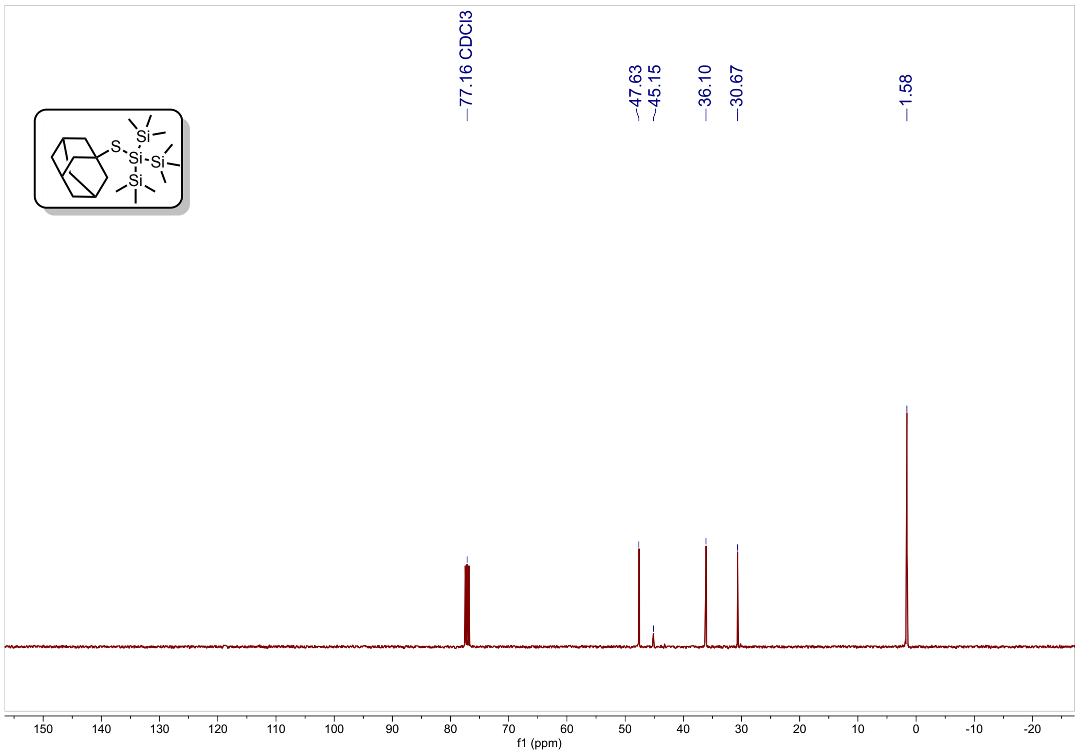

^{29}Si NMR of compound 2t (79 MHz, $CDCl_3$)

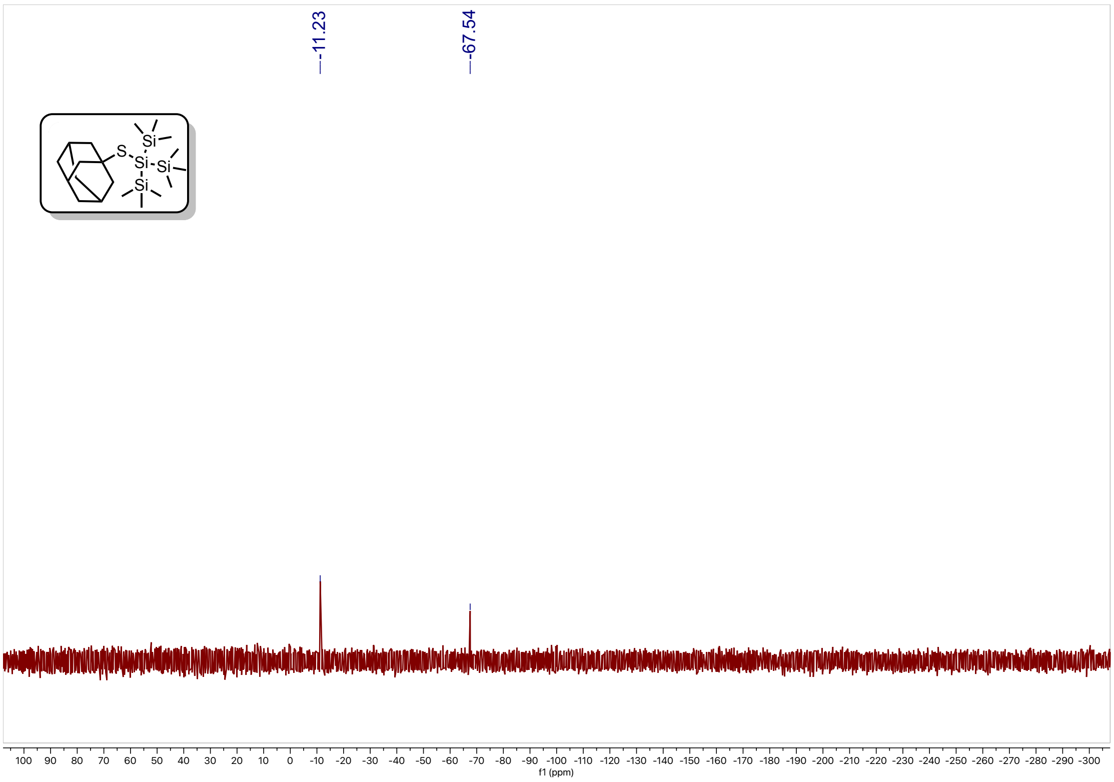

^{1}H NMR of compound 2u (400 MHz, $CDCl_3$)

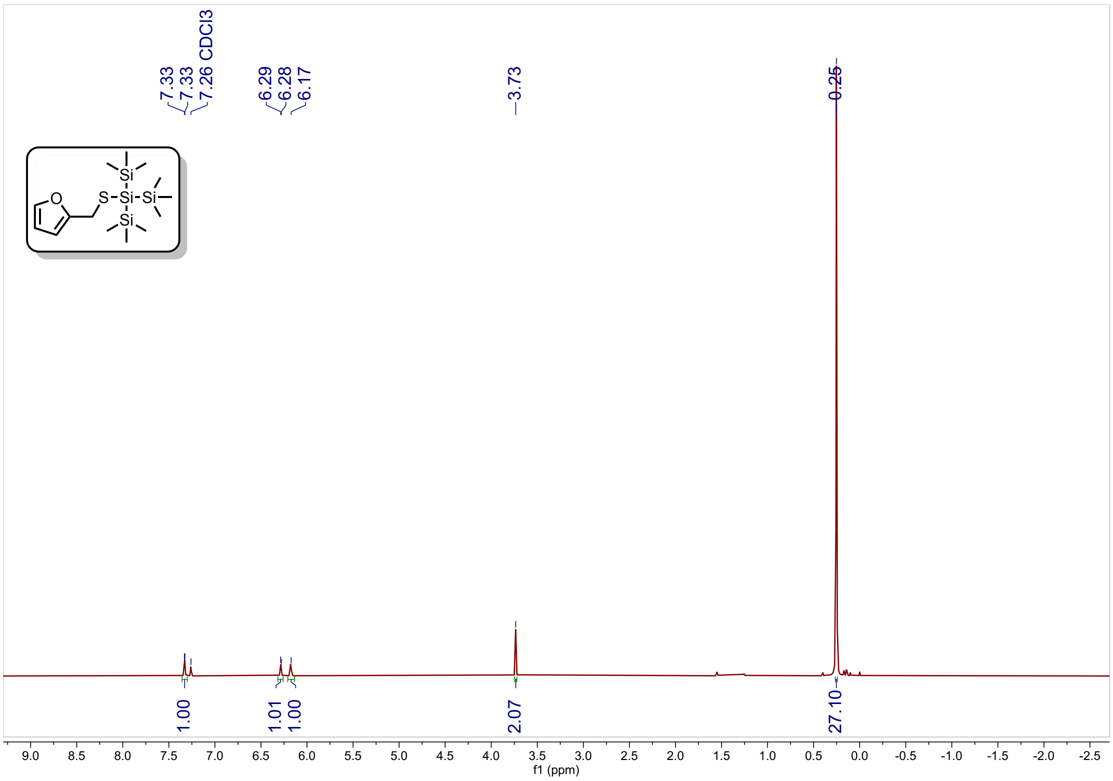

^{13}C NMR of compound 2u (100 MHz, $CDCl_3$)

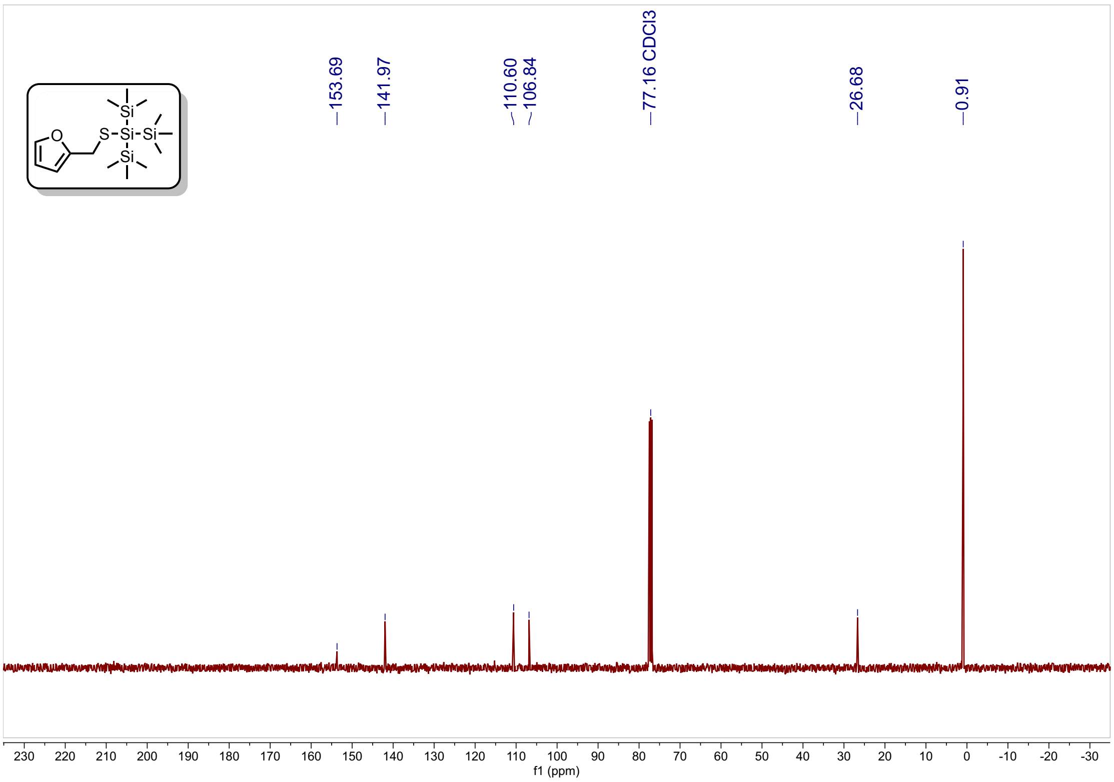

^{29}Si NMR of compound 2u (79 MHz, $CDCl_3$)

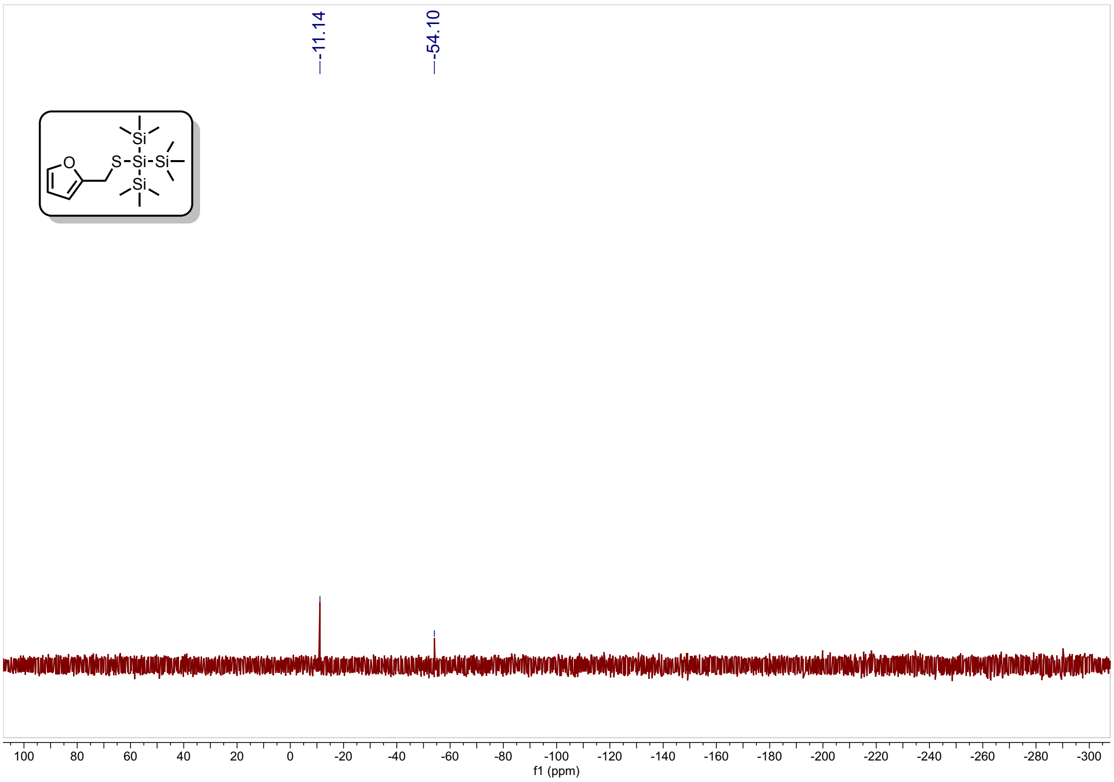

^{1}H NMR of compound 2v (400 MHz, $CDCl_3$)

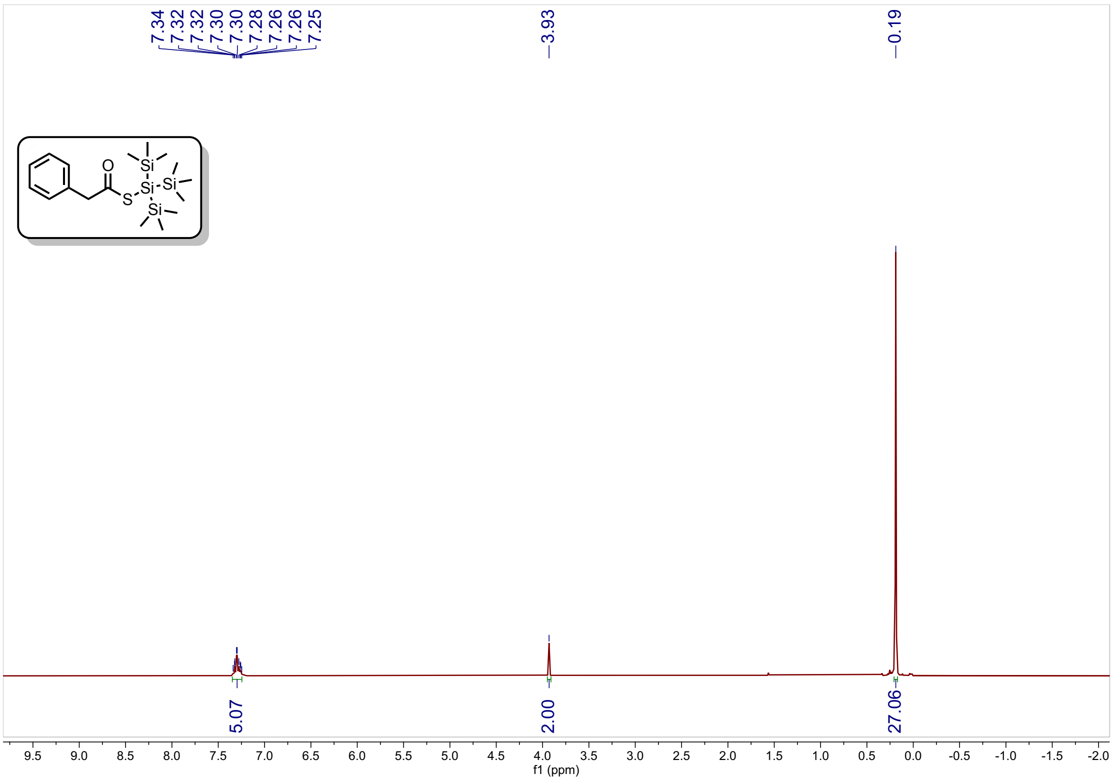

^{13}C NMR of compound 2v (100 MHz, $CDCl_3$)

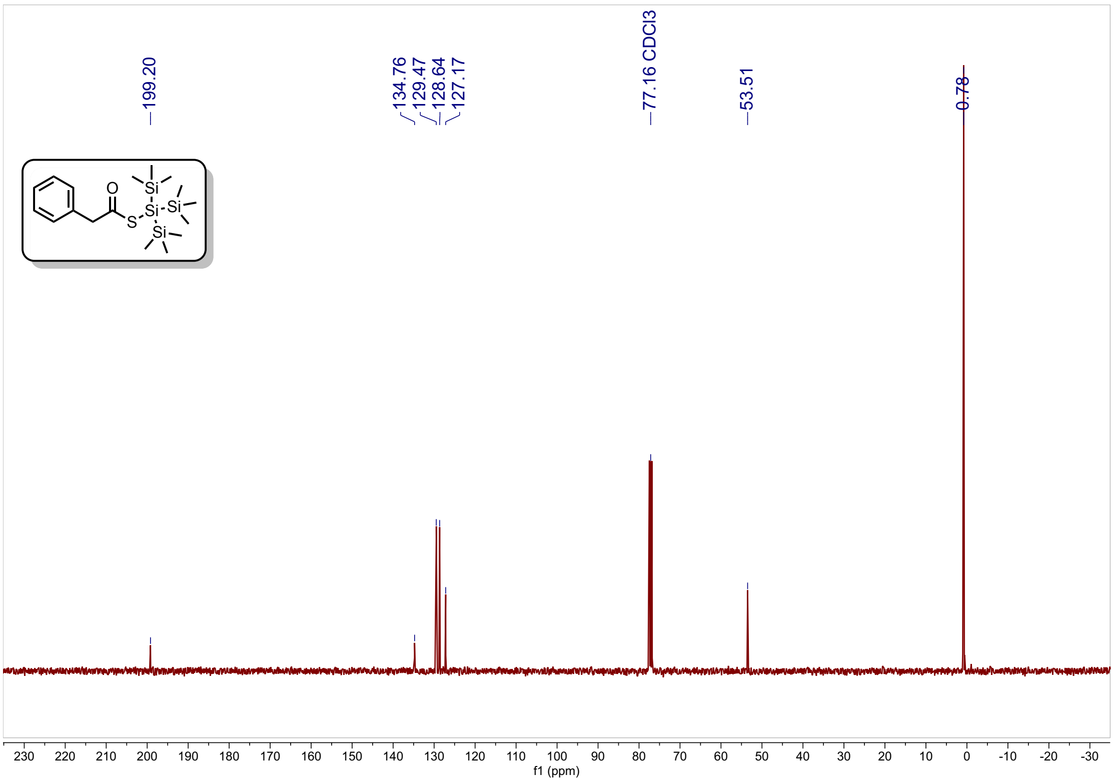

^{29}Si NMR of compound 2v (79 MHz, $CDCl_3$)

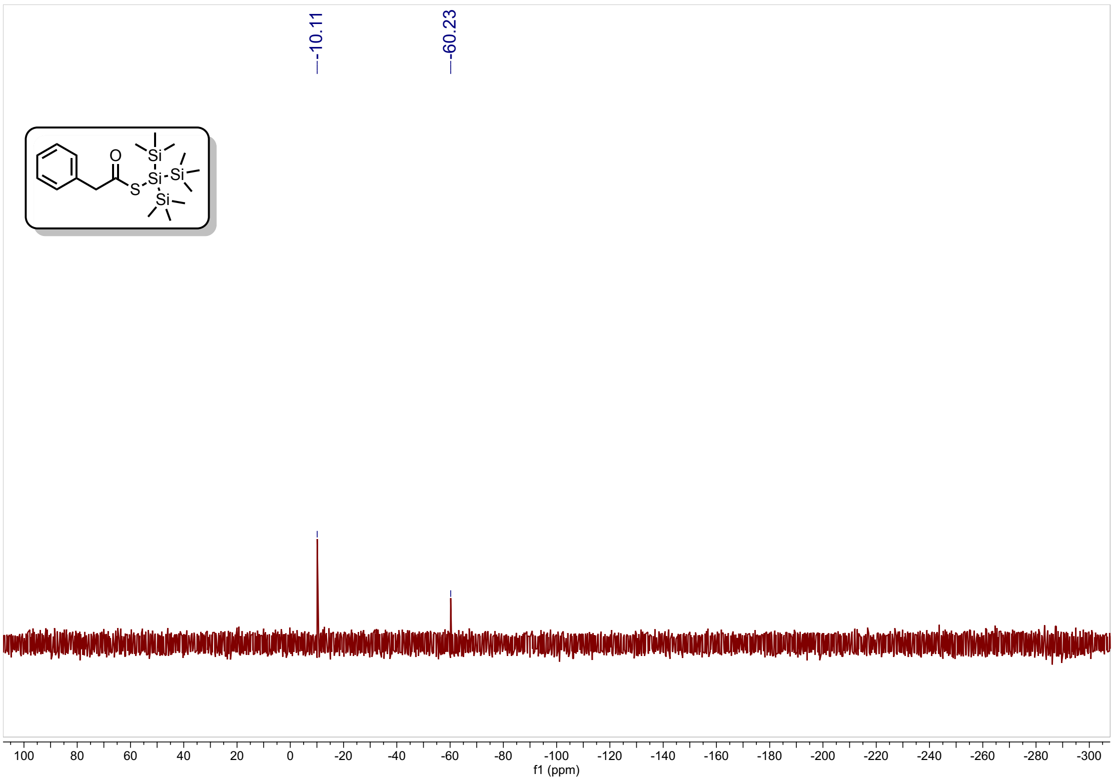

^{1}H NMR of compound 2w (600 MHz, $CDCl_3$)

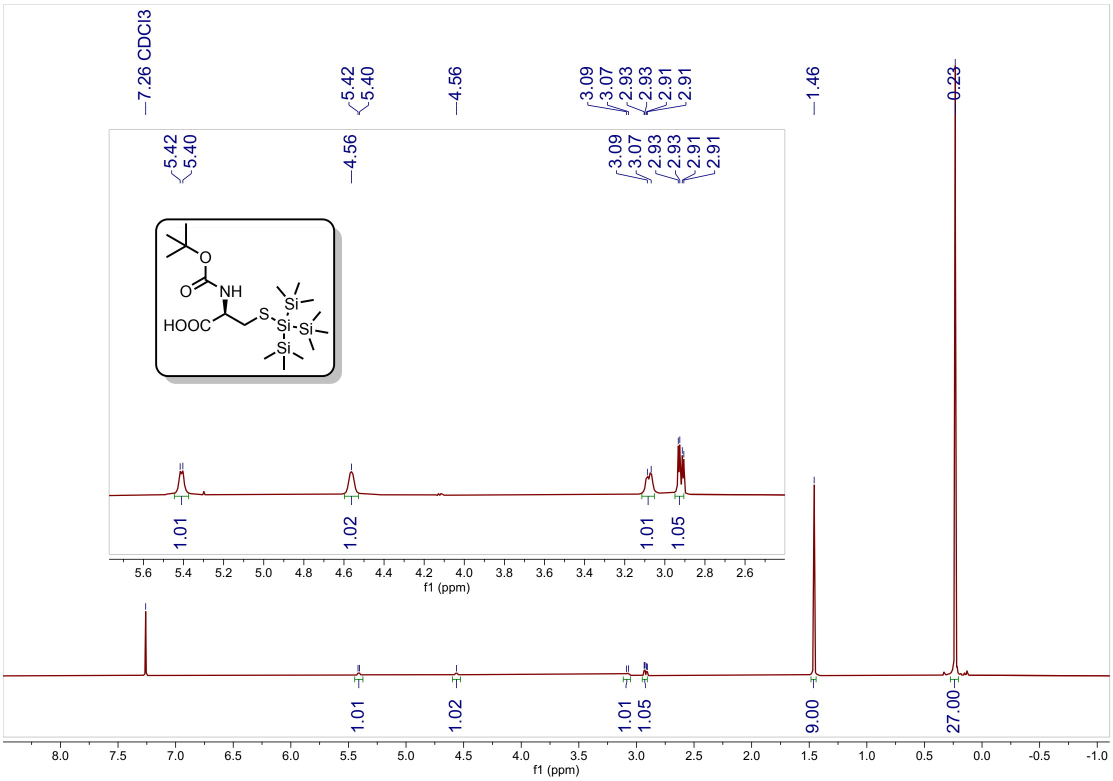

^{13}C NMR of compound 2w (100 MHz, $CDCl_3$)

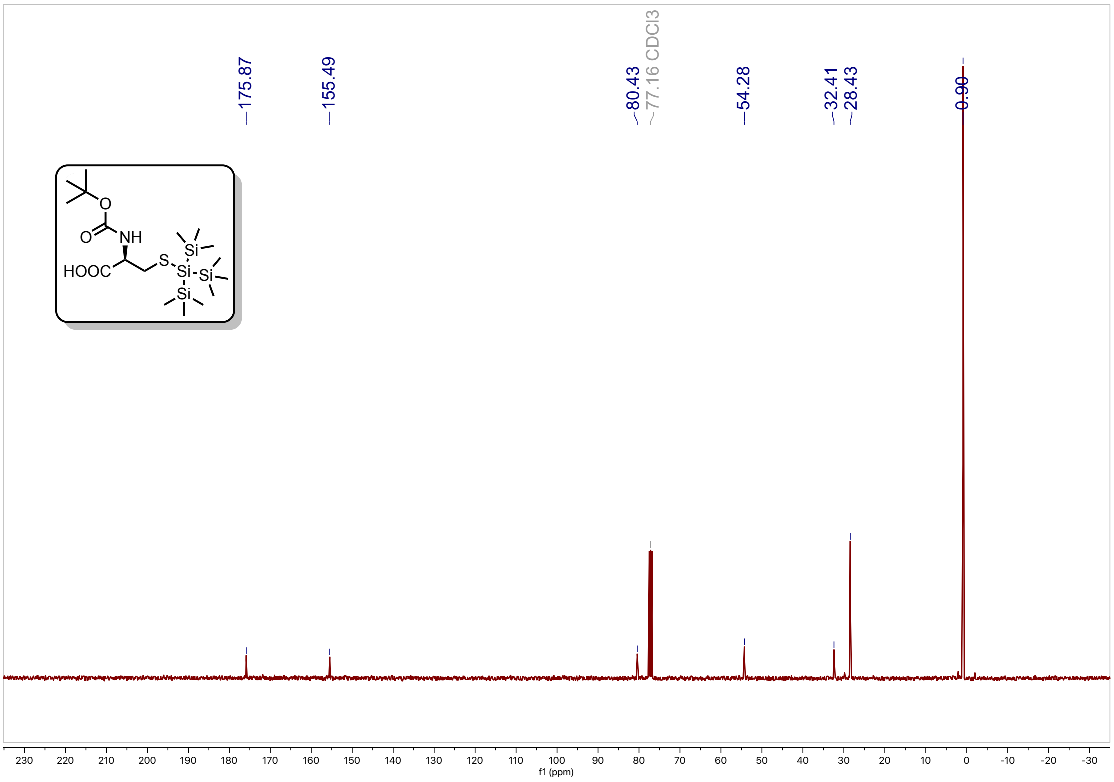

^{29}Si NMR of compound 2w (79 MHz, $CDCl_3$)

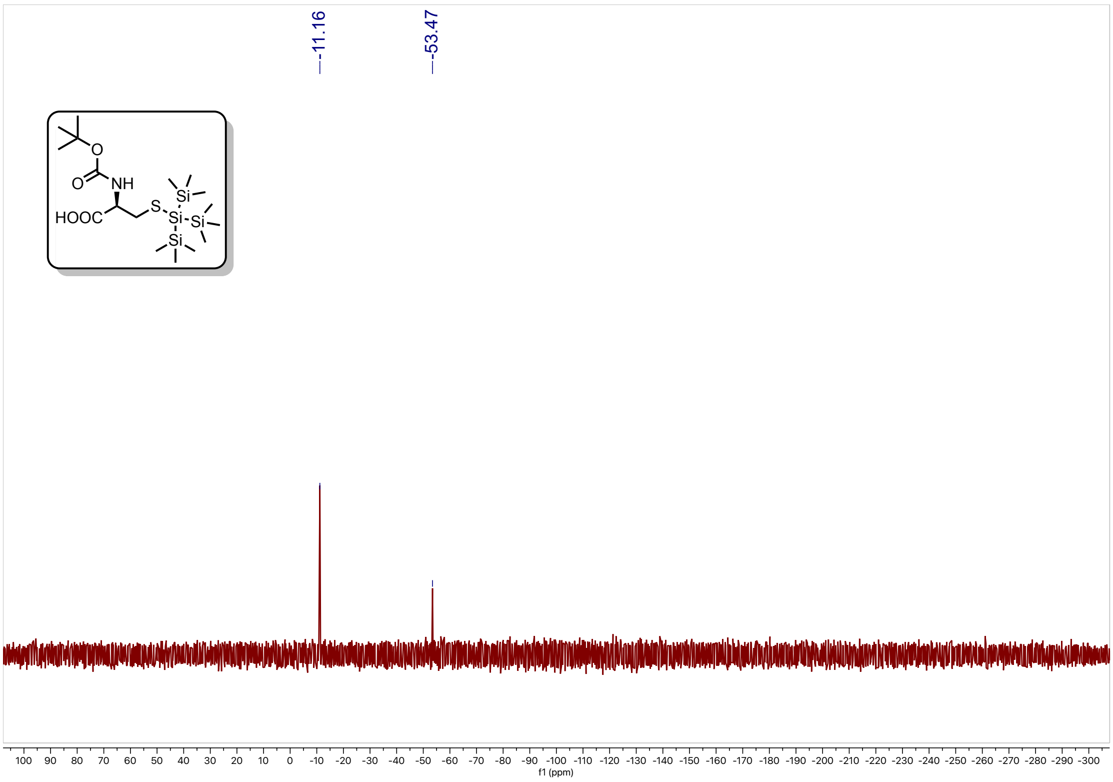

^{1}H NMR of compound 2x (400 MHz, $CDCl_3$)

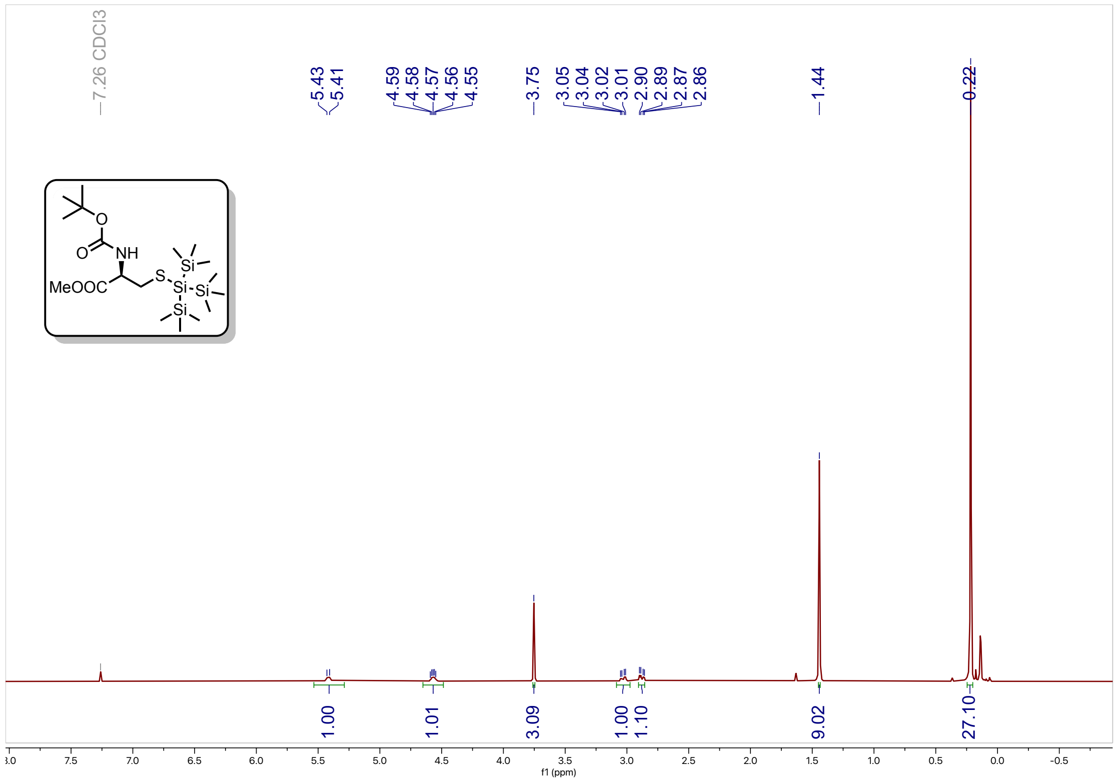

^{13}C NMR of compound 2x (100 MHz, $CDCl_3$)

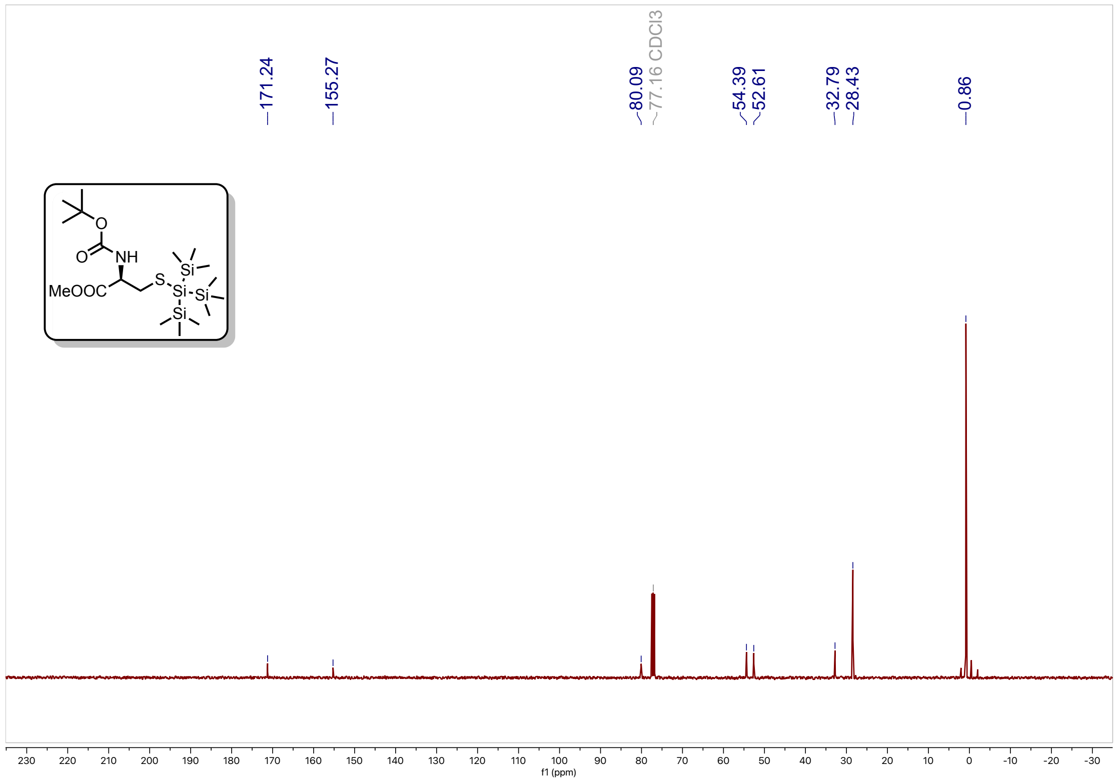

^{29}Si NMR of compound 2x (79 MHz, $CDCl_3$)

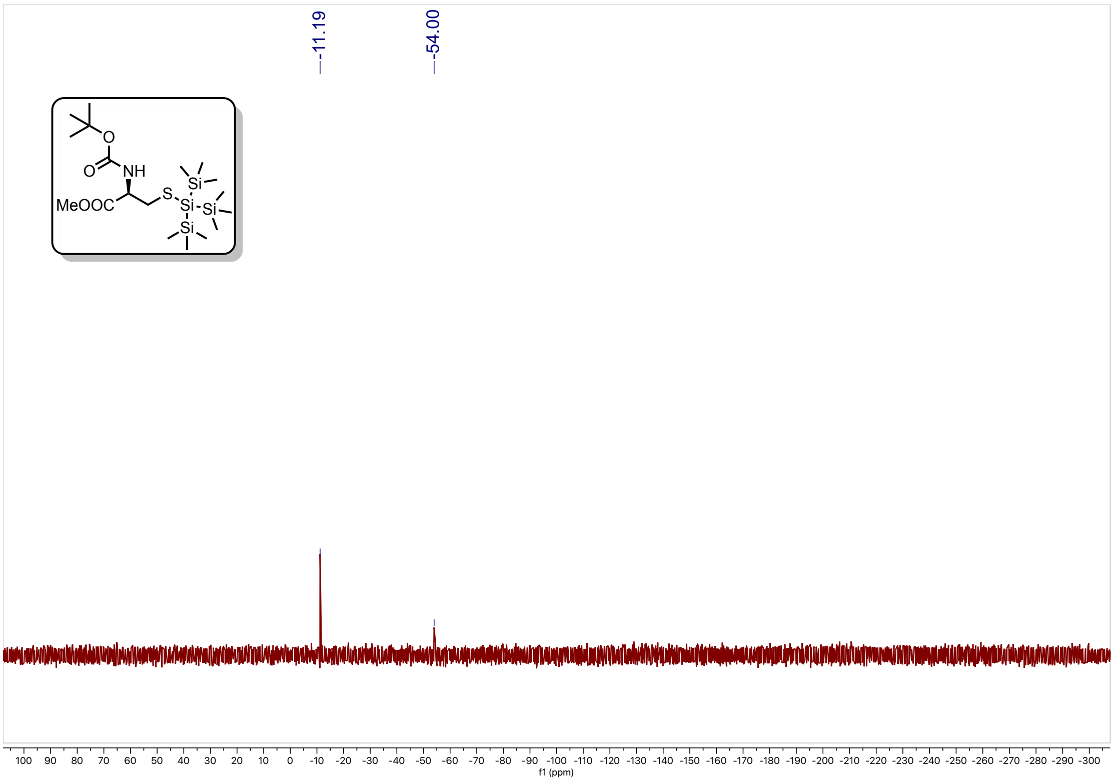

^{1}H NMR of compound 2y (400 MHz, $CDCl_3$)

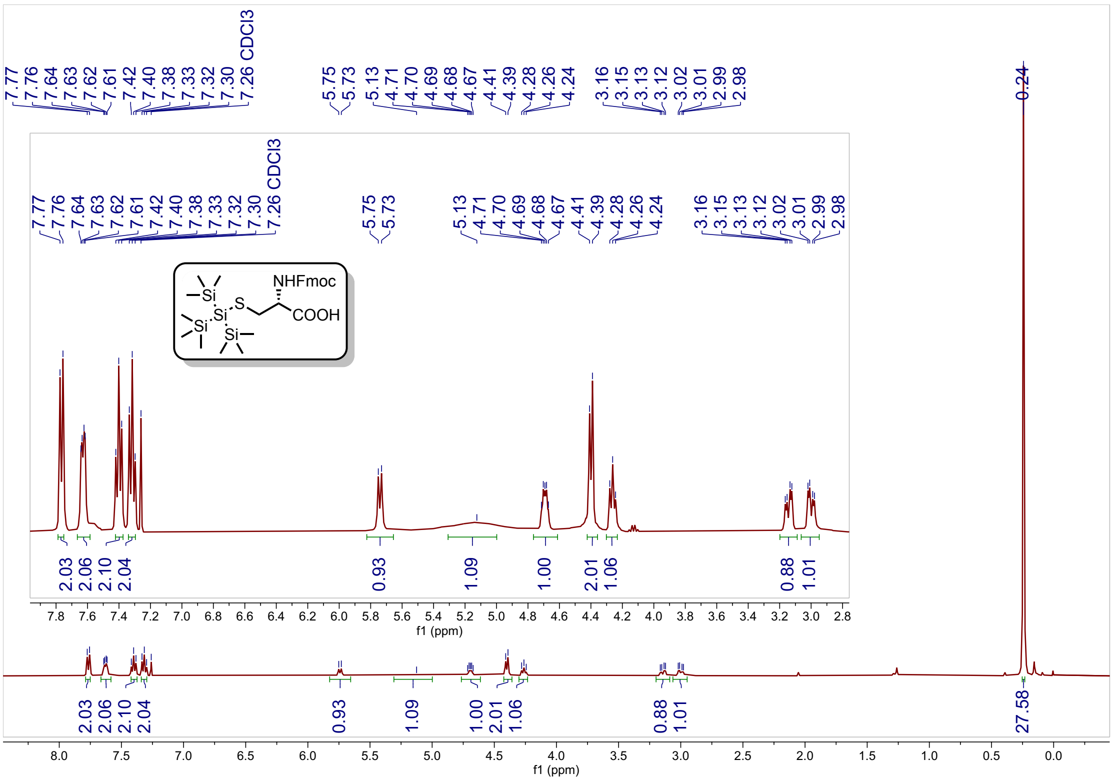

^{13}C NMR of compound 2y (100 MHz, $CDCl_3$)

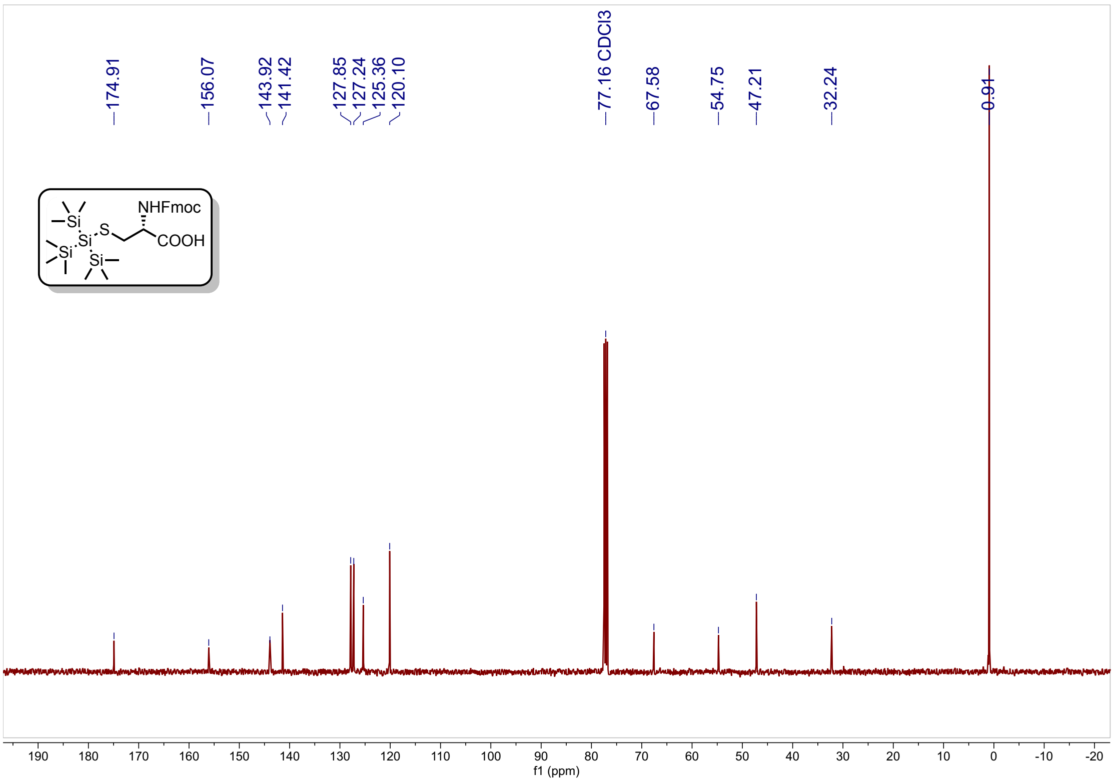

^{29}Si NMR of compound 2y (79 MHz, $CDCl_3$)

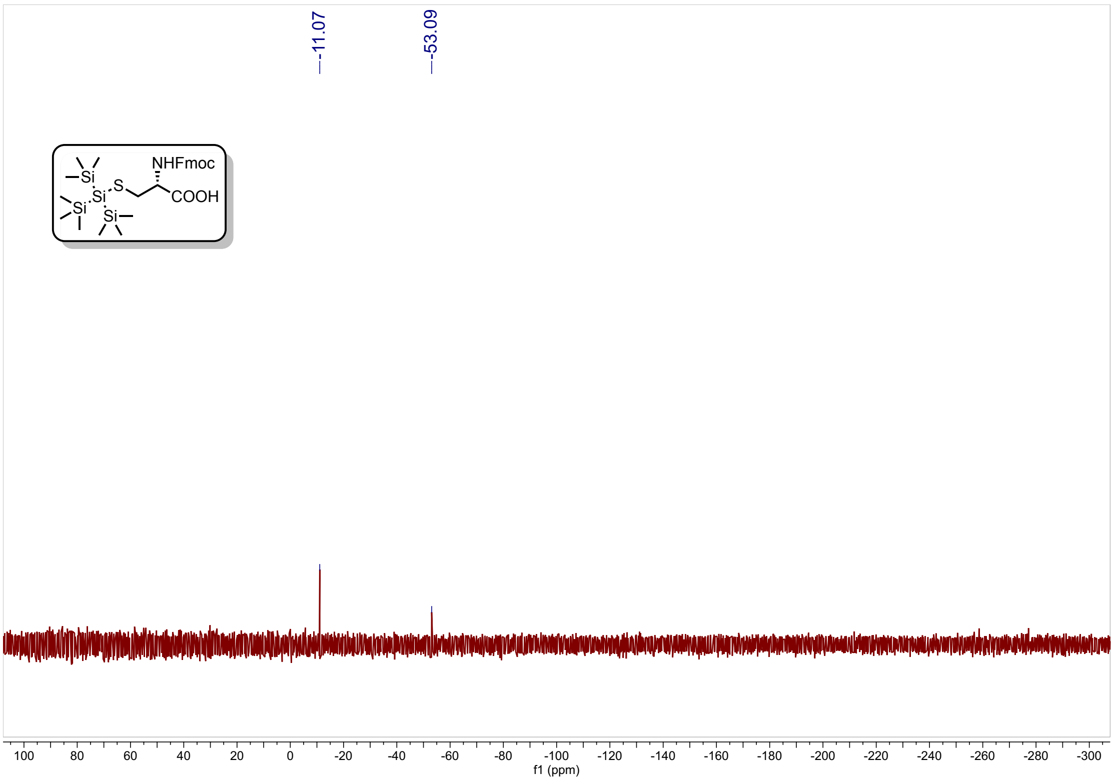

^{1}H NMR of compound 2z (400 MHz, $CDCl_3$)

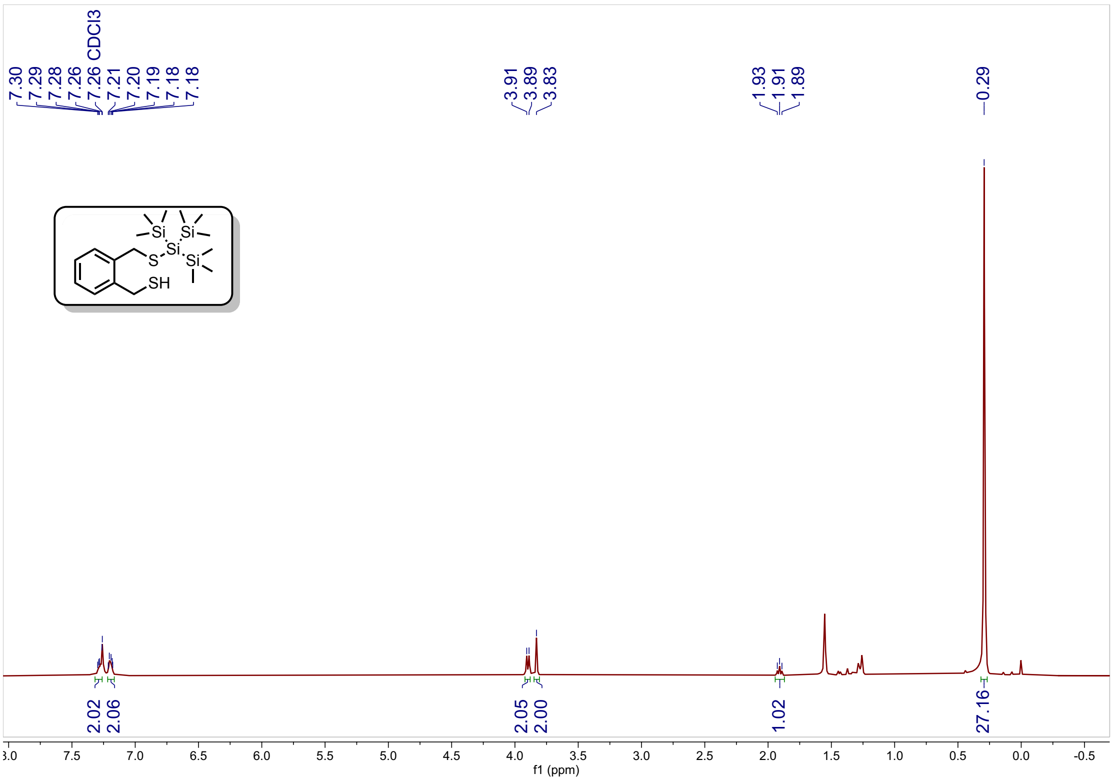

^{13}C NMR of compound 2z (100 MHz, $CDCl_3$)

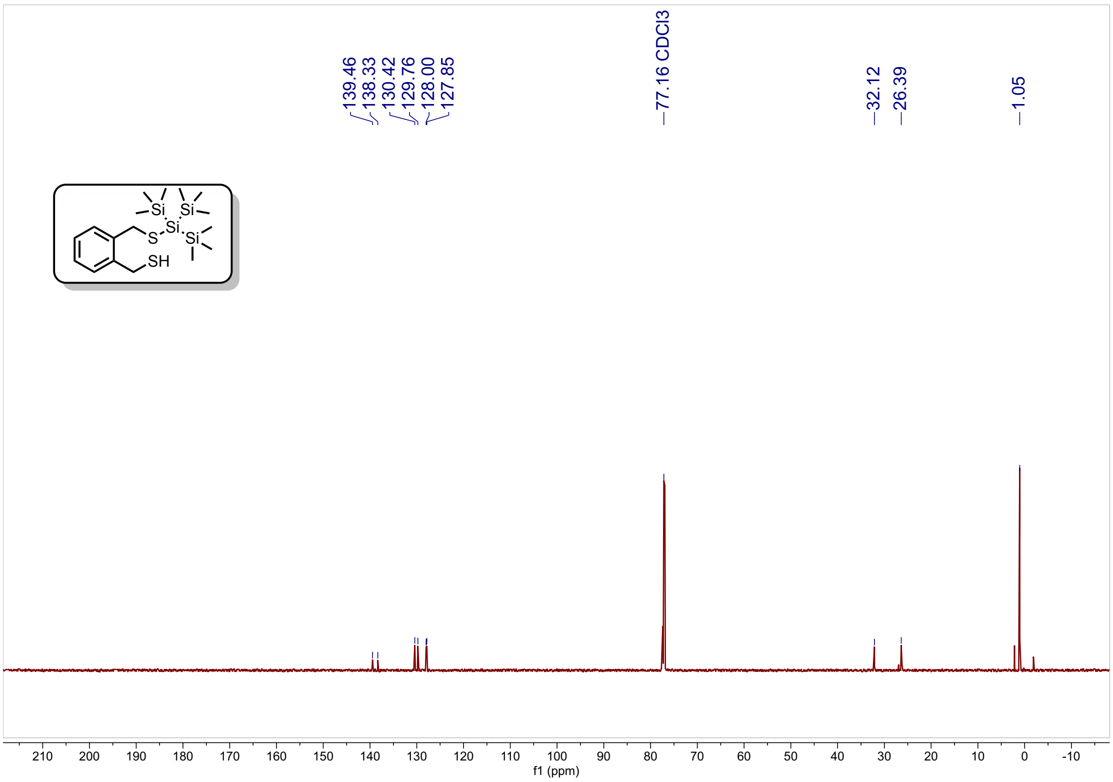

^{29}Si NMR of compound 2z (119 MHz, $CDCl_3$)

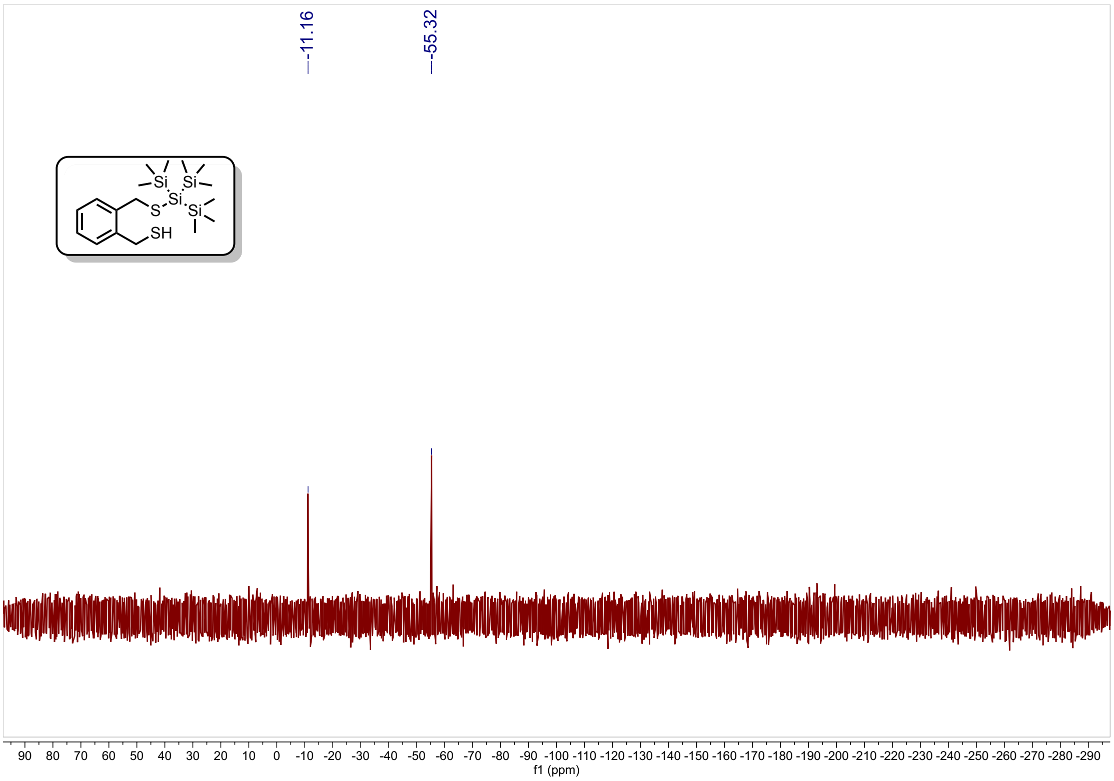

^{1}H NMR of compound 2z1 (400 MHz, $CDCl_3$)

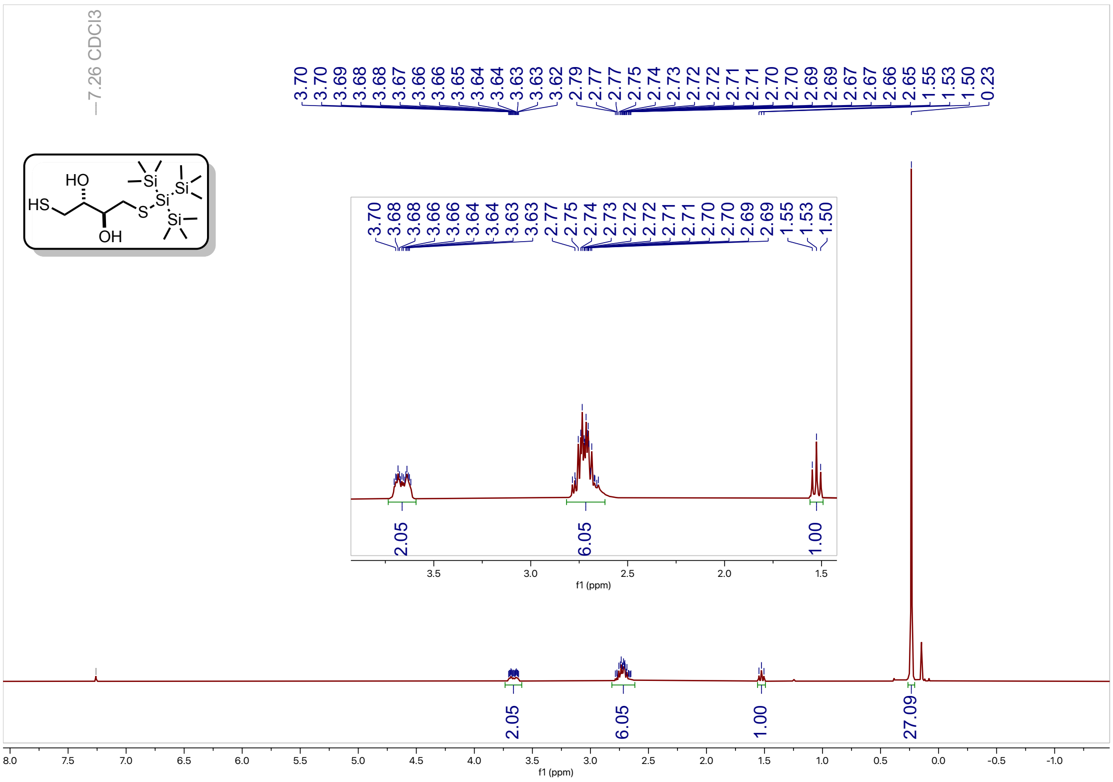

^{13}C NMR of compound 2z1 (100 MHz, $CDCl_3$)

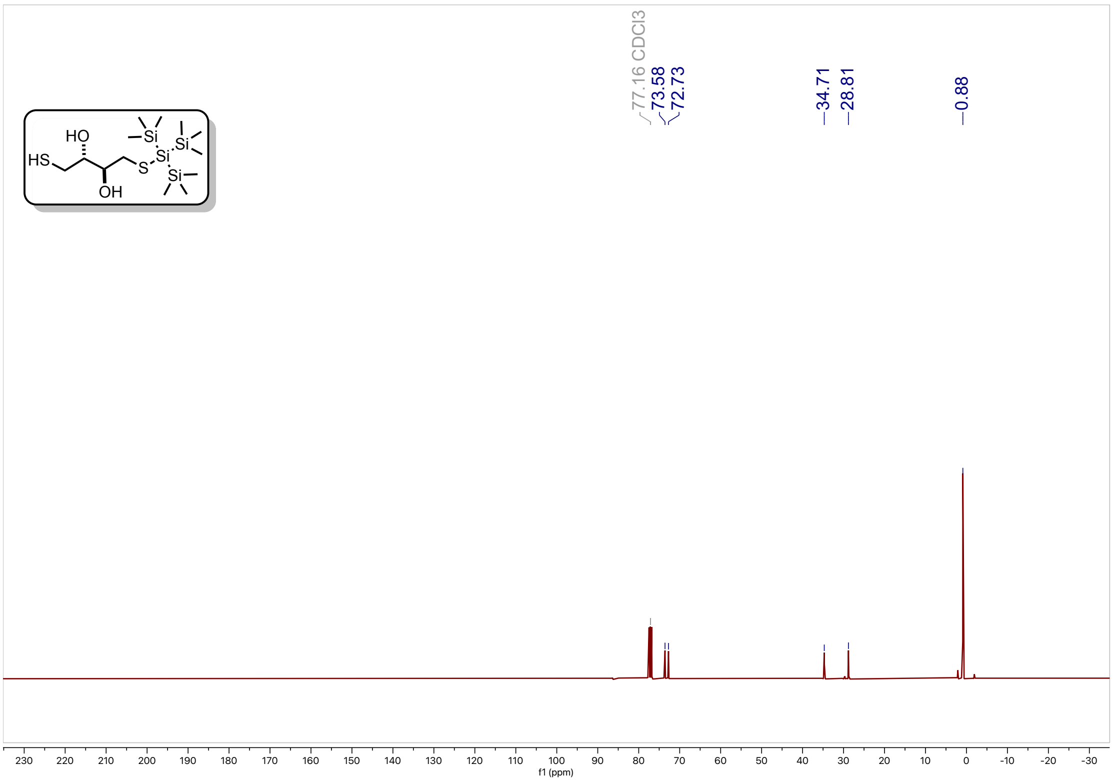

^{29}Si NMR of compound 2z1 (79 MHz, $CDCl_3$)

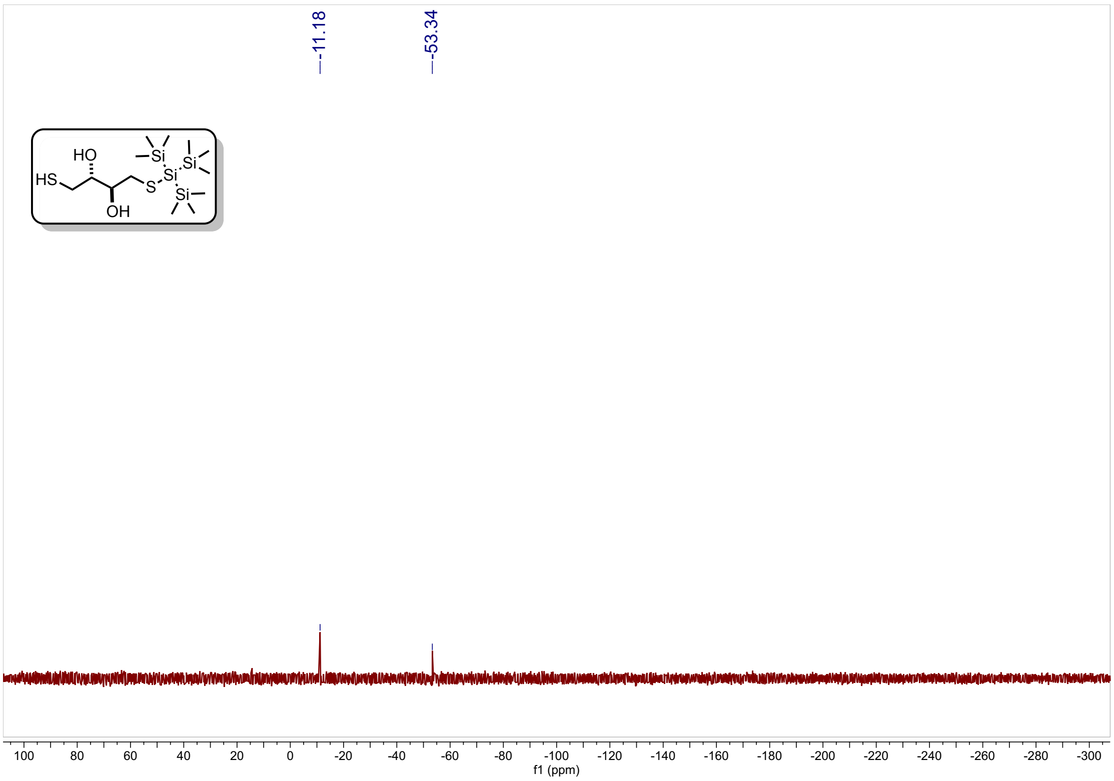

^{1}H NMR of compound 2z2(400 MHz, $CDCl_3$)

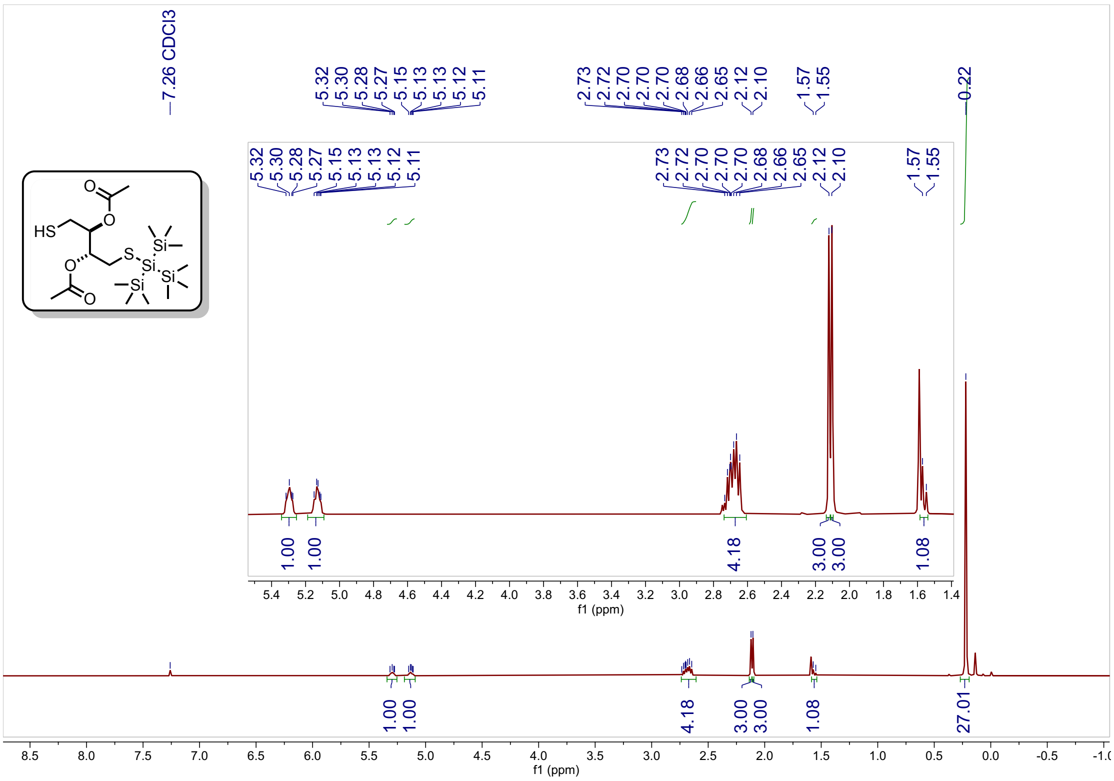

^{1}H NMR of compound 2z2 (400 MHz, $CDCl_3$)

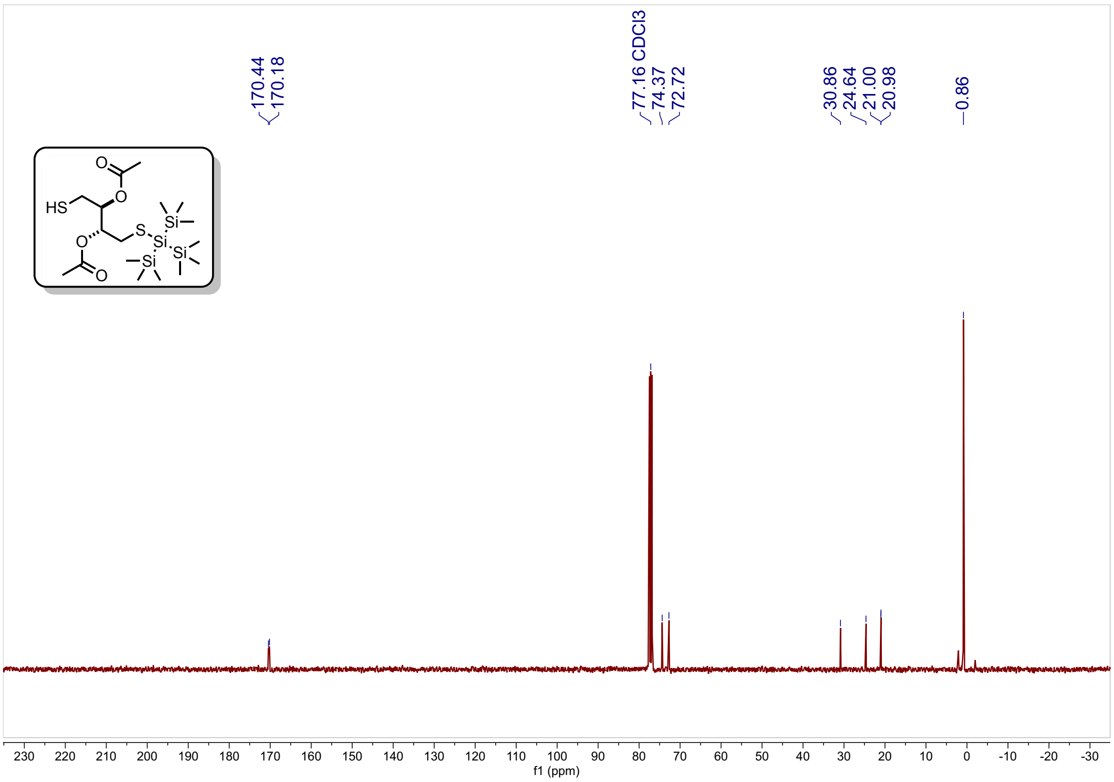

^{29}Si NMR of compound 2z2 (79 MHz, CDCl$_3$)

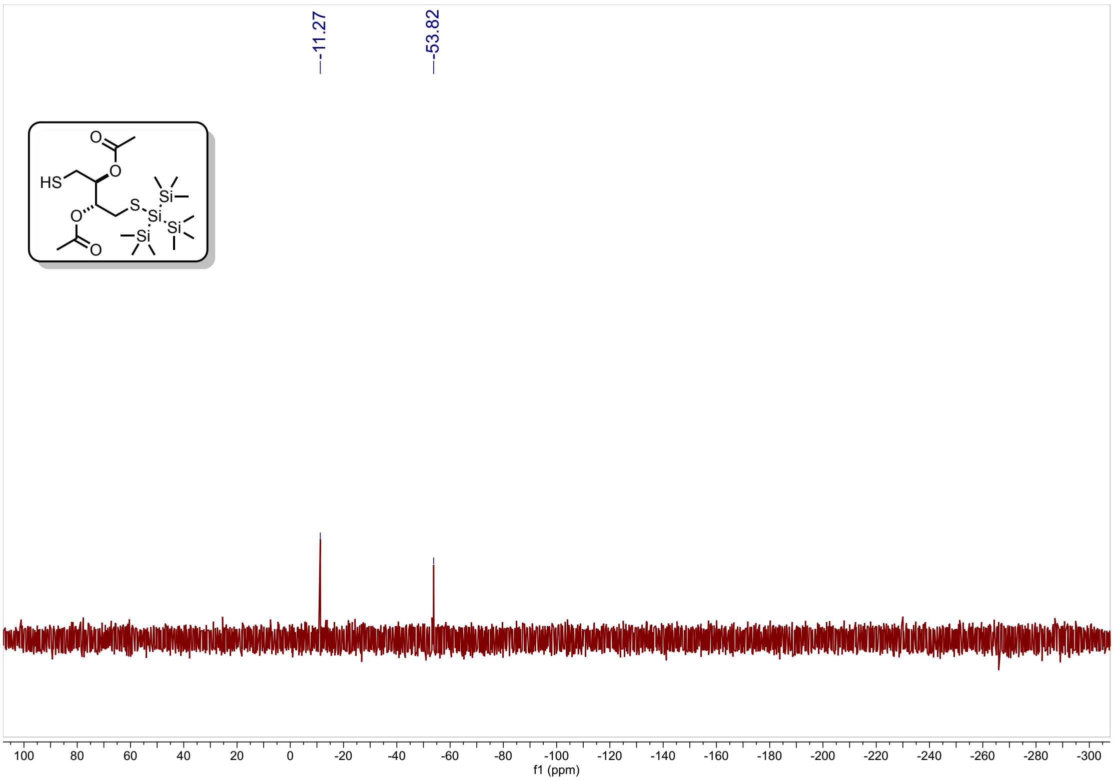

^{1}H NMR of compound 2z3 (400 MHz, $CDCl_3$)

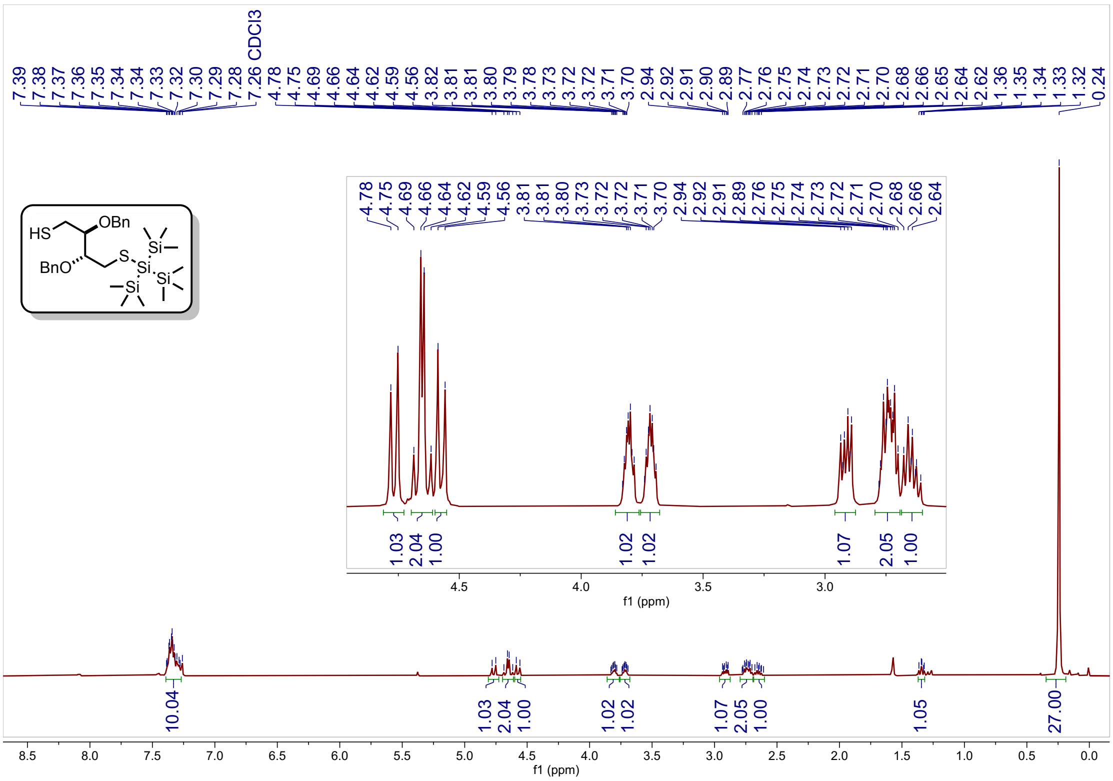

^{13}C NMR of compound 2z3 (100 MHz, $CDCl_3$)

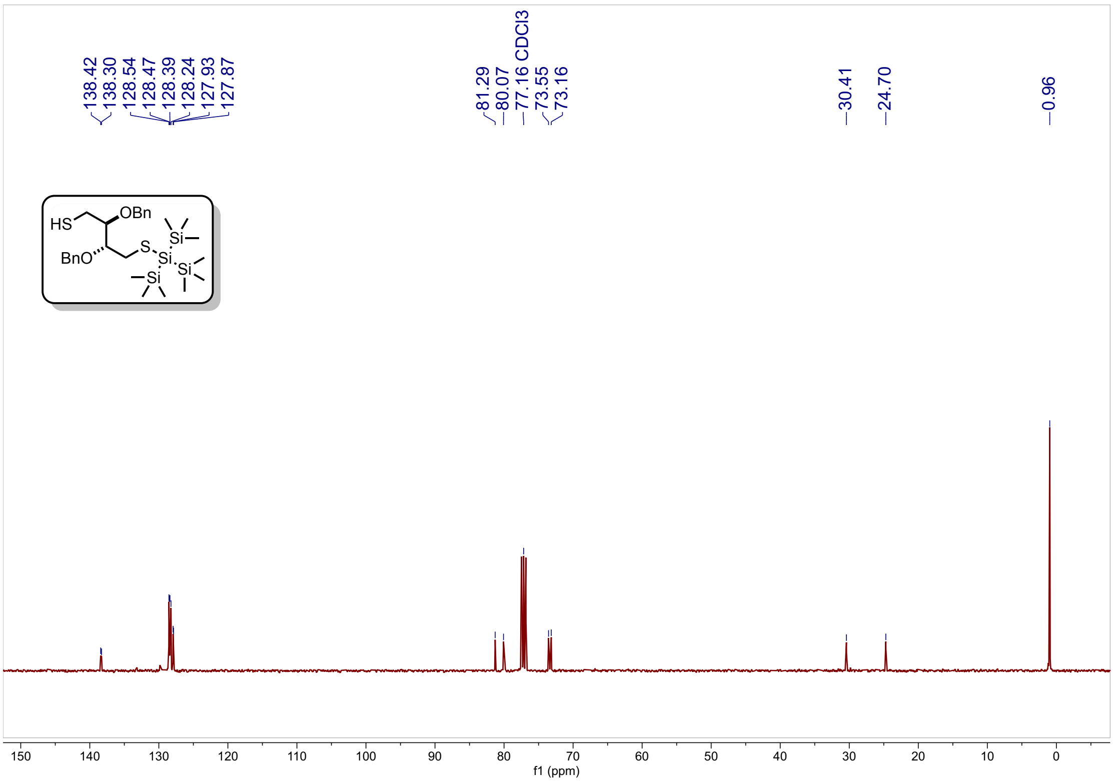

^{29}Si NMR of compound 2z3 (79 MHz, $CDCl_3$) z

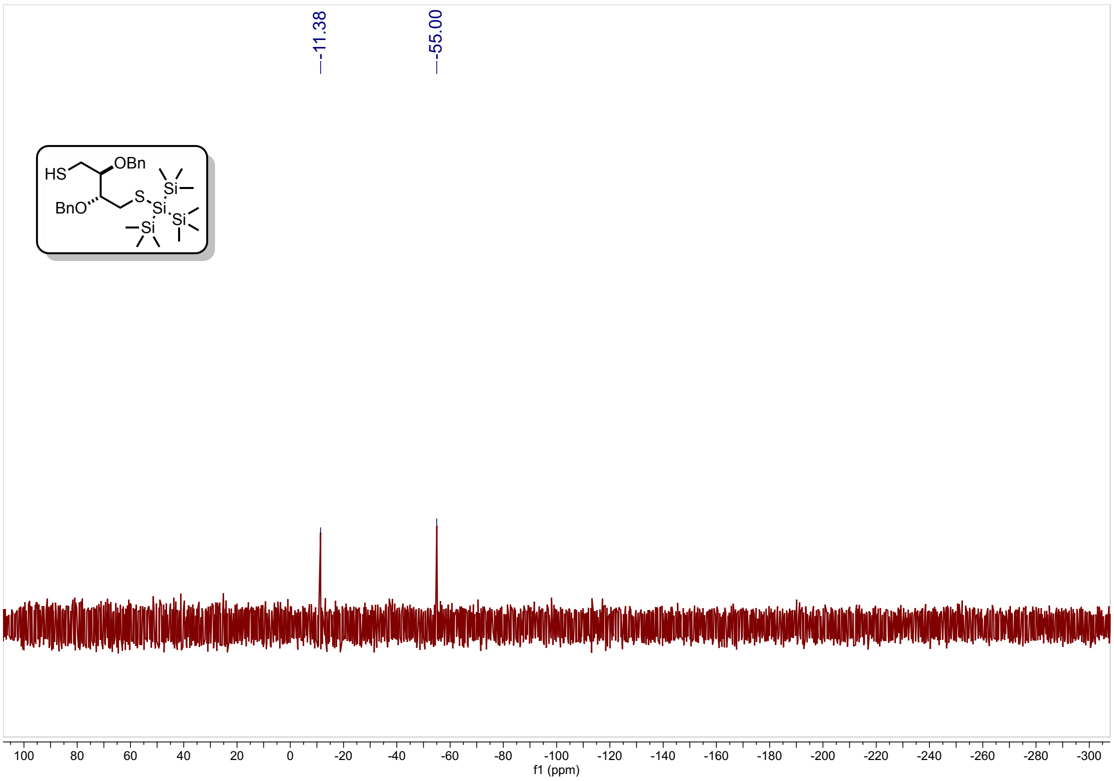

^{1}H NMR of compound 2z4 (400 MHz, $CDCl_3$)

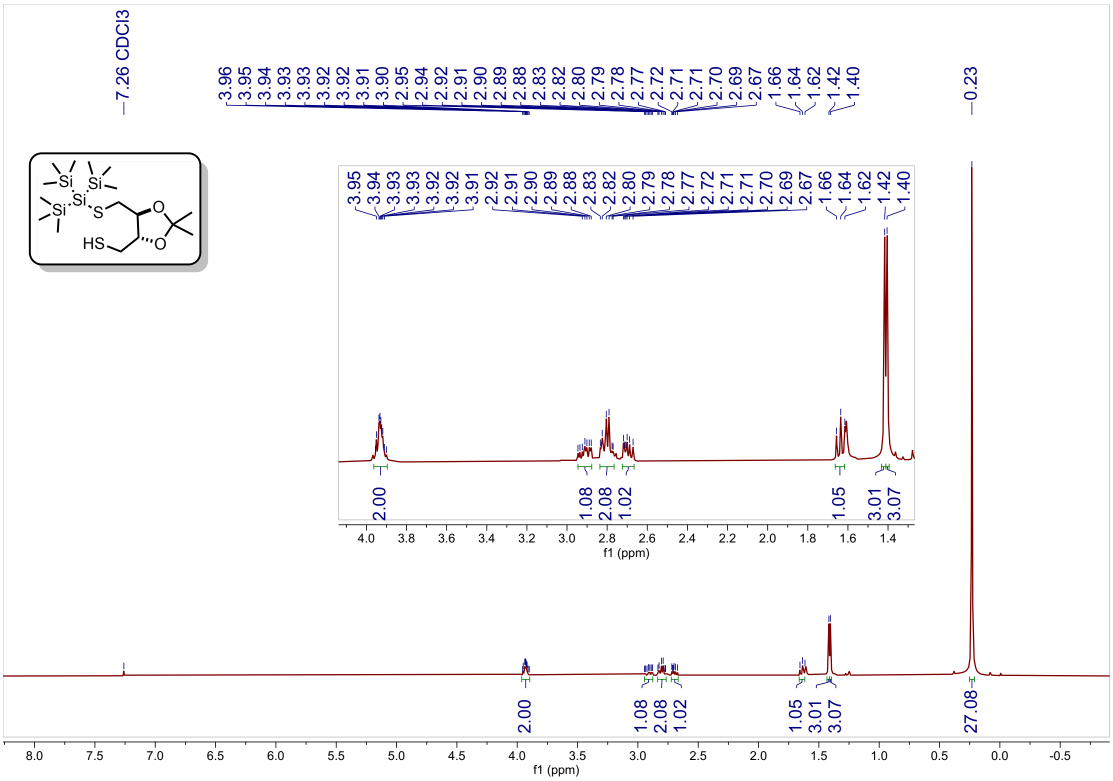

^{13}C NMR of compound 2z4 (100 MHz, $CDCl_3$)

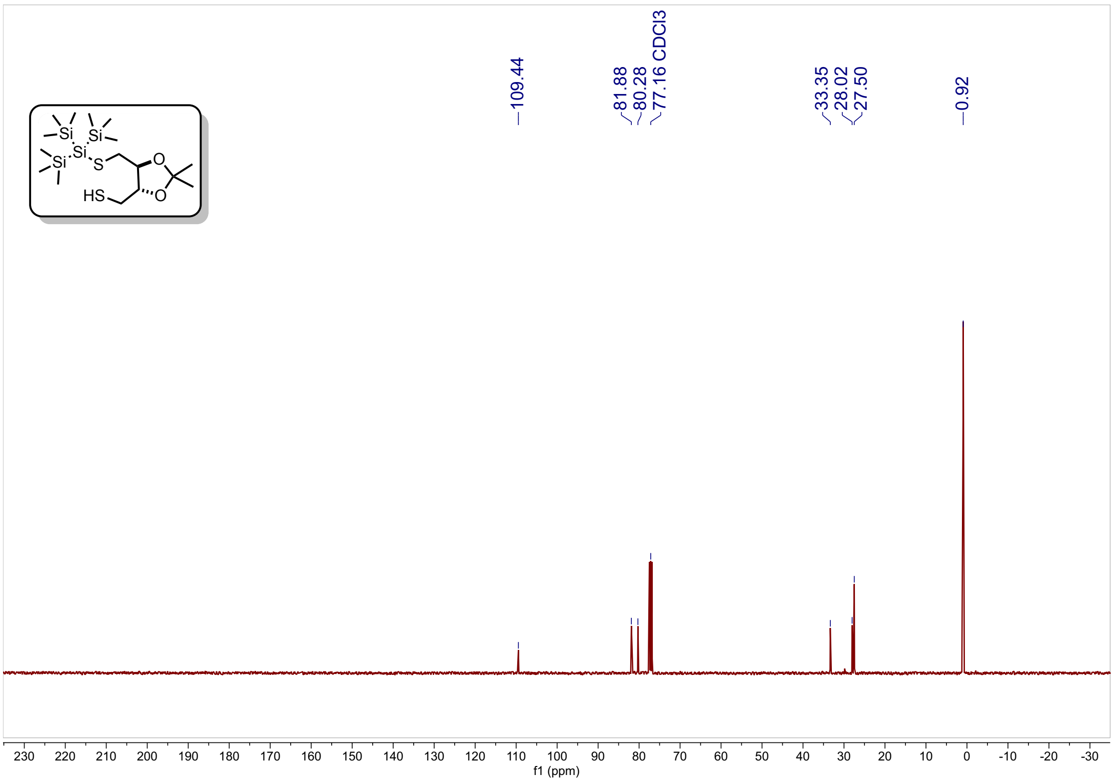

^{29}Si NMR of compound 2z4 (79 MHz, CDCl$_3$)

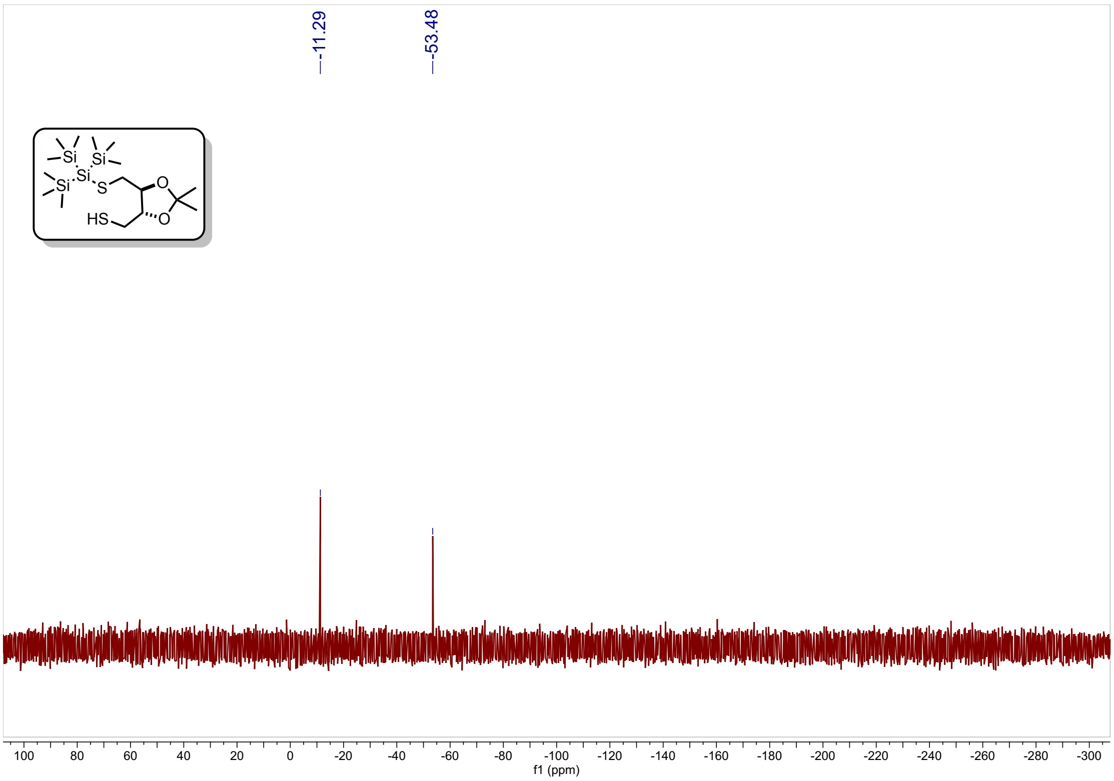

^{1}H NMR of compound 2z5 (600 MHz, $CDCl_3$)

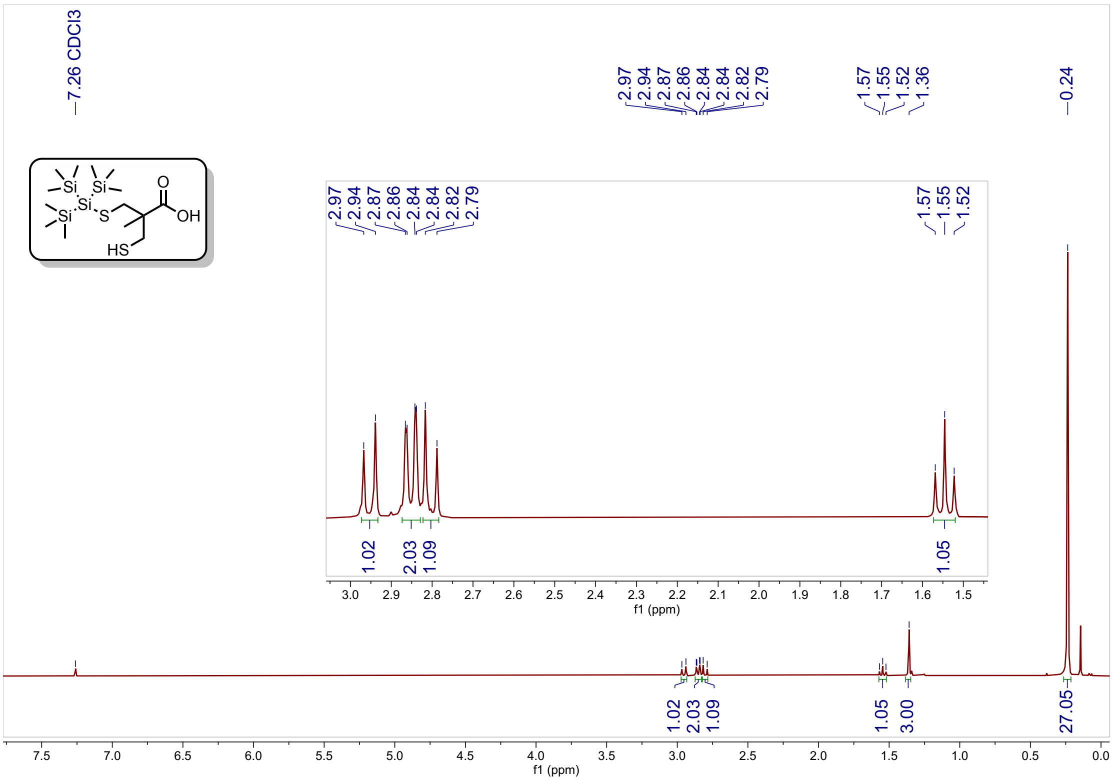

^{13}C NMR of compound 2z5 (150 MHz, $CDCl_3$)

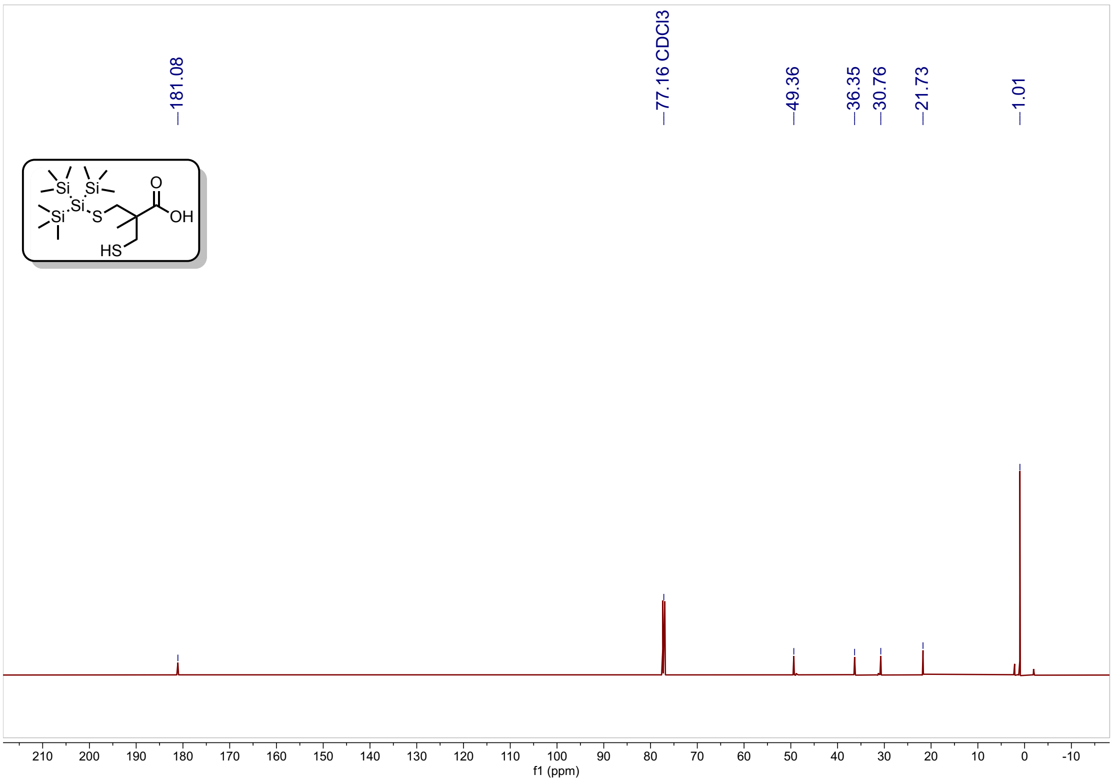

^{29}Si NMR of compound 2z5 (119 MHz, $CDCl_3$)

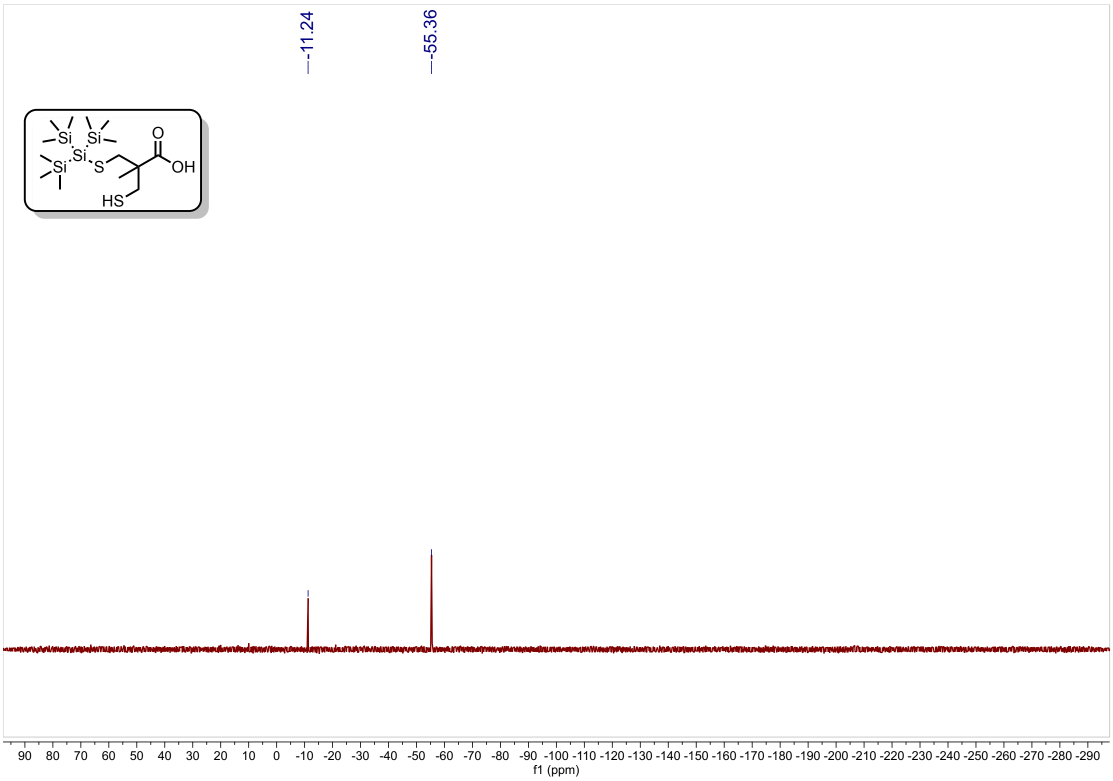

^{1}H NMR of compound 2z6 (400 MHz, $CDCl_3$)

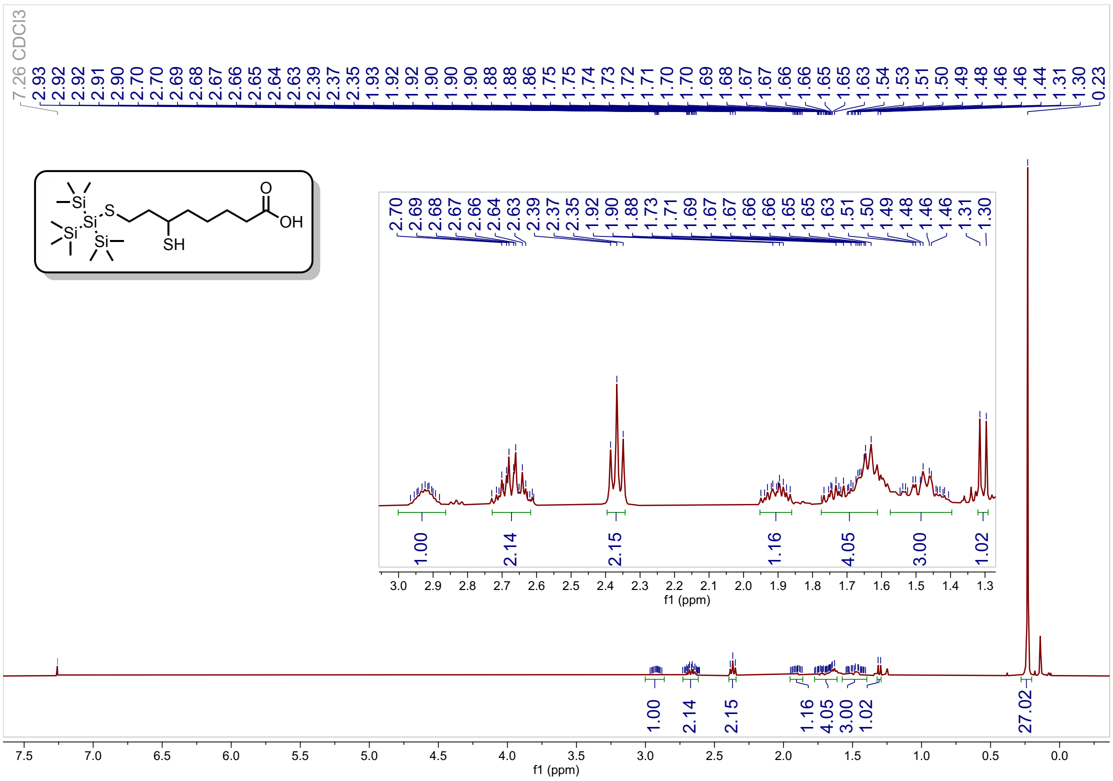

^{13}C NMR of compound 2z6 (100 MHz, $CDCl_3$)

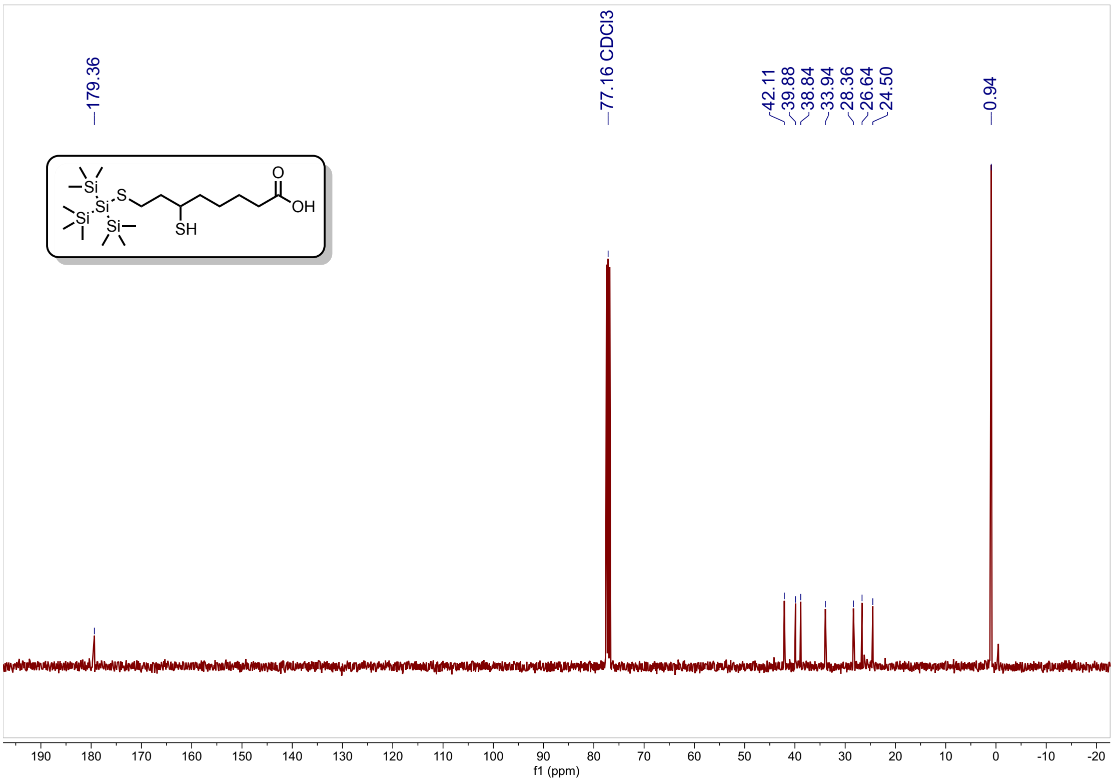

^{29}Si NMR of compound 2z6 (79 MHz, $CDCl_3$)

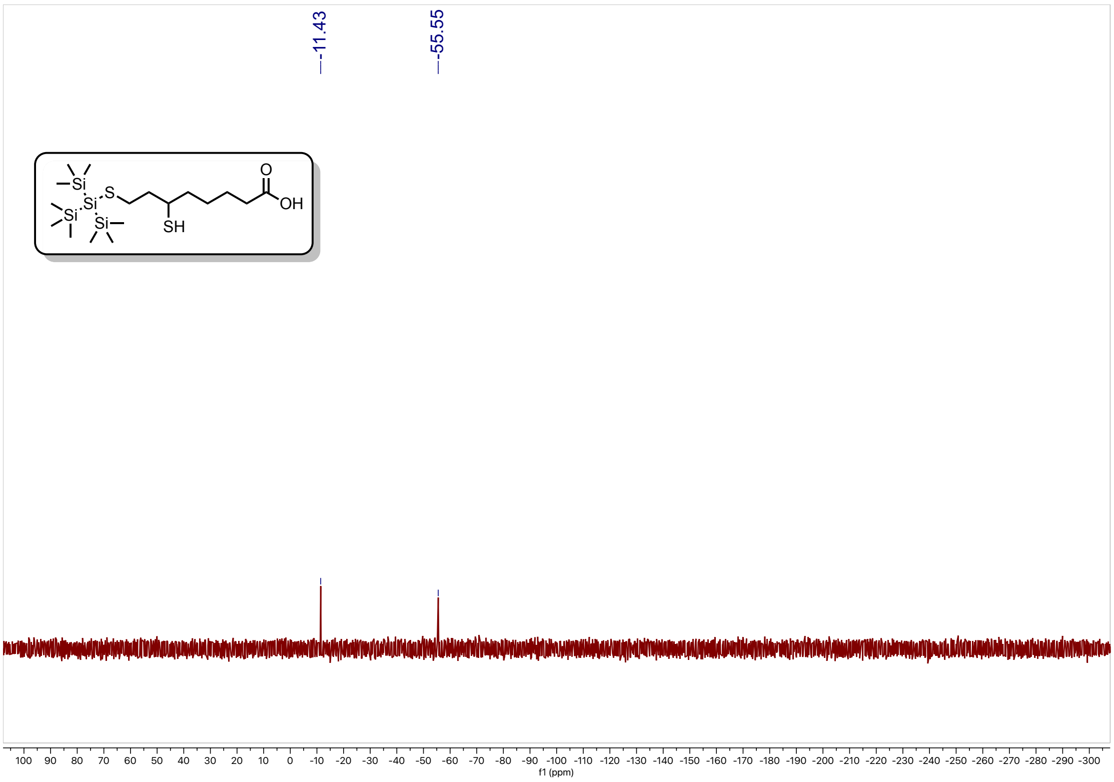

^{1}H NMR of compound 2z7 (400 MHz, $CDCl_3$)

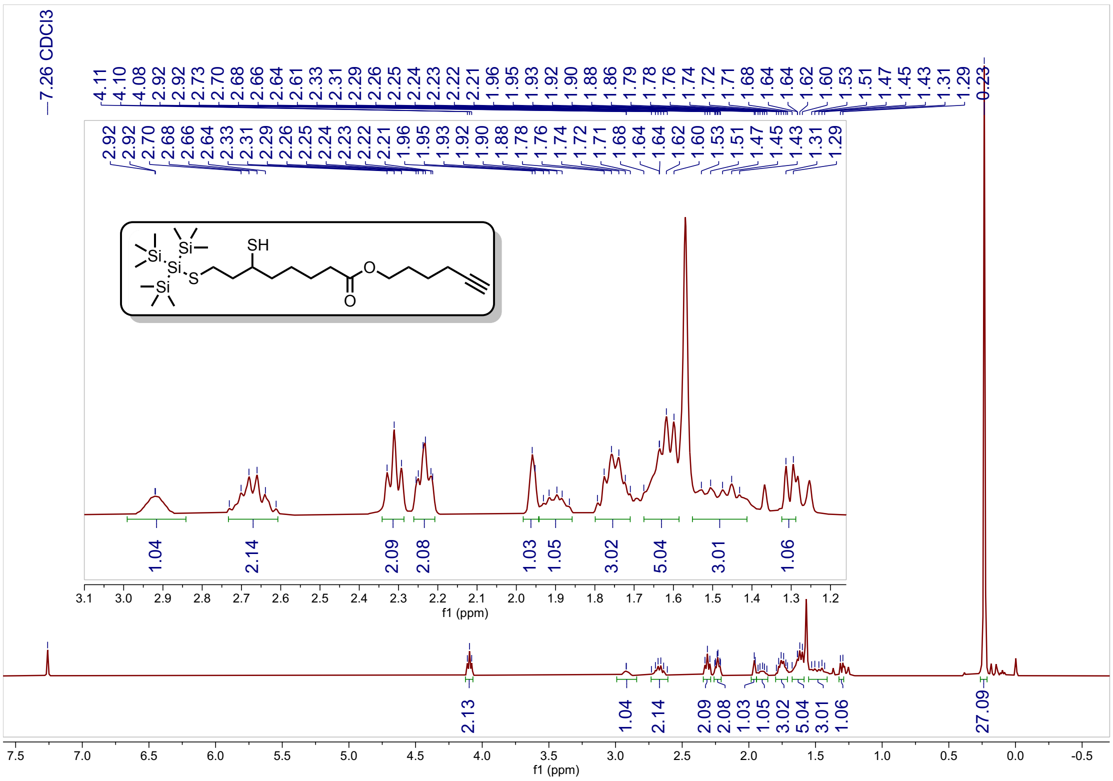

^{13}C NMR of compound 2z7 (100 MHz, $CDCl_3$)

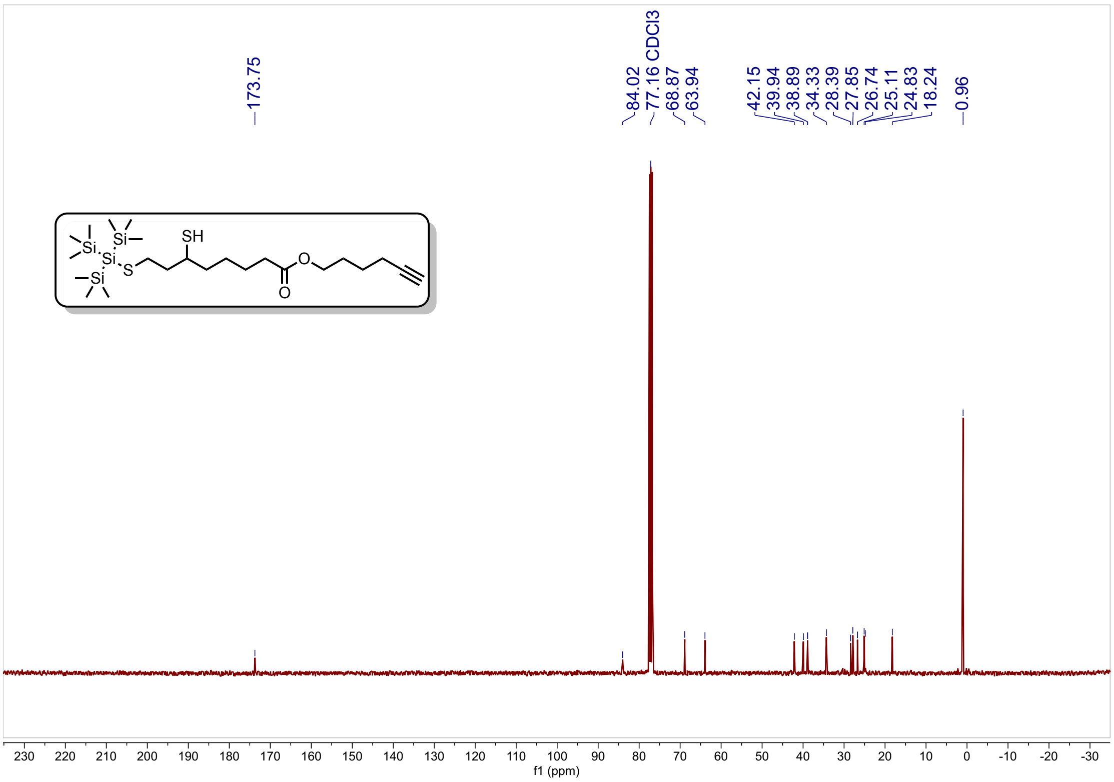

^{29}Si NMR of compound 2z7 (79 MHz, $CDCl_3$)

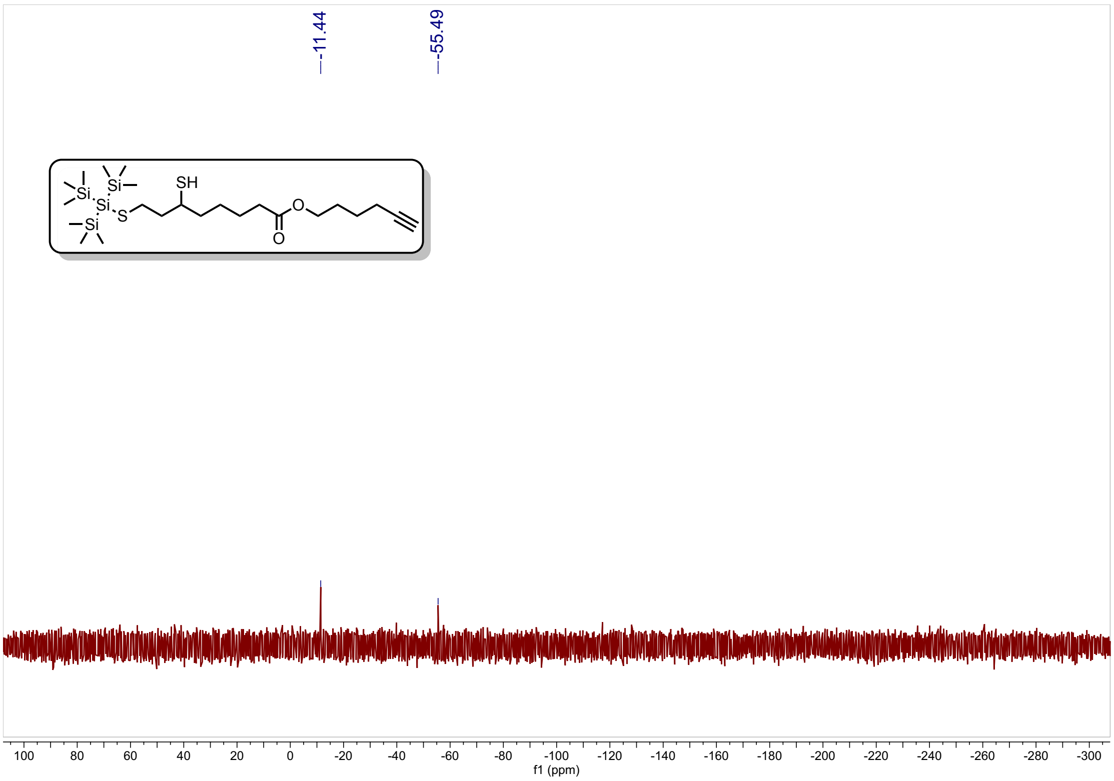

^{1}H NMR of compound 2z8 (400 MHz, $CDCl_3$)

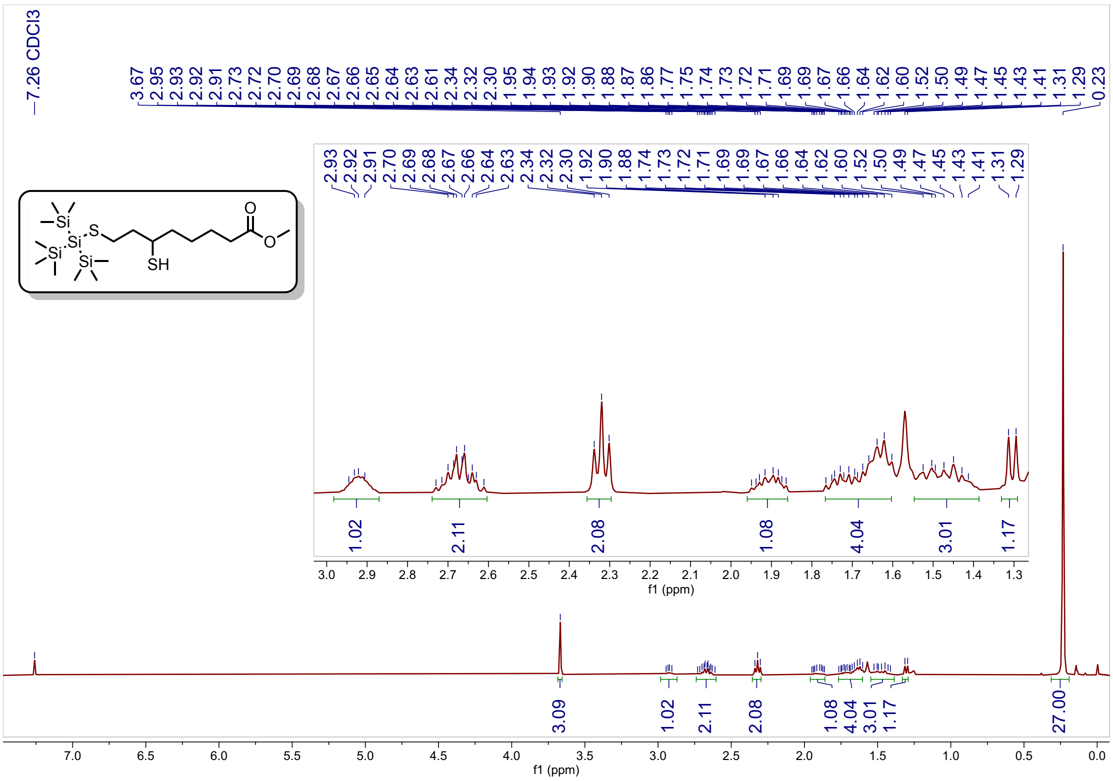

^{13}C NMR of compound 2z8 (100 MHz, $CDCl_3$)

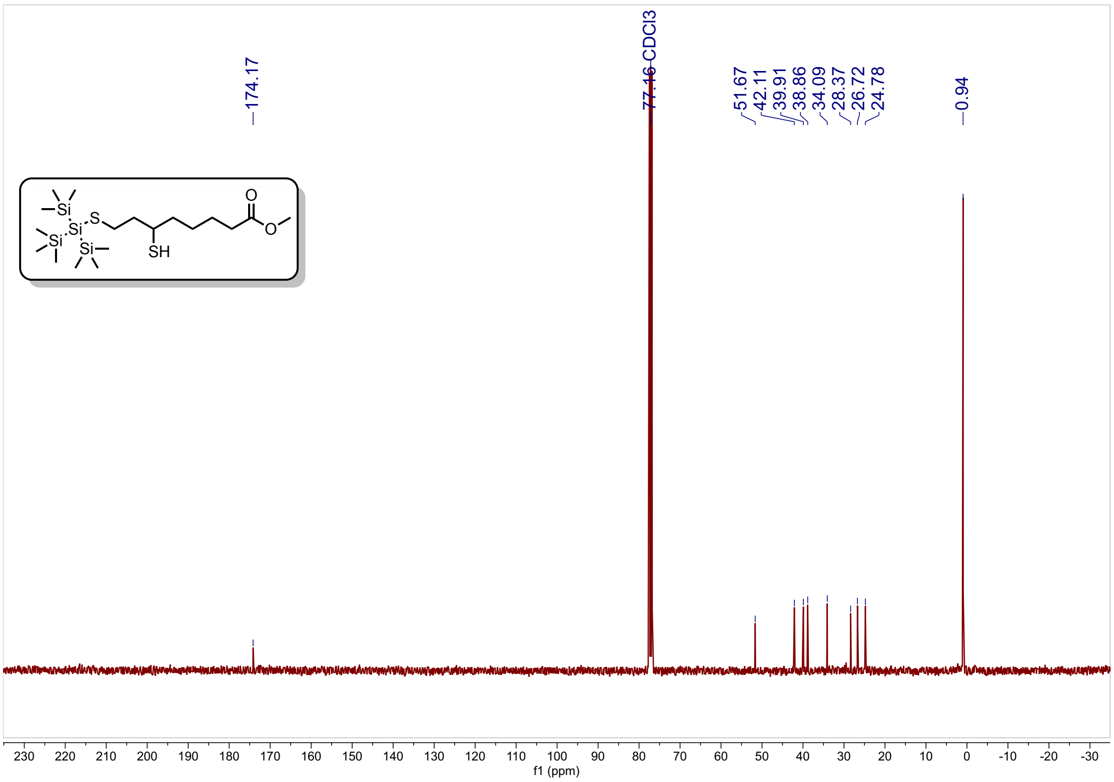

^{29}Si NMR of compound 2z8 (79 MHz, $CDCl_3$)

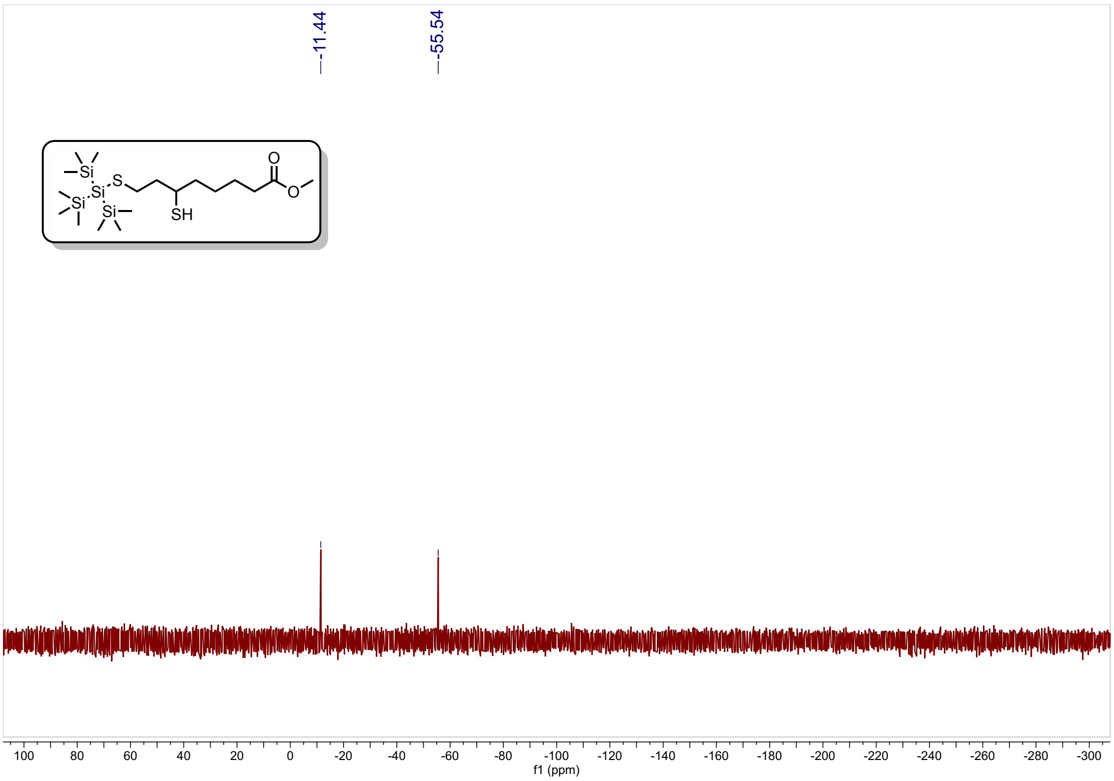

^{1}H NMR of compound 2z9 (400 MHz, CDCl$_3$)

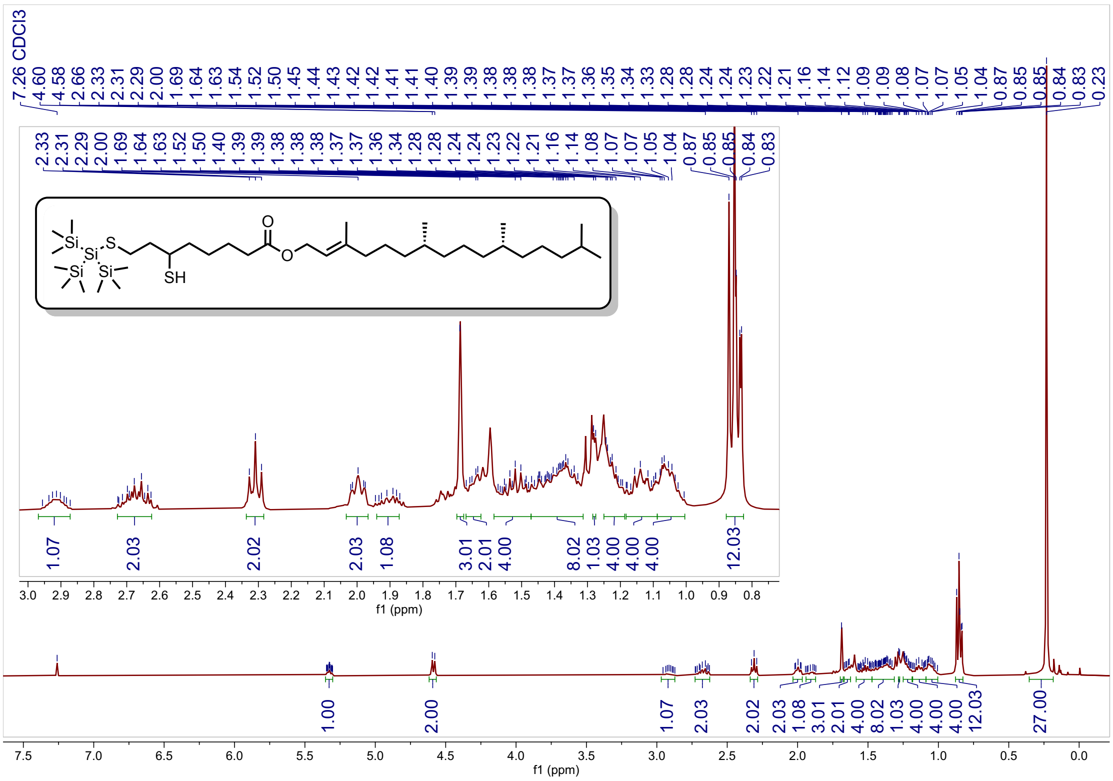

^{13}C NMR of compound 2z9 (100 MHz, CDCl$_3$)

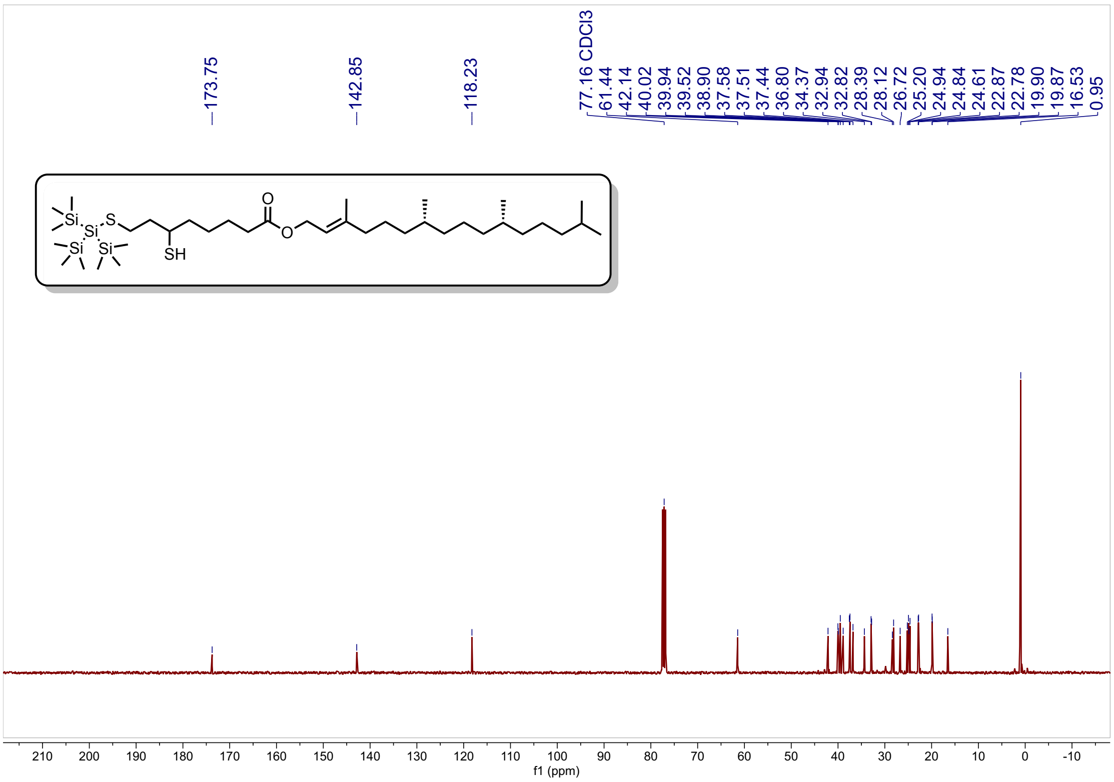

^{29}Si NMR of compound 2z9 (79 MHz, $CDCl_3$)

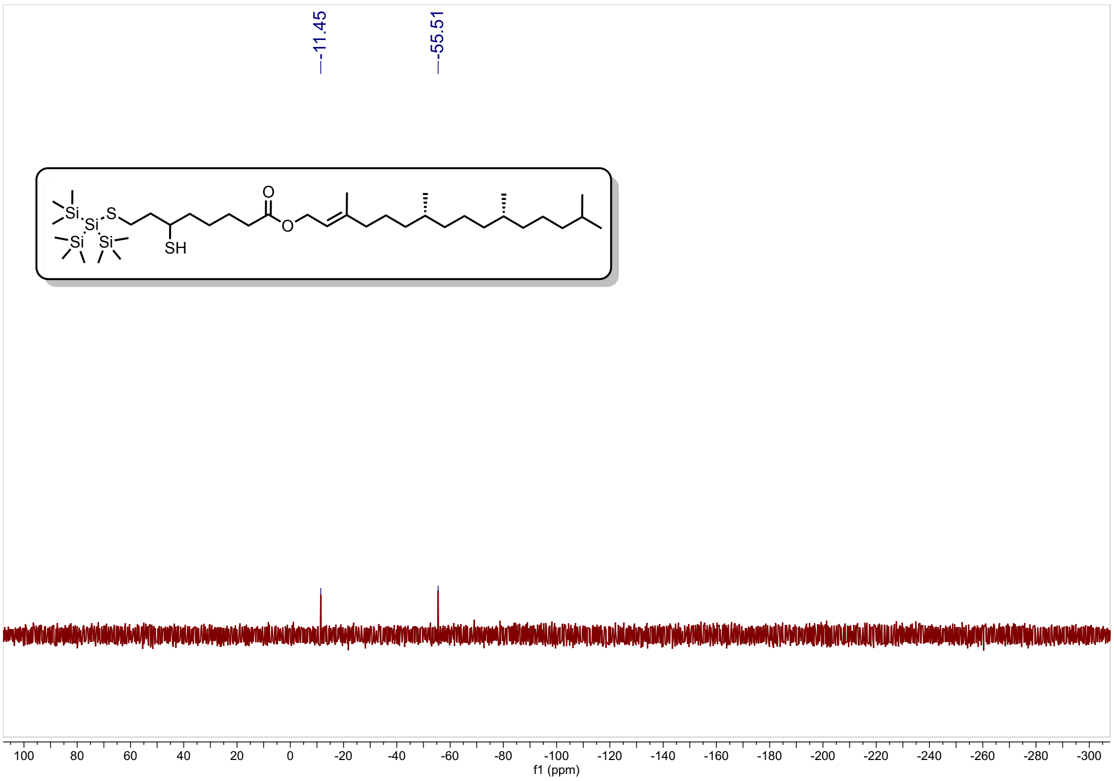

^{1}H NMR of compound 2z10 (400 MHz, $CDCl_3$)

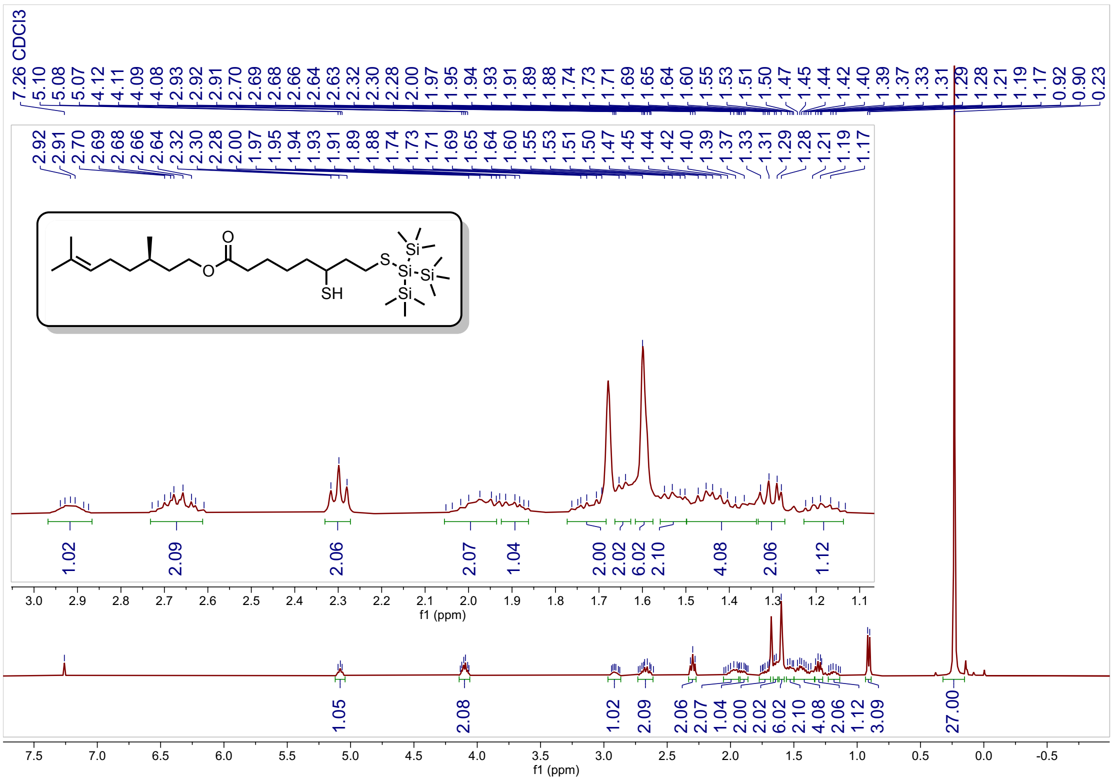

^{13}C NMR of compound 2z10 (100 MHz, $CDCl_3$)

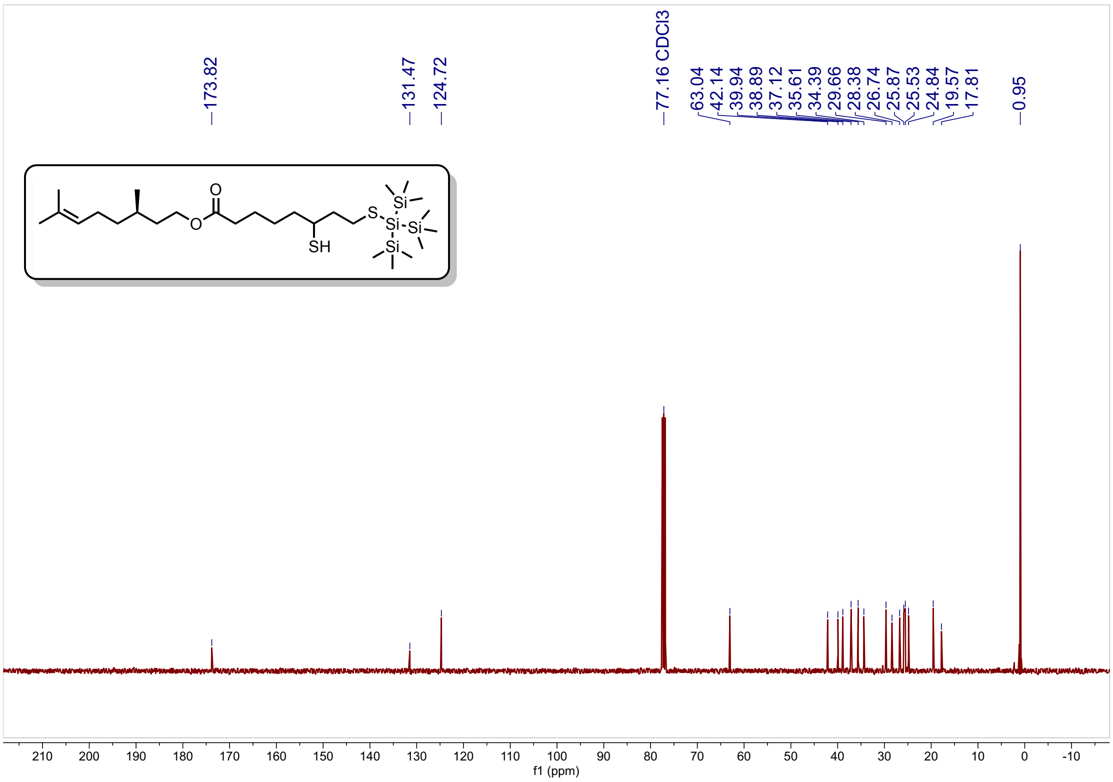

^{29}Si NMR of compound 2z10 (79 MHz, $CDCl_3$)

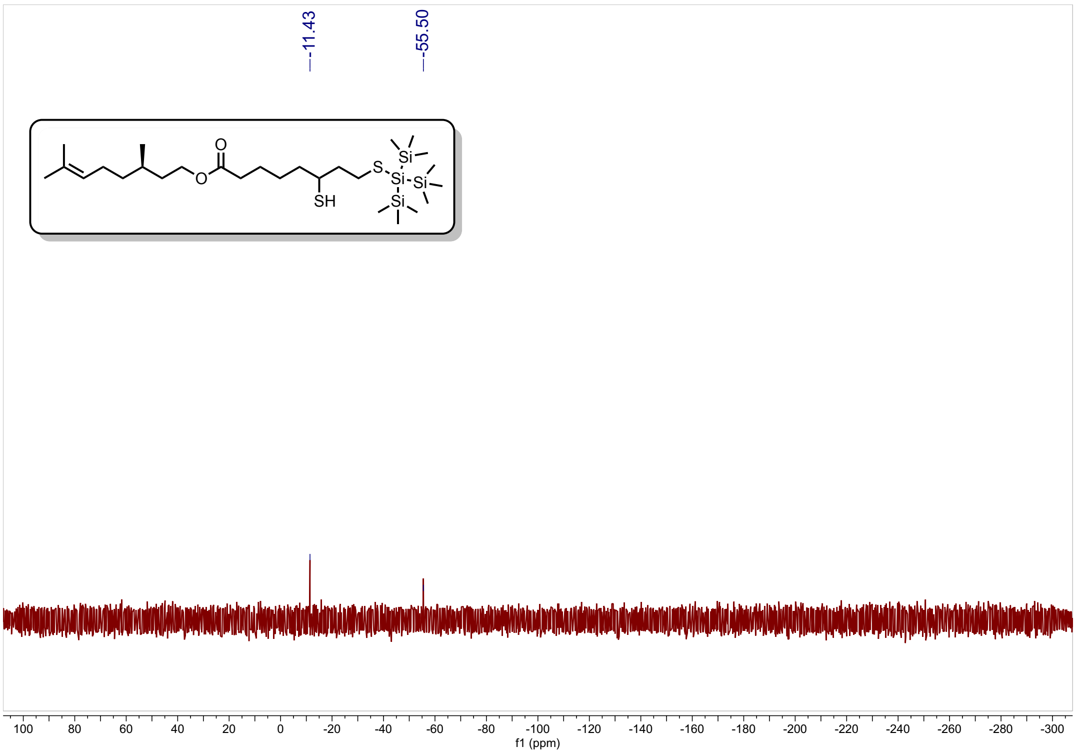

^{1}H NMR of compound 2z11 (600 MHz, $CDCl_3$)

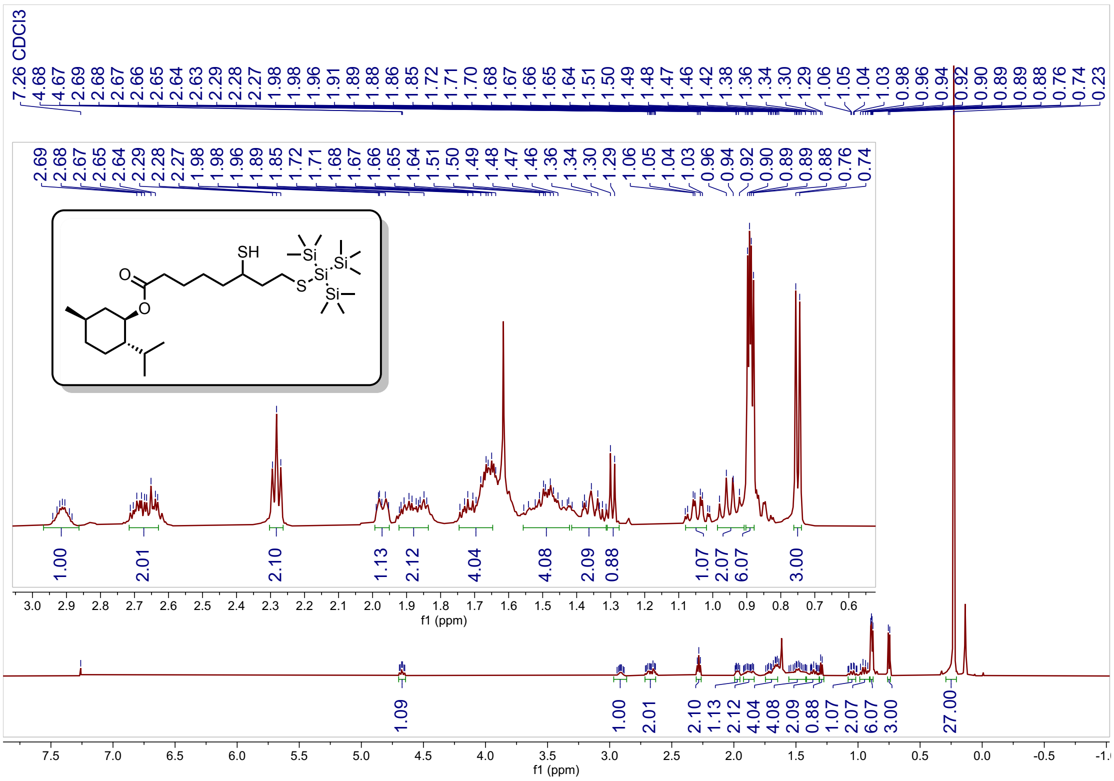

^{13}C NMR of compound 2z11 (100 MHz, $CDCl_3$)

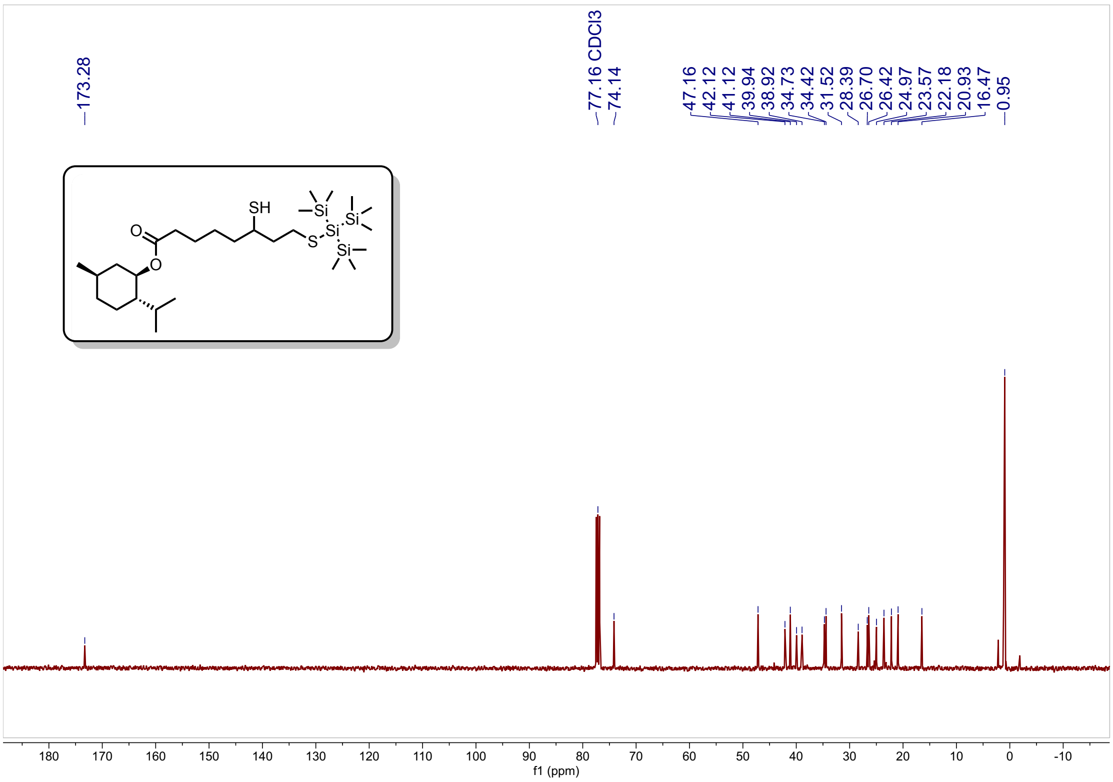

^{29}Si NMR of compound 2z11 (79 MHz, $CDCl_3$)

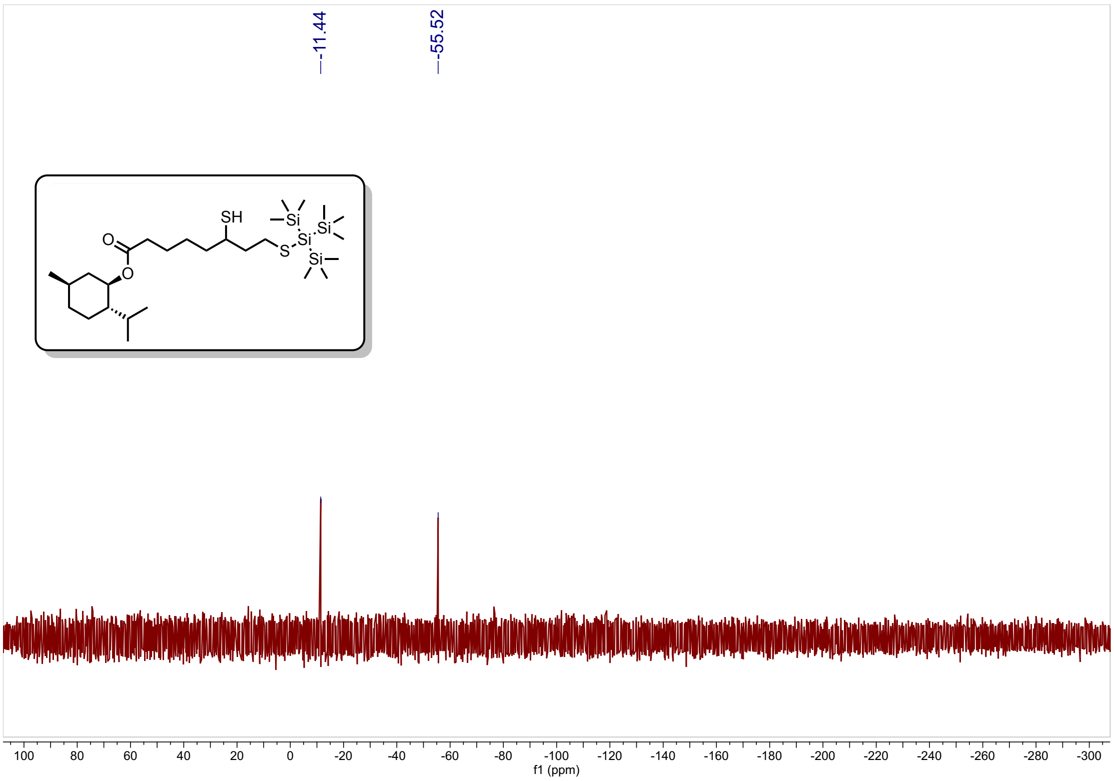

^{1}H NMR of compound 2z12 (400 MHz, $CDCl_3$)

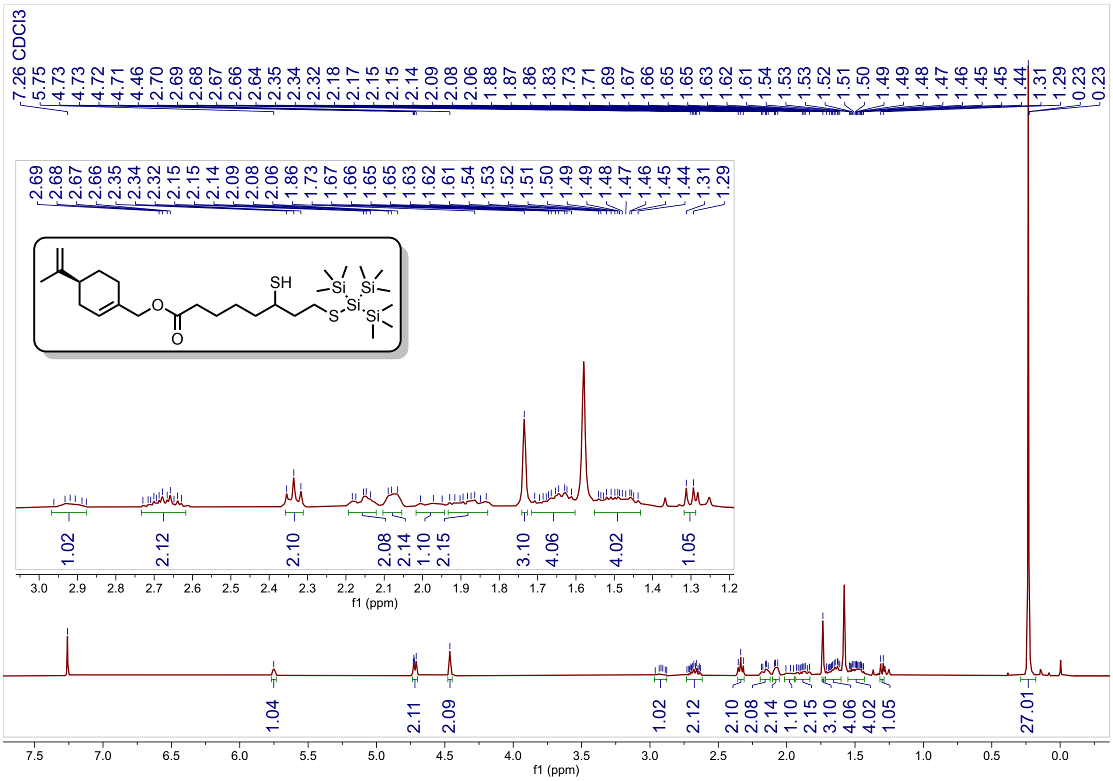

^{13}C NMR of compound 2z12 (100 MHz, $CDCl_3$)

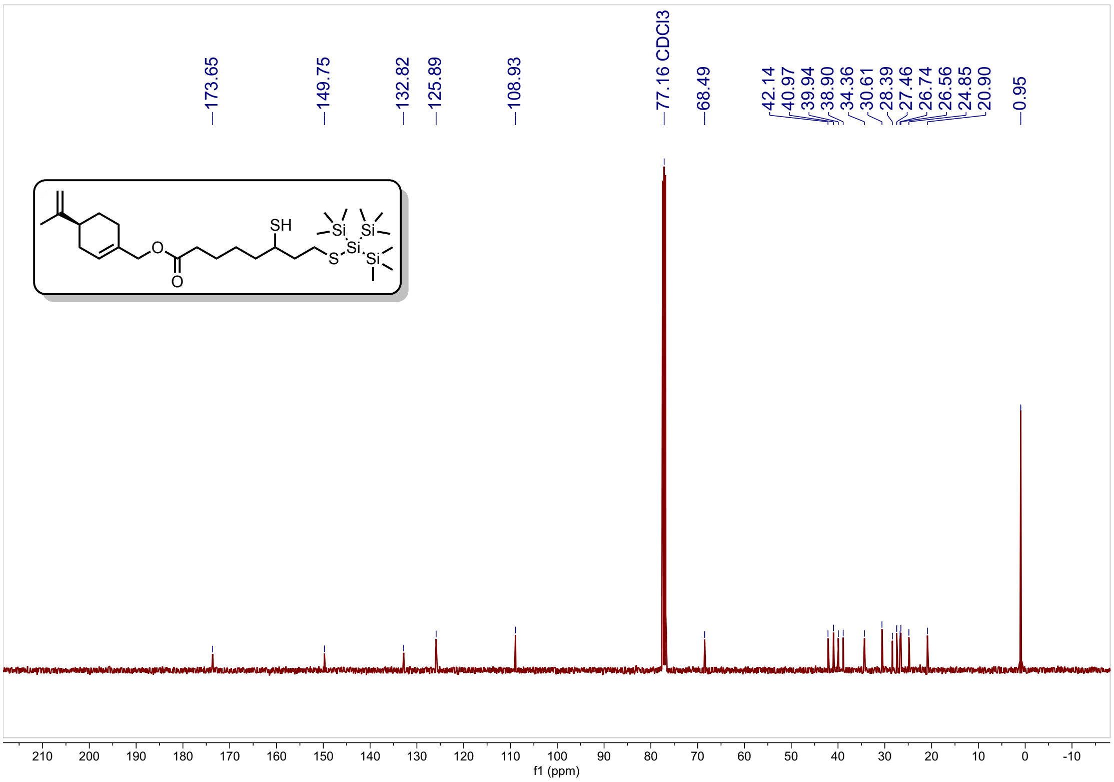

^{29}Si NMR of compound 2z12 (119 MHz, $CDCl_3$)

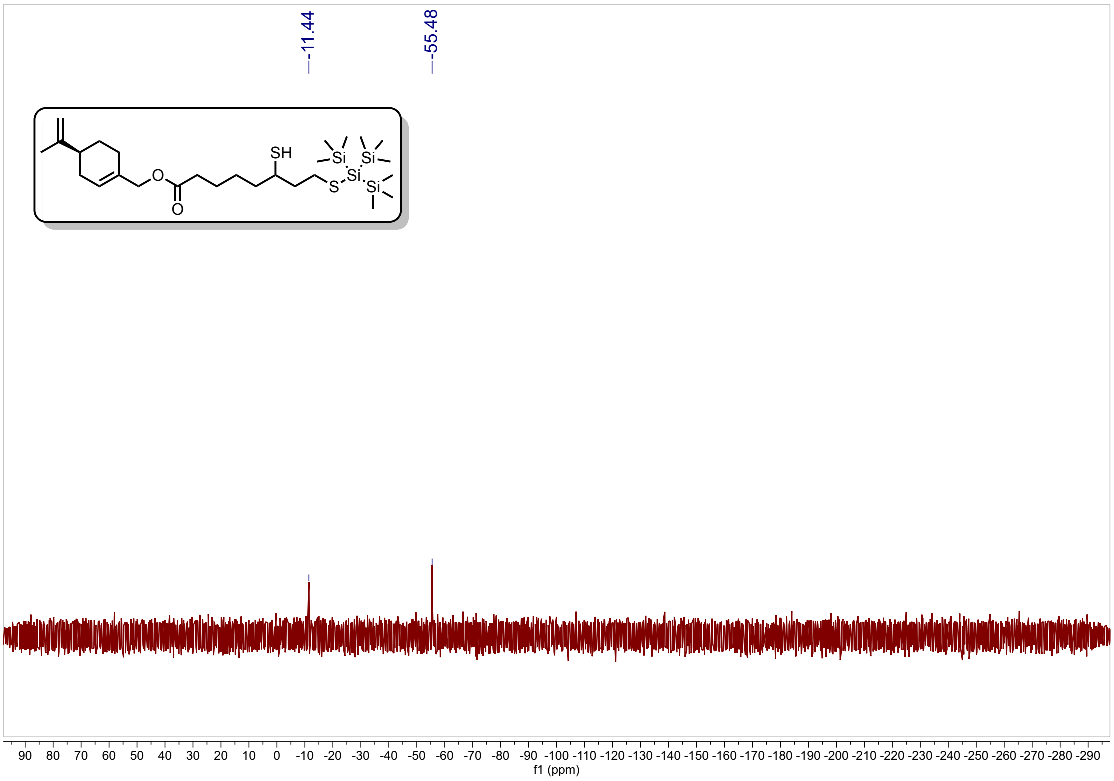

^{1}H NMR of compound 2z13 (400 MHz, $CDCl_3$)

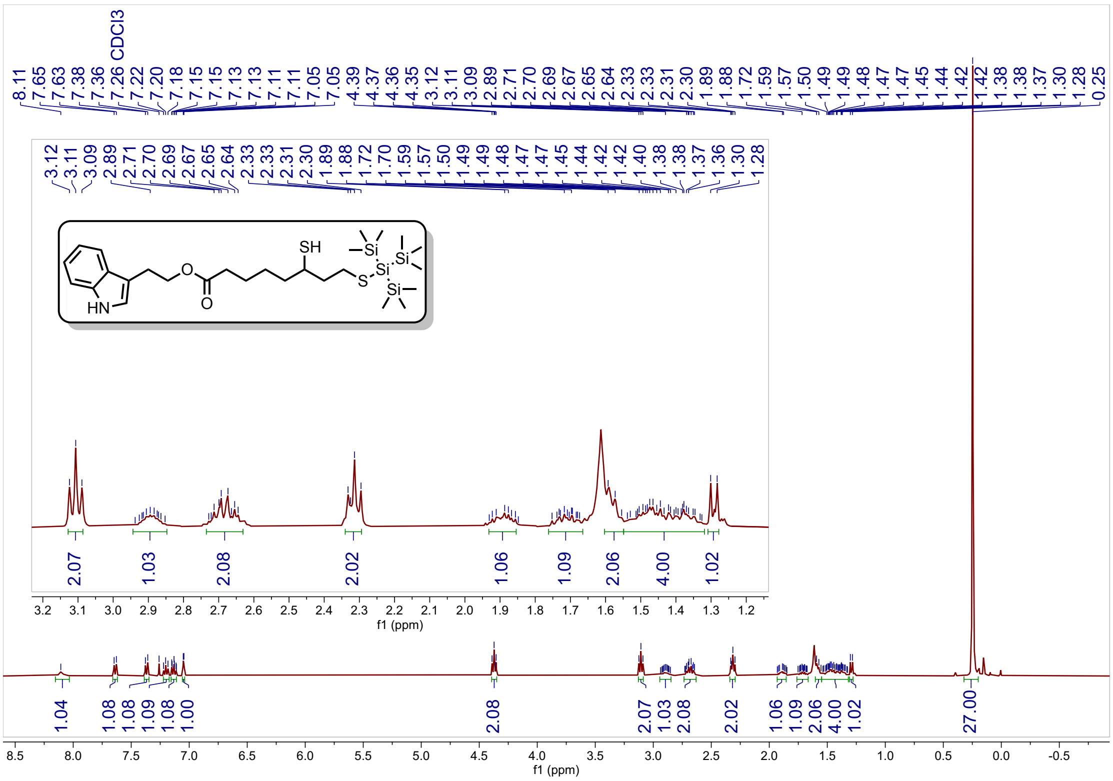

^{13}C NMR of compound 2z13 (100 MHz, $CDCl_3$)

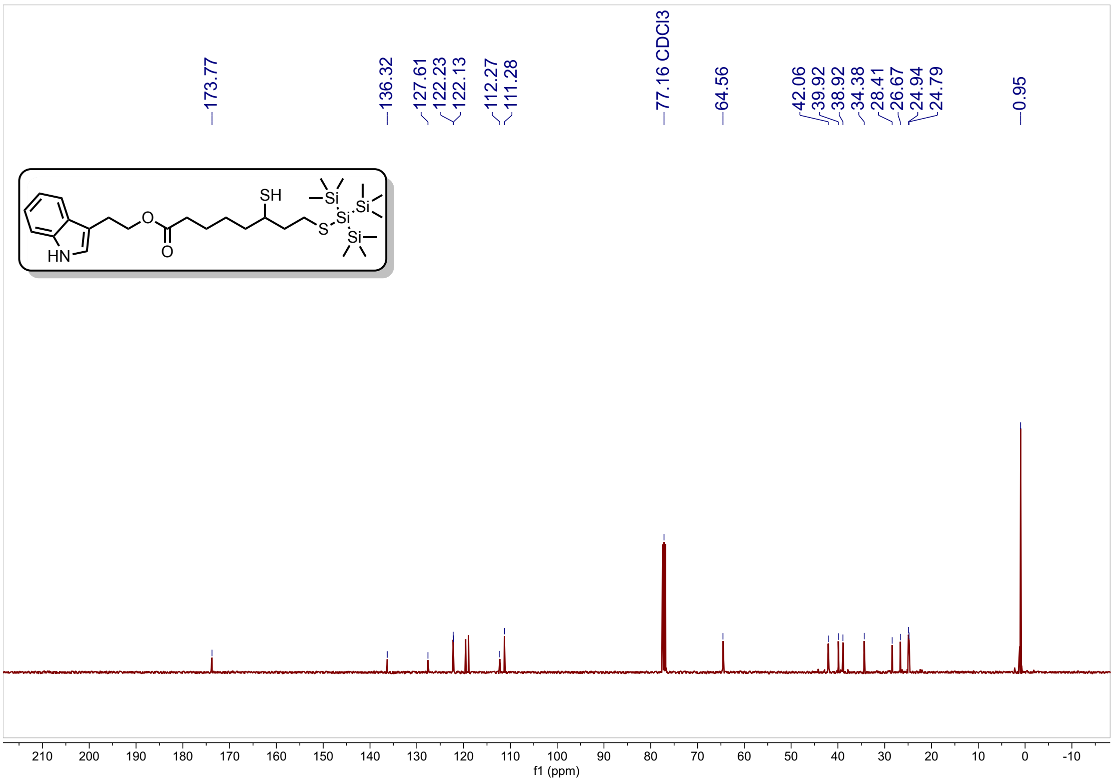

^{29}Si NMR of compound 2z13 (79 MHz, $CDCl_3$)

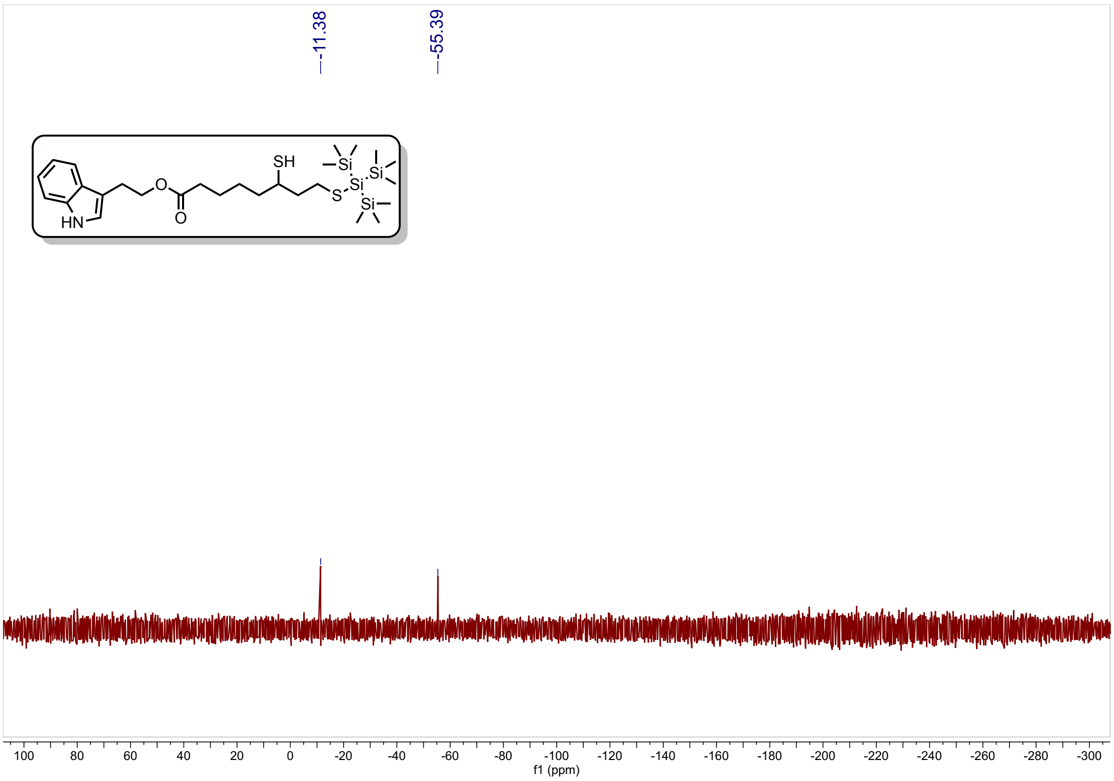

^{1}H NMR of compound 2z14 (400 MHz, $CDCl_3$)

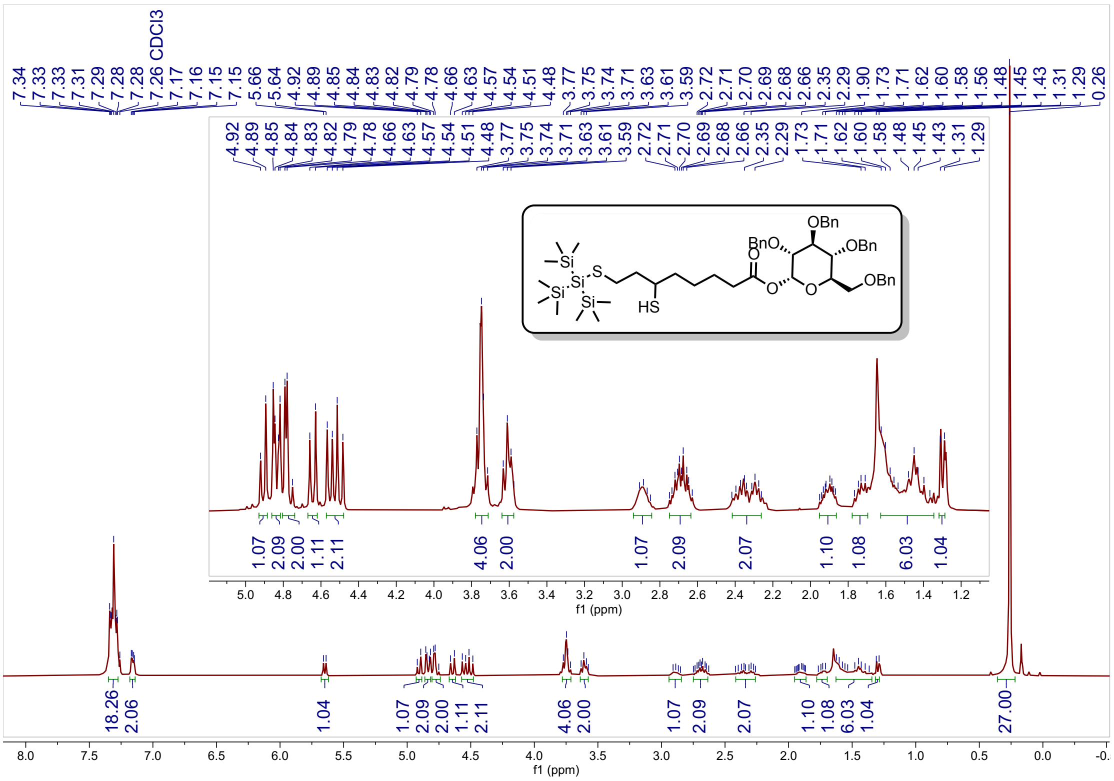

^{13}C NMR of compound 2z14 (100 MHz, $CDCl_3$)

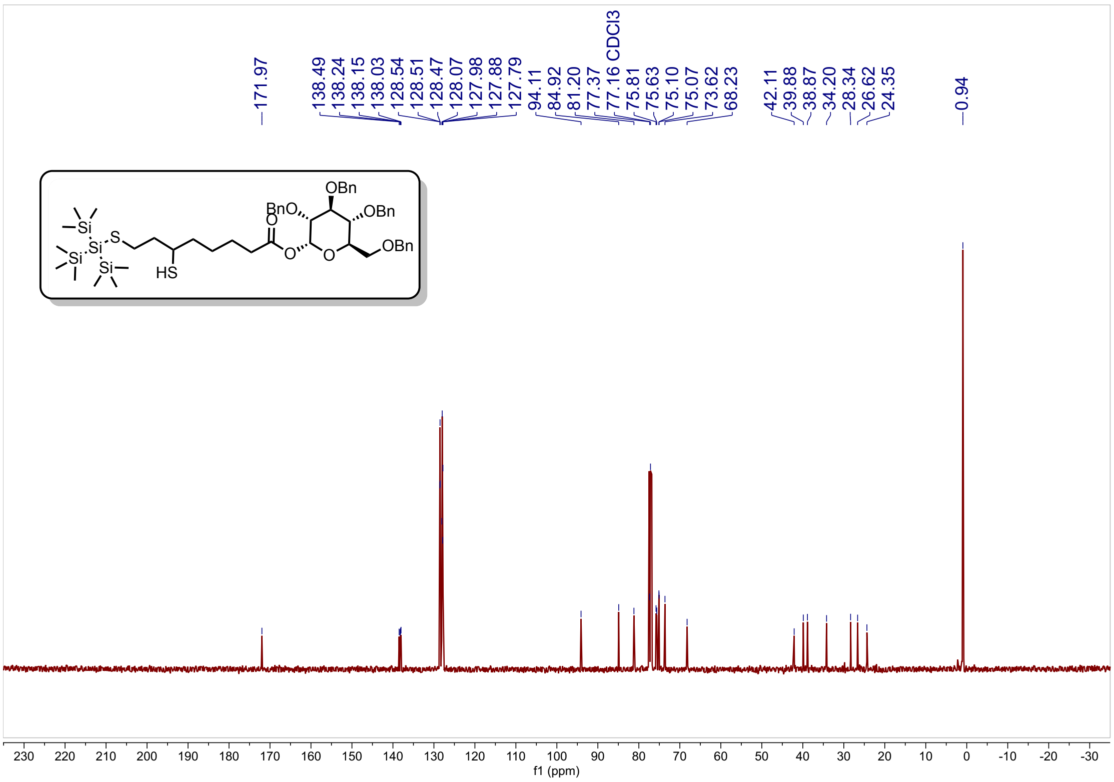

^{29}Si NMR of compound 2z14 (79 MHz, $CDCl_3$)

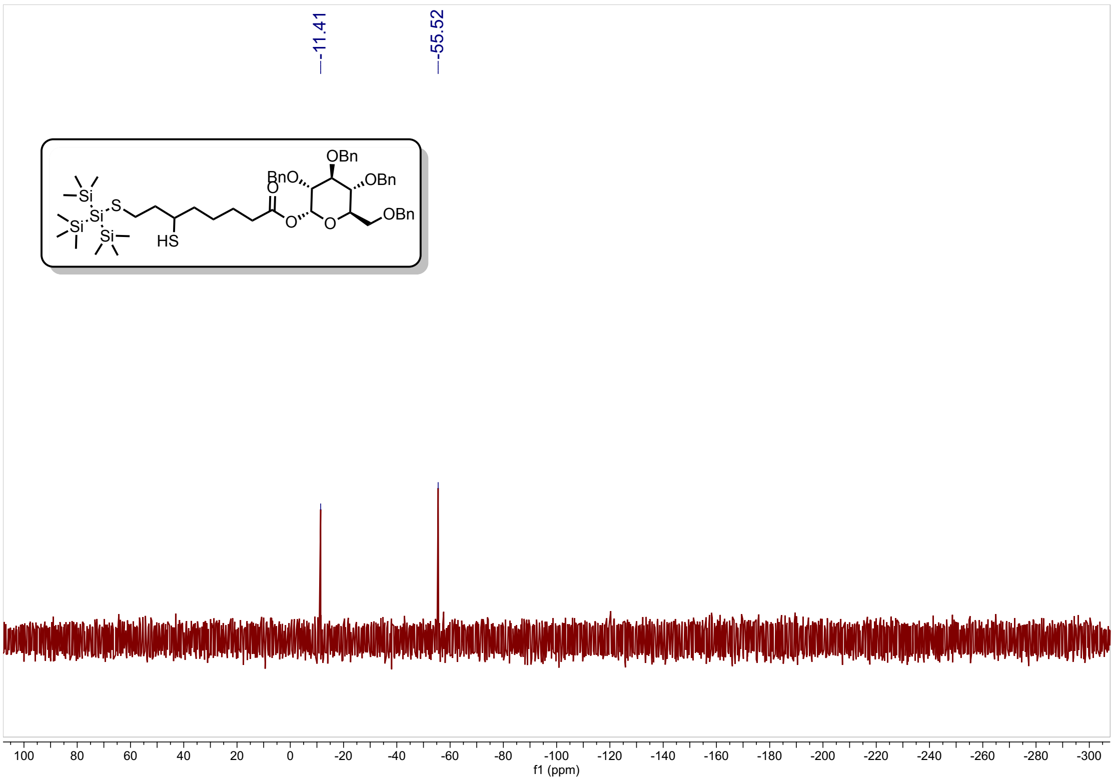

^{1}H NMR of compound 2z15 (400 MHz, $CDCl_3$)

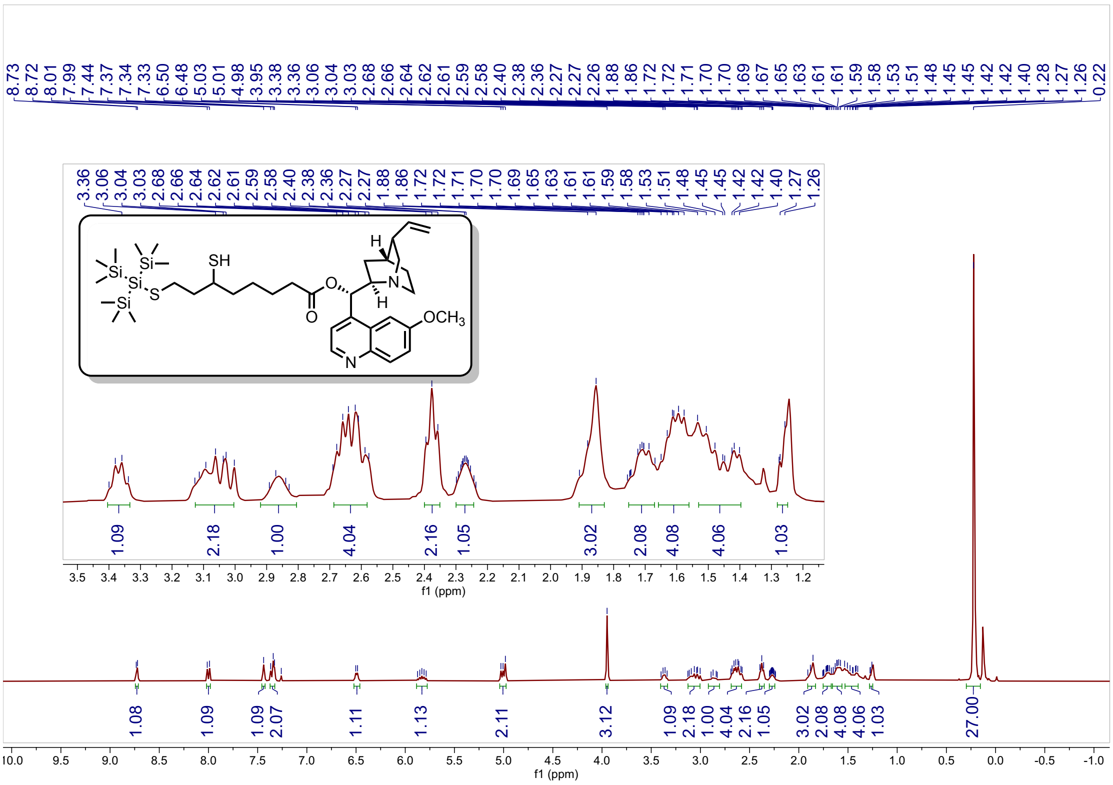

^{13}C NMR of compound 2z15 (100 MHz, $CDCl_3$)

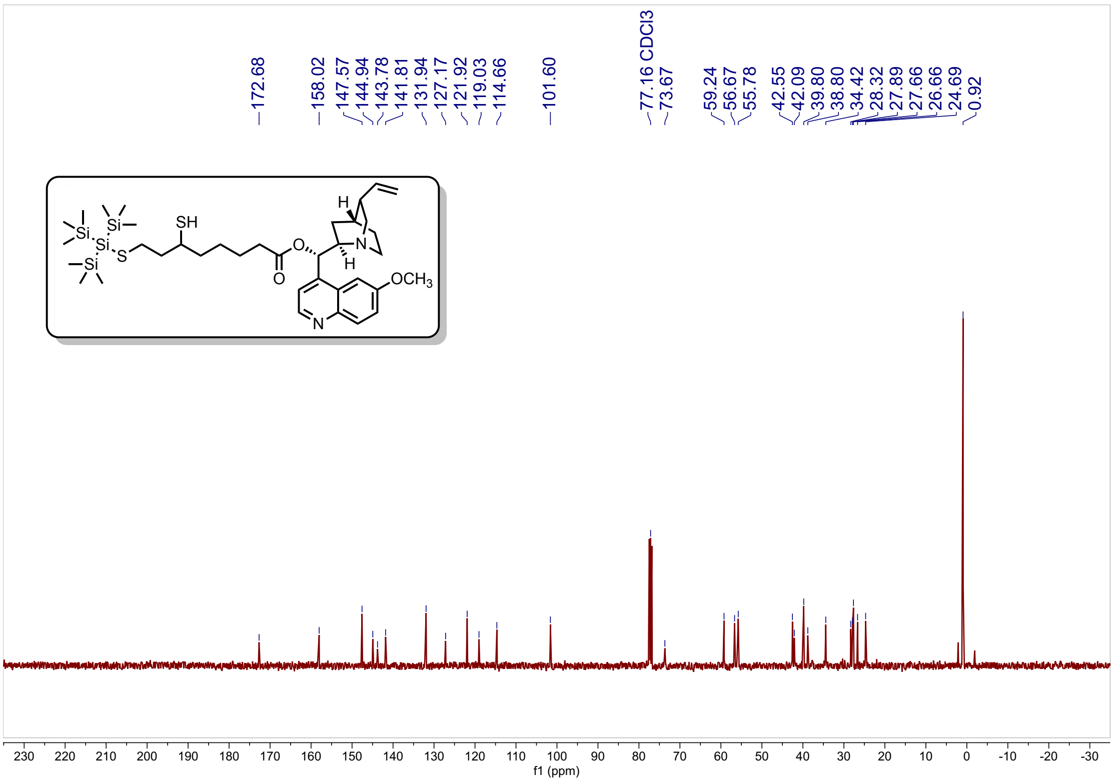

^{29}Si NMR of compound 2z15 (79 MHz, CDCl$_3$)

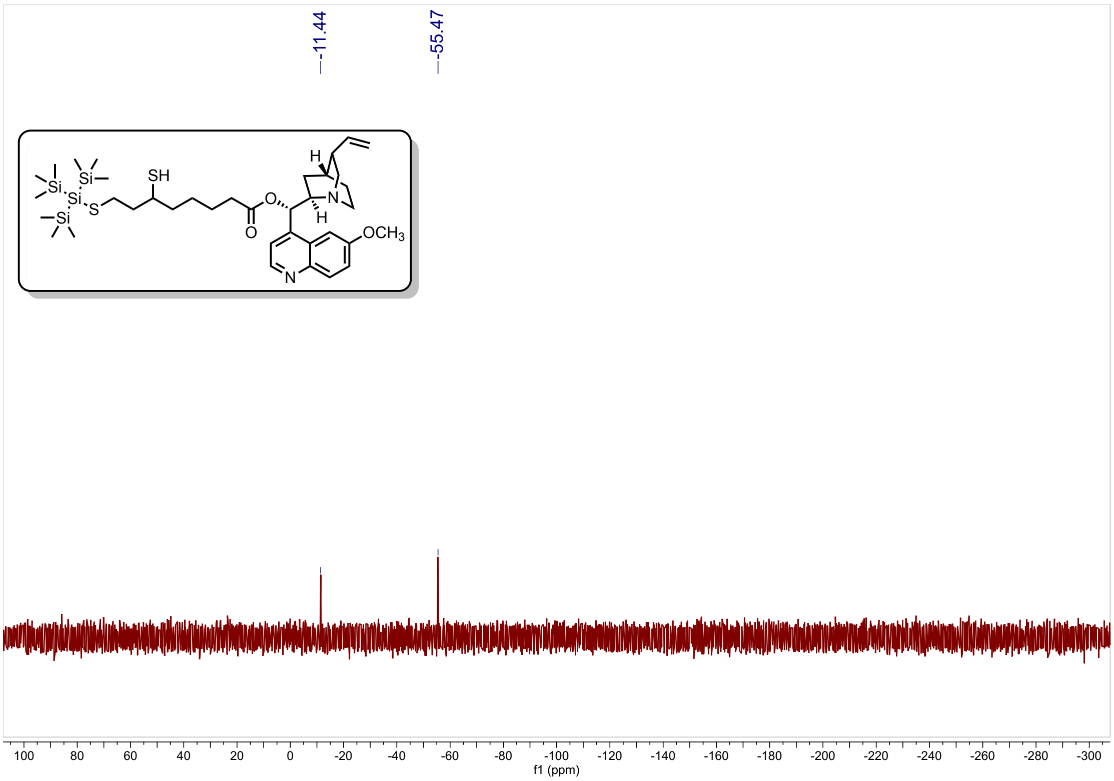

^{1}H NMR of compound 2z16 (400 MHz, CDCl$_3$)

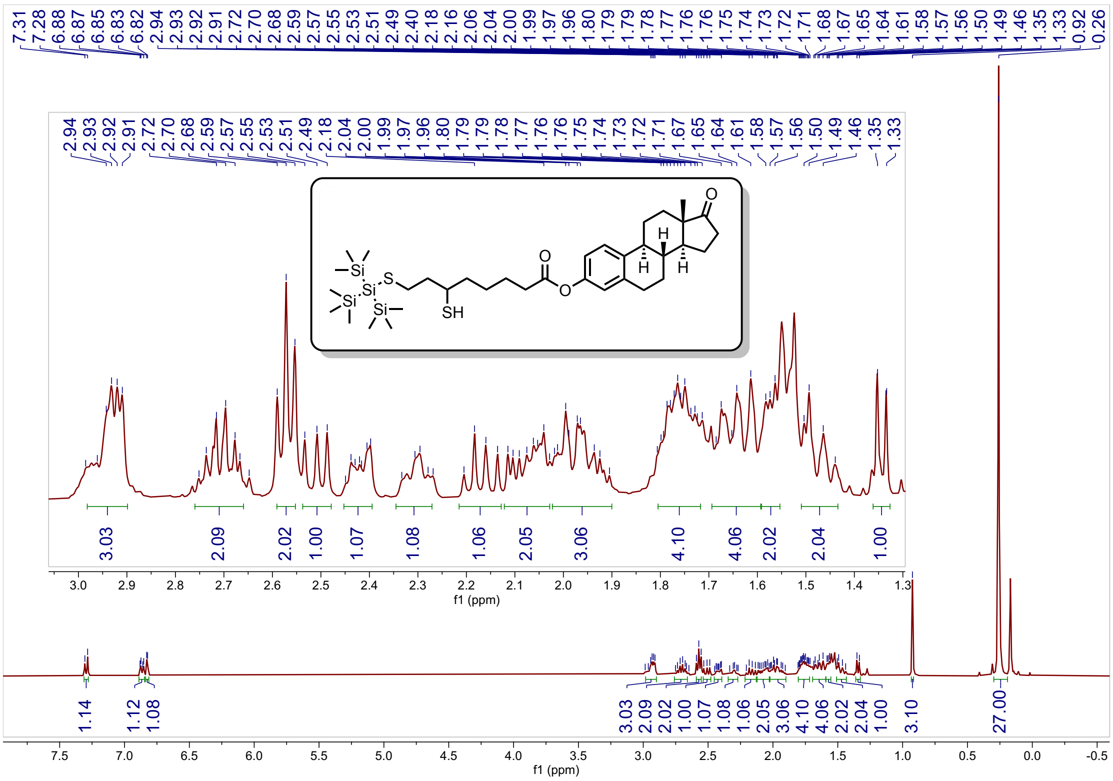

^{1}H NMR of compound 2z16 (400 MHz, CDCl$_3$)

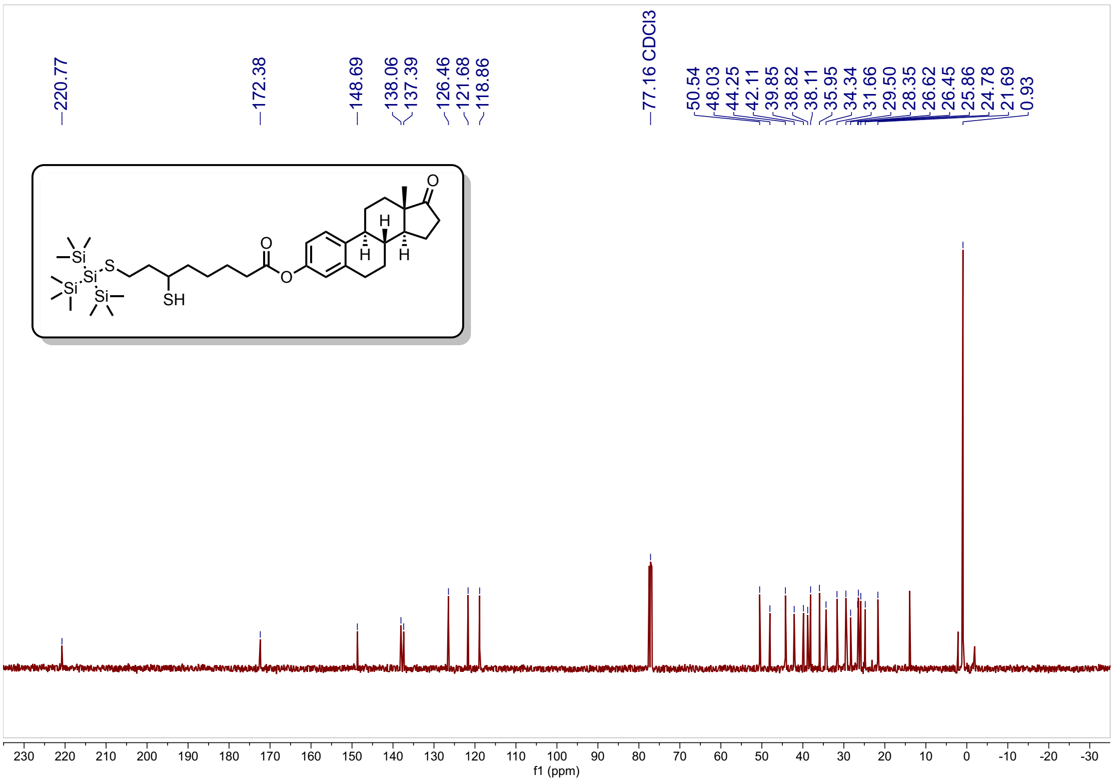

^{29}Si NMR of compound 2z16 (79 MHz, CDCl$_3$)

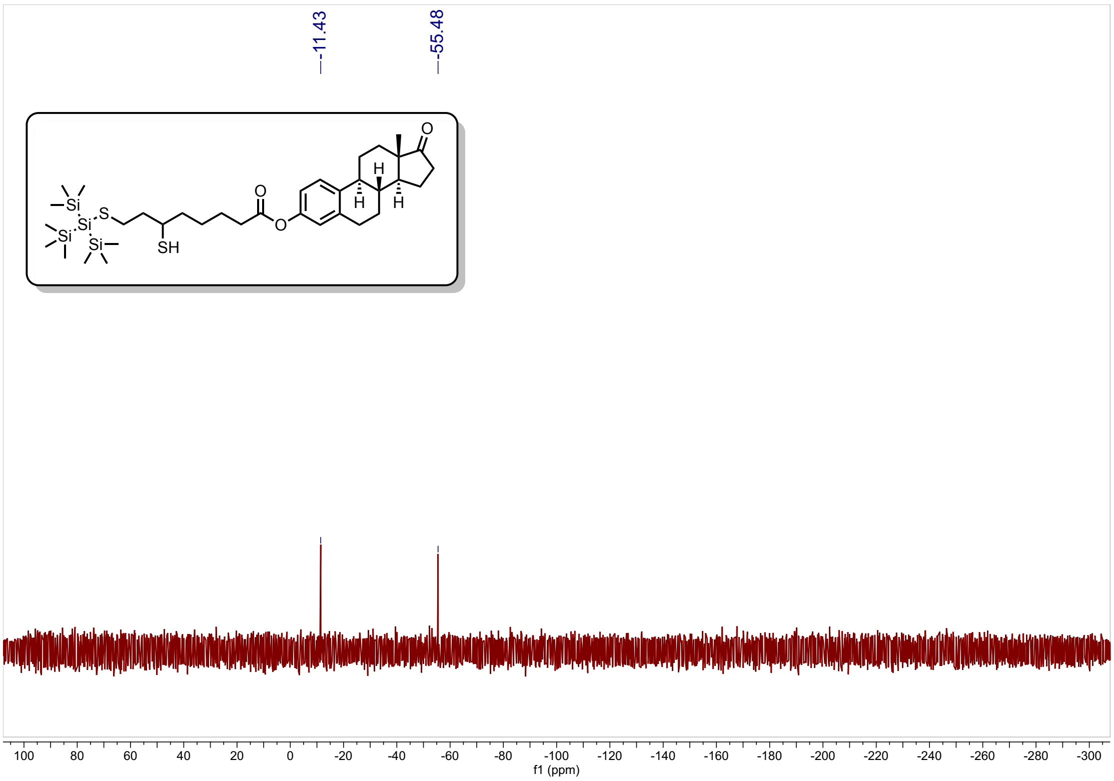

^{1}H NMR of compound 2z17 (600 MHz, $CDCl_3$)

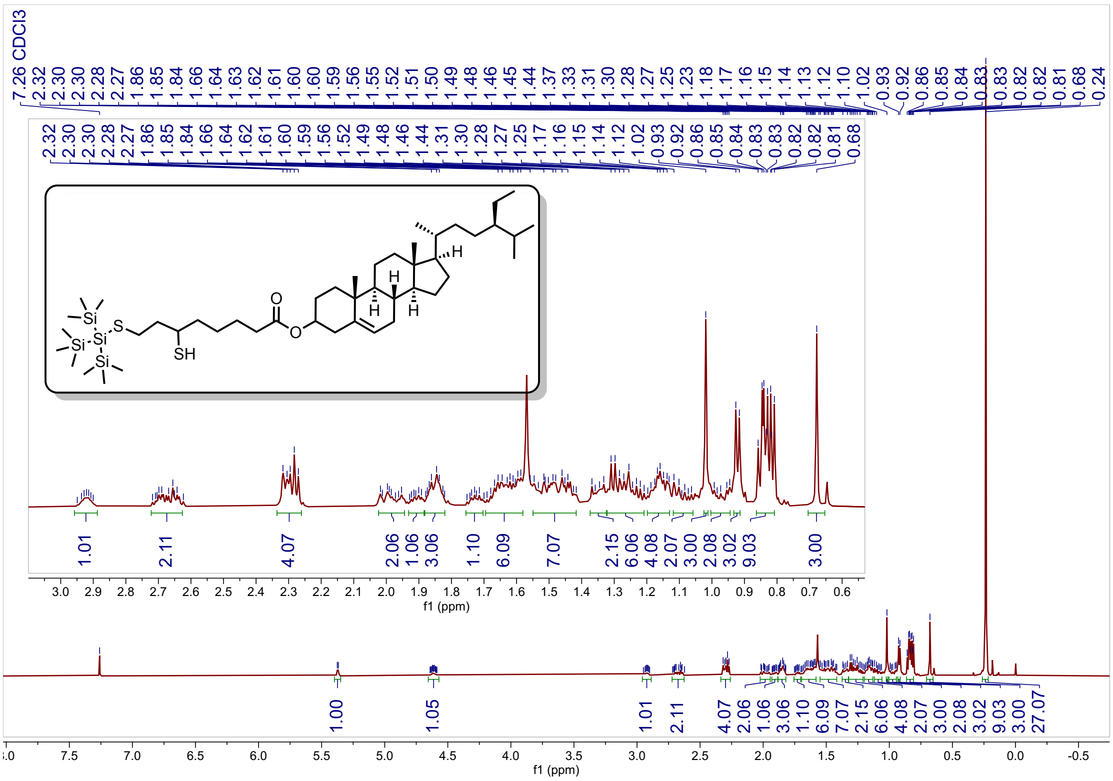

^{13}C NMR of compound 2z17 (100 MHz, $CDCl_3$)

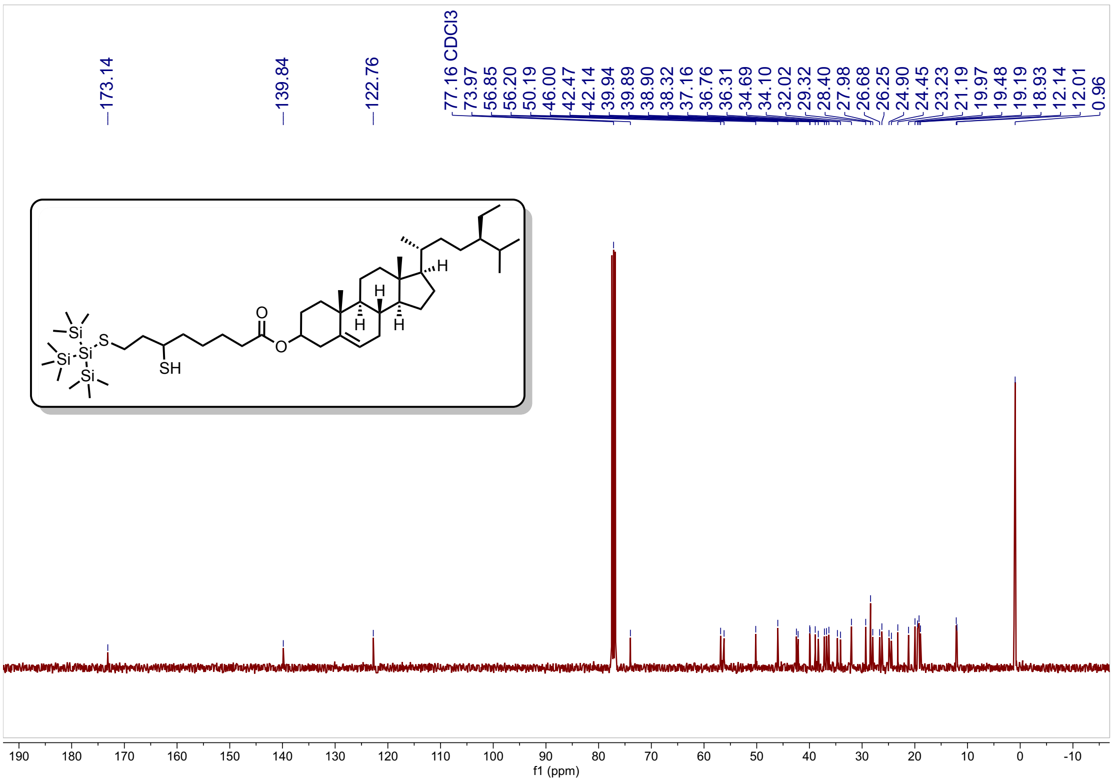

^{29}Si NMR of compound 2z17 (79 MHz, CDCl$_3$)

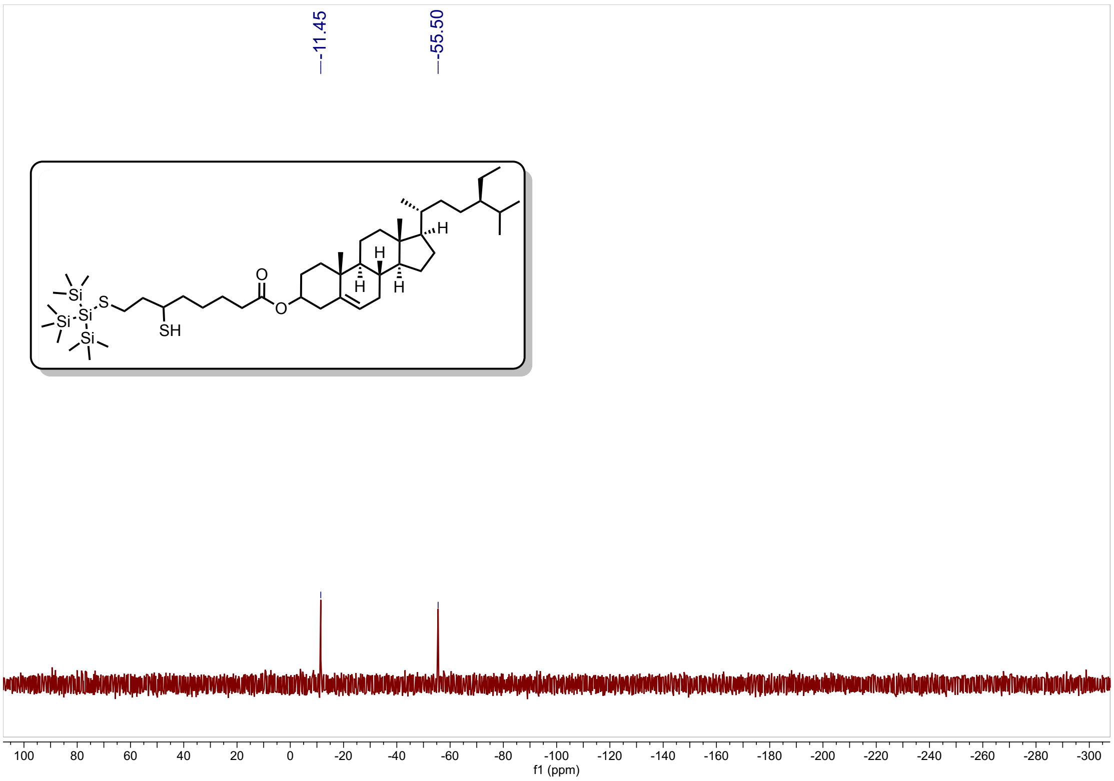

^{1}H NMR of compound 2z19 (400 MHz, $CDCl_3$)

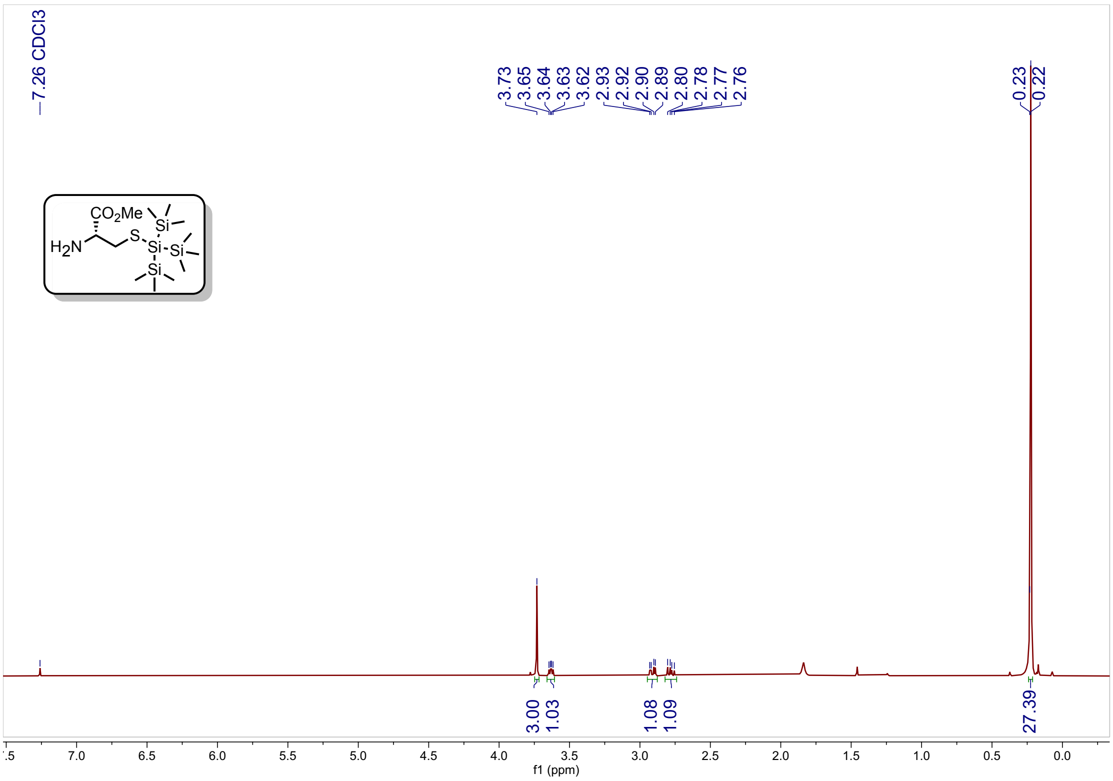

^{13}C NMR of compound 2z19 (100 MHz, $CDCl_3$)

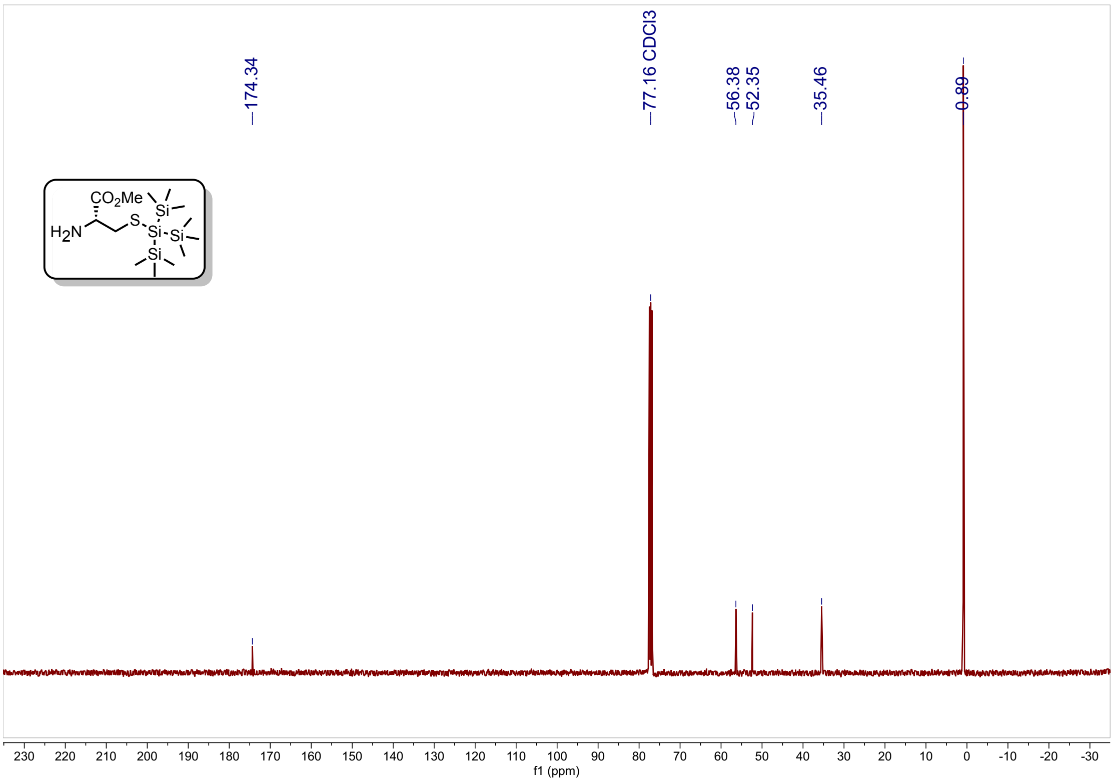

^{29}Si NMR of compound 2z19 (79 MHz, $CDCl_3$)

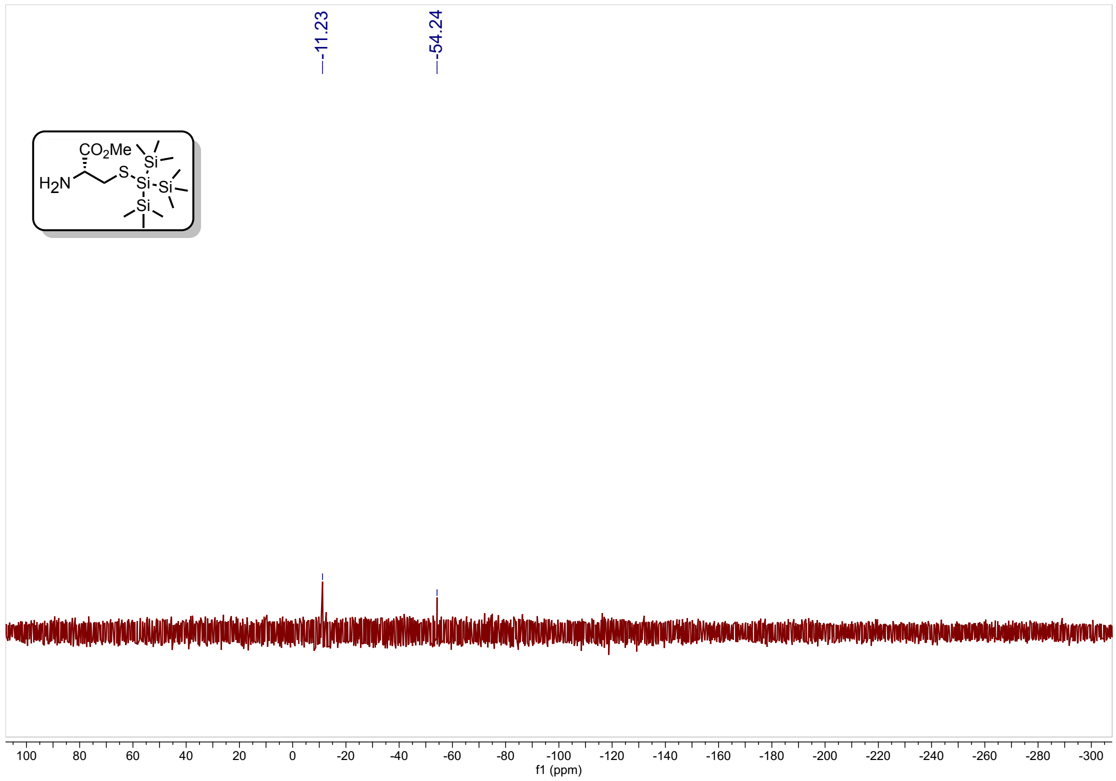

^{1}H NMR of compound 2z20 (600 MHz, $CDCl_3$)

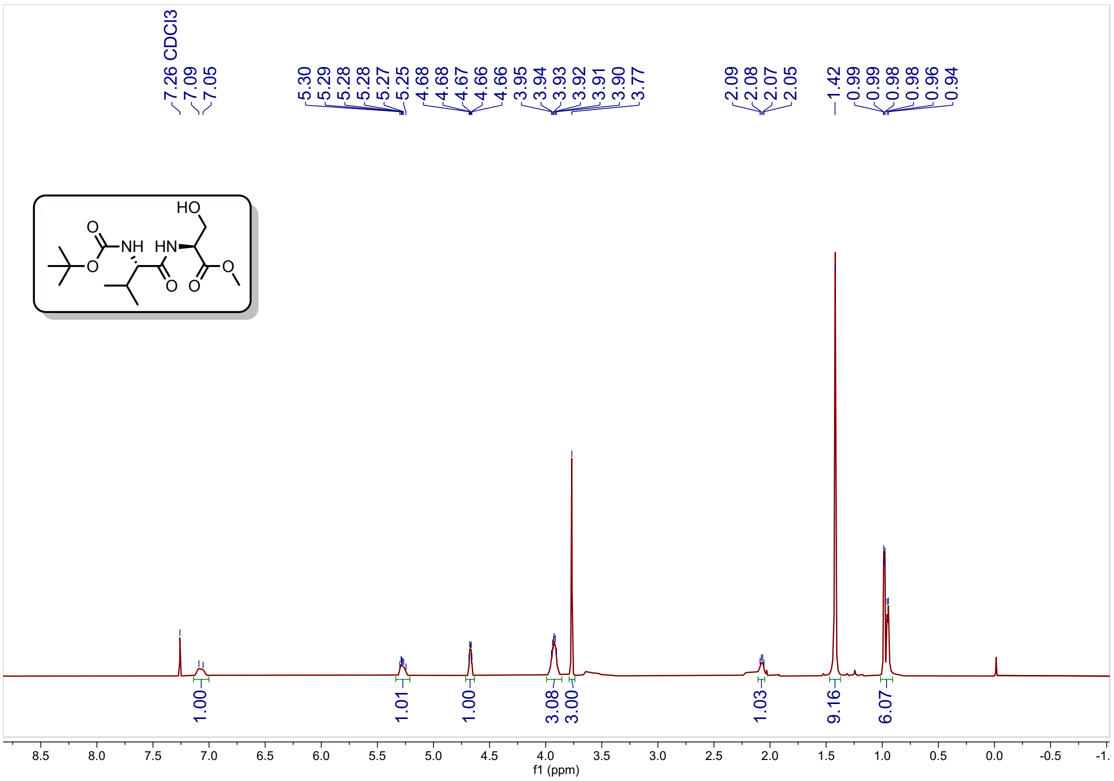

^{13}C NMR of compound 2z20 (100 MHz, $CDCl_3$)

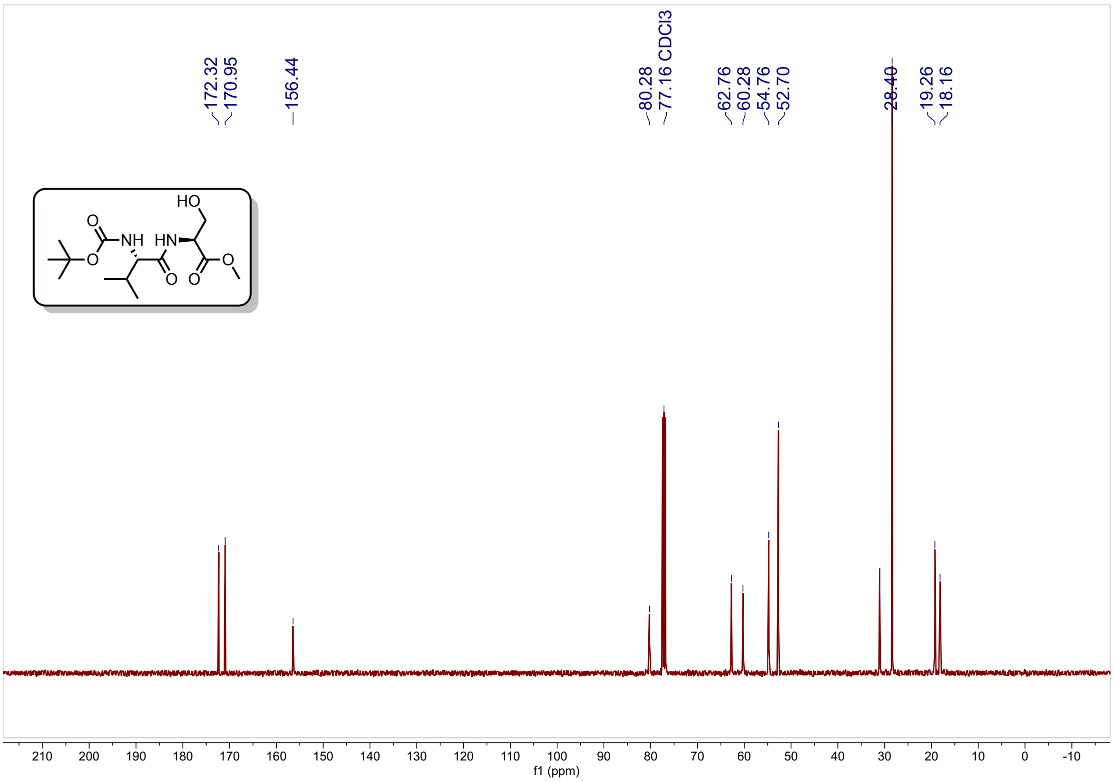

^{1}H NMR of compound 2z21 (600 MHz, $CDCl_3$)

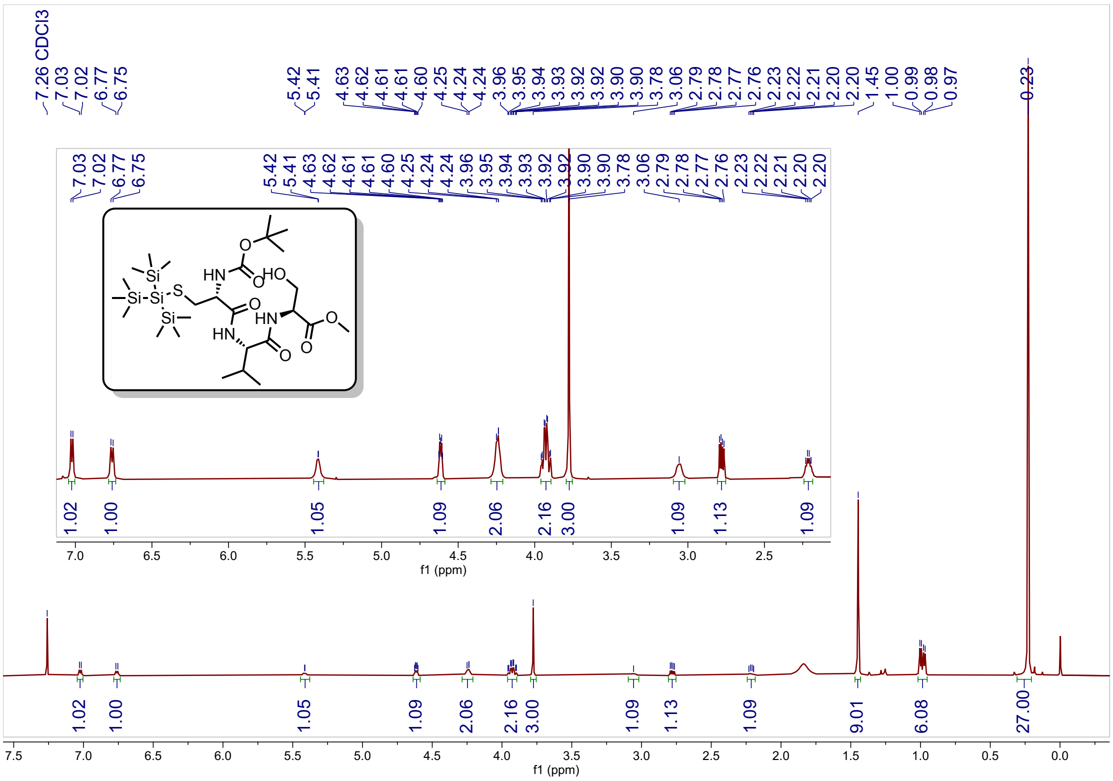

^{13}C NMR of compound 2z21 (100 MHz, $CDCl_3$)

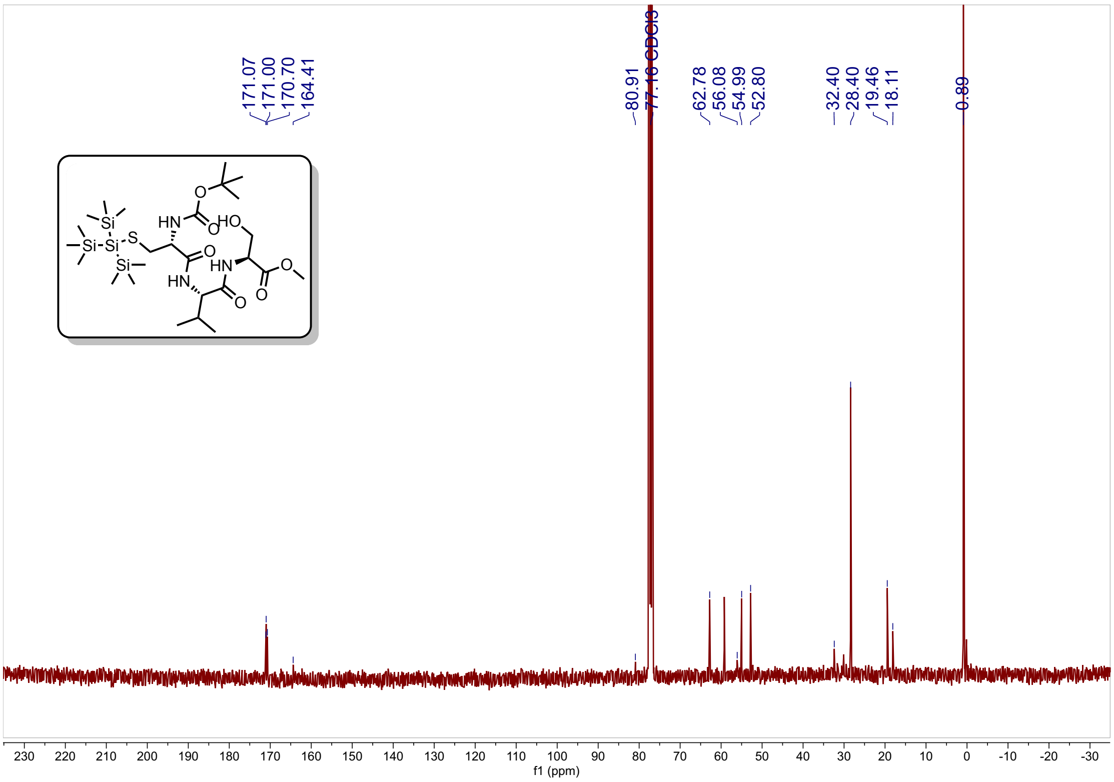

^{1}H NMR of compound 2z22 (600 MHz, $CDCl_3$)

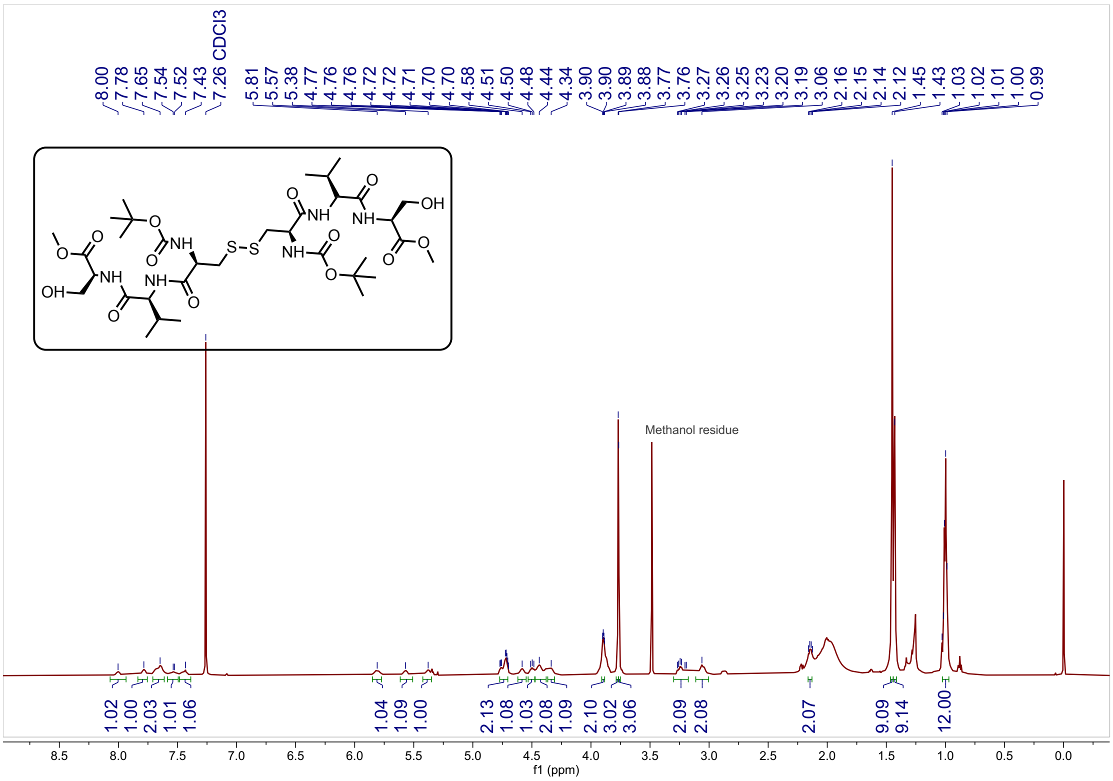

^{1}H NMR of compound 2z23 (400 MHz, $CDCl_3$)

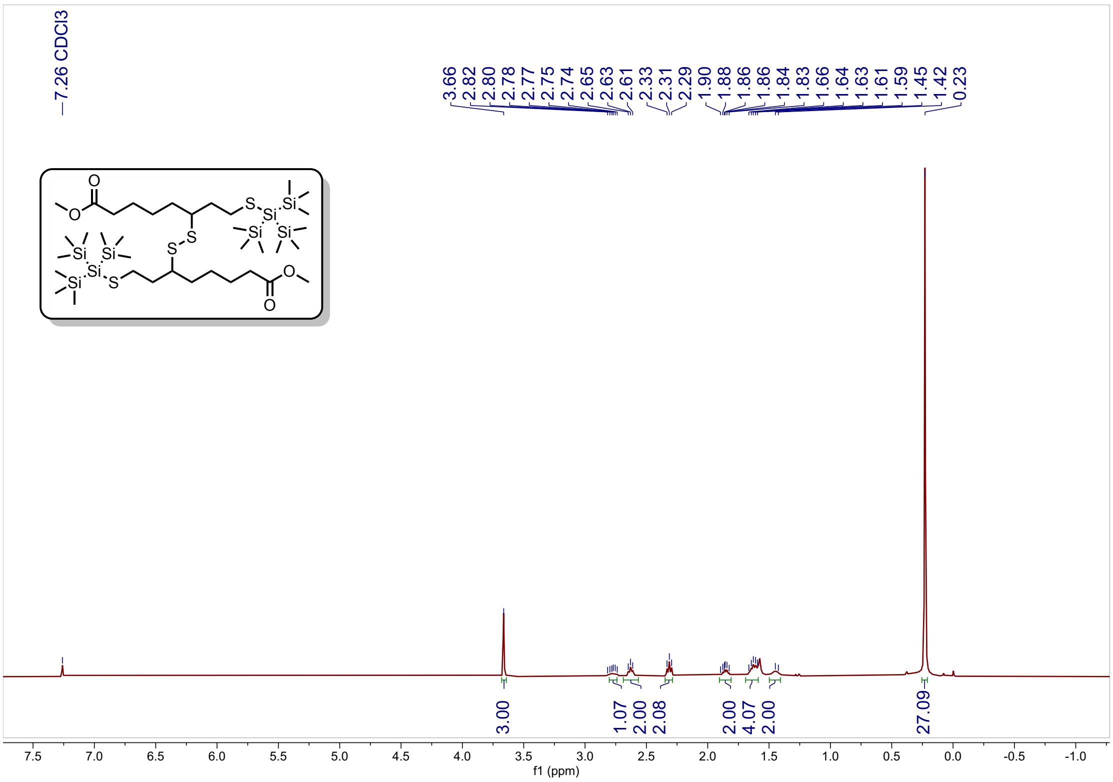

^{13}C NMR of compound 2z23 (100 MHz, CDCl3)

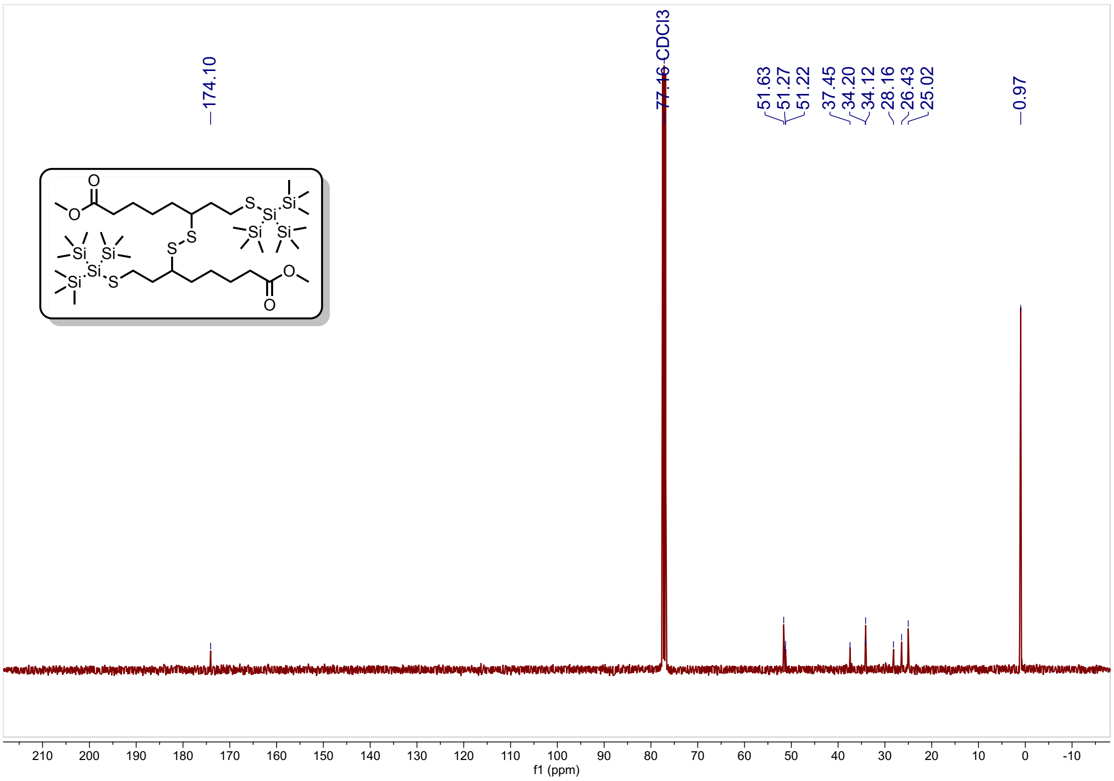

^{29}Si NMR of compound 2z23 (79 MHz, $CDCl_3$)

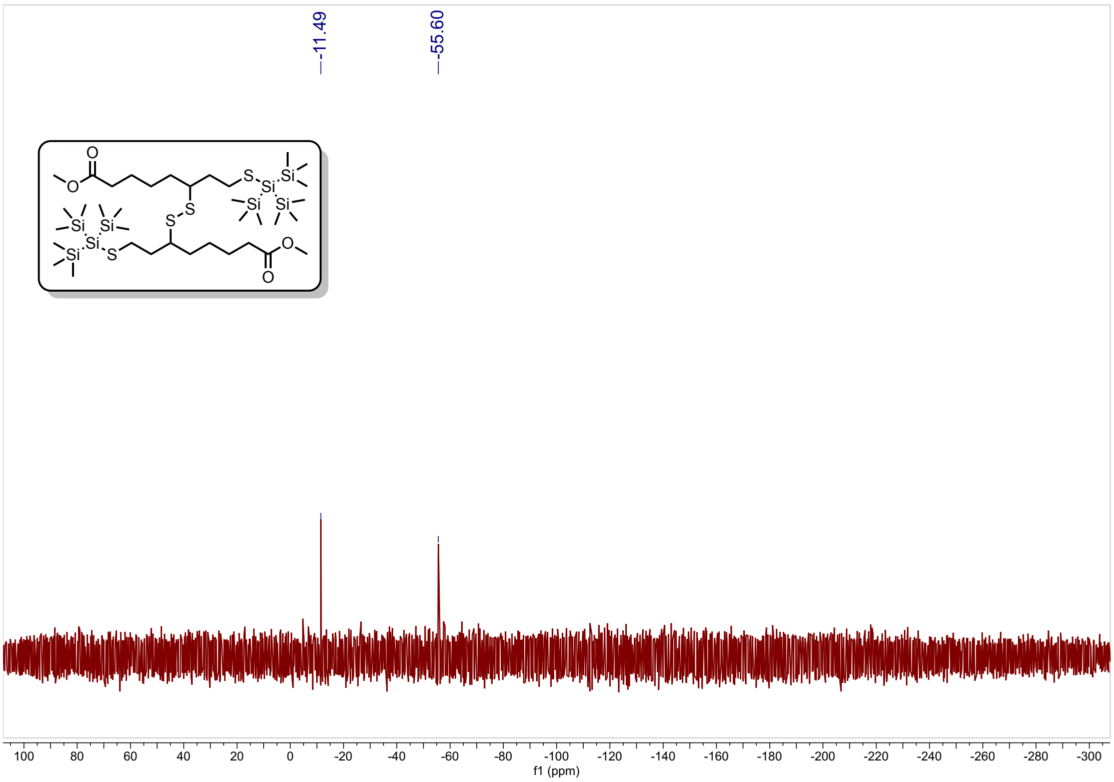

^{1}H NMR of compound 2z24 (400 MHz, $CDCl_3$)

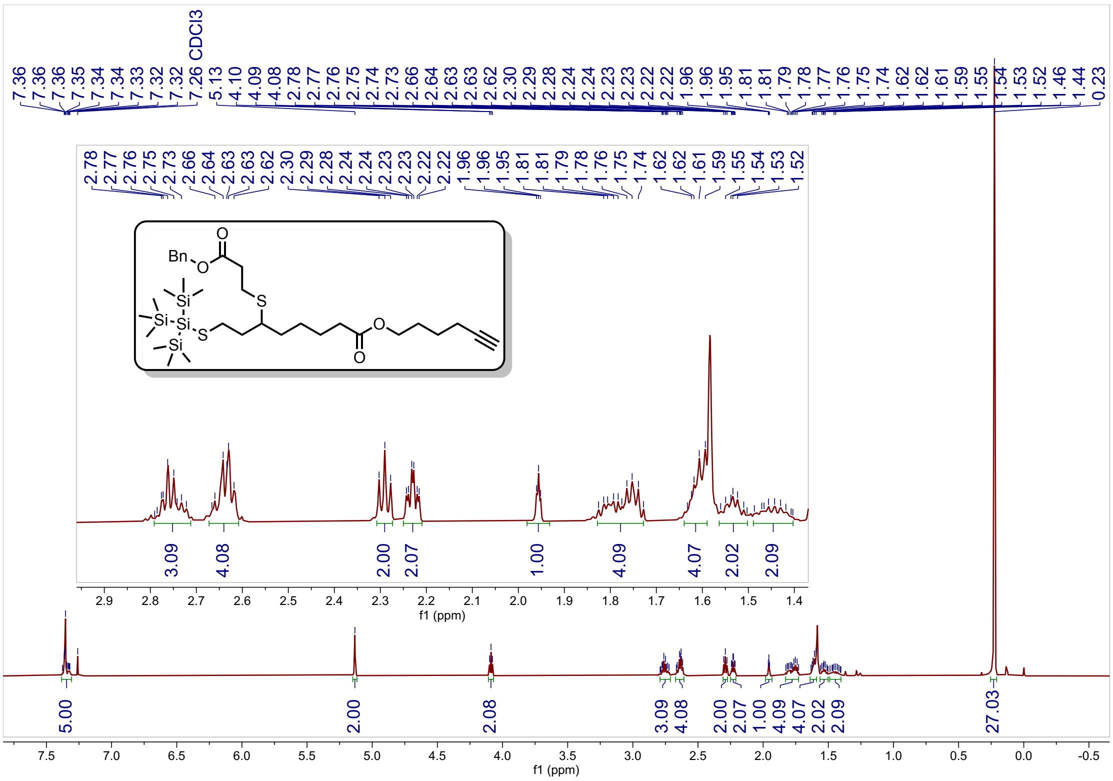

^{13}C NMR of compound 2z24 (100 MHz, $CDCl_3$)

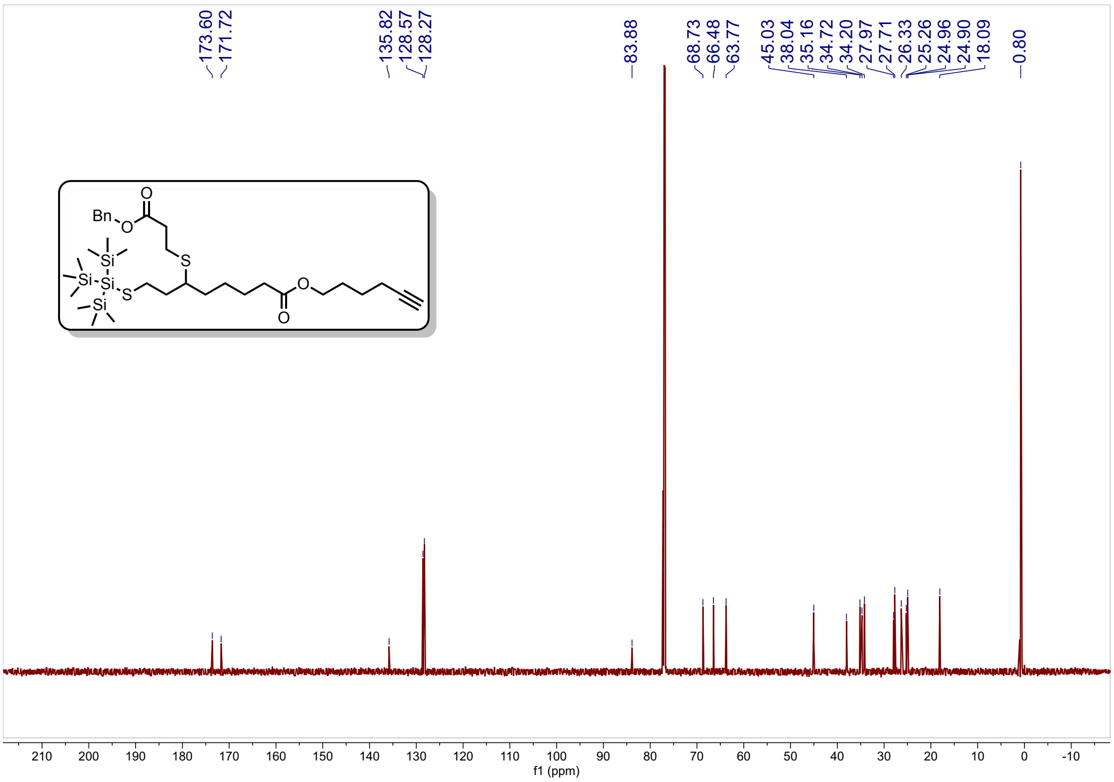

^{29}Si NMR of compound 2z24 (79 MHz, $CDCl_3$)

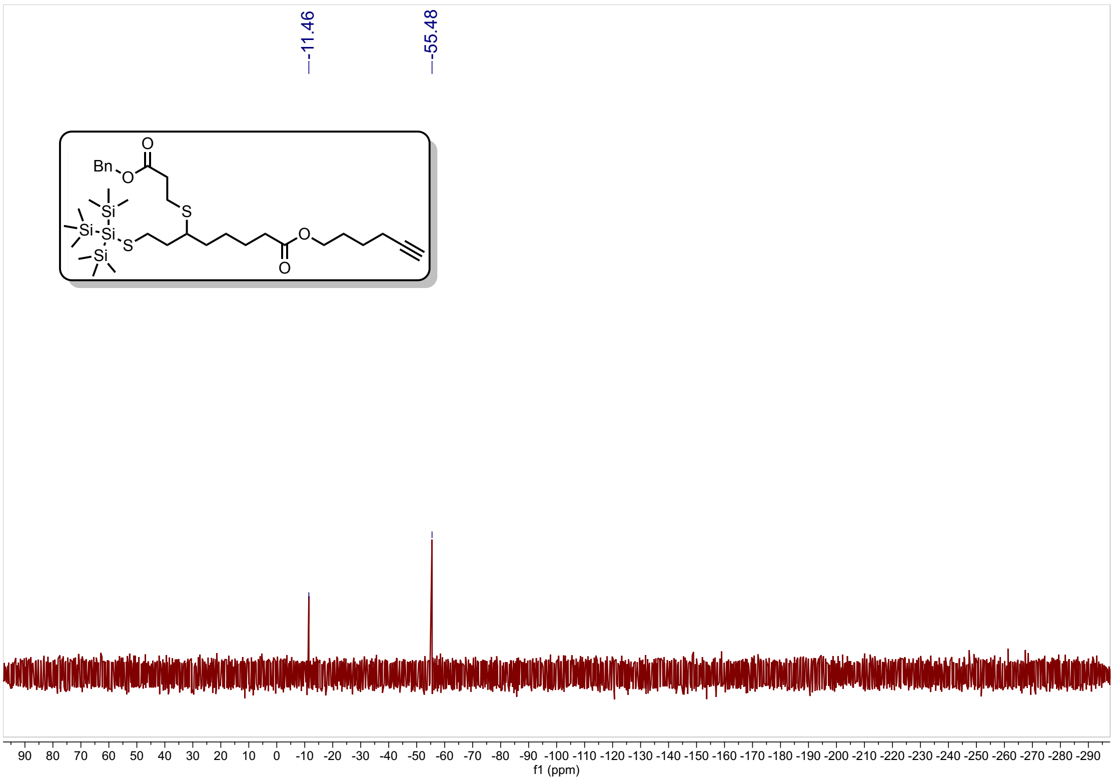

^{1}H NMR of compound 2z25 (400 MHz, CDCl$_3$)

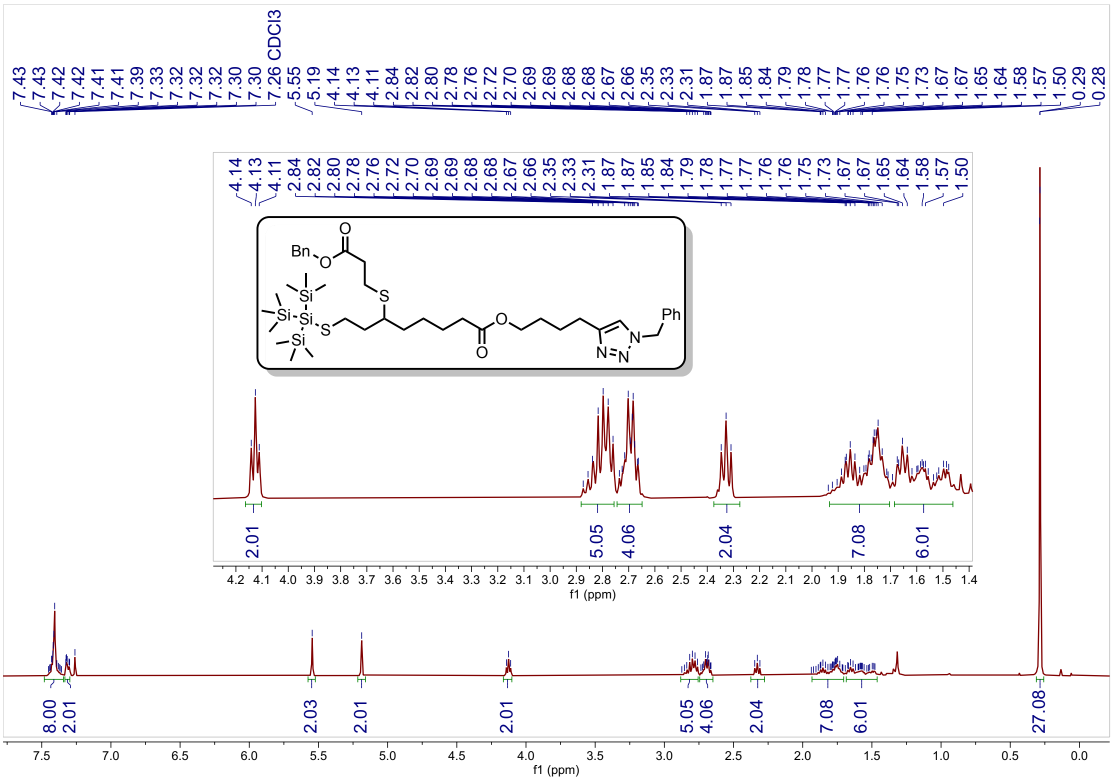

^{13}C NMR of compound 2z25 (100 MHz, CDCl$_3$)

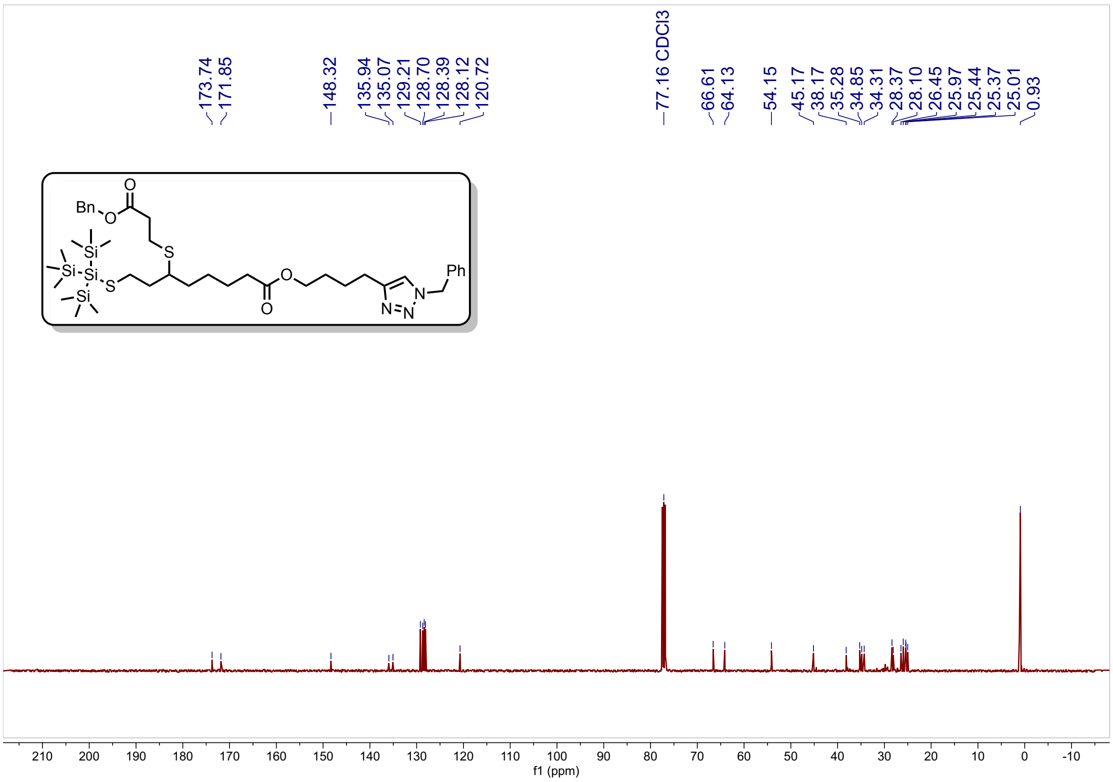

^{29}Si NMR of compound 2z25 (79 MHz, $CDCl_3$)

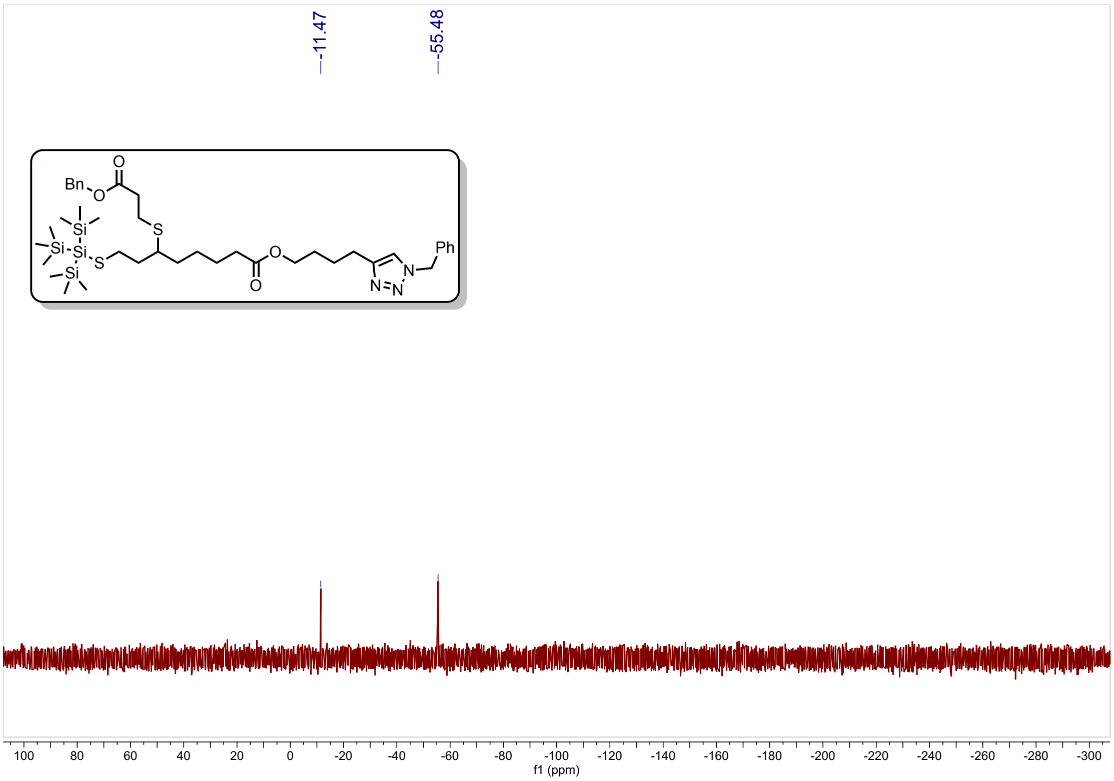

^{1}H NMR of compound 2z26 (400 MHz, $CDCl_3$)

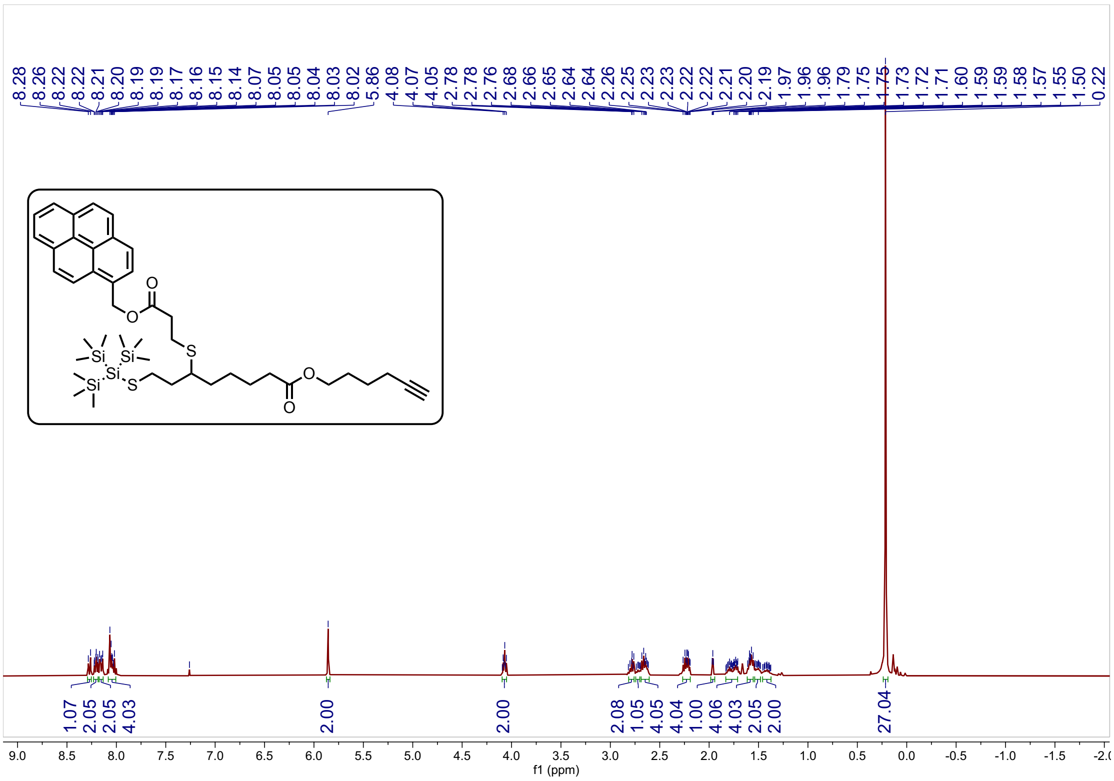

^{13}C NMR of compound 2z26 (100 MHz, CDCl3)

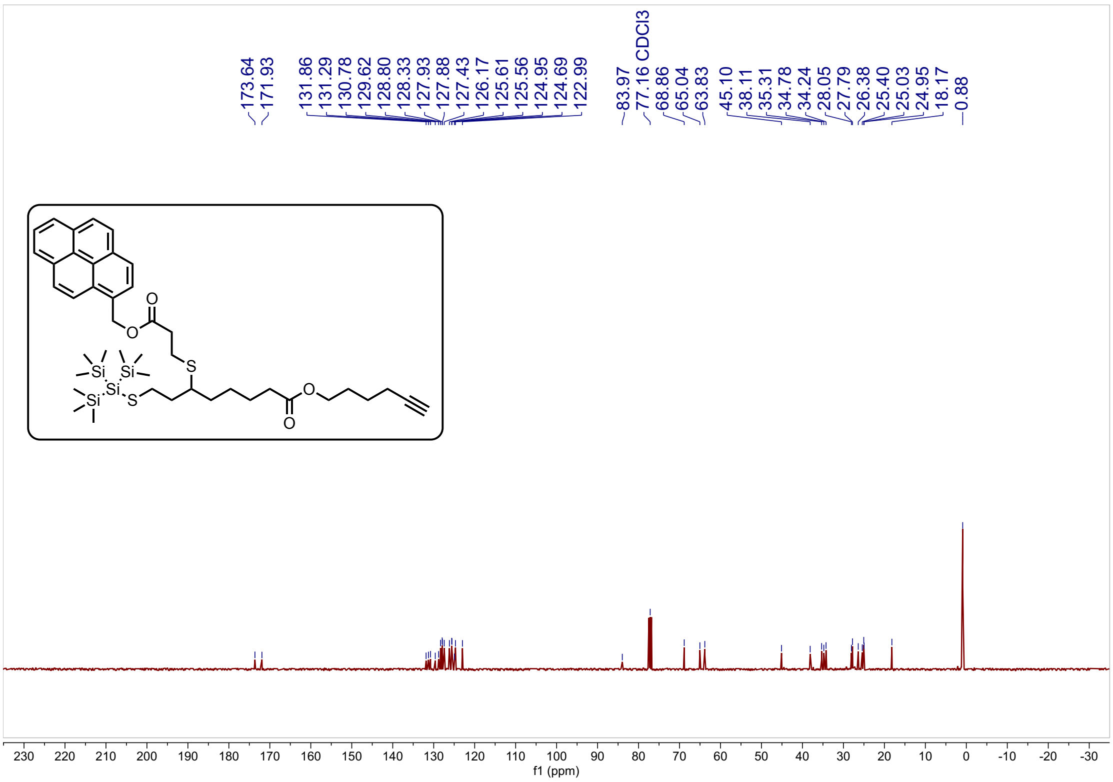

^{29}Si NMR of compound 2z26 (79 MHz, $CDCl_3$)

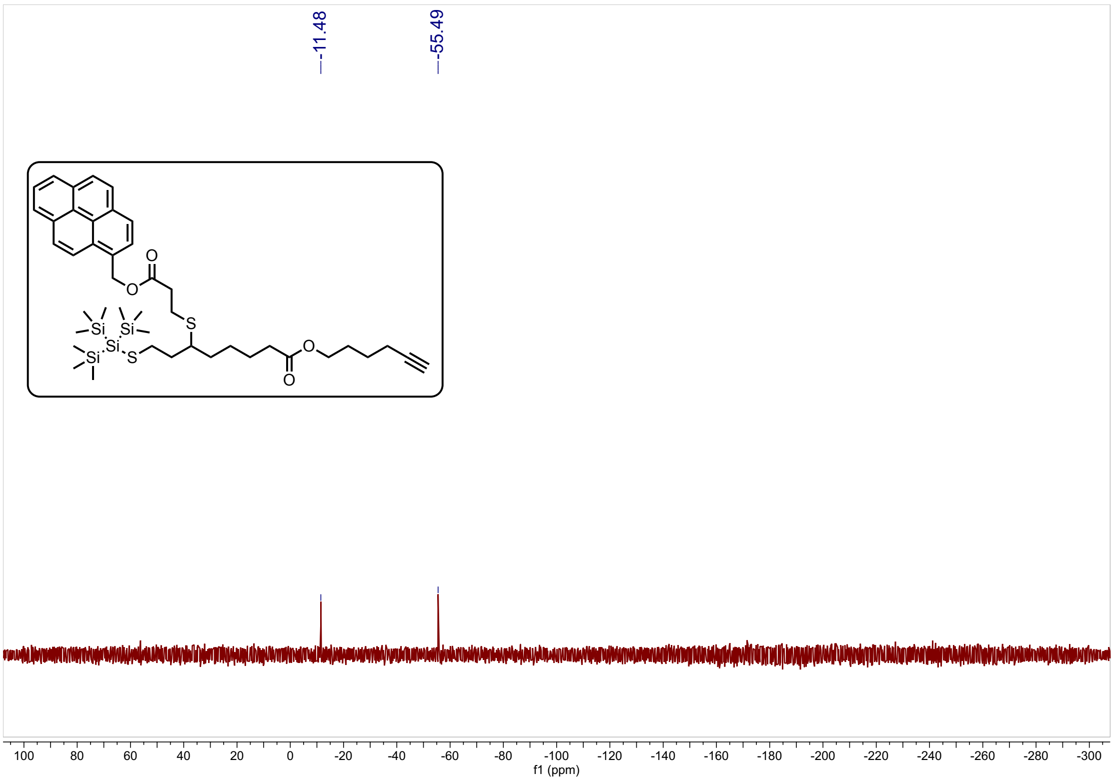

12. References cited in the SI

1. Chen, Q. et al. Metal-free NaI/TBHP-mediated sulfonylation of thiols with sulfonyl hydrazides. *Org. Biomol. Chem.* **16**, 1713-1719 (2018).
2. Levkovskyi, I. O. et al. Lipoic acid-based poly(disulfide)s: Synthesis and biomedical applications. *Nano Trans. Med.* **2**, 100006 (2023).
3. Boyd, D. R. et al. Stereoselective reductase-catalysed deoxygenation of sulfoxides in aerobic and anaerobic bacteria. *Org Biomol Chem* **2**, 554-561 (2004).
4. Zhu, Q. et al. Disulfide radical anion as a super-reductant in biology and photoredox chemistry. *Chem. Sci.* **14**, 6876-6881 (2023).
5. Li, X. et al. Combination of chemotherapy and oxidative stress to enhance cancer cell apoptosis. *Chem. Sci.* **11**, 3215-3222 (2020).
6. Burns, C. J. et al. Synthesis and Characterization of SAMs and Tethered Bilayer Membranes from Unsymmetrically Substituted 1,2-Dithianes. *Aust. J. Chem.* **58**, 738-748 (2005).
7. Miyazawa, T. et al. Synthesis of a novel 1,2-dithianenucleoside via Pummerer-like reaction, followed by Vorbruggen glycosylation between a 1,2-dithiane derivative and uracil. *Chem. Commun.* **49**, 7851-7853 (2013).
8. Liu, Y. et al. Architecture-Controlled Ring-Opening Polymerization for Dynamic Covalent Poly(disulfide)s. *J. Am. Chem. Soc.* **141**, 17075-17080 (2019).
9. Alfaro, J. F. et al. Synthesis of LuxS Inhibitors Targeting Bacterial Cell−Cell Communication. *Org. Lett.* **6**, 3043-3046 (2004).
10. Pickford, H. D. et al. Twofold Radical-Based Synthesis of N,C-Difunctionalized Bicyclo[1.1.1]pentanes. *J. Am. Chem. Soc.* **143**, 9729-9736 (2021).
11. Miura, T. et al. The stereoselective synthesis of α-amino aldols starting from terminal alkynes. *Chem. Commun.* **50**, 10474-10477 (2014).
